# Orchid Species Richness along Elevational and Environmental Gradients in Yunnan, China

**DOI:** 10.1371/journal.pone.0142621

**Published:** 2015-11-10

**Authors:** Shi-Bao Zhang, Wen-Yun Chen, Jia-Lin Huang, Ying-Feng Bi, Xue-Fei Yang

**Affiliations:** 1 Key Laboratory for Economic Plants and Biotechnology, Kunming Institute of Botany, Chinese Academy of Sciences, Kunming, China; 2 Yunnan Key Laboratory for Wild Plant Resources, Kunming, China; 3 Key Laboratory for Plant Diversity and Biogeography of East Asia, Kunming Institute of Botany, Chinese Academy of Sciences, Kunming, China; Consiglio Nazionale delle Ricerche (CNR), ITALY

## Abstract

The family Orchidaceae is not only one of the most diverse families of flowering plants, but also one of the most endangered plant taxa. Therefore, understanding how its species richness varies along geographical and environmental gradients is essential for conservation efforts. However, such knowledge is rarely available, especially on a large scale. We used a database extracted from herbarium records to investigate the relationships between orchid species richness and elevation, and to examine how elevational diversity in Yunnan Province, China, might be explained by mid-domain effect (MDE), species–area relationship (SAR), water–energy dynamics (WED), Rapoport’s Rule, and climatic variables. This particular location was selected because it is one of the primary centers of distribution for orchids. We recorded 691 species that span 127 genera and account for 88.59% of all confirmed orchid species in Yunnan. Species richness, estimated at 200-m intervals along a slope, was closely correlated with elevation, peaking at 1395 to 1723 m. The elevational pattern of orchid richness was considerably shaped by MDE, SAR, WED, and climate. Among those four predictors, climate was the strongest while MDE was the weakest for predicting the elevational pattern of orchid richness. Species richness showed parabolic responses to mean annual temperature (MAT) and mean annual precipitation (MAP), with maximum richness values recorded at 13.7 to 17.7°C for MAT and 1237 to 1414 mm for MAP. Rapoport’s Rule also helped to explain the elevational pattern of species richness in Yunnan, but those influences were not entirely uniform across all methods. These results suggested that the elevational pattern of orchid species richness in Yunnan is collectively shaped by several mechanisms related to geometric constraints, size of the land area, and environments. Because of the dominant role of climate in determining orchid richness, our findings may contribute to a better understanding of the potential effects of climate change on orchid diversity, and the development of conservation strategies for orchids.

## Introduction

The family Orchidaceae is one of the largest and most diverse families of flowering plants, comprising of more than 24,000 species that span 800 genera worldwide [1]. This family is considered to have the highest rate of speciation, but also the highest rate of extinction [2, 3]. The rapid speciation and high species diversity within Orchidaceae is linked to the family’s specialized pollination syndromes, symbiotic associations with mycorrhizal fungi, and colonization of epiphytic habitats [2]. However, because of their mycorrhizal specificity, pollinator specialization and germination limitation, many species are only distributed in specific habitats [2, 4, 5]. Moreover, due to their great economic importance to floral and pharmaceutical industries, many species have been over-collected and are becoming endangered [3–5]. All known orchid species are listed in Appendix II of the Convention on International Trade in Endangered Species of Wild Fauna and Flora (CITES), and are protected by this international convention. Understanding how geographical and environmental gradients influence species richness is essential for the conservation of orchids, and would increase our knowledge about the potential effects of global change on orchids [3, 5, 6]. However, the variability in this diversity along those gradients is rarely examined, especially on a large scale [7, 8].

The relationship between species richness and elevation is a central topic in biodiversity research because of the short geographical distance but large climatic differences found as elevation changes [8–10]. Three patterns have been identified for such correlations: monotonically decreasing pattern with elevation, hump-shaped pattern with high diversity at mid-elevations, or a diversity plateau at low elevations [9, 11, 12]. Nearly 50% of all studies have shown that hump-shaped patterns are the most common [11, 12]. Examples include orchid populations in Nepal and Bhutan [8], and in Nanling National Nature Reserve, China [7]. Those elevational patterns of species richness always depend upon the taxa, region, and scale being examined [11, 13].

Although many hypotheses have been proposed to explain elevational patterns of species richness, most of the attention from ecologists has focused on the mid-domain effect (MDE), species–area relationship (SAR), and water–energy dynamics (WED) [12–16]. The concept of MDE predicts that species richness will peak in the mid-elevation zone because geometric constraints will result in an increasing overlap of species ranges near the midpoint of a mountain [11]. This richness peak has been demonstrated by the distribution of palm trees from sea level to 5030 m in New Guinea [17]. Likewise, in surveying vascular epiphytes along an elevational gradient (30–2600 m) in Costa Rica, Cardelús et al. [18] have noted that the elevational pattern of species richness is substantially influenced by the MDE. However, this effect is not the underlying mechanism for an elevational gradient (0–2400 m) of richness among vascular plants in Crete [13]. The SAR hypothesis is used to explain the upper limit of an elevational gradient of diversity, stating that larger land areas are able to support more species and predicting that maximum richness occurs in elevational zones that cover the largest area [15, 19]. The current reduction in land area is a global elevation-related phenomenon [10] that can affect the elevational pattern of species diversity. Although efficacy of the SAR has been demonstrated in several studies [17, 20], this hypothesis explains only a small portion of the variation in frog species richness in the Hengduan Mountains of China [21]. Moreover, SARs are largely influenced by variables associated with the sampling scheme, spatial scale, and types of organisms or habitats involved [22]. The climatically based WED hypothesis claims that water and energy availabilities together generate and maintain richness gradients because the water supply affects plant productivity and how organisms utilize energy [16, 23]. Both water and energy availabilities are important factors that have been used to explain geographical patterns of species richness for vascular plants [24], woody plants [25], and orchids [6]. However, what determines the regional patterns for WED remains controversial [25].

Many environmental components vary in either a random or non-random fashion along elevational gradients [9, 10]. Because all plant species show some degree of physiological tolerance to environmental stresses, broad-scale fluctuations in species richness are strongly correlated with climate [19]. In fact, some researchers have suggested that the hump-shaped patterns of geographic variation in species richness result from an environmental gradient rather than because of any MDE [15, 26]. One climate–gradient hypothesis predicts that species density fluctuates with local environments, and will peak at the particular elevation where a combination of growing conditions proves optimal for the species of interest [19]. Water and energy seem to be the most likely variables for explaining those elevational patterns [16, 21, 23]. For example, fern richness shows a unimodal response along an energy gradient but a linear response to the moisture gradient [27]. However, only rainfall is significantly correlated with the richness of vascular epiphytic species in Costa Rica [18]. By contrast, the richness of herbaceous species is unrelated to potential evapotranspiration, mean annual rainfall, or moisture index along an elevational gradient in east Nepal [28]. Whereas liverwort richness is primarily controlled by water availability, moss richness is mainly determined by the amount of available energy [29]. These observations suggest that the environmental factors that influence diversity gradients will differ among taxa or regions.

Rapoport’s Rule has been developed to show that species richness is caused by the increasing magnitude of climatic extremes at higher elevations [14]. That is, species in those locations are able to withstand a broad range of climatic conditions while species at lower elevations are adapted to more specific environmental conditions such that they have narrow climatic tolerances [14, 28]. Thus, one can predict a positive correlation between species range size and elevation, and a negative correlation between species richness and elevation [14, 28, 30]. Although Rapoport’s Rule has been successfully applied to many scenarios [13, 31], the richness of tree species along the Himalayan (Nepal) elevational gradients does not support this Rule [30]. Therefore, because none of the hypotheses above has been able to provide a full, accurate explanation for elevational species richness, more evidence is needed for testing them.

Here, we examined the variation in orchid species diversity along large elevational (100–4300 m) and environmental gradients in Yunnan, China, using specimens collected from 1900 to 2009. The following questions were addressed: (1) how does the species richness of different life forms for orchids (i.e., epiphyte, terrestrial, and saprophyte) vary with elevation? and (2) which factors most likely explain that elevational pattern of richness? We hypothesized that species richness peaks at the mid-elevation zone due to an optimal combination of growing environments and an increasing overlap of species elevational ranges, and that a combination of MDE, SAR, WED, Rapoport’s Rule, and climatic factors has helped to shape the elevation-related patterns of species diversity for orchids growing in that mountainous region.

## Materials and Methods

### Study area

Our study area covered the entire province of Yunnan, China (97°31'39"-106°14'47"E, 21°8'32"-29°15'8"N), and encompassed approximately 394,000 km^2^. Yunnan is at the far southern edge of the Tibet Plateau, with elevations that are highest in the northwest (Kawagebo Peak, 6740.0 m) and lowest in the southeast (Red River Valley, 76.4 m). Stretching across approximately 8° of latitude from north to south, the elevational gradient is more than 6,600 m within a 900-km distance. The climate on Yunnan’s south-facing mountain slopes is influenced by both the Pacific and Indian oceans, so that much of the province has mild to warm winters and temperate summers, and an obvious alteration of wet and dry seasons, with over 80% of annual precipitation occurring between May and October. Both mean annual temperature (MAT) and mean annual precipitation (MAP) decrease with rising elevation, while the aridity index (AI) increases with elevation (Figure A in S1 File). This land area hosts a continuous succession from tropical seasonal rain forests to subtropical evergreen broadleaved forests, subalpine conifer forests, temperate deciduous broadleaved forests, and boreal forests. The combined effects of geology, geography, topography, and climate make Yunnan a biodiversity hotspot in the world [32]. Of the approximately 30,000 species of higher plants in China, more than 17,000 are found in that province. Orchids are especially rich there, with an estimated 780 species. Of these, 72 species are endemic [33].

### Data sources

Data on the elevational distribution of native orchid species were compiled from the Chinese Virtual Herbarium (http://www.cvh.org.cn) on July 17, 2014, based on original records submitted for specimens collected from 1900 to 2009. Information included site of collection, elevation, habitat, and life form (S1 File). According to the classification of Acharya et al. [8], all orchid species were divided into three groups: epiphytes, terrestrials, or saprophytes. Although both terrestrial and saprophytic species grow in soils, the saprophytic orchids do not contain chlorophyll and their survival depends entirely upon the nutrients provided by symbiotic fungi. Epiphytes include plants found on trees and rocks, with some species occurring in both habitats. For our research purposes, all data were quality-checked based on Flora Yunnanica [33], and no non-native species was included in our analysis; duplicate specimens or those lacking elevation information or reliable verification were removed from the database. The final tally of specimens used for characterizing elevational patterns was 4202 that belong to 127 genera and 691 species (including 8 varieties and 1 subspecies). Because of the small number of varieties and subspecies, we treated each as a species for the sake of simplicity. These specimens accounted for 94.07% and 88.59% of all orchid genera and species, respectively, found in Yunnan. The ratio of specimens to species (sampling density) varied from 1.0 to 2.5 across elevations (Figure B in S1 File).

### Data analysis

We used the actual frequency of species presence rather than an interpolation to analyze the elevational pattern of richness because the latter method may produce an artificial pattern [34]. The distribution of orchids in our database ranged from 110 m to 4300 m in elevation. Species richness was estimated at 200-m intervals because that distance can produce a much smoother curve when plotting the frequencies of specimens along an elevational gradient. Thus, we used 21 bands of elevation (i.e., 101−300, 301−500, etc.) for our analysis.

The compilation of the available information on species distribution in a territory possibly generates a biased estimation of species richness due to the uneven distribution of the sampling effort performed [35]. Rarefaction is a mathematical method designed to compare species richness between elevations or communities after standardizing to take in account sampling effort [9, 36]. Rarefaction curve is created by randomly re-sampling the pool of N samples multiple times, and then plotting the number of species as a function of the number of samples [37]. Generally, this curve rises very quickly at first and then levels off towards an asymptote as fewer new species are found per unit of individuals collected. If the curve becomes flatter to the right, a reasonable number of individual samples have been taken [37]. The sample-based rarefaction curve was computed by repeated re-sampling, using EcoSim 7.68 [38]. The specimen matrix used in rarefaction analyses is generated by assigning each specimen into its corresponding elevational interval. We used 1,000 simulations to obtain reliable estimates of the rarefied species richness [9]. Rarefaction curves were performed for all species in total as well as for each life form.

All climatic data were obtained from the China Meteorological Data Sharing Service System (http://cdc.cma.gov.cn). Values for MAT and MAP were calculated from regression models derived from the meteorological data of 119 stations in Yunnan Province recorded between 1961 and 2004, and were based on the longitude, latitude, and elevation of each sampling site [39]. Potential evapotranspiration (PET) served as an indicator of energy [23]. Therefore, the aridity index was computed as AI = PET ⁄ MAP while the term ‘water balance’ was defined as the difference in values between MAP and PET.

A planimetric area for each elevational band was calculated according to a global digital elevation model, with a horizontal grid-spacing of 30 arc-seconds, as stipulated from the website ttp://lpdaac.usgs.gov in Envi 4.7 (ITT Exelis, Mclean, VA, USA) and ARCGIS 9.3 (ESRI, Redlands, CA, USA). After the map containing elevation information about Yunnan was extracted from the global GTOPO 30 map, it was converted into a Lambert-Azimuthal equal area projection map and rasterized on 1×1-km grid cells. The number of cells was counted within each 200-m band, based on the elevational value for each cell, and the entire land area for each band was calculated from the total number of cells ([21]; Figure C in S1 File).

The mid-domain effect describes the increasing overlap of species ranges toward the center of a shared, bounded domain due to geometric boundary constraints in relation to the distribution of species range sizes. This produces a peak or plateau of species richness toward the center of the domain. Here, the MDE was tested by RangeModel [40], which offers animated demonstrations of the underlying mechanism and can estimate both interpolated and predicted species richness under “pure” geometric constraints [40]. Richness, as predicted by RangeModel, is mainly affected by the number of elevational bands, total number of species, range size frequency distributions, and frequency distribution of a species distributional midpoint. For this analysis, we used Model 5 (Empirical Frequency Distribution) implemented in RangeModel, which relies upon empirical midpoints and random range sizes. We ran 10,000 Monte Carlo simulations of empirical range sizes sampled without replacement, generating the mean predicted pattern of species richness within each band and producing a 95% confidence interval (CI) for total species and separate life forms (Figure D in S1 File). The predicted species richness was used as a variable in variation partitioning analyses to explore the contribution of the MDE to species richness [13, 17, 20].

The species–area relationship (SAR) predicts the increase in number of species that one might expect to occur over a particular sampling area, and is modelled by a power function [41]. When SARs were modelled for all species and each life form, this power function utilized the logarithm of both sides of the equation to obtain the following linear equation: log(*S*) = log(*c*) + z*log(*A*), where *S* and *A* are the number of species and the area covered by each elevational interval, respectively. Both *c* and *z* are constants [42]. The species richness predicted by SAR was integrated into the variation partitioning analyses to explore the contribution of the SAR to species richness. We also calculated species density for each band as *D* = *S* / Ln (*A*) [12, 13]. Densities were determined for all species in total as well as for each life form.

The water–energy dynamics (WED) hypothesis states that species richness has a positive linear correlation with water, and is a quadratic power function of energy [23]. We evaluated its explanatory power according to the following general linear model: species richness = water + energy − energy^2^. Water was best represented by annual precipitation while PET was used for quantifying energy, as calculated by the FAO Penman–Monteith approach [43]. The species richness predicted by WED was used to explore the impact of WED on species richness in variation partitioning analyses.

The method of variation partitioning based on redundancy analysis ordination (RDA) allows determining the independent and shared influences of multiple complementary sets of hypothesis or variables on species richness [44, 45]. In the present study, we used variation partitioning analysis to decompose the contributions of four hypotheses (SAR, MDE, WED, and climate) to elevational variation in orchid species richness. In the variation partitioning analyses, the species richness predicted by SAR, MDE and WED were used as the variables to explore their contributions to species richness, while the climatic data set used in the RDA consisted of the following variables: MAT, MAP, PET and AI. This process of variation partitioning analyses produced 16 components of variation: 4 components represent variation that can be explained independently by each hypothesis, 11 components represent variation that can be explained by two or three or four hypotheses simultaneously, and 1 component represents unexplained variation. Variation partitioning analyses were conducted in R 3.1.2 for Windows [46], using the function “varpart” in the package ‘‘vegan”. We used Hellinger-transformed abundance values of species as responsible variables, because such transformation allows for the use of RDA techniques to explain the variation of a species matrix containing many zeros [47, 48]. The “varpart” function primarily uses adjusted *R* ^2^ to assess the partitions explained by the explanatory variables and their combinations, because this is the only unbiased method [49]. The shared variation may be negative due to suppressor variables or due to two strongly correlated predictors with strong effects on response variables of opposite signs [49]. We repeated the same analyses for all species in total as well as for each life form.

To investigate the variations of species richness or species density with elevational gradients and climatic variables, we ran polynomial models to determine the relationships between species richness and elevation, MAT, MAP, and AI for total species and for separate life form [31]. When the models were fitted, species richness and species density were used as responsible variables, while elevation, MAT, MAP and AI were used as independent variables, respectively. For all polynomial models, adjusted *R* ^2^ values were used. These analyses were conducted with R 3.1.2 for Windows [46].

Rapoport’s Rule predicts a positive correlation between species range size and elevation [14]. We tested the relationship between mean elevational range size and elevation by the Stevens method [14], Pagel method [50], Mid-Point method [51], and Cross-Species method [52]. These differ from each other primarily in how they compute the mean range size of species for each band. The first three methods calculate the elevational range size as the mean values of range (maximum elevation–minimum elevation) for species within each elevational band. However, the Stevens method counts all species within a given band [14], while the Pagel method includes only those species that have their upper limits in a given band [50], and the Mid-Point method considers only species for which their midpoints occur in a given band [51]. By contrast, the Cross-Species method calculates the elevational range size of each species rather than the mean elevational range size of each band [52]. For some species with only one recorded elevation, that location served as the mid-point, such that the band extends 50 m on either side to represent both upper and lower limits [28]. To understand the impact of Rapoport’s Rule on elevational pattern of species richness, we conducted a linear regression of elevational range sizes and elevations [53]. Using that approach, this Rule should be supported when the relationship between the elevational range sizes and elevations is significantly positive [31].

## Results

### Species composition and orchid habitats in Yunnan

**Fig 1:**
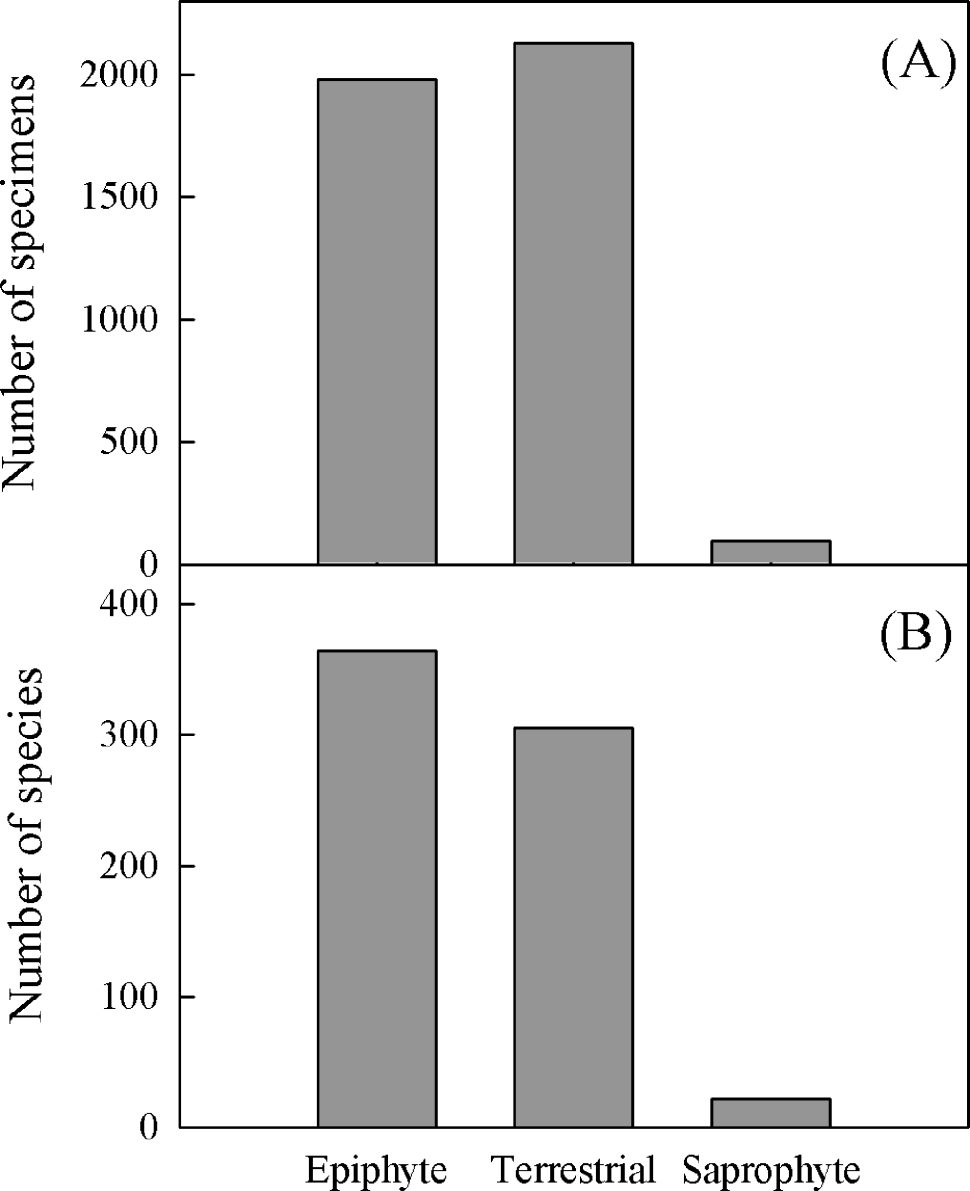
Number of specimens (A) and species (B) within each orchid life form, according to database compiled from Chinese Virtual Herbarium.

**Fig 2:**
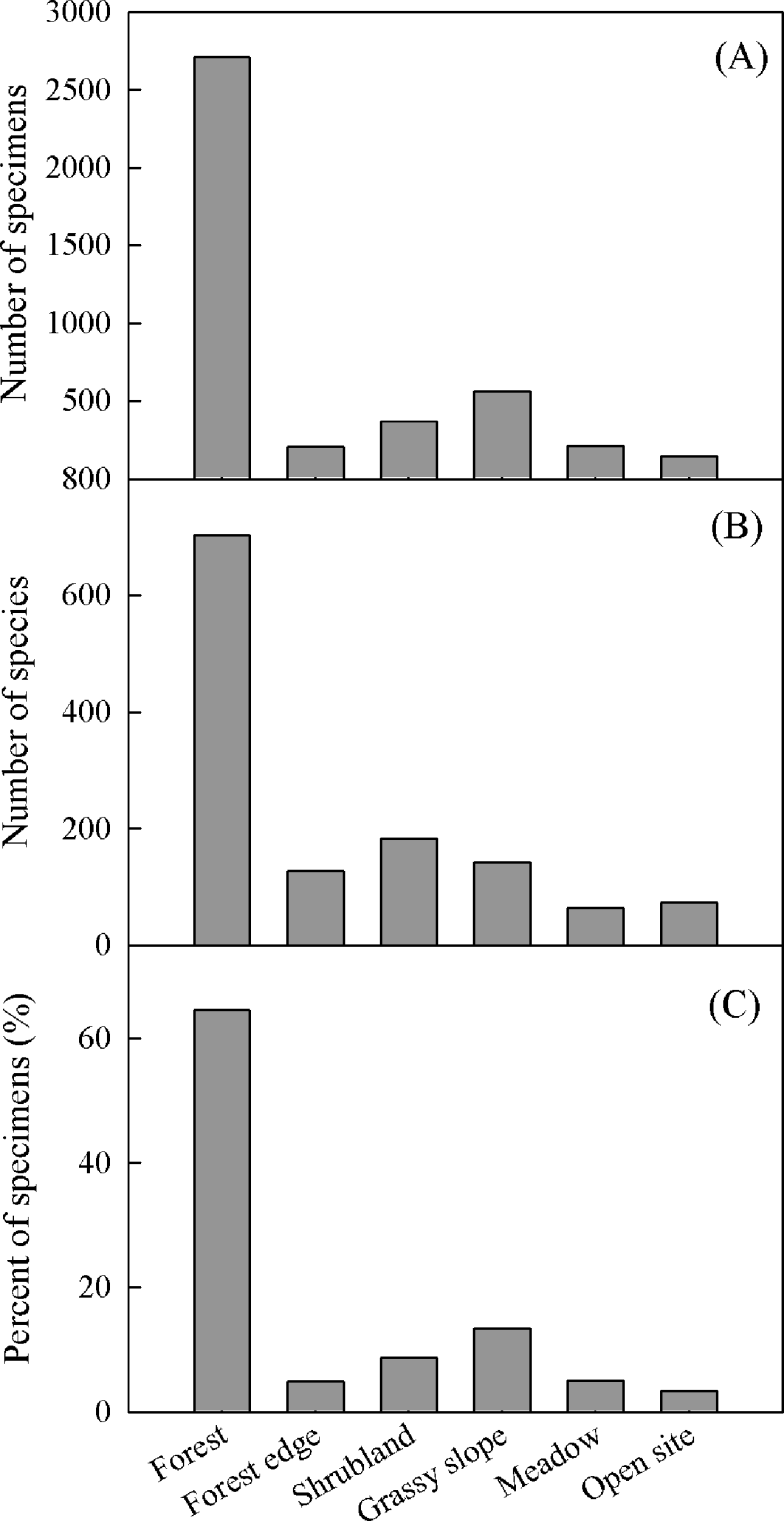
Distribution of orchid specimens (A) and species (B), and proportion of specimens occurring in different habitats (C).

In all, we examined the records for 691 species from 127 genera of the family Orchidaceae in Yunnan. Of these, 364 species were epiphytes, 305 were terrestrials, and 22 were saprophytes (Fig 1). Although all reported species have been classified as epiphytes (52.68% of the total), terrestrials (44.14%), or saprophytes (3.18%), we found that the collected specimens studied here comprised only 47.12% epiphyte, 50.59% terrestrial, and 2.28% saprophyte, thereby indicating a slight difference between specimen-based and species-based proportions of epiphytic and terrestrial forms. Among the 127 genera, 21 contained more than 10 species each (S1 File). The four most species-rich genera were *Dendrobium* (56 species), *Bulbophyllum* (42), *Habenaria* (31), and *Liparis* (30). Although many species were adaptable to various habitat types, 64.51% of all specimens had been collected from forests, 13.37% from grassy slopes, 8.74% from shrublands, and less than 5% each from forest edges, meadows, and open sites (Fig 2).

### Elevational patterns of orchid richness in Yunnan

**Fig 3:**
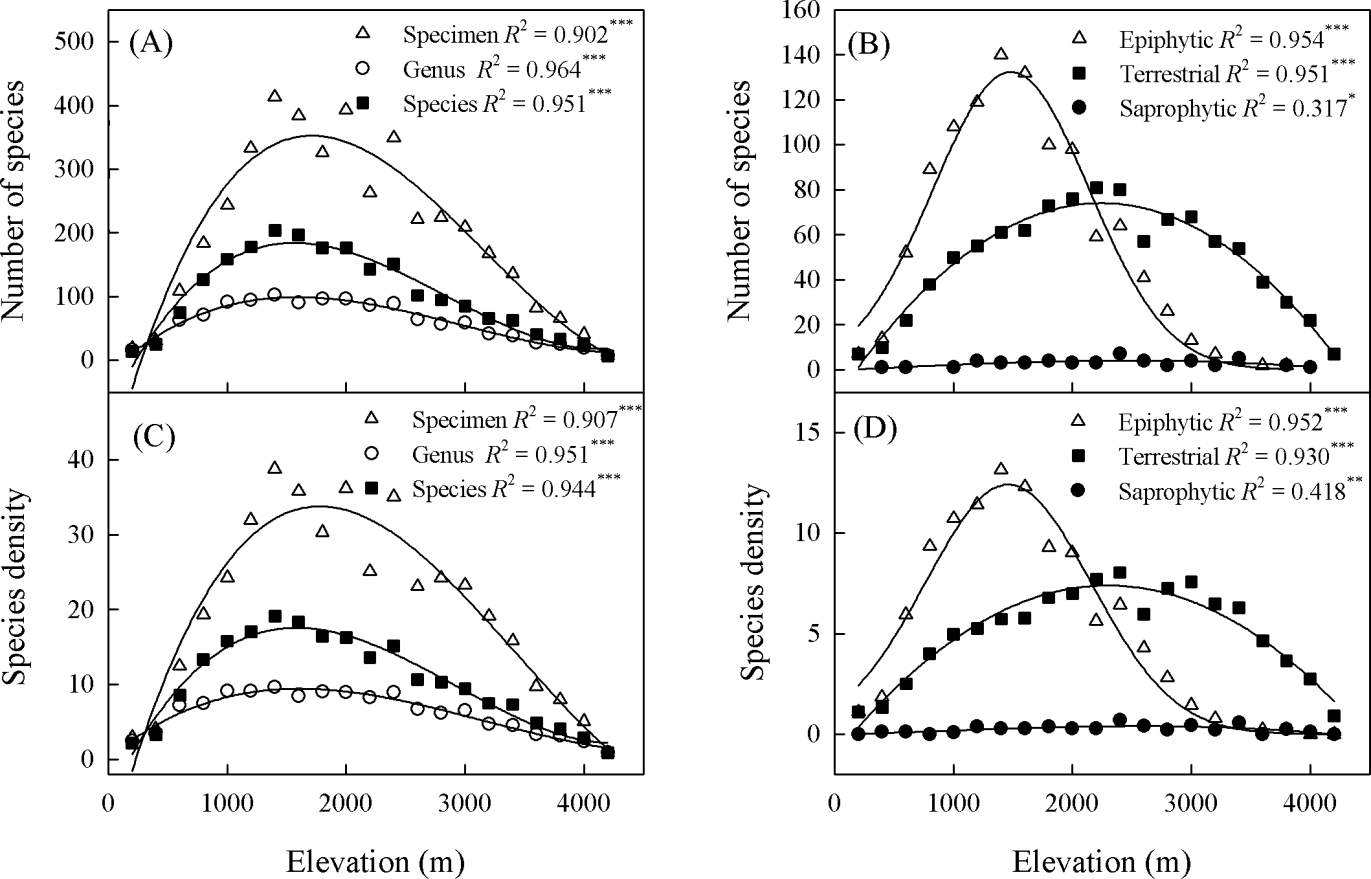
Elevational gradients for orchid species richness (SR, panel A-B) and species density (SD, panel C-D) for individual specimens, genus, and species as well as for each life form recorded in Yunnan, China. (A) Specimen: SR = -172.944 + 0.699*elevation– 3.001*10^−4^*elevation^2^ + 2.941*10^−8^*elevation^3^; Genus: SR = -19.816 + 0.171*elevation– 7.336*10^−5^*elevation^2^ + 8.226*10^−9^*elevation^3^; Species: SR = -81.096 + 0.388*elevation– 2.002*10^−4^*elevation^2^ + 2.008*10^−8^*elevation^3^. (B) Epiphytic: SR = -75.488 + 0.334*elevation − 2.003*10^−4^*elevation^2^ + 2.368*10^−8^*elevation^3^; Terrestrial: SR = -13.720 + 0.078*elevation– 1.719*10^−5^*elevation^2^–4.864*10^−11^*elevation^3^; Saprophytic: SR = -0.369 + 0.003*elevation– 7.203*10^−8^*elevation^2^–1.284*10^−10^*elevation^3^. (C) Specimen: SD = -12.394 + 0.059*elevation − 2.226*10^−5^*elevation^2^ + 2.158*10^−9^*elevation^3^; Genus: SD = 0.188 + 0.013*elevation– 5.423*10^−6^*elevation^2^ + 5.727*10^−10^*elevation^3^; Species: SD = -5.316 + 0.033*elevation − 1.426*10^−5^*elevation^2^ + 1.629*10^−9^*elevation^3^. (D) Epiphytic: SD = -5.232 + 0.028*elevation − 1.408*10^−5^*elevation^2^ + 1.858*10^−9^*elevation^3^; Terrestrial: SD = -0.058 + 0.005*elevation– 2.359*10^−7^*elevation^2^–2.061*10^−10^*elevation^3^; Saprophytic: SD = -0.026 + 2.003*10^−4^*elevation + 5.275*10^−8^*elevation^2^–2.258*10^−11^*elevation^3^.

**Fig 4:**
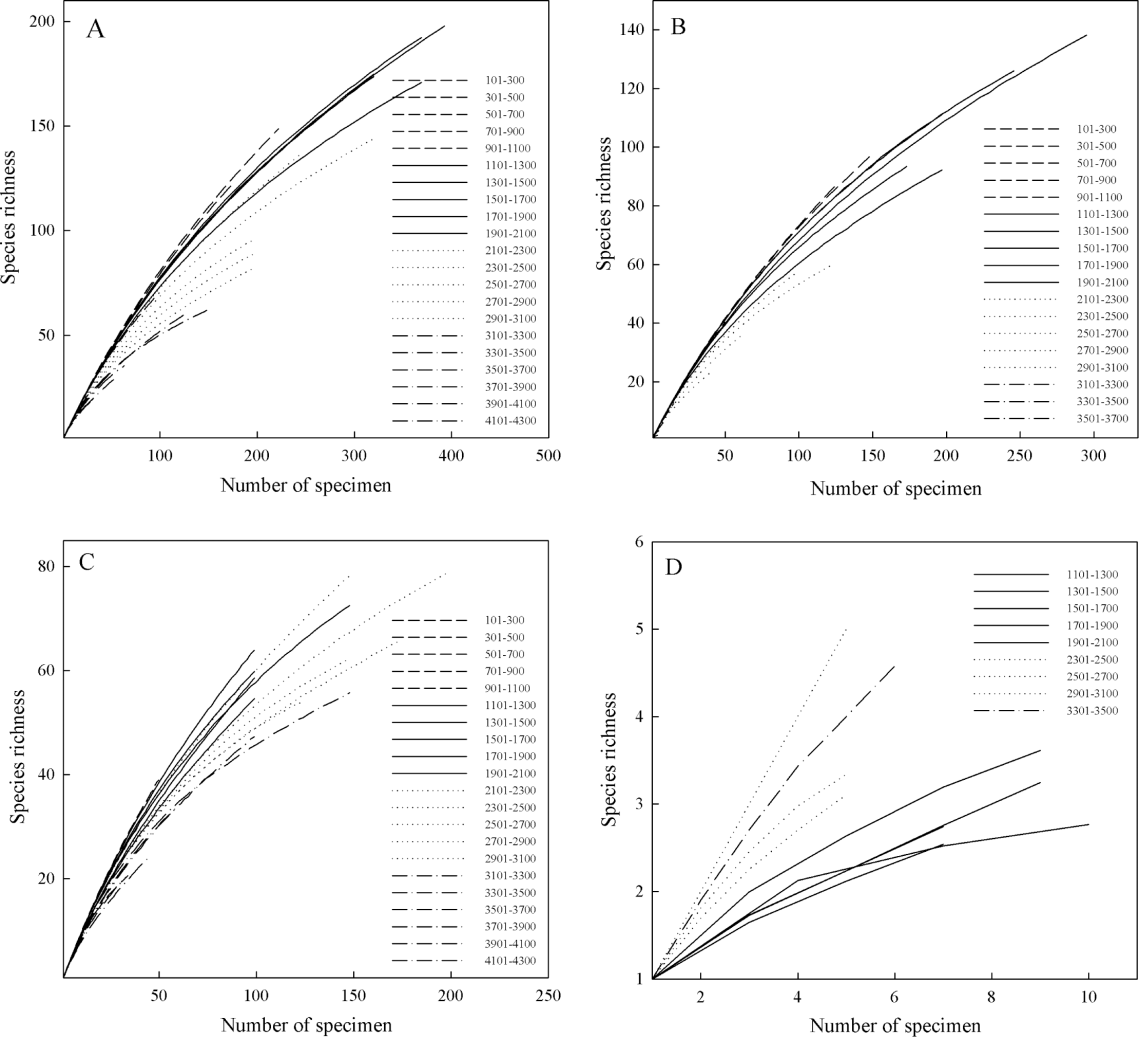
Rarefaction curves for all orchid species (A), and for terrestrial (B), epiphyte (C), and saprophyte (D) life forms in Yunnan, China. The legends refer to the elevational belts for different curves. The curves are the average result of randomly sampling the specimens 1000 times for each elevational interval.

Based on distributions, the elevational patterns of richness were decidedly humped (95% CI), peaking at 1535 to 1865 m for specimen, 1395 to 1723 m for species, and 1418 to 1762 m for genus (S1 File). Species density showed the same pattern as for richness (Fig 3). However, the peaks for richness and density differed significantly among life forms, with epiphyte populations being denser at a lower elevation (<1570 m) when compared with terrestrials (>2050 m) and saprophytes (>2300 m) (Fig 3, S1 File). The estimated species richness based on rarefaction curves peaked at 1501 to 1700 m, 1301 to 1500 m, 2101 to 2300 m and 2301 to 2500 m for total species, epiphytes, terrestrials and saprophytes, respectively (Fig 4). Apart from saprophytes, none of the rarefaction curves reached the asymptotes, and the rarefaction curves at higher elevation were flatter than those at lower elevation for all species, epiphytes, and terrestrials. However, several rarefaction curves for saprophytes at 1101 to 2100 m tended to reach the asymptotes.

### Explanatory powers of MDE, SAR, WED and climate for elevation patterns of richness

**Fig 5:**
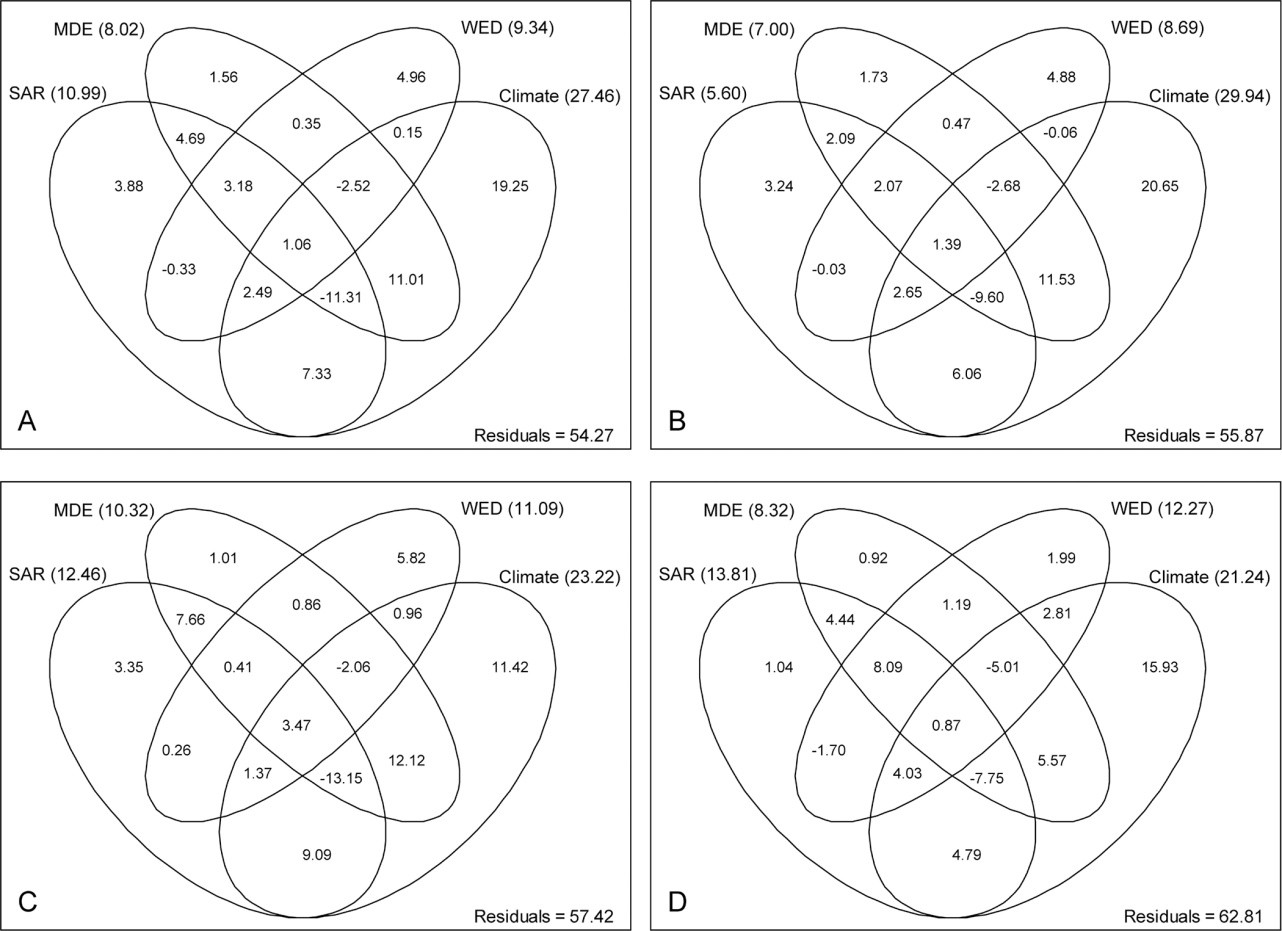
Partitioning of species richness into species-area relationship (SAR), mid-domain effect (MDE), water-energy dynamics (WED) and climate components all orchid species (A), and for epiphyte (B), terrestrial (C), and saprophyte (D) life forms in Yunnan, China. Values in the fractions represent adjusted *R* ^2^ coefficients of the independent or shared effects of the four hypotheses. The total effect (including the independent and shared effects) of each explanatory component is shown in parentheses.

Variation partitioning showed that the MDE, SAR, WED and climate together explained 45.73%, 44.13%, 42.58% and 37.19% of the variance for total species, epiphytic, terrestrial, and saprophytic life forms, respectively, while climate accounted for the largest fractions of explained variation (Fig 5). However, the explanatory powers of four predictors differed among species groups. For total species, the independent contributions of SAR, MDE, WED and climate to species richness were 3.88%, 1.56%, 4.96% and 19.25%, respectively, while the total contributions (including independent and shared effects) of SAR, MDE, WED and climate were 10.99%, 8.02%, 9.34% and 27.46%, respectively. Both independent and shared effects of climate on species richness for epiphytic orchids were larger than terrestrial and saprophytic orchids, but the total contributions of SAR and WED to species richness were lower in epiphytic life form than terrestrial and saprophytic life forms. The explanatory power of MDE for terrestrial orchids was higher than for epiphytic and saprophytic orchids. However, the unique effect of MDE was low (<1.8%) for all species groups.

**Fig 6:**
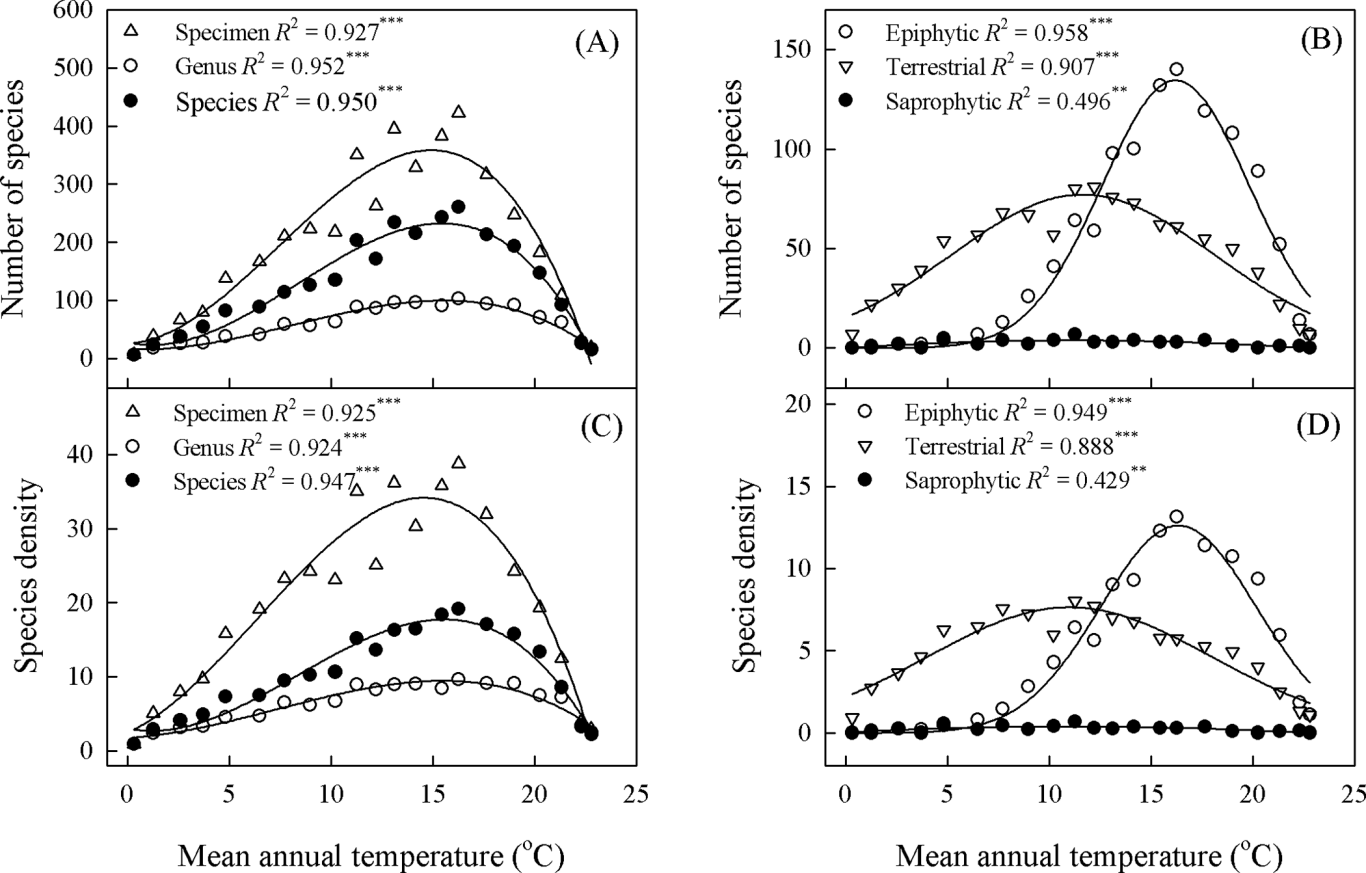
Variations in orchid species richness (SR, panel A-B) and species density (SD, panel C-D) as function of mean annual temperature (MAT). (A) Specimen: SR = 25.376 + 1.408*MAT − 4.304*MAT^2^ − 0.194*MAT^3^; Genus: SR = 16.733 − 1.682*MAT + 1.247*MAT^2^ − 0.051*MAT^3^; Species: SR = 28.621 − 8.129*MAT + 3.611*MAT^2^ − 0.144*MAT^3^. (B) Epiphytic: SR = 24.283 − 22.077*MAT + 3.673*MAT^2^ − 0.120*MAT^3^; Terrestrial: SR = 6.125 + 10.632*MAT– 0.358*MAT^2^–0.005*MAT^3^; Saprophytic: SR = -0.248 + 0.798*MAT– 0.042*MAT^2^ + 3.002*10^−4^*MAT^3^. (C) Specimen: SD = 2.468 + 1.220*MAT + 0.280*MAT^2^ − 0.015*MAT^3^; Genus: SD = 1.776 + 0.143*MAT + 0.078*MAT^2^ − 0.004*MAT^3^; Species: SD = 3.044 − 0.561*MAT + 0.254*MAT^2^ − 0.010*MAT^3^. (D) Epiphytic: SD = 2.211 − 1.990*MAT + 0.336*MAT^2^ − 0.011*MAT^3^; Terrestrial: SD = 0.853 + 1.333*MAT– 0.075*MAT^2^ + 0.001*MAT^3^; Saprophytic: SD = -0.021 + 0.096*MAT − 0.006*MAT^2^ + 9.571*10^−5^*MAT^3^.

**Fig 7:**
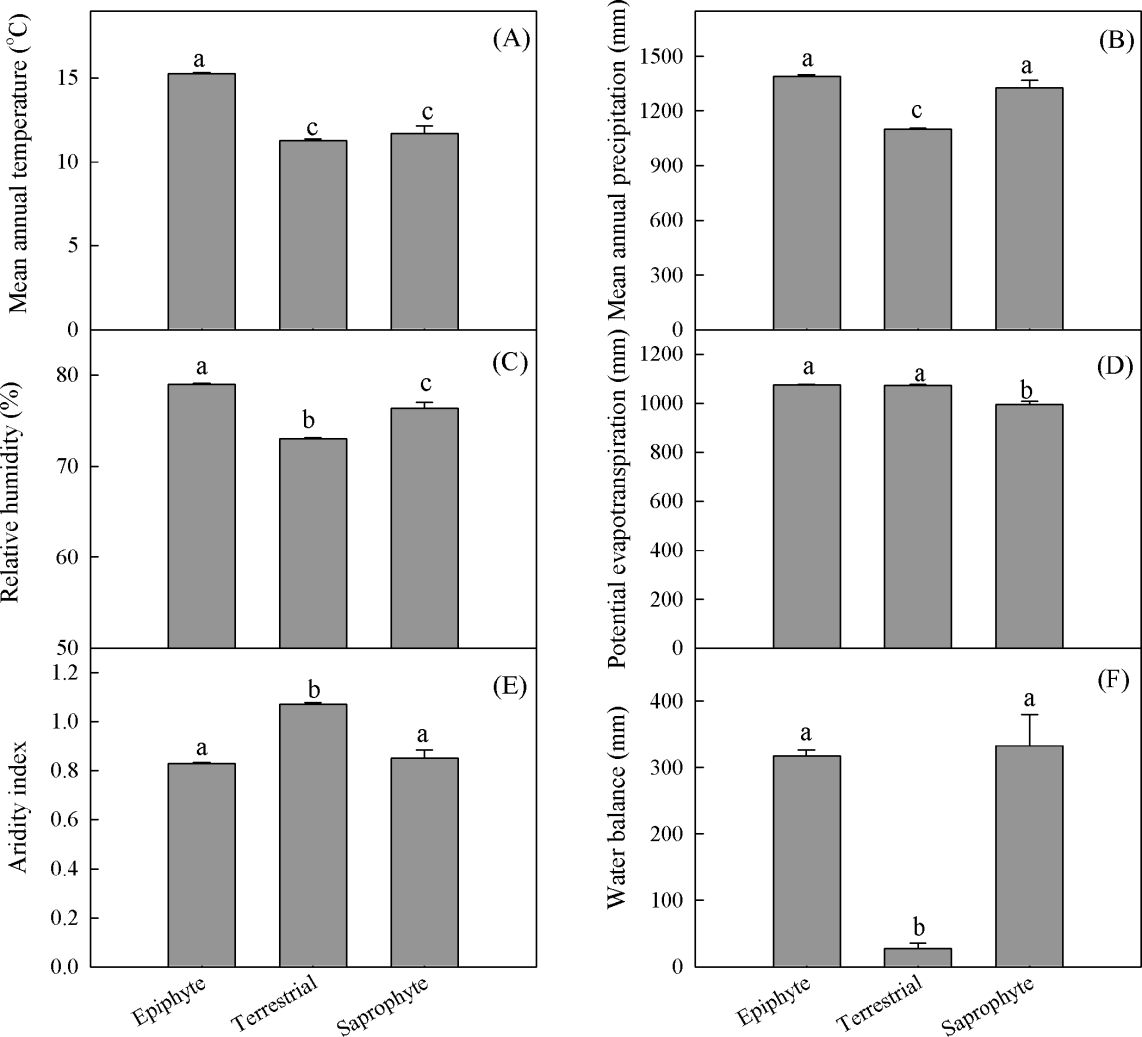
Differences in mean annual temperature (A), mean annual precipitation (B), relative humidity (C), potential evapotranspiration (D), aridity index (E), and water balance (F) associated with epiphytic, terrestrial, and saprophytic orchids. Different letters in each panel indicate significant differences among life forms at *P* <0.05.

For all species combined and for separate life forms, species richness and density varied significantly with MAT (95% CI) and showed unimodal patterns along temperature gradients (Fig 6). Densities for all species, as well as for epiphytes, terrestrials, and saprophytes separately, peaked at 13.5 to 18.1°C, 15.0 to 17.5°C, 9.9 to 12.1°C, and 9.7 to 12.1°C, respectively (Fig 6, S1 File). Epiphytes had higher MAT values when compared with terrestrials or saprophytes (Fig 7).

**Fig 8:**
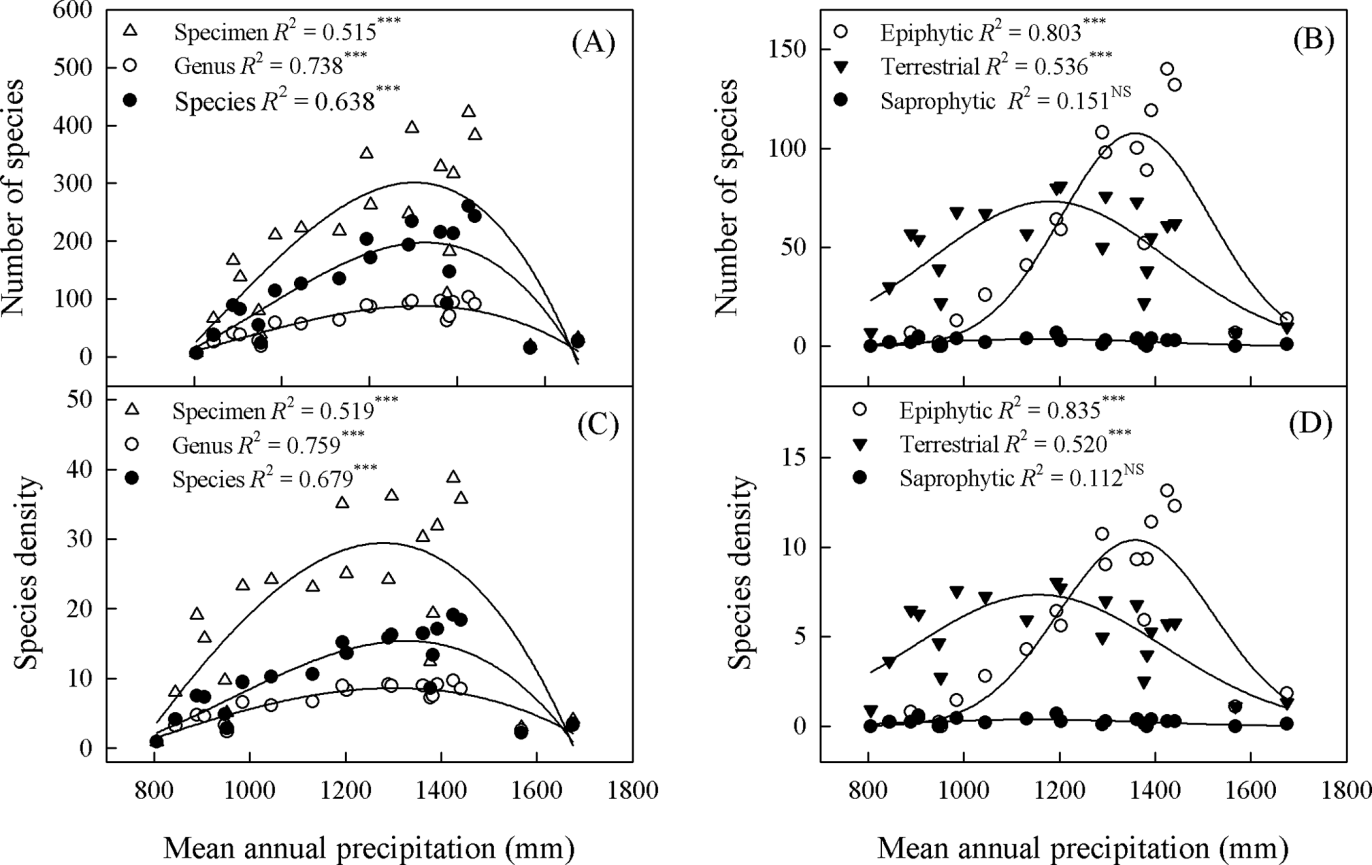
Variations in orchid species richness (SR, panel A-B) and species density (SD, panel C-D) as function of mean annual precipitation (MAP). (A) Specimen: SR = 215.161 − 2.100*MAP + 0.003*MAP2 − 1.316*10^−6^*MAP^3^; Genus: SR = 69.849 − 0.586*MAP + 0.001*MAP^2^ − 3.545*10^−7^*MAP^3^; Species: SR = 661.146 − 2.722*MAP + 0.003*MAP^2^ − 1.149*10^−6^*MAP^3^. (B) Epiphytic: SR = 1678.796 − 4.881*MAP + 0.005*MAP^2^ − 1.339*10^−6^*MAP^3^; Terrestrial: SR = -817.875 + 1.874*MAP– 0.001*MAP^2^ + 2.523*10^−7^*MAP^3^; Saprophytic: SR = -55.403 + 0.132*MAP– 9.505*10^−5^*MAP^2^ + 2.162*10^−8^*MAP^3^. (C) Specimen: SD = -52.167 − 0.004*MAP + 2.012*10^−4^*MAP^2^ − 8.056*10^−8^*MAP^3^; Genus: SD = -11.453 − 0.005*MAP + 4.268*10^−5^*MAP^2^ − 2.090*10^−8^*MAP^3^; Species: SD = 47.612 − 0.194*MAP + 2.021*10^−4^*MAP^2^ − 8.252*10^−8^*MAP^3^. (D) Epiphytic: SD = 151.873 − 0.444*MAP + 4.011*10^−4^*MAP^2^ − -1.229*10^−7^*MAP^3^; Terrestrial: SD = -97.684 + 0.234*MAP– 2.002*10^−4^*MAP^2^ + 3.751*10^−8^*MAP3; Saprophytic: SD = -6.578 + 0.016*MAP − 1.217*10^−5^*MAP^2^ + 2.922*10^−9^*MAP^3^.

**Fig 9:**
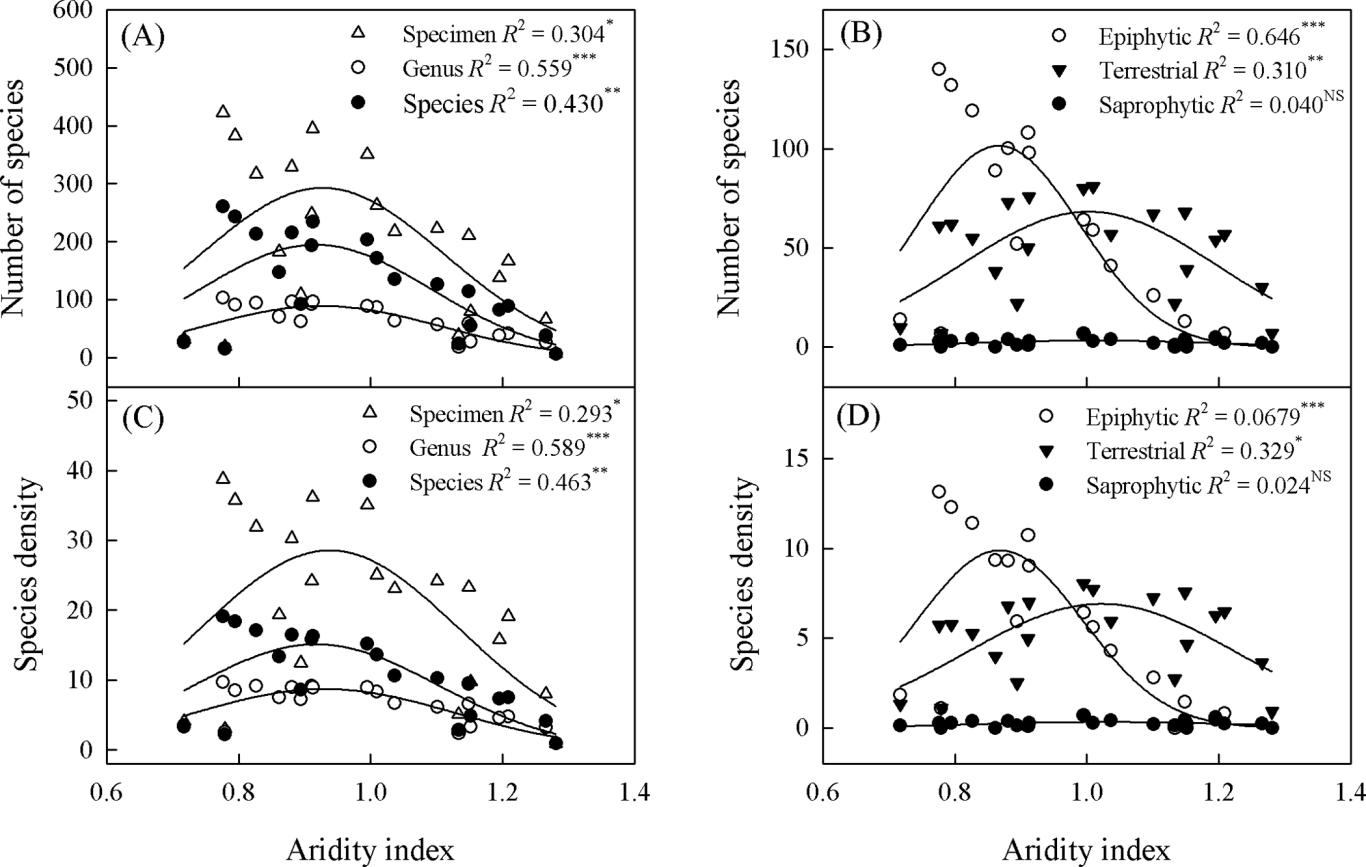
Variations in orchid species richness (SR, panel A-B) and species density (SD, panel C-D) as function of aridity index (AI). (A) Specimen: SR = -7915.83 + 23390.57*AI − 21631.42*AI^2^ + 6422.12*AI^3^; Genus: SR = -2583.24 + 7696.96*AI − 7211.44*AI^2^ + 2176.77*AI^3^; Species: SR = -457.22 + 1371.72*AI − 1296.08*AI^2^ + 394.95*AI^3^. (B) Epiphytic: SR = -4895.03 + 15199.08*AI − 15093.54*AI^2^ + 4853.48*AI^3^; Terrestrial: SR = -487.48 + 1045.23*AI– 435.60*AI^2^–55.98*AI^3^; Saprophytic: SR = 5.08 − 36.50*AI + 65.40*AI^2^–30.80*AI^3^. (C) Specimen: SD = -630.60 + 1833.93*AI − 1649.22*AI^2^ + 472.65*AI^3^; Genus: SD = -201.64 + 597.98*AI − 551.41*AI^2^ + 163.02*AI^3^; Species: SD = -457.22 − 457.22*AI − 1296.08*AI^2^ + 394.95*AI^3^. (D) Epiphytic: SD = -462.81 + 1436.61*AI − 1424.98*AI^2^ + 457.52*AI^3^; Terrestrial: SD = 2.16 − 52.26*AI + 113.41*AI^2^ − 56.58*AI^3^; Saprophytic: SD = 3.43 − 12.62*AI + 15.49*AI^2^ − 5.98*AI^3^.

The availability of water, as represented by MAP and AI, had a significant impact (95% CI) on richness or density for all species and for the separate categories of epiphytes and terrestrials, but had no such effect on saprophytes (Figs 7–9). Compared with terrestrials, epiphytes had higher values for MAP and water balance (Fig 7). Richness peaked at MAP levels of 1237 to 1414 mm, 1309 to 1411 mm, and 1099 to 1259 mm for all species combined, epiphytes, and terrestrials, respectively (Fig 8, S1 File). By contrast, epiphytes had lower AI values than terrestrials. Richness for terrestrial orchids peaked at an AI of 0.94 to 1.06, while epiphytic orchids peaked at an AI of 0.83 to 0.91 (Fig 9, S1 File).

### Rapoport’s Rule in relation to elevation patterns of orchid richness

**Fig 10:**
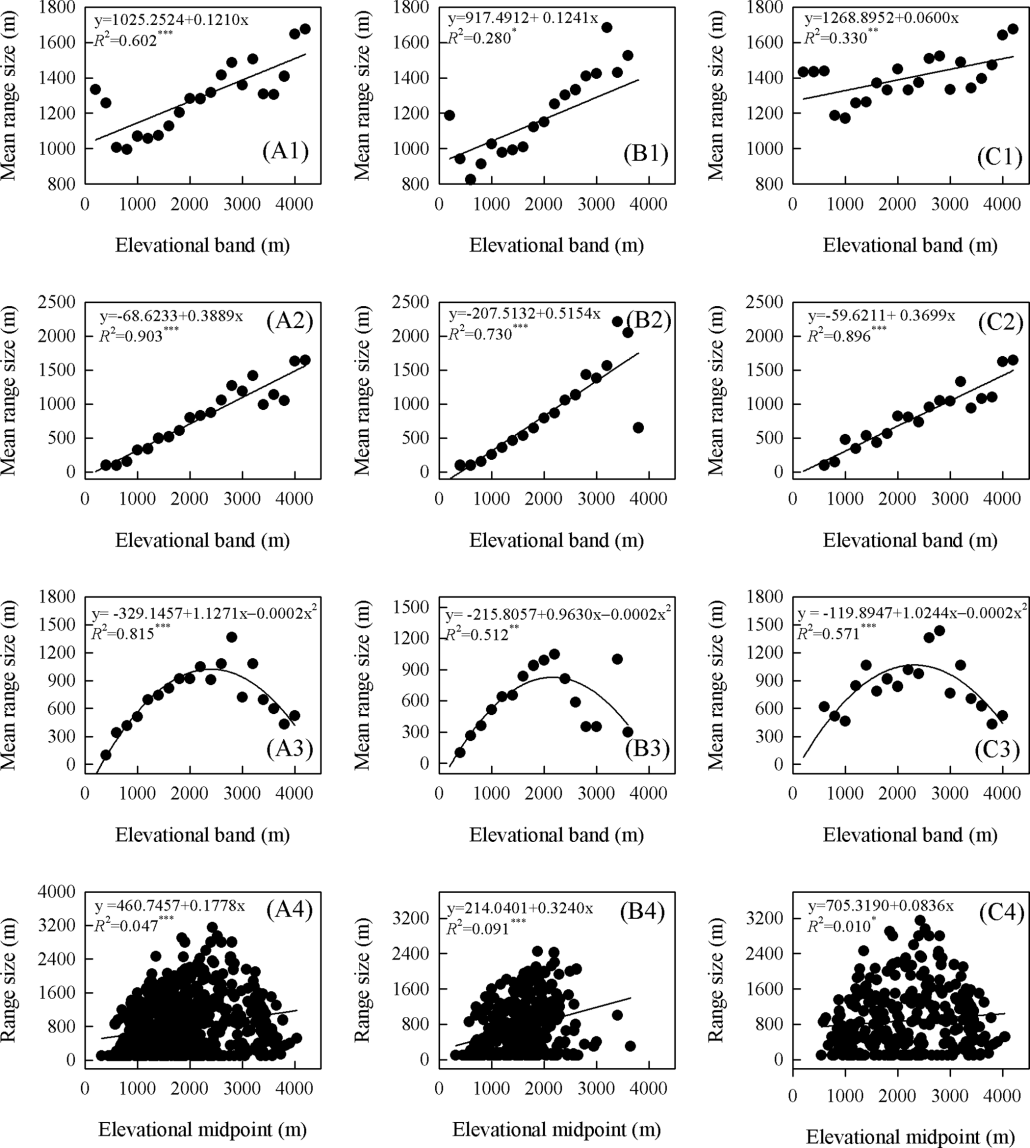
Elevation-related patterns of range size for all species (A), and for epiphyte (B) and terrestrial (C) forms. Suffixes in each panel indicate analytical method: 1, Stevens; 2, Pagel; 3, Mid-Point; and 4, Cross-Species.

When Rapoport’s Rule was applied, the mean range sizes for all species, epiphytes, and terrestrials presented significantly linear correlations with elevation when tested by the Stevens, Pagel, and Cross-Species methods (Fig 10). Although the mean range size increased with elevation, testing via the Mid-Point method indicated that the mean range sizes of species for all groups showed curvilinear correlations rather than linear correlations with elevation (Fig 10). This suggested that Rapoport’s Rule was supported by the first three methods, but not by the Mid-Point method.

## Discussion

### Patterns of elevation-related diversity

We reviewed 691 orchid species from 127 genera, which comprise 88.59%% of all known orchids in Yunnan [33]. This confirmed that our findings are fairly representative of the orchid species richness in Yunnan, where the response to elevation was humped, peaking at 1418 m to 1762 m. Previous studies have suggested that spatial patterns of species richness may be affected by sampling effort [29, 37]. In the present study, richness based on rarefaction curves showed a similar elevational pattern to that for richness based on either species numbers or specimen numbers. We found that sample density did not vary greatly across elevational zones. These indicated that sampling effort did not significantly influence the elevational patterns of richness. Apart from saprophytes, none of the rarefaction curves reached the asymptotes, indicating that there were still species to be discovered in every elevational band. The incomplete sampling effort may be common at a large scale [9]. However, because the small amount of specimen and species (Fig 1), saprophytes have little contribution to the entire species richness. Similar elevational patterns of richness have been reported for many other habitats and plant species [12, 18, 27, 54]. These include the relationship between orchid diversity and elevation in Nepal and Bhutan, where maximum richness occurs at 1600 m [8]. However, some researchers found that richness declines monotonically with elevation [13, 17, 55]. This discrepancy is probably caused by variations in sampling techniques, regional sizes, and the taxa that are studied [11, 13].

Approximately 50% of the orchid species recorded in Yunnan is epiphytic, a proportion that is significantly lower than the worldwide estimate of 73% calculated for this life form [56]. This contrast is probably due to a smaller area of the tropical zone being examined in the current study, because orchid populations are the most diverse in tropical regions [5, 56]. In our investigation, the elevational patterns of richness were the same for epiphytes and terrestrials as for the pooled species, but richness values peaked at a higher elevation for terrestrials (2052−2598 m) than for epiphytes (1367−1563 m). In Coast Rica, the maximum species richness for epiphytic ferns occurs at 1000 m while that for terrestrial ferns peaks at a mid-elevation level and remains relatively constant at higher elevations (1000−2600 m) [57]. Therefore, our findings demonstrated that elevational patterns of orchid species richness are dictated by life form. Furthermore, the mechanism controlling these patterns likely involves a combination of factors related to biology, environmental conditions, and geometric constraints on geographical ranges [57].

### Influence of MDE, SAR, WED and climate on elevation-related patterns

We noted that the mid-domain effect, species–area relationship, water–energy dynamics and climate considerably influenced the elevational patterns of orchid richness. Among those four, climate was the strongest predictor for total species as well as for separate life forms, while MDE was the weakest. The extent to which MDE can effectively explain spatial patterns of richness is quite controversial because it fails to exclude the influence of environment [26, 58]. Some studies have suggested that the MDE is not the main underlying mechanism that determines the richness of vascular plants [13, 59], while numerous studies have produced evidence for a potential role by MDE in the elevational gradients of richness, particularly for wide-ranging species [13, 18, 58]. In the present study, the sum of the unique and shared effects explained by MDE was more than 7% (Fig 5), but the unique contribution of MDE to species richness was low. This indicated that the MDE is not a crucial mechanism for explaining the elevational pattern of orchid richness in Yunnan.

Land area may play an important role in shaping elevational patterns because a larger space can accommodate more species [13, 19]. Increased habitat heterogeneity and resource availability from a larger area also contribute to higher species diversity [60]. The effect of land area on elevation-related richness has been documented in several studies [13, 21]. Bachman et al. [17] found that, at different elevations, land area greatly influences the richness of palm species in New Guinea. However, area is a poor predictor of elevational fern diversity in Costa Rica [58] and bryophyte richness in eastern China [29]. These discrepancies may be linked to the tested scale because SARs are scale-dependent [22]. Some environmental factors (e.g., temperature) associated with elevation might also dramatically diminish the effect of area [10]. We found that SAR explained a certain portion of the variance for each life form, but the contribution of SAR to epiphytic orchid diversity was lower than that for terrestrial and saprophytic orchids (Fig 5). This is because most of epiphytes grow on trees. Thus, land area is a determinant of the elevational pattern for orchid species richness in Yunnan.

Although WED predicted species richness for all life forms, it was especially powerful for terrestrial and saprophytic species. Previous studies have found that WED has an important function in explaining spatial variations in plant diversity [16, 23]. For example, orchid species richness is closely correlated with the availability of environmental energy and water in China [6]. This availability, when associated with geographical positioning, may affect the photosynthesis and physiological processes of plants, and, therefore, the geographical patterns of species diversity [23].

Climate was the strongest predictor for explaining orchid species richness in Yunnan. Previous studies have suggested that the hump-shaped patterns of geographic variation in species richness mainly result from an environmental gradient [15, 26]. We identified a unimodal relationship between species richness and MAT, with epiphytes preferring a higher temperature (15.0−17.4°C) than terrestrials (10.1−13.3°C) or saprophytes (9.9−12.9°C). A recent study has also shown that the mean temperatures during the warmest and coldest months provide a good explanation for orchid richness in China [6]. However, Watkins et al. [57] found little evidence that environmental gradients drive elevational patterns of fern diversity in Costa Rica. A decline in richness at higher elevations is a consequence of low temperatures [54] while an increase in species diversity as the average temperature rises is mainly due to the effect of heat on productivity [11]. In addition, different orchid life forms vary in their responses to MAT, which ultimately changes the balance of species composition between epiphytic and terrestrial orchids under different temperature regimes.

Water availability significantly influences the richness and composition of orchid species. When compared with terrestrials, epiphytes are associated with higher MAP values but lower AI values. However, Zhang et al. [6] did not find that the species richness of epiphytic orchids is more sensitive than terrestrial orchids to water availability. Water-adaptive strategies tend to differ between those two forms, with the relationship between humidity level and plant growth being tighter for epiphytes because they must depend more upon moisture supplied from precipitation and fog [29, 61, 62]. The species richness of liverworts and moss is also primarily controlled by water availability [29]. A mid-elevation peak is usually observed in mean annual rainfall [63], and higher humidity at mid-elevations is more suitable for maintaining a greater richness of epiphytes [64].

We determined that species richness showed a parabolic response to MAP, peaking at 1309 to 1411 mm and 1099 to 1259 mm for epiphytes and terrestrials, respectively. In Mexico, the diversity of vascular epiphytes (orchids, bromeliads, and ferns) declines when MAP exceeds 2500 mm, perhaps because wind-dispersed epiphytes have difficulty becoming established during frequent downpour events [65]. Many orchids are characterized by velamina of multiple layers of dead cells that can absorb and store water. However, when the velamen cell lumens are completely filled by water, the increased diffusive pathway between the atmosphere and root core may severely impede gas exchange [66]. Because the root anatomy of orchids might have evolved to adapt to distinct environments [66], it is reasonable to assume that their succulent roots have low tolerance to high soil water contents. This would support the theory that orchids have evolved from a terrestrial to an epiphytic form.

During the past five decades, MAT values for Yunnan Province have increased at a rate of 0.3°C/decade while the evapotranspiration and relative humidity have decreased [67, 68]. Although MAP for that region showed a non-statistically significant decline between 1961 and 2008 [69], extreme climatic changes, e.g., extensive drought periods, have become more frequent in recent years [70]. In fact, climate warming has had a great influence on the survival of *Cymbidium sinense* in wild populations [71]. These phenomena are of particular importance when considering the effects of global warming on orchid populations [5]. Climate warming may cause species’ elevational range shifts and occasional extinctions [72, 73], because a species’ range is at least partly determined by its environmental requirement and tolerance [74]. A previous study has found that climate warming drives geographical range shifts of 87% endemic plant species in the Sikkim Himalaya, and the upper range extensions of species increase species richness in the upper alpine zone [73]. Thus, it was reasonable to speculate that global warming may result in the uplift of the peak for orchid richness in Yunnan, although the effects of climate change on orchid populations require further investigation.

Apart from climatic factors, habitat also influences species richness [65, 75]. Vascular epiphytes often show clear habitat preferences [56]. Previous studies have found that epiphyte diversity is significantly higher in sites with canopy soil or a moss mat than on bare bark [75], and the distribution and density of epiphytic orchids are positively associated with bryophyte species richness [76]. Riverine forests can provide the most hospitable habitats for the growth and development of epiphytic orchids [77]. For our study, the majority (64.51%) of orchids occurred in forests (Fig 2). In the Gaoligong Mountains in Yunnan, species richness for trees peaks at an elevation of about 2,000 m [12], which was similar to the elevation for total orchid species, but slightly higher than that for epiphytic orchids in the same region (Figure E in S1 File). Meanwhile, the species richness peaked at a lower elevation for epiphytic orchids than for terrestrial orchids (Fig 3), and the proportion of epiphytes tended to decrease with increasing elevation. In Yunnan, the upper limit of tree growth attains elevations of ca. 4600m [78], but no epiphytic orchid was found over 3,800 m above sea level (S1 File). Only 20 epiphytic orchids grow on trees (rhododendron) or rocks above an elevation of 3,000 m. These indicated that orchid (especially epiphytic) species richness was related to tree development, and epiphyte richness decayed before and faster than terrestrial richness.

Human activity has an impact on the elevational gradient of species richness [7, 59, 79]. The interdependence between montane ecosystems and human activities has led to a reduction in natural lowland habitats [79]. A previous study has found that disturbed habitats harbor fewer fern and orchid species but more bromeliad species than do primary forests [80]. Because the growth and reproduction of epiphytic orchids strongly depend upon the existence of host trees, and orchids are of great economic value to floral and pharmaceutical industries, species richness may be affected by over-collection and habitat destruction [3, 4, 6]. In the present study, because no non-native species was included in our analysis, and the re-introduction of orchid to the wild is rare, the orchid species richness would not be over-estimated. Moreover, although the strongest human activity in Yunnan mainly occurred at the mid-elevation zone, the maximum species richness for orchids still was found at this elevational range (1395 to 1723 m). A previous study also confirmed that maximum orchid diversity occurs at 1600 m in Nepal and Bhutan [8]. Thus, up to now, human activity would have a limited influence on the elevational pattern of orchid richness in Yunnan. However, conserving the entire ecosystem *in situ* may be of particular importance when sustaining orchid diversity.

Elevational gradients in species diversity may result from a combination of ecological and evolutionary processes, rather than from the independent effect of one overriding force [19]. Although a geometric model might explain much of the pattern that develops in species richness, the predicted peaks in richness as well as an overlap in favorable environmental conditions all coincide at middle elevations [58]. The appropriate environmental factors associated with those positions contribute to the hump-shaped diversity patterns [54, 58]. Körner [10] has suggested that, although some factors such as temperature are physically linked to the number of meters above sea level at which a site is located, other factors (such as moisture) are not generally elevation-specific. For different mountains in Yunnan, some climatic variables do not vary with elevation to the same extent even though both temperature and precipitation generally tend to decrease with increasing elevation (Figure A in S1 File). This is because the mountain slopes there are south-facing, topography inclines from northwest to southeast, and the latitudinal span is small in that region. This makes temperature and precipitation two reliable climatic variables for explaining the elevational pattern of species richness. However, a comprehensive analysis of boundary constraints and environmental factors is probably a better approach to take if one is to explain such richness patterns [9].

### Effect of Rapoport’s Rule on elevation-related species richness

In our investigation, Rapoport’s Rule played a considerable role in explaining the elevational patterns of richness for total species, and for the separate classes of terrestrials and epiphytes in Yunnan. This was demonstrated by the positive correlations between range size and elevation when tested by the Stevens, Pagel, and Cross-Species methods. Our results were consistent with those previously reported [31]. However, findings by Trigas et al. [13] only partially conformed to Rapoport’s Rule for explaining the elevational gradient of species richness in Crete, while those by others provided no support at all for this Rule in Himalaya and Nepal [28, 81]. Species in tropical regions show narrow elevational ranges at the lower end of the gradient that can be predicted in part by Rapoport’s Rule, possibly because of their microhabitat requirements [14]. Our results were not entirely uniform across all methods, perhaps because their different levels of sensitivities reduced the original information to basic data for analyses [64]. Overall, the Pagel method presented a more powerful explanation than the other three with regard to elevational patterns of orchid species richness.

## Conclusions

We found that the orchid species richness in Yunnan varies significantly along elevational and environmental gradients, presenting a humped pattern with a peak at the lower end of the elevational gradient. This elevational pattern of richness in Yunnan is shaped by several mechanisms related to geometric constraints, land area, and growing environments, but climate can better explain the elevational patterns of species richness. Our findings help improve our knowledge about the potential effects of global change on orchids and will be a useful tool when developing conservation strategies for these desirable plants.

## Supporting Information

S1 File All relevant data within the present paper. Variations in mean annual temperature, mean annual precipitation, and aridity index with elevation in Yunnan, China (Figure A). Sampling density for different elevational intervals (Figure B). Land areas for different elevational intervals (Figure C). Elevation-related patterns of richness for all orchid species, terrestrial, epiphyte, and saprophyte forms in Yunnan, China (Figure D). Elevational gradients for orchid species richness in the Gaoligong Mountains, Yunnan (Figure E). Important genera within the Orchidaceae family in Yunnan Province, China (Table A). Estimated parameters of linear regression models used to explore the relationships among species richness and density, elevation, and other environmental variables (Table B). Site of collection, elevation, habitat, life form, and environmental variables associated with each studied specimen (Table C).
(PDF)
