# Supplementary material for: Orchid Species Richness along Elevational and Environmental Gradients in Yunnan, China
Source: PLoS One. 2015 Nov 10;10(11):e0142621. doi: 10.1371/journal.pone.0142621 (PMC4640717; doi:10.1371/journal.pone.0142621)
Supplement: S1 File — Variations in mean annual temperature, mean annual precipitation, and aridity index with elevation in Yunnan, China (Figure A). Sampling density for different elevational intervals (Figure B). Land areas for different elevational intervals (Figure C). Elevation-related patterns of richness for all orchid species, terrestrial, epiphyte, and saprophyte forms in Yunnan, China (Figure D). Elevational gradients for orchid species richness in the Gaoligong Mountains, Yunnan (Figure E). Important genera within the Orchidaceae family in Yunnan Province, China (Table A). Estimated parameters of linear regression models used to explore the relationships among species richness and density, elevation, and other environmental variables (Table B). Site of collection, elevation, habitat, life form, and environmental variables associated with each studied specimen (Table C). (PDF) [file pone.0142621.s001.pdf]

Supporting Information

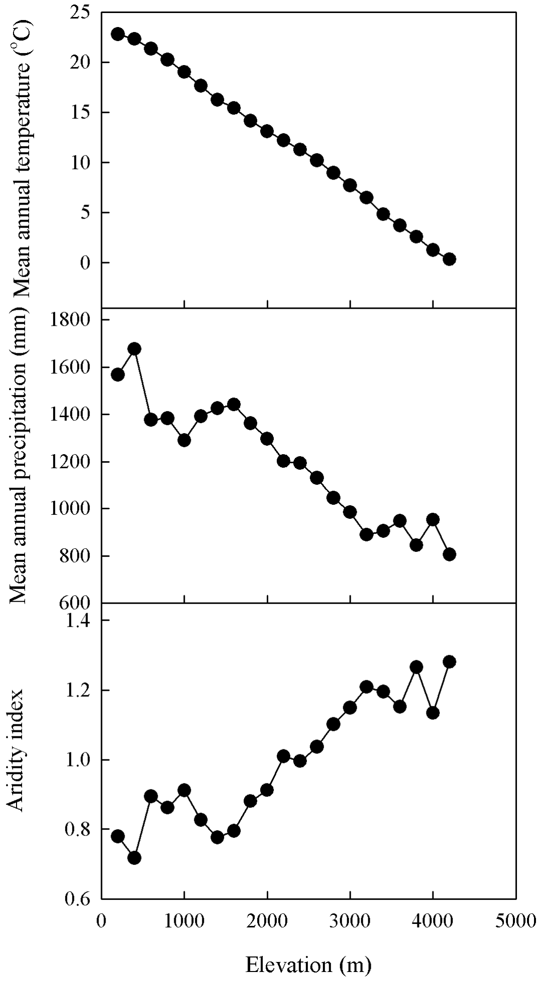

Figure A. Variations in mean annual temperature, mean annual precipitation, and aridity index with elevation in Yunnan Province, China.

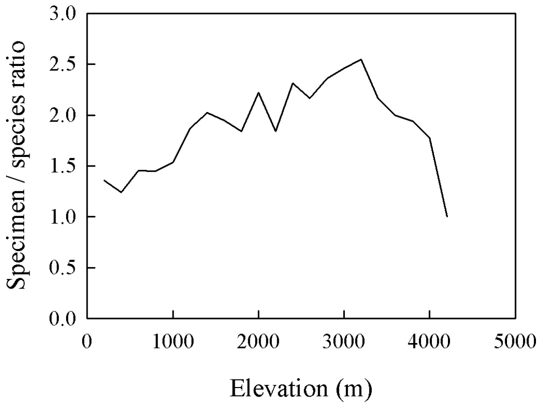

Figure B. Sampling density for different elevational intervals.

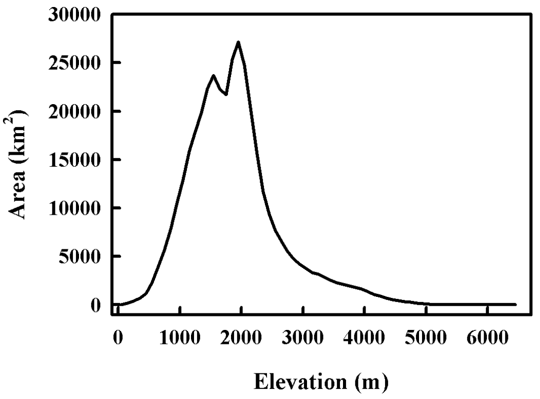

Figure C. Land areas for different elevational intervals.

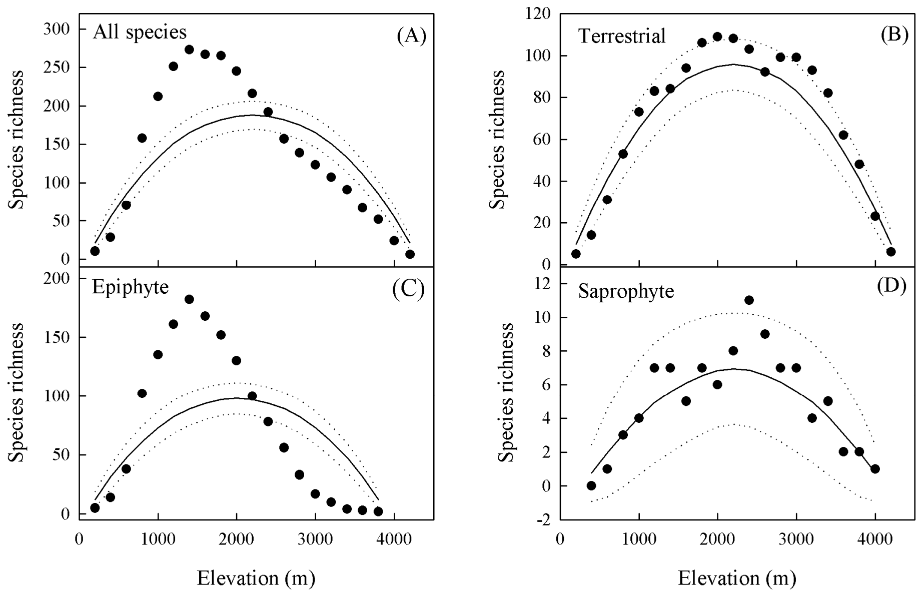

Figure D. Elevation-related patterns of richness for all orchid species (A), and terrestrial (B), epiphyte (C), and saprophyte (D) forms in Yunnan, China. Solid dots and solid lines represent observed and predicted species richness, respectively. Dashed lines show 95% confidence intervals predicted by MDE randomization model.

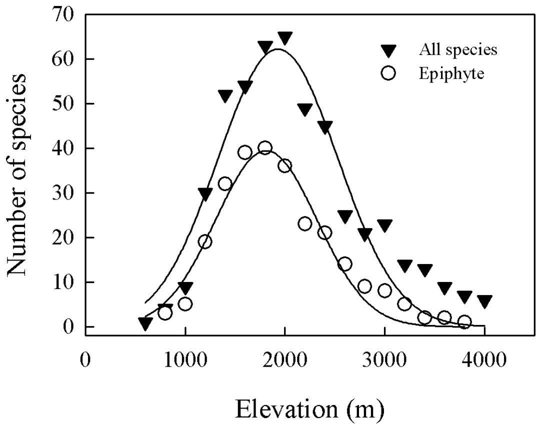

Figure E. Elevational gradients for orchid species richness in the Gaoligong Mountains, Yunnan.

Table A. Important genera within the Orchidaceae family in Yunnan Province, China.

Genus	Number of species	Genus	Number of species
Dendrobium	56	*Oberonia*	16
Bulbophyllum	42	*Peristylus*	16
Habenaria	31	*Goodyera*	14
Liparis	30	*Anoectochilus*	12
Calanthe	27	*Cleisostoma*	10
Eria	25	*Malaxis*	10
Cymbidium	23	*Orchis*	10
Platanthera	21	*Paphiopedilum*	10
Coelogyne	18	*Pholidota*	10
Cypripedium	16	*Pleione*	10
Herminium	16		

Table B. Estimated parameters of linear regression models used to explore the relationships among species richness and density, elevation, and other environmental variables. Each midpoint indicates where the value of response was maximum. Aridity index was calculated as potential evapotranspiration rate divided by mean annual precipitation.

Independent variable	Response variable	Confidence interval (95%) for independent variable (midpoint value)	Related figure
Elevation (m)	Specimen richness	1535−1865 (1700)	3A
	Genus richness	1395−1723 (1559)	3A
	Species richness	1418−1762 (1590)	3A
	Species richness for epiphytes	1367−1563 (1465)	3B
	Species richness for terrestrials	2052−2598 (2325)	3B
	Species richness for saprophytes	2307−2663 (2485)	3B
	Specimen density	1528−2028 (1778)	3C
	Genus density	1411−1769 (1590)	3C
	Species density	1414−1798 (1606)	3C
	Species density for epiphytes	1344−1556 (1450)	3D
	Species density for terrestrials	2059−2527 (2293)	3D
	Species density for saprophytes	2341−2777 (2559)	3D
Mean annual temperature (°C)	Specimen richness	12.8−17.0 (14.9)	6A
	Genus richness	13.1−18.1 (15.6)	6A
	Species richness	13.7−17.7 (15.7)	6A
	Species richness for epiphytes	15.0−17.4 (16.2)	6B
	Species richness for terrestrials	10.1−13.3 (11.7)	6B
	Species richness for saprophytes	9.9−12.9 (11.4)	6B
	Specimen density	12.3−16.7 (14.5)	6C
	Genus density	13.1−17.9 (15.5)	6C

	Species density	13.5−18.1 (15.6)	6C
	Species density for epiphytes	15.0−17.5 (16.2)	6D
	Species density for terrestrials	9.9−12.1 (11.0)	6D
	Species density for saprophytes	9.7−12.1 (10.9)	6D
Mean annual precipitation (mm)	Specimen richness	1207−1397 (1302)	8A
	Genus richness	1216−1414 (1315)	8A
	Species richness	1237−1414 (1326)	8A
	Species richness for epiphytes	1309−1411 (1360)	8B
	Species richness for terrestrials	1099−1259 (1179)	8B
	Species richness for saprophytes	1094−1244 (1169)	8B
	Specimen density	1179−1377 (1278)	8C
	Genus density	1191−1417 (1304)	8C
	Species density	1232−1426 (1329)	8C
	Species density for epiphytes	1307−1405 (1356)	8D
	Species density for terrestrials	1078−1234 (1156)	8D
	Species density for saprophytes	1065−1225 (1145)	8D
Aridity index	Specimen richness	0.87−0.99 (0.93)	9A
	Genus richness	0.86−1.00 (0.93)	9A
	Species richness	0.86−0.98 (0.92)	9A
	Species richness for epiphytes	0.83−0.91 (0.87)	9B
	Species richness for terrestrials	0.94−1.06 (1.00)	9B
	Species richness for saprophytes	0.95−1.07 (1.01)	9B
	Specimen density	0.86−1.00 (0.94)	9C
	Genus density	0.88−0.98 (0.93)	9C
	Species density	0.86−0.98 (0.92)	9C
	Species density for epiphytes	0.83−0.89 (0.86)	9D
	Species density for terrestrials	0.95−1.09 (1.02)	9D
	Species density for saprophytes	0.97−1.09 (1.03)	9D

Table C. Site of collection, elevation, habitat, life form, and environmental variables associated with each studied specimen.

Species	Site	Elevation	Mean annual temperature	Mean annual precipitation	Relative humidity	Potential Evapotranspiration	Aridity index	Life form	Habitat	Endemism
Acampe multiflora	Pingbian	320	22.90	1648.57	86.34	990.16	0.60	Epiphyte	Sparse forest	Non-endemic
Acampe multiflora	Puer	1010	20.20	1429.78	79.51	1123.79	0.79	Epiphyte	Open site	Non-endemic
Acampe multiflora	Lijiang	2020	14.95	982.53	63.24	1077.65	1.10	Epiphyte	Sparse forest	Non-endemic
Acampe ochracea	Menghai	1100	18.96	1314.38	80.83	1150.91	0.88	Epiphyte	Dense forest	Non-endemic
Acampe rigida	Mengla	650	21.30	1514.70	84.25	1146.68	0.76	Epiphyte	Dense forest	Non-endemic
Acampe rigida	Luoping	800	19.24	1686.24	82.51	1040.03	0.62	Epiphyte	Forest edge	Non-endemic
Acampe rigida	Jinghong	900	20.32	1161.08	80.46	1256.19	1.08	Epiphyte	Shrubland	Non-endemic
Acampe rigida	Lushui	1000	20.05	1195.57	70.63	911.65	0.76	Epiphyte	Dense forest	Non-endemic
Acampe rigida	Menghai	1000	19.56	1314.38	80.83	1150.91	0.88	Epiphyte	Dense forest	Non-endemic
Acampe rigida	Jinghong	1100	19.12	1161.08	80.46	1256.19	1.08	Epiphyte	Dense forest	Non-endemic
Acampe rigida	Jingdong	1100	18.98	1128.40	76.80	1142.11	1.01	Epiphyte	Dense forest	Non-endemic
Acampe rigida	Jinghong	1100	19.12	1161.08	80.46	1256.19	1.08	Epiphyte	Forest edge	Non-endemic
Acampe rigida	Zhenyuan	1200	19.04	1254.48	77.40	1138.06	0.91	Epiphyte	Dense forest	Non-endemic
Acanthephippium striatum	Pingbian	910	19.36	1648.57	86.34	990.16	0.60	Terrestrial	Dense forest	Non-endemic
Acanthephippium sylhetense	Jinghong	540	22.48	1161.08	80.46	1256.19	1.08	Epiphyte	Dense forest	Non-endemic
Aerides falcata	Jinping	800	20.69	2305.17	83.81	1030.46	0.45	Epiphyte	Dense forest	Non-endemic
Aerides flabellata	Menghai	1080	19.08	1314.38	80.83	1150.91	0.88	Epiphyte	Forest edge	Non-endemic
Aerides rosea	Menghai	1080	19.08	1314.38	80.83	1150.91	0.88	Epiphyte	Dense forest	Non-endemic
Aerides rosea	Menghai	1100	18.96	1314.38	80.83	1150.91	0.88	Epiphyte	Dense forest	Non-endemic
Aerides rosea	Menghai	1100	18.96	1314.38	80.83	1150.91	0.88	Epiphyte	Forest edge	Non-endemic
Aerides rosea	Menghai	1350	17.46	1314.38	80.83	1150.91	0.88	Epiphyte	Sparse forest	Non-endemic
Aerides rosea	Menghai	1530	16.38	1314.38	80.83	1150.91	0.88	Epiphyte	Dense forest	Non-endemic
Agrostophyllum callosum	Pingbian	1300	17.02	1648.57	86.34	990.16	0.60	Epiphyte	Dense forest	Non-endemic
Agrostophyllum callosum	Hekou	1465	14.96	1768.58	84.25	1166.26	0.66	Epiphyte	Dense forest	Non-endemic
Agrostophyllum callosum	Gongshan	1500	15.08	1738.42	78.47	860.43	0.50	Epiphyte	Dense forest	Non-endemic
Agrostophyllum callosum	Luxi	1750	14.58	1650.33	79.33	1183.39	0.72	Epiphyte	Dense forest	Non-endemic
Agrostophyllum callosum	Jinghong	1800	14.92	1161.08	80.46	1256.19	1.08	Epiphyte	Dense forest	Non-endemic
Agrostophyllum callosum	Luchun	1900	15.30	2013.54	78.64	1151.56	0.57	Epiphyte	Dense forest	Non-endemic
Agrostophyllum callosum	Jingdong	2100	12.98	1128.40	76.80	1142.11	1.01	Epiphyte	Grassy slope	Non-endemic
Agrostophyllum callosum	Menglian	2200	12.38	1357.68	80.47	1185.94	0.87	Epiphyte	Meadow	Non-endemic
Agrostophyllum callosum	Jingdong	2400	11.18	1128.40	76.80	1142.11	1.01	Epiphyte	Dense forest	Non-endemic
Amitostigma basifoliatum	Lijiang	2600	11.47	982.53	63.24	1077.65	1.10	Terrestrial	Dense forest	Non-endemic
Amitostigma basifoliatum	Lijiang	2750	10.57	982.53	63.24	1077.65	1.10	Terrestrial	Meadow	Non-endemic

Amitostigma basifoliatum	Lijiang	2800	10.27	982.53	63.24	1077.65	1.10	Terrestrial	Grassy slope	Non-endemic
Amitostigma basifoliatum	Heqing	2800	9.99	977.00	65.16	1211.84	1.24	Terrestrial	Meadow	Non-endemic
Amitostigma basifoliatum	Lijiang	2900	9.67	982.53	63.24	1077.65	1.10	Terrestrial	Meadow	Non-endemic
Amitostigma basifoliatum	Weixi	3000	7.43	970.70	69.95	1021.03	1.05	Terrestrial	Meadow	Non-endemic
Amitostigma basifoliatum	Huize	3600	3.77	799.98	71.46	1179.17	1.47	Terrestrial	Meadow	Non-endemic
Amitostigma basifoliatum	Zhongdian	4175	0.39	641.73	69.05	914.89	1.43	Terrestrial	Meadow	Non-endemic
Amitostigma faberi	Lijiang	2600	11.47	982.53	63.24	1077.65	1.10	Terrestrial	Open site	Non-endemic
Amitostigma faberi	Lijiang	2950	9.37	982.53	63.24	1077.65	1.10	Terrestrial	Open site	Non-endemic
Amitostigma farreri	Gongshan	3600	2.48	1738.42	78.47	860.43	0.50	Terrestrial	Grassy slope	Endemic
Amitostigma hemipilioides	Zhongdian	2500	10.44	641.73	69.05	914.89	1.43	Terrestrial	Shrubland	Non-endemic
Amitostigma monanthum	Lijiang	3200	7.87	982.53	63.24	1077.65	1.10	Terrestrial	Open site	Non-endemic
Amitostigma monanthum	Gongshan	3600	2.48	1738.42	78.47	860.43	0.50	Terrestrial	Grassy slope	Non-endemic
Amitostigma monanthum	Deqin	3700	2.97	639.48	70.85	896.66	1.40	Terrestrial	Open site	Non-endemic
Amitostigma monanthum var.forrestii	Zhongdian	3700	3.24	641.73	69.05	914.89	1.43	Terrestrial	Open site	Non-endemic
Amitostigma monanthum var.forrestii	Zhongdian	4000	1.44	641.73	69.05	914.89	1.43	Terrestrial	Open site	Non-endemic
Amitostigma monanthum var.forrestii	Deqin	4100	0.57	639.48	70.85	896.66	1.40	Terrestrial	Open site	Non-endemic
Amitostigma simplex	Gongshan	2200	10.88	1738.42	78.47	860.43	0.50	Terrestrial	Grassy slope	Non-endemic
Amitostigma tetralobum	Dali	1500	17.82	1082.70	68.61	1256.27	1.16	Terrestrial	Grassy slope	Non-endemic
Amitostigma tetralobum	Dali	2300	13.02	1082.70	68.61	1256.27	1.16	Terrestrial	Open site	Non-endemic
Amitostigma tetralobum	Zhenyuan	2500	11.24	1254.48	77.40	1138.06	0.91	Terrestrial	Meadow	Non-endemic
Amitostigma tibeticum	Fugong	3800	1.26	1441.43	79.99	906.18	0.63	Terrestrial	Open site	Non-endemic
Amitostigma yuanum	Fugong	3700	1.86	1441.43	79.99	906.18	0.63	Terrestrial	Dense forest	Endemic
Amitostigma yuanum	Yongde	3100	8.53	1266.25	69.00	1283.63	1.01	Terrestrial	Grassy slope	Endemic
Androcorys pugioniformis	Zhongdian	3880	2.16	641.73	69.05	914.89	1.43	Terrestrial	Dense forest	Non-endemic
Androcorys spiralis	Zhongdian	3500	4.44	641.73	69.05	914.89	1.43	Terrestrial	Dense forest	Non-endemic
Ania angustifolia	Cangyuan	1200	18.02	1733.34	81.63	1060.82	0.61	Terrestrial	Forest edge	Non-endemic
Ania angustifolia	Jinghong	1650	15.82	1161.08	80.46	1256.19	1.08	Terrestrial	Dense forest	Non-endemic
Ania hookeriana	Gongshan	1600	14.48	1738.42	78.47	860.43	0.50	Terrestrial	Shrubland	Non-endemic
Anoectochilus burmanicus	Jinghong	1050	19.42	1161.08	80.46	1256.19	1.08	Terrestrial	Dense forest	Non-endemic
Anoectochilus burmanicus	Luchun	2150	13.80	2013.54	78.64	1151.56	0.57	Terrestrial	Dense forest	Non-endemic
Anoectochilus chapaensis	Pingbian	1380	16.54	1648.57	86.34	990.16	0.60	Terrestrial	Dense forest	Non-endemic
Anoectochilus crispus	Zhenyuan	1800	15.44	1254.48	77.40	1138.06	0.91	Terrestrial	Dense forest	Non-endemic
Anoectochilus elwesii	Maguan	800	20.21	1330.52	83.25	1086.43	0.82	Terrestrial	Dense forest	Non-endemic

Anoectochilus elwesii	Xichou	1200	17.73	1267.54	82.98	985.97	0.78	Terrestrial	Dense forest	Non-endemic
Anoectochilus elwesii	Menghai	1480	16.68	1314.38	80.83	1150.91	0.88	Terrestrial	Dense forest	Non-endemic
Anoectochilus elwesii	Gongshan	1500	15.08	1738.42	78.47	860.43	0.50	Terrestrial	Dense forest	Non-endemic
Anoectochilus gengmanensis	Fengqing	2500	11.45	1352.80	73.24	1172.52	0.87	Terrestrial	Dense forest	Endemic
Anoectochilus gengmanensis	Gengma	2500	19.04	1327.70	77.28	1181.78	0.89	Terrestrial	Dense forest	Endemic
Anoectochilus grandiflorus	Mengla	590	21.66	1514.70	84.25	1146.68	0.76	Terrestrial	Dense forest	Non-endemic
Anoectochilus grandiflorus	Pingbian	1260	17.26	1648.57	86.34	990.16	0.60	Terrestrial	Dense forest	Non-endemic
Anoectochilus lanceolatus	Gongshan	1800	13.28	1738.42	78.47	860.43	0.50	Terrestrial	Dense forest	Non-endemic
Anoectochilus minor	Gongshan	2800	7.28	1738.42	78.47	860.43	0.50	Terrestrial	Dense forest	Non-endemic
Anoectochilus moulmeinensis	Gongshan	1380	15.80	1738.42	78.47	860.43	0.50	Terrestrial	Dense forest	Non-endemic
Anoectochilus moulmeinensis	Malipo	1500	15.26	1063.54	85.83	1053.97	0.99	Terrestrial	Dense forest	Non-endemic
Anoectochilus moulmeinensis	Zhenyuan	1800	15.44	1254.48	77.40	1138.06	0.91	Terrestrial	Dense forest	Non-endemic
Anoectochilus roxburghii	Mengla	650	21.30	1514.70	84.25	1146.68	0.76	Terrestrial	Grassy slope	Non-endemic
Anoectochilus roxburghii	Malipo	1000	18.26	1063.54	85.83	1053.97	0.99	Terrestrial	Dense forest	Non-endemic
Anoectochilus roxburghii	Yanshan	1100	18.86	1003.57	79.42	1172.21	1.17	Terrestrial	Dense forest	Non-endemic
Anoectochilus roxburghii	Pingbian	1500	15.82	1648.57	86.34	990.16	0.60	Terrestrial	Dense forest	Non-endemic
Anoectochilus roxburghii	Menghai	1600	15.96	1314.38	80.83	1150.91	0.88	Terrestrial	Dense forest	Non-endemic
Anoectochilus roxburghii	Jinghong	1800	14.92	1161.08	80.46	1256.19	1.08	Terrestrial	Dense forest	Non-endemic
Anoectochilus roxburghii	Lianghe	2130	11.69	1388.65	79.72	1192.10	0.86	Terrestrial	Dense forest	Non-endemic
Anoectochilus tortus	Pingbian	1000	18.82	1648.57	86.34	990.16	0.60	Terrestrial	Dense forest	Non-endemic
Anoectochilus yunnanensis	Xichou	1550	15.63	1267.54	82.98	985.97	0.78	Terrestrial	Dense forest	Endemic
Anoectochilus yunnanensis	Wenshan	1820	14.74	988.87	76.70	1272.98	1.29	Terrestrial	Dense forest	Endemic
Anoectochilus yunnanensis	Gongshan	1850	12.98	1738.42	78.47	860.43	0.50	Terrestrial	Dense forest	Endemic
Anoectochilus yunnanensis	Gongshan	2000	12.08	1738.42	78.47	860.43	0.50	Terrestrial	Dense forest	Endemic
Anota densiflora	Malipo	1000	18.26	1063.54	85.83	1053.97	0.99	Terrestrial	Sparse forest	Non-endemic
Anthogonium gracile	Menghai	858	20.41	1314.38	80.83	1150.91	0.88	Terrestrial	Open site	Non-endemic
Anthogonium gracile	Zhenyuan	1000	20.24	1254.48	77.40	1138.06	0.91	Terrestrial	Forest edge	Non-endemic
Anthogonium gracile	Wenshan	1250	18.16	988.87	76.70	1272.98	1.29	Terrestrial	Grassy slope	Non-endemic
Anthogonium gracile	Puer	1300	18.46	1429.78	79.51	1123.79	0.79	Terrestrial	Dense forest	Non-endemic
Anthogonium gracile	Luchun	1300	18.90	2013.54	78.64	1151.56	0.57	Terrestrial	Open site	Non-endemic
Anthogonium gracile	Tengchong	1320	17.08	1501.45	78.06	857.93	0.57	Terrestrial	Sparse forest	Non-endemic
Anthogonium gracile	Pingbian	1380	16.54	1648.57	86.34	990.16	0.60	Terrestrial	Dense forest	Non-endemic
Anthogonium gracile	Gongshan	1450	15.38	1738.42	78.47	860.43	0.50	Terrestrial	Open site	Non-endemic
Anthogonium gracile	Shiping	1466	17.77	919.48	75.04	1244.05	1.35	Terrestrial	Dense forest	Non-endemic
Anthogonium gracile	Guangnan	1500	15.25	1044.26	78.94	1090.35	1.04	Terrestrial	Shrubland	Non-endemic
Anthogonium gracile	Zhenyuan	1500	17.24	1254.48	77.40	1138.06	0.91	Terrestrial	Shrubland	Non-endemic

Anthogonium gracile	Jingdong	1600	15.98	1128.40	76.80	1142.11	1.01	Terrestrial	Dense forest	Non-endemic
Anthogonium gracile	Jiangcheng	1600	15.53	2264.27	84.63	1061.90	0.47	Terrestrial	Dense forest	Non-endemic
Anthogonium gracile	Zhenyuan	1600	16.64	1254.48	77.40	1138.06	0.91	Terrestrial	Grassy slope	Non-endemic
Anthogonium gracile	Zhenyuan	1600	16.64	1254.48	77.40	1138.06	0.91	Terrestrial	Open site	Non-endemic
Anthogonium gracile	Fengqing	1600	16.60	1352.80	73.24	1172.52	0.87	Terrestrial	Shrubland	Non-endemic
Anthogonium gracile	Menghai	1600	15.96	1314.38	80.83	1150.91	0.88	Terrestrial	Sparse forest	Non-endemic
Anthogonium gracile	Jingdong	1650	15.68	1128.40	76.80	1142.11	1.01	Terrestrial	Dense forest	Non-endemic
Anthogonium gracile	Zhenyuan	1650	16.34	1254.48	77.40	1138.06	0.91	Terrestrial	Open site	Non-endemic
Anthogonium gracile	Jingdong	1660	15.62	1128.40	76.80	1142.11	1.01	Terrestrial	Dense forest	Non-endemic
Anthogonium gracile	Jiangchuan	1700	15.80	872.74	74.72	1181.99	1.35	Terrestrial	Dense forest	Non-endemic
Anthogonium gracile	Luchun	1700	16.50	2013.54	78.64	1151.56	0.57	Terrestrial	Shrubland	Non-endemic
Anthogonium gracile	Wenshan	1800	14.86	988.87	76.70	1272.98	1.29	Terrestrial	Shrubland	Non-endemic
Anthogonium gracile	Zhenyuan	1850	15.14	1254.48	77.40	1138.06	0.91	Terrestrial	Grassy slope	Non-endemic
Anthogonium gracile	Eshan	1890	13.87	938.39	78.62	1123.48	1.20	Terrestrial	Shrubland	Non-endemic
Anthogonium gracile	Gongshan	1900	12.68	1738.42	78.47	860.43	0.50	Terrestrial	Forest edge	Non-endemic
Anthogonium gracile	Wenshan	1900	14.26	988.87	76.70	1272.98	1.29	Terrestrial	Grassy slope	Non-endemic
Anthogonium gracile	Jiangchuan	1950	14.30	872.74	74.72	1181.99	1.35	Terrestrial	Dense forest	Non-endemic
Anthogonium gracile	Kunming	1950	14.58	1019.14	72.30	1197.62	1.18	Terrestrial	Dense forest	Non-endemic
Anthogonium gracile	Maguan	1950	13.31	1330.52	83.25	1086.43	0.82	Terrestrial	Dense forest	Non-endemic
Anthogonium gracile	Yuliang	1962	13.75	905.45	75.03	1218.48	1.35	Terrestrial	Dense forest	Non-endemic
Anthogonium gracile	Gongshan	2000	12.08	1738.42	78.47	860.43	0.50	Terrestrial	Grassy slope	Non-endemic
Anthogonium gracile	Hekou	2000	11.75	1768.58	84.25	1166.26	0.66	Terrestrial	Open site	Non-endemic
Anthogonium gracile	Zhenyuan	2000	14.24	1254.48	77.40	1138.06	0.91	Terrestrial	Shrubland	Non-endemic
Anthogonium gracile	Jinping	2250	11.99	2305.17	83.81	1030.46	0.45	Terrestrial	Dense forest	Non-endemic
Anthogonium gracile	Gongshan	2300	10.28	1738.42	78.47	860.43	0.50	Terrestrial	Dense forest	Non-endemic
Anthogonium gracile	Kunming	2300	12.48	1019.14	72.30	1197.62	1.18	Terrestrial	Grassy slope	Non-endemic
Anthogonium gracile	Yongde	2300	13.33	1266.25	69.00	1283.63	1.01	Terrestrial	Grassy slope	Non-endemic
Aphyllorchis caudata	Zhenkang	2650	8.94	1602.96	81.14	1089.09	0.68	Saprophyte	Dense forest	Non-endemic
Aphyllorchis gollanii	Yongde	2400	12.73	1266.25	69.00	1283.63	1.01	Terrestrial	Dense forest	Non-endemic
Aphyllorchis montana	Jinghong	1500	16.72	1161.08	80.46	1256.19	1.08	Saprophyte	Dense forest	Non-endemic
Appendicula cornuta	Mengla	830	20.22	1514.70	84.25	1146.68	0.76	Epiphyte	Dense forest	Non-endemic
Arachnis labrosa	Menghai	1080	19.08	1314.38	80.83	1150.91	0.88	Epiphyte	Dense forest	Non-endemic
Arundina graminifolia	Hekou	120	23.03	1768.58	84.25	1166.26	0.66	Terrestrial	Open site	Non-endemic
Arundina graminifolia	Hekou	170	22.73	1768.58	84.25	1166.26	0.66	Terrestrial	Sparse forest	Non-endemic
Arundina graminifolia	Hekou	280	22.07	1768.58	84.25	1166.26	0.66	Terrestrial	Dense forest	Non-endemic
Arundina graminifolia	Hekou	365	21.56	1768.58	84.25	1166.26	0.66	Terrestrial	Dense forest	Non-endemic

Arundina graminifolia	Malipo	500	21.26	1063.54	85.83	1053.97	0.99	Terrestrial	Sparse forest	Non-endemic
Arundina graminifolia	Luchun	530	23.52	2013.54	78.64	1151.56	0.57	Terrestrial	Open site	Non-endemic
Arundina graminifolia	Luchun	530	23.52	2013.54	78.64	1151.56	0.57	Terrestrial	Shrubland	Non-endemic
Arundina graminifolia	Jinping	550	22.19	2305.17	83.81	1030.46	0.45	Terrestrial	Dense forest	Non-endemic
Arundina graminifolia	Luchun	600	23.10	2013.54	78.64	1151.56	0.57	Terrestrial	Open site	Non-endemic
Arundina graminifolia	Hekou	900	18.35	1768.58	84.25	1166.26	0.66	Terrestrial	Open site	Non-endemic
Arundina graminifolia	Mengzi	950	20.76	855.35	71.82	1434.56	1.68	Terrestrial	Open site	Non-endemic
Arundina graminifolia	Luxi	1000	19.08	1650.33	79.33	1183.39	0.72	Terrestrial	Grassy slope	Non-endemic
Arundina graminifolia	Mengla	1040	18.96	1514.70	84.25	1146.68	0.76	Terrestrial	Grassy slope	Non-endemic
Arundina graminifolia	Zhenkang	1180	17.76	1602.96	81.14	1089.09	0.68	Terrestrial	Dense forest	Non-endemic
Arundina graminifolia	Fugong	1200	16.86	1441.43	79.99	906.18	0.63	Terrestrial	Dense forest	Non-endemic
Arundina graminifolia	Lancang	1200	18.50	1596.50	77.88	1183.80	0.74	Terrestrial	Dense forest	Non-endemic
Arundina graminifolia	Jiangcheng	1200	17.93	2264.27	84.63	1061.90	0.47	Terrestrial	Grassy slope	Non-endemic
Arundina graminifolia	Jinghong	1200	18.52	1161.08	80.46	1256.19	1.08	Terrestrial	Open site	Non-endemic
Arundina graminifolia	Hekou	1250	16.25	1768.58	84.25	1166.26	0.66	Terrestrial	Open site	Non-endemic
Arundina graminifolia	Lincang	1300	18.72	1165.84	72.08	1167.73	1.00	Terrestrial	Dense forest	Non-endemic
Arundina graminifolia	Funing	1300	15.82	1161.58	79.07	1147.17	0.99	Terrestrial	Forest edge	Non-endemic
Arundina graminifolia	Gongshan	1300	16.28	1738.42	78.47	860.43	0.50	Terrestrial	Shrubland	Non-endemic
Arundina graminifolia	Tengchong	1320	17.08	1501.45	78.06	857.93	0.57	Terrestrial	Dense forest	Non-endemic
Arundina graminifolia	Funing	1400	15.22	1161.58	79.07	1147.17	0.99	Terrestrial	Dense forest	Non-endemic
Arundina graminifolia	Maguan	1500	16.01	1330.52	83.25	1086.43	0.82	Terrestrial	Dense forest	Non-endemic
Arundina graminifolia	Xichou	1500	15.93	1267.54	82.98	985.97	0.78	Terrestrial	Open site	Non-endemic
Arundina graminifolia	Shuangjiang	1500	16.98	1006.64	75.26	1272.13	1.26	Terrestrial	Sparse forest	Non-endemic
Arundina graminifolia	Yuanjiang	1600	16.58	805.59	69.08	1652.32	2.05	Terrestrial	Grassy slope	Non-endemic
Arundina graminifolia	Tengchong	1600	15.40	1501.45	78.06	857.93	0.57	Terrestrial	Shrubland	Non-endemic
Arundina graminifolia	Menglian	1650	15.68	1357.68	80.47	1185.94	0.87	Terrestrial	Meadow	Non-endemic
Arundina graminifolia	Luchun	1700	16.50	2013.54	78.64	1151.56	0.57	Terrestrial	Shrubland	Non-endemic
Arundina graminifolia	Fugong	1850	12.96	1441.43	79.99	906.18	0.63	Terrestrial	Open site	Non-endemic
Arundina graminifolia	Lianghe	2068	12.06	1388.65	79.72	1192.10	0.86	Terrestrial	Open site	Non-endemic
Arundina graminifolia	Fugong	2100	11.46	1441.43	79.99	906.18	0.63	Terrestrial	Open site	Non-endemic
Arundina graminifolia	Maguan	2400	10.61	1330.52	83.25	1086.43	0.82	Terrestrial	Grassy slope	Non-endemic
Arundina graminifolia	Eryuan	2580	11.05	745.16	68.27	1210.94	1.63	Terrestrial	Dense forest	Non-endemic
Ascocentrum ampullaceum	Jinghong	1150	18.82	1161.08	80.46	1256.19	1.08	Epiphyte	Dense forest	Non-endemic
Ascocentrum ampullaceum	Cangyuan	1200	18.02	1733.34	81.63	1060.82	0.61	Epiphyte	Dense forest	Non-endemic
Ascocentrum ampullaceum	Lancang	1500	16.70	1596.50	77.88	1183.80	0.74	Epiphyte	Dense forest	Non-endemic
Ascocentrum himalaicum	Longling	1510	15.06	2098.66	84.65	973.45	0.46	Epiphyte	Dense forest	Non-endemic

Ascocentrum himalaicum	Menghai	1770	14.94	1314.38	80.83	1150.91	0.88	Epiphyte	Sparse forest	Non-endemic
Ascocentrum himalaicum	Gongshan	1900	12.68	1738.42	78.47	860.43	0.50	Epiphyte	Dense forest	Non-endemic
Bletilla formosana	Funing	900	18.22	1161.58	79.07	1147.17	0.99	Terrestrial	Grassy slope	Non-endemic
Bletilla formosana	Jinping	900	20.09	2305.17	83.81	1030.46	0.45	Terrestrial	Grassy slope	Non-endemic
Bletilla formosana	Yanshan	1200	18.26	1003.57	79.42	1172.21	1.17	Terrestrial	Sparse forest	Non-endemic
Bletilla formosana	Xichou	1350	16.83	1267.54	82.98	985.97	0.78	Terrestrial	Sparse forest	Non-endemic
Bletilla formosana	Gongshan	1400	15.68	1738.42	78.47	860.43	0.50	Terrestrial	Forest edge	Non-endemic
Bletilla formosana	Jinping	1400	17.09	2305.17	83.81	1030.46	0.45	Terrestrial	Grassy slope	Non-endemic
Bletilla formosana	Wenshan	1400	17.26	988.87	76.70	1272.98	1.29	Terrestrial	Grassy slope	Non-endemic
Bletilla formosana	Weixin	1450	11.74	1041.32	84.86	804.04	0.77	Terrestrial	Grassy slope	Non-endemic
Bletilla formosana	Dali	1500	17.82	1082.70	68.61	1256.27	1.16	Terrestrial	Grassy slope	Non-endemic
Bletilla formosana	Gongshan	1680	14.00	1738.42	78.47	860.43	0.50	Terrestrial	Grassy slope	Non-endemic
Bletilla formosana	Midu	1830	15.36	767.67	68.75	1326.41	1.73	Terrestrial	Grassy slope	Non-endemic
Bletilla formosana	Dayao	1900	15.53	810.80	65.01	1427.38	1.76	Terrestrial	Shrubland	Non-endemic
Bletilla formosana	Lushui	2000	14.05	1195.57	70.63	911.65	0.76	Terrestrial	Dense forest	Non-endemic
Bletilla formosana	Gongshan	2000	12.08	1738.42	78.47	860.43	0.50	Terrestrial	Dense forest	Non-endemic
Bletilla formosana	Lijiang	2000	15.07	982.53	63.24	1077.65	1.10	Terrestrial	Dense forest	Non-endemic
Bletilla formosana	Dayao	2000	14.93	810.80	65.01	1427.38	1.76	Terrestrial	Shrubland	Non-endemic
Bletilla formosana	Luoping	2000	12.04	1686.24	82.51	1040.03	0.62	Terrestrial	Sparse forest	Non-endemic
Bletilla formosana	Jingdong	2032	13.39	1128.40	76.80	1142.11	1.01	Terrestrial	Dense forest	Non-endemic
Bletilla formosana	Luquan	2100	13.12	965.09	73.60	1160.89	1.20	Terrestrial	Shrubland	Non-endemic
Bletilla formosana	Binchuan	2200	13.50	573.87	62.93	1418.42	2.47	Terrestrial	Forest edge	Non-endemic
Bletilla formosana	Kunming	2300	12.48	1019.14	72.30	1197.62	1.18	Terrestrial	Open site	Non-endemic
Bletilla formosana	Zhongdian	2300	11.64	641.73	69.05	914.89	1.43	Terrestrial	Shrubland	Non-endemic
Bletilla formosana	Kunming	2320	12.36	1019.14	72.30	1197.62	1.18	Terrestrial	Grassy slope	Non-endemic
Bletilla formosana	Zhongdian	2350	11.34	641.73	69.05	914.89	1.43	Terrestrial	Shrubland	Non-endemic
Bletilla formosana	Songming	2400	11.19	1010.18	74.14	1134.24	1.12	Terrestrial	Open site	Non-endemic
Bletilla formosana	Yongde	2400	12.73	1266.25	69.00	1283.63	1.01	Terrestrial	Open site	Non-endemic
Bletilla formosana	Songming	2400	11.19	1010.18	74.14	1134.24	1.12	Terrestrial	Open site	Non-endemic
Bletilla formosana	Deqin	2500	10.17	639.48	70.85	896.66	1.40	Terrestrial	Dense forest	Non-endemic
Bletilla formosana	Zhongdian	2500	10.44	641.73	69.05	914.89	1.43	Terrestrial	Grassy slope	Non-endemic
Bletilla formosana	Deqin	2500	10.17	639.48	70.85	896.66	1.40	Terrestrial	Grassy slope	Non-endemic
Bletilla formosana	Kunming	2500	11.28	1019.14	72.30	1197.62	1.18	Terrestrial	Grassy slope	Non-endemic
Bletilla formosana	Deqin	2500	10.17	639.48	70.85	896.66	1.40	Terrestrial	Grassy slope	Non-endemic
Bletilla formosana	Gongshan	2600	8.48	1738.42	78.47	860.43	0.50	Terrestrial	Dense forest	Non-endemic
Bletilla formosana	Lijiang	2600	11.47	982.53	63.24	1077.65	1.10	Terrestrial	Grassy slope	Non-endemic

Bletilla formosana	Deqin	2700	8.97	639.48	70.85	896.66	1.40	Terrestrial	Dense forest	Non-endemic
Bletilla formosana	Jianchuan	2744	9.08	753.13	70.16	1154.48	1.53	Terrestrial	Dense forest	Non-endemic
Bletilla formosana	Eryuan	3050	8.23	745.16	68.27	1210.94	1.63	Terrestrial	Dense forest	Non-endemic
Bletilla formosana	Fugong	3100	5.46	1441.43	79.99	906.18	0.63	Terrestrial	Dense forest	Non-endemic
Bletilla ochracea	Pingbian	400	22.42	1648.57	86.34	990.16	0.60	Terrestrial	Shrubland	Non-endemic
Bletilla ochracea	Suijiang	550	17.03	917.85	78.74	923.40	1.01	Terrestrial	Open site	Non-endemic
Bletilla ochracea	Xichou	804	20.11	1267.54	82.98	985.97	0.78	Terrestrial	Dense forest	Non-endemic
Bletilla ochracea	Xichou	1350	16.83	1267.54	82.98	985.97	0.78	Terrestrial	Dense forest	Non-endemic
Bletilla ochracea	Jingdong	1380	17.30	1128.40	76.80	1142.11	1.01	Terrestrial	Dense forest	Non-endemic
Bletilla ochracea	Luquan	1960	13.96	965.09	73.60	1160.89	1.20	Terrestrial	Open site	Non-endemic
Bletilla ochracea	Anning	2100	13.43	896.67	71.23	1167.91	1.30	Terrestrial	Dense forest	Non-endemic
Bletilla ochracea	Kunming	2200	13.08	1019.14	72.30	1197.62	1.18	Terrestrial	Dense forest	Non-endemic
Bletilla ochracea	Zhongdian	2350	11.34	641.73	69.05	914.89	1.43	Terrestrial	Shrubland	Non-endemic
Bletilla ochracea	Kunming	2500	11.28	1019.14	72.30	1197.62	1.18	Terrestrial	Grassy slope	Non-endemic
Bletilla ochracea	Heqing	2840	9.75	977.00	65.16	1211.84	1.24	Terrestrial	Grassy slope	Non-endemic
Bletilla ochracea	Zhongdian	3300	5.64	641.73	69.05	914.89	1.43	Terrestrial	Sparse forest	Non-endemic
Bletilla striata	Fuyuan	2250	11.37	1084.02	74.80	1096.10	1.01	Terrestrial	Grassy slope	Non-endemic
Brachycorythis galeandra	Huaping	1200	19.95	1092.50	61.93	1424.09	1.30	Terrestrial	Shrubland	Non-endemic
Brachycorythis galeandra	Yangbi	2000	13.99	1044.23	72.19	1161.15	1.11	Terrestrial	Grassy slope	Non-endemic
Brachycorythis galeandra	Yangbi	2000	13.99	1044.23	72.19	1161.15	1.11	Terrestrial	Grassy slope	Non-endemic
Brachycorythis henryi	Mengla	500	22.20	1514.70	84.25	1146.68	0.76	Terrestrial	Dense forest	Non-endemic
Brachycorythis henryi	Jinghong	550	22.42	1161.08	80.46	1256.19	1.08	Terrestrial	Open site	Non-endemic
Brachycorythis henryi	Jinghong	700	21.52	1161.08	80.46	1256.19	1.08	Terrestrial	Dense forest	Non-endemic
Brachycorythis henryi	Mengla	950	19.50	1514.70	84.25	1146.68	0.76	Terrestrial	Dense forest	Non-endemic
Brachycorythis henryi	Jinghong	960	19.96	1161.08	80.46	1256.19	1.08	Terrestrial	Dense forest	Non-endemic
Brachycorythis henryi	Jinghong	960	19.96	1161.08	80.46	1256.19	1.08	Terrestrial	Dense forest	Non-endemic
Brachycorythis henryi	Jinghong	1060	19.36	1161.08	80.46	1256.19	1.08	Terrestrial	Dense forest	Non-endemic
Brachycorythis henryi	Menglian	1650	15.68	1357.68	80.47	1185.94	0.87	Terrestrial	Open site	Non-endemic
Brachycorythis henryi	Jinghong	1750	15.22	1161.08	80.46	1256.19	1.08	Terrestrial	Dense forest	Non-endemic
Brachycorythis henryi	Jinghong	1750	15.22	1161.08	80.46	1256.19	1.08	Terrestrial	Dense forest	Non-endemic
Bulbophyllum affine	Menghai	1100	18.96	1314.38	80.83	1150.91	0.88	Epiphyte	Dense forest	Non-endemic
Bulbophyllum affine	Menghai	1300	17.76	1314.38	80.83	1150.91	0.88	Epiphyte	Dense forest	Non-endemic
Bulbophyllum affine	Menghai	1530	16.38	1314.38	80.83	1150.91	0.88	Epiphyte	Dense forest	Non-endemic
Bulbophyllum affine	Menghai	1550	16.26	1314.38	80.83	1150.91	0.88	Epiphyte	Dense forest	Non-endemic
Bulbophyllum ambrosia	Xichou	1300	17.13	1267.54	82.98	985.97	0.78	Epiphyte	Dense forest	Non-endemic
Bulbophyllum amplifolium	Gongshan	1800	13.28	1738.42	78.47	860.43	0.50	Epiphyte	Forest edge	Non-endemic

Bulbophyllum amplifolium	Gongshan	2000	12.08	1738.42	78.47	860.43	0.50	Epiphyte	Dense forest	Non-endemic
Bulbophyllum amplifolium	Gongshan	2000	12.08	1738.42	78.47	860.43	0.50	Epiphyte	Dense forest	Non-endemic
Bulbophyllum andersonii	Yanshan	1200	18.26	1003.57	79.42	1172.21	1.17	Epiphyte	Dense forest	Non-endemic
Bulbophyllum andersonii	Pingbian	1400	16.42	1648.57	86.34	990.16	0.60	Epiphyte	Dense forest	Non-endemic
Bulbophyllum andersonii	Menghai	1500	16.56	1314.38	80.83	1150.91	0.88	Epiphyte	Dense forest	Non-endemic
Bulbophyllum andersonii	Xichou	1500	15.93	1267.54	82.98	985.97	0.78	Epiphyte	Dense forest	Non-endemic
Bulbophyllum andersonii	Xichou	1500	15.93	1267.54	82.98	985.97	0.78	Epiphyte	Sparse forest	Non-endemic
Bulbophyllum andersonii	Xichou	1550	15.63	1267.54	82.98	985.97	0.78	Epiphyte	Dense forest	Non-endemic
Bulbophyllum andersonii	Xichou	1550	15.63	1267.54	82.98	985.97	0.78	Epiphyte	Sparse forest	Non-endemic
Bulbophyllum andersonii	Xichou	1600	15.33	1267.54	82.98	985.97	0.78	Epiphyte	Dense forest	Non-endemic
Bulbophyllum andersonii	Malipo	1800	13.46	1063.54	85.83	1053.97	0.99	Epiphyte	Dense forest	Non-endemic
Bulbophyllum andersonii	Malipo	2000	12.26	1063.54	85.83	1053.97	0.99	Epiphyte	Sparse forest	Non-endemic
Bulbophyllum bomiense	Gongshan	2700	7.88	1738.42	78.47	860.43	0.50	Epiphyte	Dense forest	Non-endemic
Bulbophyllum corallinum	Jinghong	1270	18.10	1161.08	80.46	1256.19	1.08	Epiphyte	Dense forest	Non-endemic
Bulbophyllum corallinum	Menghai	1500	16.56	1314.38	80.83	1150.91	0.88	Epiphyte	Dense forest	Non-endemic
Bulbophyllum corallinum	Menghai	1530	16.38	1314.38	80.83	1150.91	0.88	Epiphyte	Dense forest	Non-endemic
Bulbophyllum crassipes	Menghai	1150	18.66	1314.38	80.83	1150.91	0.88	Epiphyte	Dense forest	Non-endemic
Bulbophyllum crassipes	Cangyuan	1200	18.02	1733.34	81.63	1060.82	0.61	Epiphyte	Dense forest	Non-endemic
Bulbophyllum crassipes	Menghai	1300	17.76	1314.38	80.83	1150.91	0.88	Epiphyte	Dense forest	Non-endemic
Bulbophyllum crassipes	Menghai	1950	13.86	1314.38	80.83	1150.91	0.88	Epiphyte	Sparse forest	Non-endemic
Bulbophyllum cylindraceum	Xichou	1500	15.93	1267.54	82.98	985.97	0.78	Epiphyte	Dense forest	Non-endemic
Bulbophyllum cylindraceum	Malipo	1600	14.66	1063.54	85.83	1053.97	0.99	Epiphyte	Dense forest	Non-endemic
Bulbophyllum cylindraceum	Zhenkang	1640	15.00	1602.96	81.14	1089.09	0.68	Epiphyte	Dense forest	Non-endemic
Bulbophyllum cylindraceum	Fengqing	1700	16.00	1352.80	73.24	1172.52	0.87	Epiphyte	Forest edge	Non-endemic
Bulbophyllum cylindraceum	Gongshan	1800	13.28	1738.42	78.47	860.43	0.50	Epiphyte	Dense forest	Non-endemic
Bulbophyllum cylindraceum	Gongshan	1800	13.28	1738.42	78.47	860.43	0.50	Epiphyte	Dense forest	Non-endemic
Bulbophyllum cylindraceum	Lushui	2200	12.85	1195.57	70.63	911.65	0.76	Epiphyte	Dense forest	Non-endemic
Bulbophyllum cylindraceum	Jingdong	2400	11.18	1128.40	76.80	1142.11	1.01	Epiphyte	Dense forest	Non-endemic
Bulbophyllum delitescens	Yongde	2600	11.53	1266.25	69.00	1283.63	1.01	Epiphyte	Dense forest	Non-endemic
Bulbophyllum drymoglossum	Xichou	1700	14.73	1267.54	82.98	985.97	0.78	Epiphyte	Dense forest	Non-endemic
Bulbophyllum drymoglossum	Jingdong	2400	11.18	1128.40	76.80	1142.11	1.01	Epiphyte	Shrubland	Non-endemic
Bulbophyllum elatum	Gongshan	2200	10.88	1738.42	78.47	860.43	0.50	Epiphyte	Forest edge	Non-endemic
Bulbophyllum emarginatum	Gongshan	1200	16.88	1738.42	78.47	860.43	0.50	Epiphyte	Dense forest	Non-endemic
Bulbophyllum emarginatum	Gongshan	1600	14.48	1738.42	78.47	860.43	0.50	Epiphyte	Dense forest	Non-endemic
Bulbophyllum emarginatum	Gongshan	1700	13.88	1738.42	78.47	860.43	0.50	Epiphyte	Dense forest	Non-endemic
Bulbophyllum emarginatum	Gongshan	1900	12.68	1738.42	78.47	860.43	0.50	epiphyte	Dense forest	Non-endemic

Bulbophyllum emarginatum	Gongshan	2000	12.08	1738.42	78.47	860.43	0.50	Epiphyte	Dense forest	Non-endemic
Bulbophyllum eublepharum	Gongshan	2080	11.60	1738.42	78.47	860.43	0.50	Epiphyte	Dense forest	Non-endemic
Bulbophyllum fooningense	Funing	1000	17.62	1161.58	79.07	1147.17	0.99	Epiphyte	Dense forest	Non-endemic
Bulbophyllum fordii	Jingdong	1950	13.88	1128.40	76.80	1142.11	1.01	Epiphyte	Sparse forest	Non-endemic
Bulbophyllum forrestii	Menghai	849	20.46	1314.38	80.83	1150.91	0.88	Epiphyte	Dense forest	Non-endemic
Bulbophyllum griffithii	Xichou	1000	18.93	1267.54	82.98	985.97	0.78	Epiphyte	Dense forest	Non-endemic
Bulbophyllum griffithii	Yuxi	2000	13.77	928.04	74.41	1159.15	1.25	epiphyte	Dense forest	Non-endemic
Bulbophyllum hastatum	Hekou	365	21.56	1768.58	84.25	1166.26	0.66	Epiphyte	Dense forest	Non-endemic
Bulbophyllum helenae	Menghai	1800	14.76	1314.38	80.83	1150.91	0.88	Epiphyte	Dense forest	Non-endemic
Bulbophyllum helenae	Jingdong	1950	13.88	1128.40	76.80	1142.11	1.01	Epiphyte	Open site	Non-endemic
Bulbophyllum helenae	Lushui	1980	14.17	1195.57	70.63	911.65	0.76	Epiphyte	Forest edge	Non-endemic
Bulbophyllum helenae	Fengqing	2300	12.40	1352.80	73.24	1172.52	0.87	Epiphyte	Dense forest	Non-endemic
Bulbophyllum insulsum	Xichou	830	19.95	1267.54	82.98	985.97	0.78	Epiphyte	Dense forest	Non-endemic
Bulbophyllum insulsum	Gongshan	1320	16.16	1738.42	78.47	860.43	0.50	Epiphyte	Dense forest	Non-endemic
Bulbophyllum insulsum	Xichou	1460	16.17	1267.54	82.98	985.97	0.78	Epiphyte	Dense forest	Non-endemic
Bulbophyllum insulsum	Gongshan	1560	14.72	1738.42	78.47	860.43	0.50	Epiphyte	Dense forest	Non-endemic
Bulbophyllum kwangtungense	Yanshan	1200	18.26	1003.57	79.42	1172.21	1.17	Epiphyte	Dense forest	Non-endemic
Bulbophyllum longibrachiatum	Malipo	1600	14.66	1063.54	85.83	1053.97	0.99	Epiphyte	Dense forest	Endemic
Bulbophyllum menghaiense	Menghai	1500	16.56	1314.38	80.83	1150.91	0.88	Epiphyte	Shrubland	Endemic
Bulbophyllum nigrescens	Yongde	1300	19.33	1266.25	69.00	1283.63	1.01	Epiphyte	Dense forest	Non-endemic
Bulbophyllum nigrescens	Mengla	1300	17.40	1514.70	84.25	1146.68	0.76	Epiphyte	Dense forest	Non-endemic
Bulbophyllum nigrescens	Lancang	1600	16.10	1596.50	77.88	1183.80	0.74	epiphyte	Open site	Non-endemic
Bulbophyllum nigrescens	Lancang	1800	14.90	1596.50	77.88	1183.80	0.74	Epiphyte	Dense forest	Non-endemic
Bulbophyllum odoratissimum	Mengla	650	21.30	1514.70	84.25	1146.68	0.76	Epiphyte	Dense forest	Non-endemic
Bulbophyllum odoratissimum	Maguan	800	20.21	1330.52	83.25	1086.43	0.82	Epiphyte	Forest edge	Non-endemic
Bulbophyllum odoratissimum	Xichou	827	19.97	1267.54	82.98	985.97	0.78	Epiphyte	Dense forest	Non-endemic
Bulbophyllum odoratissimum	Menglian	1000	19.58	1357.68	80.47	1185.94	0.87	Epiphyte	Dense forest	Non-endemic
Bulbophyllum odoratissimum	Menghai	1100	18.96	1314.38	80.83	1150.91	0.88	Epiphyte	Dense forest	Non-endemic
Bulbophyllum odoratissimum	Menghai	1100	18.96	1314.38	80.83	1150.91	0.88	Epiphyte	Dense forest	Non-endemic
Bulbophyllum odoratissimum	Yanshan	1200	18.26	1003.57	79.42	1172.21	1.17	Epiphyte	Dense forest	Non-endemic
Bulbophyllum odoratissimum	Jinghong	1250	18.22	1161.08	80.46	1256.19	1.08	Epiphyte	Dense forest	Non-endemic
Bulbophyllum odoratissimum	Menghai	1340	17.52	1314.38	80.83	1150.91	0.88	Epiphyte	Shrubland	Non-endemic
Bulbophyllum odoratissimum	Xichou	1350	16.83	1267.54	82.98	985.97	0.78	Epiphyte	Dense forest	Non-endemic
Bulbophyllum odoratissimum	Jinghong	1500	16.72	1161.08	80.46	1256.19	1.08	Epiphyte	Dense forest	Non-endemic
Bulbophyllum odoratissimum	Menghai	1500	16.56	1314.38	80.83	1150.91	0.88	Epiphyte	Dense forest	Non-endemic
Bulbophyllum odoratissimum	Menghai	1540	16.32	1314.38	80.83	1150.91	0.88	Epiphyte	Dense forest	Non-endemic

Bulbophyllum odoratissimum	Menghai	1540	16.32	1314.38	80.83	1150.91	0.88	Epiphyte	Shrubland	Non-endemic
Bulbophyllum odoratissimum	Gongshan	1600	14.48	1738.42	78.47	860.43	0.50	Epiphyte	Dense forest	Non-endemic
Bulbophyllum odoratissimum	Gongshan	1700	13.88	1738.42	78.47	860.43	0.50	Epiphyte	Dense forest	Non-endemic
Bulbophyllum odoratissimum	Ruili	1700	14.80	1436.05	78.54	1237.71	0.86	Epiphyte	Dense forest	Non-endemic
Bulbophyllum odoratissimum	Menghai	1760	15.00	1314.38	80.83	1150.91	0.88	Epiphyte	Dense forest	Non-endemic
Bulbophyllum odoratissimum	Lancang	1800	14.90	1596.50	77.88	1183.80	0.74	Epiphyte	Dense forest	Non-endemic
Bulbophyllum odoratissimum	Jingdong	1800	14.78	1128.40	76.80	1142.11	1.01	Epiphyte	Dense forest	Non-endemic
Bulbophyllum odoratissimum	Fugong	1800	13.26	1441.43	79.99	906.18	0.63	Epiphyte	Dense forest	Non-endemic
Bulbophyllum odoratissimum	Fugong	1800	13.26	1441.43	79.99	906.18	0.63	Epiphyte	Dense forest	Non-endemic
Bulbophyllum odoratissimum	Jingdong	2050	13.28	1128.40	76.80	1142.11	1.01	Epiphyte	Dense forest	Non-endemic
Bulbophyllum odoratissimum	Lincang	2300	12.72	1165.84	72.08	1167.73	1.00	Epiphyte	Dense forest	Non-endemic
Bulbophyllum odoratissimum	Jingdong	2400	11.18	1128.40	76.80	1142.11	1.01	Epiphyte	Dense forest	Non-endemic
Bulbophyllum otoglossum	Deqin	2600	9.57	639.48	70.85	896.66	1.40	Epiphyte	Dense forest	Non-endemic
Bulbophyllum pectenveneris	Xichou	1600	15.33	1267.54	82.98	985.97	0.78	Epiphyte	Forest edge	Non-endemic
Bulbophyllum pectinatum	Pingbian	1460	16.06	1648.57	86.34	990.16	0.60	Epiphyte	Dense forest	Non-endemic
Bulbophyllum pectinatum	Weishan	1988	14.23	812.54	70.80	1216.02	1.50	Epiphyte	Dense forest	Non-endemic
Bulbophyllum psittacoglossum	Xichou	1500	15.93	1267.54	82.98	985.97	0.78	Epiphyte	Dense forest	Non-endemic
Bulbophyllum rassipes	Jinghong	860	20.56	1161.08	80.46	1256.19	1.08	Epiphyte	Dense forest	Non-endemic
Bulbophyllum rassipes	Menghai	1300	17.76	1314.38	80.83	1150.91	0.88	Epiphyte	Dense forest	Non-endemic
Bulbophyllum rassipes	Menghai	1500	16.56	1314.38	80.83	1150.91	0.88	Epiphyte	Dense forest	Non-endemic
Bulbophyllum reptans	Mengla	900	19.80	1514.70	84.25	1146.68	0.76	Epiphyte	Dense forest	Non-endemic
Bulbophyllum reptans	Malipo	1000	18.26	1063.54	85.83	1053.97	0.99	Epiphyte	Dense forest	Non-endemic
Bulbophyllum reptans	Malipo	1000	18.26	1063.54	85.83	1053.97	0.99	Epiphyte	Dense forest	Non-endemic
Bulbophyllum reptans	Hekou	1000	17.75	1768.58	84.25	1166.26	0.66	Epiphyte	Dense forest	Non-endemic
Bulbophyllum reptans	Xichou	1000	18.93	1267.54	82.98	985.97	0.78	Epiphyte	Dense forest	Non-endemic
Bulbophyllum reptans	Gongshan	1300	16.28	1738.42	78.47	860.43	0.50	Epiphyte	Dense forest	Non-endemic
Bulbophyllum reptans	Gongshan	1300	16.28	1738.42	78.47	860.43	0.50	Epiphyte	Forest edge	Non-endemic
Bulbophyllum reptans	Gongshan	1350	15.98	1738.42	78.47	860.43	0.50	Epiphyte	Dense forest	Non-endemic
Bulbophyllum reptans	Gongshan	1350	15.98	1738.42	78.47	860.43	0.50	Epiphyte	Dense forest	Non-endemic
Bulbophyllum reptans	Jingdong	1350	17.48	1128.40	76.80	1142.11	1.01	Epiphyte	Dense forest	Non-endemic
Bulbophyllum reptans	Gongshan	1360	15.92	1738.42	78.47	860.43	0.50	Epiphyte	Dense forest	Non-endemic
Bulbophyllum reptans	Gongshan	1380	15.80	1738.42	78.47	860.43	0.50	Epiphyte	Dense forest	Non-endemic
Bulbophyllum reptans	Gongshan	1400	15.68	1738.42	78.47	860.43	0.50	Epiphyte	Dense forest	Non-endemic
Bulbophyllum reptans	Gongshan	1400	15.68	1738.42	78.47	860.43	0.50	Epiphyte	Dense forest	Non-endemic
Bulbophyllum reptans	Gongshan	1400	15.68	1738.42	78.47	860.43	0.50	Epiphyte	Dense forest	Non-endemic
Bulbophyllum reptans	Gongshan	1400	15.68	1738.42	78.47	860.43	0.50	Epiphyte	Dense forest	Non-endemic

Bulbophyllum reptans	Gongshan	1450	15.38	1738.42	78.47	860.43	0.50	Epiphyte	Dense forest	Non-endemic
Bulbophyllum reptans	Yongde	1600	17.53	1266.25	69.00	1283.63	1.01	Epiphyte	Dense forest	Non-endemic
Bulbophyllum reptans	Gongshan	1700	13.88	1738.42	78.47	860.43	0.50	Epiphyte	Dense forest	Non-endemic
Bulbophyllum reptans	Malipo	1800	13.46	1063.54	85.83	1053.97	0.99	Epiphyte	Dense forest	Non-endemic
Bulbophyllum reptans	Gongshan	1800	13.28	1738.42	78.47	860.43	0.50	Epiphyte	Dense forest	Non-endemic
Bulbophyllum reptans	Pingbian	1850	13.72	1648.57	86.34	990.16	0.60	Epiphyte	Dense forest	Non-endemic
Bulbophyllum reptans	Gongshan	1850	12.98	1738.42	78.47	860.43	0.50	Epiphyte	Dense forest	Non-endemic
Bulbophyllum reptans	Pingbian	1900	13.42	1648.57	86.34	990.16	0.60	Epiphyte	Dense forest	Non-endemic
Bulbophyllum reptans	Gongshan	2000	12.08	1738.42	78.47	860.43	0.50	Epiphyte	Dense forest	Non-endemic
Bulbophyllum reptans	Gongshan	2000	12.08	1738.42	78.47	860.43	0.50	Epiphyte	Dense forest	Non-endemic
Bulbophyllum reptans	Gongshan	2000	12.08	1738.42	78.47	860.43	0.50	Epiphyte	Dense forest	Non-endemic
Bulbophyllum reptans	Gongshan	2000	12.08	1738.42	78.47	860.43	0.50	Epiphyte	Dense forest	Non-endemic
Bulbophyllum reptans	Gongshan	2000	12.08	1738.42	78.47	860.43	0.50	Epiphyte	Dense forest	Non-endemic
Bulbophyllum reptans	Gongshan	2000	12.08	1738.42	78.47	860.43	0.50	Epiphyte	Dense forest	Non-endemic
Bulbophyllum reptans	Gongshan	2030	11.90	1738.42	78.47	860.43	0.50	Epiphyte	Dense forest	Non-endemic
Bulbophyllum reptans	Lushui	2050	13.75	1195.57	70.63	911.65	0.76	Epiphyte	Dense forest	Non-endemic
Bulbophyllum reptans	Gongshan	2100	11.48	1738.42	78.47	860.43	0.50	Epiphyte	Dense forest	Non-endemic
Bulbophyllum reptans	Gongshan	2100	11.48	1738.42	78.47	860.43	0.50	Epiphyte	Dense forest	Non-endemic
Bulbophyllum reptans	Gongshan	2200	10.88	1738.42	78.47	860.43	0.50	Epiphyte	Dense forest	Non-endemic
Bulbophyllum reptans	Jingdong	2200	12.38	1128.40	76.80	1142.11	1.01	Epiphyte	Dense forest	Non-endemic
Bulbophyllum reptans	Jingdong	2250	12.08	1128.40	76.80	1142.11	1.01	Epiphyte	Dense forest	Non-endemic
Bulbophyllum reptans	Jingdong	2400	11.18	1128.40	76.80	1142.11	1.01	Epiphyte	Shrubland	Non-endemic
Bulbophyllum reptans	Zhenkang	2450	10.14	1602.96	81.14	1089.09	0.68	Epiphyte	Dense forest	Non-endemic
Bulbophyllum reptans	Gongshan	2500	9.08	1738.42	78.47	860.43	0.50	Epiphyte	Dense forest	Non-endemic
Bulbophyllum reptans	Gongshan	2500	9.08	1738.42	78.47	860.43	0.50	Epiphyte	Dense forest	Non-endemic
Bulbophyllum reptans	Lushui	2500	11.05	1195.57	70.63	911.65	0.76	Epiphyte	Dense forest	Non-endemic
Bulbophyllum reptans	Gongshan	2650	8.18	1738.42	78.47	860.43	0.50	Epiphyte	Dense forest	Non-endemic
Bulbophyllum reptans	Fugong	2800	7.26	1441.43	79.99	906.18	0.63	Epiphyte	Dense forest	Non-endemic
Bulbophyllum reptans	Lincang	2800	9.72	1165.84	72.08	1167.73	1.00	Epiphyte	Dense forest	Non-endemic
Bulbophyllum retusiusculum	Xichou	1600	15.33	1267.54	82.98	985.97	0.78	Epiphyte	Dense forest	Non-endemic
Bulbophyllum retusiusculum	Xichou	1600	15.33	1267.54	82.98	985.97	0.78	Epiphyte	Dense forest	Non-endemic
Bulbophyllum retusiusculum	Pingbian	1850	13.72	1648.57	86.34	990.16	0.60	Epiphyte	Dense forest	Non-endemic
Bulbophyllum retusiusculum	Pingbian	1850	13.72	1648.57	86.34	990.16	0.60	Epiphyte	Dense forest	Non-endemic
Bulbophyllum retusiusculum	Pingbian	1900	13.42	1648.57	86.34	990.16	0.60	Epiphyte	Dense forest	Non-endemic
Bulbophyllum retusiusculum	Gongshan	2000	12.08	1738.42	78.47	860.43	0.50	Epiphyte	Dense forest	Non-endemic
Bulbophyllum retusiusculum	Jingdong	2000	13.58	1128.40	76.80	1142.11	1.01	Epiphyte	Dense forest	Non-endemic

Bulbophyllum retusiusculum	Fugong	2000	12.06	1441.43	79.99	906.18	0.63	Epiphyte	Dense forest	Non-endemic
Bulbophyllum retusiusculum	Luchun	2100	14.10	2013.54	78.64	1151.56	0.57	Epiphyte	Dense forest	Non-endemic
Bulbophyllum retusiusculum	Jingdong	2200	12.38	1128.40	76.80	1142.11	1.01	Epiphyte	Dense forest	Non-endemic
Bulbophyllum retusiusculum	Tengchong	2300	11.20	1501.45	78.06	857.93	0.57	Epiphyte	Dense forest	Non-endemic
Bulbophyllum retusiusculum	Gongshan	2400	9.68	1738.42	78.47	860.43	0.50	Epiphyte	Dense forest	Non-endemic
Bulbophyllum retusiusculum	Jingdong	2400	11.18	1128.40	76.80	1142.11	1.01	Epiphyte	Shrubland	Non-endemic
Bulbophyllum retusiusculum	Lijiang	2420	12.55	982.53	63.24	1077.65	1.10	Epiphyte	Sparse forest	Non-endemic
Bulbophyllum retusiusculum	Yongde	2470	12.31	1266.25	69.00	1283.63	1.01	Epiphyte	Dense forest	Non-endemic
Bulbophyllum retusiusculum	Yongde	2500	12.13	1266.25	69.00	1283.63	1.01	Epiphyte	Dense forest	Non-endemic
Bulbophyllum retusiusculum	Lincang	2500	11.52	1165.84	72.08	1167.73	1.00	Epiphyte	Dense forest	Non-endemic
Bulbophyllum retusiusculum	Fugong	2500	9.06	1441.43	79.99	906.18	0.63	Epiphyte	Open site	Non-endemic
Bulbophyllum retusiusculum	Fugong	2600	8.46	1441.43	79.99	906.18	0.63	Epiphyte	Dense forest	Non-endemic
Bulbophyllum retusiusculum	Yongde	2600	11.53	1266.25	69.00	1283.63	1.01	Epiphyte	Dense forest	Non-endemic
Bulbophyllum retusiusculum	Lijiang	2640	11.23	982.53	63.24	1077.65	1.10	Epiphyte	Dense forest	Non-endemic
Bulbophyllum retusiusculum	Lijiang	2650	11.17	982.53	63.24	1077.65	1.10	Epiphyte	Dense forest	Non-endemic
Bulbophyllum retusiusculum	Lincang	2700	10.32	1165.84	72.08	1167.73	1.00	Epiphyte	Dense forest	Non-endemic
Bulbophyllum retusiusculum	Lincang	2800	9.72	1165.84	72.08	1167.73	1.00	Epiphyte	Dense forest	Non-endemic
Bulbophyllum retusiusculum	Gongshan	3648	2.19	1738.42	78.47	860.43	0.50	Epiphyte	Dense forest	Non-endemic
Bulbophyllum rothschildianum	Jinghong	1550	16.42	1161.08	80.46	1256.19	1.08	Epiphyte	Dense forest	Non-endemic
Bulbophyllum rothschildianum	Shuangjiang	1600	16.38	1006.64	75.26	1272.13	1.26	Epiphyte	Dense forest	non-endemic
Bulbophyllum rufinum	Mengla	850	20.10	1514.70	84.25	1146.68	0.76	Epiphyte	Forest edge	Non-endemic
Bulbophyllum shanicum	Lincang	1850	15.42	1165.84	72.08	1167.73	1.00	Epiphyte	Dense forest	Non-endemic
Bulbophyllum shweliense	Gongshan	1300	16.28	1738.42	78.47	860.43	0.50	Epiphyte	Dense forest	Endemic
Bulbophyllum shweliense	Gongshan	1400	15.68	1738.42	78.47	860.43	0.50	Epiphyte	Dense forest	Endemic
Bulbophyllum shweliense	Gongshan	1500	15.08	1738.42	78.47	860.43	0.50	Epiphyte	Dense forest	Endemic
Bulbophyllum shweliense	Menghai	1760	15.00	1314.38	80.83	1150.91	0.88	Epiphyte	Dense forest	Endemic
Bulbophyllum spathulatum	Jinghong	860	20.56	1161.08	80.46	1256.19	1.08	Epiphyte	Dense forest	Non-endemic
Bulbophyllum stenobulbon	Xichou	830	19.95	1267.54	82.98	985.97	0.78	Epiphyte	Dense forest	Non-endemic
Bulbophyllum sutepense	Menghai	1530	16.38	1314.38	80.83	1150.91	0.88	Epiphyte	Dense forest	Non-endemic
Bulbophyllum sutepense	Menghai	1550	16.26	1314.38	80.83	1150.91	0.88	Epiphyte	Dense forest	Non-endemic
Bulbophyllum sutepense	Jingdong	2400	11.18	1128.40	76.80	1142.11	1.01	Epiphyte	Dense forest	Non-endemic
Bulbophyllum tengchongense	Yongde	1700	16.93	1266.25	69.00	1283.63	1.01	Epiphyte	Dense forest	Endemic
Bulbophyllum umbellatum	Menghai	1530	16.38	1314.38	80.83	1150.91	0.88	Epiphyte	Dense forest	Non-endemic
Bulbophyllum umbellatum	Menghai	1550	16.26	1314.38	80.83	1150.91	0.88	Epiphyte	Dense forest	Non-endemic
Bulbophyllum umbellatum	Gongshan	1800	13.28	1738.42	78.47	860.43	0.50	Epiphyte	Dense forest	Non-endemic
Bulbophyllum umbellatum	Gongshan	2000	12.08	1738.42	78.47	860.43	0.50	Epiphyte	Sparse forest	Non-endemic

Bulbophyllum umbellatum	Fengqing	2200	13.00	1352.80	73.24	1172.52	0.87	Epiphyte	Dense forest	Non-endemic
Bulbophyllum umbellatum	Jingdong	2400	11.18	1128.40	76.80	1142.11	1.01	Epiphyte	Dense forest	Non-endemic
Bulbophyllum wallichii	Longchuan	1600	14.95	1604.50	80.46	1149.57	0.72	Epiphyte	Dense forest	Non-endemic
Bulleyia yunnanensis	Funing	700	19.42	1161.58	79.07	1147.17	0.99	Epiphyte	Open site	Endemic
Bulleyia yunnanensis	Funing	750	19.12	1161.58	79.07	1147.17	0.99	Epiphyte	Sparse forest	Endemic
Bulleyia yunnanensis	Malipo	1000	18.26	1063.54	85.83	1053.97	0.99	Epiphyte	Dense forest	Endemic
Bulleyia yunnanensis	Gongshan	1240	16.64	1738.42	78.47	860.43	0.50	Epiphyte	Dense forest	Endemic
Bulleyia yunnanensis	Dali	1300	19.02	1082.70	68.61	1256.27	1.16	Epiphyte	Dense forest	Endemic
Bulleyia yunnanensis	Gongshan	1350	15.98	1738.42	78.47	860.43	0.50	Epiphyte	Dense forest	Endemic
Bulleyia yunnanensis	Jingdong	1370	17.36	1128.40	76.80	1142.11	1.01	Epiphyte	Dense forest	Endemic
Bulleyia yunnanensis	Gongshan	1380	15.80	1738.42	78.47	860.43	0.50	Epiphyte	Dense forest	Endemic
Bulleyia yunnanensis	Pingbian	1600	15.22	1648.57	86.34	990.16	0.60	Epiphyte	Dense forest	Endemic
Bulleyia yunnanensis	Gongshan	1620	14.36	1738.42	78.47	860.43	0.50	Epiphyte	Dense forest	Endemic
Bulleyia yunnanensis	Gongshan	1690	13.94	1738.42	78.47	860.43	0.50	Epiphyte	Dense forest	Endemic
Bulleyia yunnanensis	Gongshan	1850	12.98	1738.42	78.47	860.43	0.50	Epiphyte	Dense forest	Endemic
Bulleyia yunnanensis	Jingdong	1900	14.18	1128.40	76.80	1142.11	1.01	Epiphyte	Dense forest	Endemic
Bulleyia yunnanensis	Luchun	1900	15.30	2013.54	78.64	1151.56	0.57	Epiphyte	Dense forest	Endemic
Bulleyia yunnanensis	Gongshan	1900	12.68	1738.42	78.47	860.43	0.50	Epiphyte	Forest edge	Endemic
Bulleyia yunnanensis	Gongshan	1915	12.59	1738.42	78.47	860.43	0.50	Epiphyte	Dense forest	Endemic
Bulleyia yunnanensis	Jingdong	1950	13.88	1128.40	76.80	1142.11	1.01	Epiphyte	Dense forest	Endemic
Bulleyia yunnanensis	Hekou	1950	12.05	1768.58	84.25	1166.26	0.66	Epiphyte	Dense forest	Endemic
Bulleyia yunnanensis	Gongshan	2000	12.08	1738.42	78.47	860.43	0.50	Epiphyte	Dense forest	Endemic
Bulleyia yunnanensis	Gongshan	2000	12.08	1738.42	78.47	860.43	0.50	Epiphyte	Dense forest	Endemic
Bulleyia yunnanensis	Jingdong	2000	13.58	1128.40	76.80	1142.11	1.01	Epiphyte	Dense forest	Endemic
Bulleyia yunnanensis	Fuming	2000	13.97	871.62	71.93	1209.56	1.39	Epiphyte	Dense forest	Endemic
Bulleyia yunnanensis	Gongshan	2000	12.08	1738.42	78.47	860.43	0.50	Epiphyte	Forest edge	Endemic
Bulleyia yunnanensis	Weixi	2015	13.34	970.70	69.95	1021.03	1.05	Epiphyte	Dense forest	Endemic
Bulleyia yunnanensis	Gongshan	2300	10.28	1738.42	78.47	860.43	0.50	Epiphyte	Dense forest	Endemic
Bulleyia yunnanensis	Yongde	2340	13.09	1266.25	69.00	1283.63	1.01	Epiphyte	Open site	Endemic
Bulleyia yunnanensis	Yongde	2340	13.09	1266.25	69.00	1283.63	1.01	Epiphyte	Sparse forest	Endemic
Bulleyia yunnanensis	Yangbi	2490	11.05	1044.23	72.19	1161.15	1.11	Epiphyte	Dense forest	Endemic
Bulleyia yunnanensis	Weixi	2500	10.43	970.70	69.95	1021.03	1.05	Epiphyte	Dense forest	Endemic
Bulleyia yunnanensis	Lincang	2500	11.52	1165.84	72.08	1167.73	1.00	Epiphyte	Dense forest	Endemic
Bulleyia yunnanensis	Lincang	2500	11.52	1165.84	72.08	1167.73	1.00	Epiphyte	Dense forest	Endemic
Bulleyia yunnanensis	Fugong	2500	9.06	1441.43	79.99	906.18	0.63	Epiphyte	Dense forest	Endemic
Bulleyia yunnanensis	Fugong	2700	7.86	1441.43	79.99	906.18	0.63	Epiphyte	Dense forest	Endemic

Calanthe alismaefolia	Pingbian	1100	18.22	1648.57	86.34	990.16	0.60	Terrestrial	Dense forest	Non-endemic
Calanthe alismaefolia	Hekou	1280	16.07	1768.58	84.25	1166.26	0.66	Terrestrial	Dense forest	Non-endemic
Calanthe alismaefolia	Xichou	1400	16.53	1267.54	82.98	985.97	0.78	Terrestrial	Shrubland	Non-endemic
Calanthe alismaefolia	Cangyuan	1450	16.52	1733.34	81.63	1060.82	0.61	Terrestrial	Dense forest	Non-endemic
Calanthe alismaefolia	Gongshan	1600	14.48	1738.42	78.47	860.43	0.50	Terrestrial	Dense forest	Non-endemic
Calanthe alismaefolia	Gongshan	1700	13.88	1738.42	78.47	860.43	0.50	Terrestrial	Dense forest	Non-endemic
Calanthe alismaefolia	Gongshan	1700	13.88	1738.42	78.47	860.43	0.50	Terrestrial	Forest edge	Non-endemic
Calanthe alismaefolia	Gongshan	2000	12.08	1738.42	78.47	860.43	0.50	Terrestrial	Dense forest	Non-endemic
Calanthe alismaefolia	Jingdong	2100	12.98	1128.40	76.80	1142.11	1.01	Terrestrial	Dense forest	Non-endemic
Calanthe alpina	Lijiang	2450	12.37	982.53	63.24	1077.65	1.10	Terrestrial	Forest edge	Non-endemic
Calanthe alpina	Yangbi	2498	11.00	1044.23	72.19	1161.15	1.11	Terrestrial	Dense forest	Non-endemic
Calanthe alpina	Gongshan	2500	9.08	1738.42	78.47	860.43	0.50	Terrestrial	Dense forest	Non-endemic
Calanthe alpina	Gongshan	2500	9.08	1738.42	78.47	860.43	0.50	Terrestrial	Forest edge	Non-endemic
Calanthe alpina	Yangbi	2600	10.39	1044.23	72.19	1161.15	1.11	Terrestrial	Dense forest	Non-endemic
Calanthe alpina	Yangbi	2700	9.79	1044.23	72.19	1161.15	1.11	Terrestrial	Dense forest	Non-endemic
Calanthe alpina	Yongde	2820	10.21	1266.25	69.00	1283.63	1.01	Terrestrial	Forest edge	Non-endemic
Calanthe alpina	Dongchuan	2900	8.43	1021.73	71.65	1186.61	1.16	Terrestrial	Forest edge	Non-endemic
Calanthe alpina	Dayao	3100	8.33	810.80	65.01	1427.38	1.76	Terrestrial	Dense forest	Non-endemic
Calanthe alpina	Zhongdian	3100	6.84	641.73	69.05	914.89	1.43	Terrestrial	Dense forest	Non-endemic
Calanthe alpina	Zhongdian	3400	5.04	641.73	69.05	914.89	1.43	Terrestrial	Dense forest	Non-endemic
Calanthe alpina	Zhongdian	3450	4.74	641.73	69.05	914.89	1.43	Terrestrial	Dense forest	Non-endemic
Calanthe alpina	Weixi	3500	4.43	970.70	69.95	1021.03	1.05	Terrestrial	Forest edge	Non-endemic
Calanthe arcuata	Gongshan	2200	10.88	1738.42	78.47	860.43	0.50	Terrestrial	Dense forest	Non-endemic
Calanthe arcuata	Yongde	2360	12.97	1266.25	69.00	1283.63	1.01	Terrestrial	Grassy slope	Non-endemic
Calanthe arcuata	Qiaojia	2600	8.02	878.15	76.01	1034.97	1.18	Terrestrial	Grassy slope	Non-endemic
Calanthe arcuata	Gongshan	3100	5.48	1738.42	78.47	860.43	0.50	Terrestrial	Dense forest	Non-endemic
Calanthe argenteo-striata	Malipo	1000	18.26	1063.54	85.83	1053.97	0.99	Terrestrial	Dense forest	Non-endemic
Calanthe biloba	Jingdong	1800	14.78	1128.40	76.80	1142.11	1.01	Terrestrial	Shrubland	Non-endemic
Calanthe brevicornu	Gongshan	1880	12.80	1738.42	78.47	860.43	0.50	Terrestrial	Dense forest	Non-endemic
Calanthe brevicornu	Gongshan	2000	12.08	1738.42	78.47	860.43	0.50	Terrestrial	Dense forest	Non-endemic
Calanthe brevicornu	Gongshan	2000	12.08	1738.42	78.47	860.43	0.50	Terrestrial	Dense forest	Non-endemic
Calanthe brevicornu	Gongshan	2300	10.28	1738.42	78.47	860.43	0.50	Terrestrial	Dense forest	Non-endemic
Calanthe brevicornu	Gongshan	2300	10.28	1738.42	78.47	860.43	0.50	Terrestrial	Dense forest	Non-endemic
Calanthe brevicornu	Weixi	2300	11.63	970.70	69.95	1021.03	1.05	Terrestrial	Forest edge	Non-endemic
Calanthe brevicornu	Weixi	2300	11.63	970.70	69.95	1021.03	1.05	Terrestrial	Forest edge	Non-endemic
Calanthe brevicornu	Tengchong	2400	10.60	1501.45	78.06	857.93	0.57	Terrestrial	Dense forest	Non-endemic

Calanthe brevicornu	Lushui	2400	11.65	1195.57	70.63	911.65	0.76	Terrestrial	Forest edge	Non-endemic
Calanthe brevicornu	Yangbi	2498	11.00	1044.23	72.19	1161.15	1.11	Terrestrial	Dense forest	Non-endemic
Calanthe brevicornu	Weixi	2500	10.43	970.70	69.95	1021.03	1.05	Terrestrial	Dense forest	Non-endemic
Calanthe brevicornu	Yongde	2600	11.53	1266.25	69.00	1283.63	1.01	Terrestrial	Forest edge	Non-endemic
Calanthe brevicornu	Yongde	2600	11.53	1266.25	69.00	1283.63	1.01	Terrestrial	Forest edge	Non-endemic
Calanthe brevicornu	Luquan	2600	10.12	965.09	73.60	1160.89	1.20	Terrestrial	Shrubland	Non-endemic
Calanthe brevicornu	Weixi	2800	8.63	970.70	69.95	1021.03	1.05	Terrestrial	Dense forest	Non-endemic
Calanthe brevicornu	Weixi	2800	8.63	970.70	69.95	1021.03	1.05	Terrestrial	Dense forest	Non-endemic
Calanthe brevicornu	Weixi	2800	8.63	970.70	69.95	1021.03	1.05	Terrestrial	Dense forest	Non-endemic
Calanthe brevicornu	Jingdong	2800	8.78	1128.40	76.80	1142.11	1.01	Terrestrial	Dense forest	Non-endemic
Calanthe clavata	Malipo	750	19.76	1063.54	85.83	1053.97	0.99	Terrestrial	Dense forest	Non-endemic
Calanthe clavata	Malipo	1000	18.26	1063.54	85.83	1053.97	0.99	Terrestrial	Dense forest	Non-endemic
Calanthe davidii	Xichou	830	19.95	1267.54	82.98	985.97	0.78	Terrestrial	Dense forest	Non-endemic
Calanthe davidii	Xichou	850	19.83	1267.54	82.98	985.97	0.78	Terrestrial	Dense forest	Non-endemic
Calanthe davidii	Luoping	1300	16.24	1686.24	82.51	1040.03	0.62	Terrestrial	Dense forest	Non-endemic
Calanthe davidii	Gongshan	1330	16.10	1738.42	78.47	860.43	0.50	Terrestrial	Dense forest	Non-endemic
Calanthe davidii	Gongshan	1350	15.98	1738.42	78.47	860.43	0.50	Terrestrial	Dense forest	Non-endemic
Calanthe davidii	Pingbian	1400	16.42	1648.57	86.34	990.16	0.60	Terrestrial	Dense forest	Non-endemic
Calanthe davidii	Pingbian	1700	14.62	1648.57	86.34	990.16	0.60	Terrestrial	Dense forest	Non-endemic
Calanthe davidii	Gongshan	1800	13.28	1738.42	78.47	860.43	0.50	Terrestrial	Dense forest	Non-endemic
Calanthe davidii	Kunming	1900	14.88	1019.14	72.30	1197.62	1.18	Terrestrial	Dense forest	Non-endemic
Calanthe davidii	Malipo	1900	12.86	1063.54	85.83	1053.97	0.99	Terrestrial	Sparse forest	Non-endemic
Calanthe davidii	Longling	2000	12.12	2098.66	84.65	973.45	0.46	Terrestrial	Dense forest	Non-endemic
Calanthe davidii	Gongshan	2000	12.08	1738.42	78.47	860.43	0.50	Terrestrial	Dense forest	Non-endemic
Calanthe davidii	Pingbian	2200	11.62	1648.57	86.34	990.16	0.60	Terrestrial	Dense forest	Non-endemic
Calanthe davidii	Fuyuan	2200	11.67	1084.02	74.80	1096.10	1.01	Terrestrial	Shrubland	Non-endemic
Calanthe davidii	Yangbi	2280	12.31	1044.23	72.19	1161.15	1.11	Terrestrial	Dense forest	Non-endemic
Calanthe davidii	Lushui	2400	11.65	1195.57	70.63	911.65	0.76	Terrestrial	Dense forest	Non-endemic
Calanthe davidii	Weixi	2400	11.03	970.70	69.95	1021.03	1.05	Terrestrial	Forest edge	Non-endemic
Calanthe davidii	Gongshan	3000	6.08	1738.42	78.47	860.43	0.50	Terrestrial	Dense forest	Non-endemic
Calanthe delavayi	Jingdong	2600	9.98	1128.40	76.80	1142.11	1.01	Terrestrial	Dense forest	Non-endemic
Calanthe delavayi	Lijiang	2800	10.27	982.53	63.24	1077.65	1.10	Terrestrial	Dense forest	Non-endemic
Calanthe delavayi	Lijiang	2850	9.97	982.53	63.24	1077.65	1.10	Terrestrial	Forest edge	Non-endemic
Calanthe delavayi	Zhongdian	3150	6.54	641.73	69.05	914.89	1.43	Terrestrial	Meadow	Non-endemic
Calanthe delavayi	Lijiang	3300	7.27	982.53	63.24	1077.65	1.10	Terrestrial	Dense forest	Non-endemic
Calanthe delavayi	Zhongdian	3300	5.64	641.73	69.05	914.89	1.43	Terrestrial	Forest edge	Non-endemic

Calanthe delavayi	Lijiang	3300	7.27	982.53	63.24	1077.65	1.10	Terrestrial	Sparse forest	Non-endemic
Calanthe delavayi	Zhongdian	3500	4.44	641.73	69.05	914.89	1.43	Terrestrial	Dense forest	Non-endemic
Calanthe densiflora	Mengla	800	20.40	1514.70	84.25	1146.68	0.76	Terrestrial	Dense forest	Non-endemic
Calanthe densiflora	Xichou	1300	17.13	1267.54	82.98	985.97	0.78	Terrestrial	Dense forest	Non-endemic
Calanthe densiflora	Malipo	1500	15.26	1063.54	85.83	1053.97	0.99	Terrestrial	Dense forest	Non-endemic
Calanthe densiflora	Xichou	1550	15.63	1267.54	82.98	985.97	0.78	Terrestrial	Dense forest	Non-endemic
Calanthe densiflora	Pingbian	1680	14.74	1648.57	86.34	990.16	0.60	Terrestrial	Dense forest	Non-endemic
Calanthe densiflora	Gongshan	1700	13.88	1738.42	78.47	860.43	0.50	Terrestrial	Dense forest	Non-endemic
Calanthe densiflora	Gongshan	2100	11.48	1738.42	78.47	860.43	0.50	Terrestrial	Dense forest	Non-endemic
Calanthe densiflora	Fugong	2100	11.46	1441.43	79.99	906.18	0.63	Terrestrial	Dense forest	Non-endemic
Calanthe densiflora	Gongshan	2300	10.28	1738.42	78.47	860.43	0.50	Terrestrial	Shrubland	Non-endemic
Calanthe densiflora	Weixi	2500	10.43	970.70	69.95	1021.03	1.05	Terrestrial	Dense forest	Non-endemic
Calanthe densiflora	Fugong	2800	7.26	1441.43	79.99	906.18	0.63	Terrestrial	Dense forest	Non-endemic
Calanthe densiflora	Fugong	2800	7.26	1441.43	79.99	906.18	0.63	terrestrial	Dense forest	Non-endemic
Calanthe graciliflora	Longling	1700	13.92	2098.66	84.65	973.45	0.46	Terrestrial	Shrubland	Non-endemic
Calanthe graciliflora	Jingdong	2400	11.18	1128.40	76.80	1142.11	1.01	Terrestrial	Dense forest	Non-endemic
Calanthe hancockii	Funing	1000	17.62	1161.58	79.07	1147.17	0.99	Terrestrial	Dense forest	Non-endemic
Calanthe hancockii	Guangnan	1340	16.21	1044.26	78.94	1090.35	1.04	Terrestrial	Grassy slope	Non-endemic
Calanthe hancockii	Jingdong	1600	15.98	1128.40	76.80	1142.11	1.01	Terrestrial	Dense forest	Non-endemic
Calanthe hancockii	Guangnan	1600	14.65	1044.26	78.94	1090.35	1.04	Terrestrial	Dense forest	Non-endemic
Calanthe hancockii	Pingbian	1900	13.42	1648.57	86.34	990.16	0.60	Terrestrial	Dense forest	Non-endemic
Calanthe hancockii	Jingdong	1960	13.82	1128.40	76.80	1142.11	1.01	Terrestrial	Dense forest	Non-endemic
Calanthe hancockii	Weixi	2000	13.43	970.70	69.95	1021.03	1.05	Terrestrial	Dense forest	Non-endemic
Calanthe hancockii	Gongshan	2000	12.08	1738.42	78.47	860.43	0.50	Terrestrial	Forest edge	Non-endemic
Calanthe hancockii	Shuangbai	2040	14.64	942.53	72.21	1300.52	1.38	Terrestrial	Dense forest	Non-endemic
Calanthe hancockii	Qiaojia	2100	11.02	878.15	76.01	1034.97	1.18	Terrestrial	Dense forest	Non-endemic
Calanthe hancockii	Gongshan	2300	10.28	1738.42	78.47	860.43	0.50	Terrestrial	Dense forest	Non-endemic
Calanthe hancockii	Wenshan	2500	10.66	988.87	76.70	1272.98	1.29	Terrestrial	Dense forest	Non-endemic
Calanthe hancockii	Weixi	2600	9.83	970.70	69.95	1021.03	1.05	Terrestrial	Dense forest	Non-endemic
Calanthe hancockii	Weixi	2600	9.83	970.70	69.95	1021.03	1.05	Terrestrial	Dense forest	Non-endemic
Calanthe hancockii	Weixi	2600	9.83	970.70	69.95	1021.03	1.05	Terrestrial	Dense forest	Non-endemic
Calanthe hancockii	Jingdong	2600	9.98	1128.40	76.80	1142.11	1.01	Terrestrial	Dense forest	Non-endemic
Calanthe hancockii	Weixi	2700	9.23	970.70	69.95	1021.03	1.05	Terrestrial	Dense forest	Non-endemic
Calanthe hancockii	Weixi	2800	8.63	970.70	69.95	1021.03	1.05	Terrestrial	Dense forest	Non-endemic
Calanthe hancockii	Gongshan	2800	7.28	1738.42	78.47	860.43	0.50	Terrestrial	Dense forest	Non-endemic
Calanthe hancockii	Weixi	2800	8.63	970.70	69.95	1021.03	1.05	Terrestrial	Dense forest	Non-endemic

Calanthe hancockii	Weixi	3600	3.83	970.70	69.95	1021.03	1.05	Terrestrial	Grassy slope	Non-endemic
Calanthe herbacea	Pingbian	1450	16.12	1648.57	86.34	990.16	0.60	Terrestrial	Dense forest	Non-endemic
Calanthe labrosa	Mengla	760	20.64	1514.70	84.25	1146.68	0.76	Terrestrial	Dense forest	Non-endemic
Calanthe labrosa	Mengla	800	20.40	1514.70	84.25	1146.68	0.76	Terrestrial	Dense forest	Non-endemic
Calanthe mannii	Luoping	1300	16.24	1686.24	82.51	1040.03	0.62	Terrestrial	Sparse forest	Non-endemic
Calanthe metoensis	Wenshan	1850	14.56	988.87	76.70	1272.98	1.29	Terrestrial	Dense forest	Non-endemic
Calanthe odora	Pingbian	1300	17.02	1648.57	86.34	990.16	0.60	Terrestrial	Dense forest	Non-endemic
Calanthe petelotiana	Pingbian	1700	14.62	1648.57	86.34	990.16	0.60	Terrestrial	Dense forest	Non-endemic
Calanthe plantaginea	Tengchong	2200	11.80	1501.45	78.06	857.93	0.57	Terrestrial	Dense forest	Non-endemic
Calanthe puberula	Pingbian	1500	15.82	1648.57	86.34	990.16	0.60	Terrestrial	Dense forest	Non-endemic
Calanthe puberula	Fugong	2000	12.06	1441.43	79.99	906.18	0.63	Terrestrial	Dense forest	Non-endemic
Calanthe puberula	Tengchong	2070	12.58	1501.45	78.06	857.93	0.57	Terrestrial	Dense forest	Non-endemic
Calanthe puberula	Zhenkang	2450	10.14	1602.96	81.14	1089.09	0.68	Terrestrial	Dense forest	Non-endemic
Calanthe puberula	Fugong	2500	9.06	1441.43	79.99	906.18	0.63	Terrestrial	Dense forest	Non-endemic
Calanthe puberula	Lushui	2500	11.05	1195.57	70.63	911.65	0.76	Terrestrial	Dense forest	Non-endemic
Calanthe puberula	Zhenkang	3000	6.84	1602.96	81.14	1089.09	0.68	Terrestrial	Grassy slope	Non-endemic
Calanthe reflexa	Longling	1250	16.62	2098.66	84.65	973.45	0.46	Terrestrial	Shrubland	Non-endemic
Calanthe simplex	Jingdong	2450	10.88	1128.40	76.80	1142.11	1.01	Terrestrial	Dense forest	Non-endemic
Calanthe sinica	Wenshan	1600	16.06	988.87	76.70	1272.98	1.29	Terrestrial	Dense forest	Non-endemic
Calanthe sylvatica	Hekou	1050	17.45	1768.58	84.25	1166.26	0.66	Terrestrial	Dense forest	Non-endemic
Calanthe sylvatica	Yongde	1300	19.33	1266.25	69.00	1283.63	1.01	Terrestrial	Dense forest	Non-endemic
Calanthe sylvatica	Maguan	1700	14.81	1330.52	83.25	1086.43	0.82	Terrestrial	Dense forest	Non-endemic
Calanthe tricarinata	Lincang	1700	16.32	1165.84	72.08	1167.73	1.00	Terrestrial	Dense forest	Non-endemic
Calanthe tricarinata	Kunming	1900	14.88	1019.14	72.30	1197.62	1.18	Terrestrial	Dense forest	Non-endemic
Calanthe tricarinata	Yiliang	1900	10.88	751.45	71.67	1097.64	1.46	Terrestrial	Dense forest	Non-endemic
Calanthe tricarinata	Gongshan	2100	11.48	1738.42	78.47	860.43	0.50	Terrestrial	Dense forest	Non-endemic
Calanthe tricarinata	Lijiang	2100	14.47	982.53	63.24	1077.65	1.10	Terrestrial	Forest edge	Non-endemic
Calanthe tricarinata	Weixi	2200	12.23	970.70	69.95	1021.03	1.05	Terrestrial	Dense forest	Non-endemic
Calanthe tricarinata	Weixi	2300	11.63	970.70	69.95	1021.03	1.05	Terrestrial	Dense forest	Non-endemic
Calanthe tricarinata	Gongshan	2300	10.28	1738.42	78.47	860.43	0.50	Terrestrial	Dense forest	Non-endemic
Calanthe tricarinata	Lushui	2350	11.95	1195.57	70.63	911.65	0.76	Terrestrial	Dense forest	Non-endemic
Calanthe tricarinata	Zhongdian	2400	11.04	641.73	69.05	914.89	1.43	Terrestrial	Dense forest	Non-endemic
Calanthe tricarinata	Zhongdian	2400	11.04	641.73	69.05	914.89	1.43	Terrestrial	Dense forest	Non-endemic
Calanthe tricarinata	Lijiang	2400	12.67	982.53	63.24	1077.65	1.10	Terrestrial	Dense forest	Non-endemic
Calanthe tricarinata	Weixi	2400	11.03	970.70	69.95	1021.03	1.05	Terrestrial	Grassy slope	Non-endemic
Calanthe tricarinata	Gongshan	2500	9.08	1738.42	78.47	860.43	0.50	Terrestrial	Dense forest	Non-endemic

Calanthe tricarinata	Lijiang	2500	12.07	982.53	63.24	1077.65	1.10	Terrestrial	Dense forest	Non-endemic
Calanthe tricarinata	Gongshan	2500	9.08	1738.42	78.47	860.43	0.50	Terrestrial	Dense forest	Non-endemic
Calanthe tricarinata	Gongshan	2500	9.08	1738.42	78.47	860.43	0.50	Terrestrial	Dense forest	Non-endemic
Calanthe tricarinata	Qiaojia	2500	8.62	878.15	76.01	1034.97	1.18	Terrestrial	Dense forest	Non-endemic
Calanthe tricarinata	Lijiang	2600	11.47	982.53	63.24	1077.65	1.10	Terrestrial	Dense forest	Non-endemic
Calanthe tricarinata	Gongshan	2600	8.48	1738.42	78.47	860.43	0.50	Terrestrial	Dense forest	Non-endemic
Calanthe tricarinata	Fengqing	2650	10.30	1352.80	73.24	1172.52	0.87	Terrestrial	Forest edge	Non-endemic
Calanthe tricarinata	Lijiang	2700	10.87	982.53	63.24	1077.65	1.10	Terrestrial	Dense forest	Non-endemic
Calanthe tricarinata	Weixi	2700	9.23	970.70	69.95	1021.03	1.05	Terrestrial	Dense forest	Non-endemic
Calanthe tricarinata	Weixi	2900	8.03	970.70	69.95	1021.03	1.05	Terrestrial	Dense forest	Non-endemic
Calanthe tricarinata	Deqin	3000	7.17	639.48	70.85	896.66	1.40	Terrestrial	Dense forest	Non-endemic
Calanthe tricarinata	Weixi	3000	7.43	970.70	69.95	1021.03	1.05	Terrestrial	Dense forest	Non-endemic
Calanthe tricarinata	Deqin	3000	7.17	639.48	70.85	896.66	1.40	Terrestrial	Dense forest	Non-endemic
Calanthe tricarinata	Zhongdian	3100	6.84	641.73	69.05	914.89	1.43	Terrestrial	Dense forest	Non-endemic
Calanthe tricarinata	Zhongdian	3150	6.54	641.73	69.05	914.89	1.43	Terrestrial	Dense forest	Non-endemic
Calanthe tricarinata	Lijiang	3150	8.17	982.53	63.24	1077.65	1.10	Terrestrial	Forest edge	Non-endemic
Calanthe tricarinata	Weixi	3500	4.43	970.70	69.95	1021.03	1.05	Terrestrial	Dense forest	Non-endemic
Calanthe tricarinata	Weixi	3500	4.43	970.70	69.95	1021.03	1.05	Terrestrial	Shrubland	Non-endemic
Calanthe trifida	Longling	1700	13.92	2098.66	84.65	973.45	0.46	Terrestrial	Shrubland	Non-endemic
Calanthe triplicata	Malipo	550	20.96	1063.54	85.83	1053.97	0.99	Terrestrial	Dense forest	Non-endemic
Calanthe triplicata	Mengla	680	21.12	1514.70	84.25	1146.68	0.76	Terrestrial	Dense forest	Non-endemic
Calanthe triplicata	Pingbian	690	20.68	1648.57	86.34	990.16	0.60	Terrestrial	Dense forest	Non-endemic
Calanthe triplicata	Funing	800	18.82	1161.58	79.07	1147.17	0.99	Terrestrial	Dense forest	Non-endemic
Calanthe triplicata	Jinping	950	19.79	2305.17	83.81	1030.46	0.45	Terrestrial	Grassy slope	Non-endemic
Calanthe triplicata	Funing	1000	17.62	1161.58	79.07	1147.17	0.99	Terrestrial	Dense forest	Non-endemic
Calanthe triplicata	Malipo	1100	17.66	1063.54	85.83	1053.97	0.99	Terrestrial	Dense forest	Non-endemic
Calanthe triplicata	Gongshan	1300	16.28	1738.42	78.47	860.43	0.50	Terrestrial	Dense forest	Non-endemic
Calanthe triplicata	Maguan	1450	16.31	1330.52	83.25	1086.43	0.82	Terrestrial	Dense forest	Non-endemic
Calanthe triplicata	Maguan	1450	16.31	1330.52	83.25	1086.43	0.82	Terrestrial	Dense forest	Non-endemic
Calanthe triplicata	Xichou	1500	15.93	1267.54	82.98	985.97	0.78	Terrestrial	Dense forest	Non-endemic
Calanthe triplicata	Pingbian	1560	15.46	1648.57	86.34	990.16	0.60	Terrestrial	Dense forest	Non-endemic
Calanthe triplicata	Kunming	1900	14.88	1019.14	72.30	1197.62	1.18	Terrestrial	Dense forest	Non-endemic
Calanthe triplicata	Jingdong	2400	11.18	1128.40	76.80	1142.11	1.01	Terrestrial	Dense forest	Non-endemic
Cephalanthera alba	Luoping	2200	10.84	1686.24	82.51	1040.03	0.62	Terrestrial	Shrubland	Non-endemic
Cephalanthera bijiangensis	Fugong	2000	12.06	1441.43	79.99	906.18	0.63	Terrestrial	Open site	Endemic
Cephalanthera bijiangensis	Gongshan	2260	10.52	1738.42	78.47	860.43	0.50	Terrestrial	Open site	Endemic

Cephalanthera calcarata	Yangbi	2400	11.59	1044.23	72.19	1161.15	1.11	Saprophyte	Dense forest	Non-endemic
Cephalanthera calcarata	Qiaojia	2600	8.02	878.15	76.01	1034.97	1.18	Saprophyte	Dense forest	Non-endemic
Cephalanthera damasonium	Lijiang	2100	14.47	982.53	63.24	1077.65	1.10	Terrestrial	Sparse forest	Non-endemic
Cephalanthera erecta	Lijiang	2400	12.67	982.53	63.24	1077.65	1.10	Terrestrial	Dense forest	Non-endemic
Cephalanthera erecta	Fugong	2420	9.54	1441.43	79.99	906.18	0.63	Terrestrial	Dense forest	Non-endemic
Cephalanthera erecta	Fugong	2420	9.54	1441.43	79.99	906.18	0.63	Terrestrial	Dense forest	Non-endemic
Cephalanthera falcata	Gongshan	1100	17.48	1738.42	78.47	860.43	0.50	Terrestrial	Dense forest	Non-endemic
Cephalanthera falcata	Gongshan	1350	15.98	1738.42	78.47	860.43	0.50	Terrestrial	Dense forest	Non-endemic
Cephalanthera longifolia	Gongshan	1000	18.08	1738.42	78.47	860.43	0.50	Terrestrial	Forest edge	Non-endemic
Cephalanthera longifolia	Weishan	2020	14.04	812.54	70.80	1216.02	1.50	Terrestrial	Dense forest	Non-endemic
Cephalanthera longifolia	Kunming	2300	12.48	1019.14	72.30	1197.62	1.18	Terrestrial	Dense forest	Non-endemic
Cephalanthera longifolia	Weixi	2550	10.13	970.70	69.95	1021.03	1.05	Terrestrial	Dense forest	Non-endemic
Cephalanthera longifolia	Dali	2600	11.22	1082.70	68.61	1256.27	1.16	Terrestrial	Forest edge	Non-endemic
Cephalanthera longifolia	Zhongdian	2600	9.84	641.73	69.05	914.89	1.43	Terrestrial	Grassy slope	Non-endemic
Cephalanthera longifolia	Lijiang	2800	10.27	982.53	63.24	1077.65	1.10	Terrestrial	Dense forest	Non-endemic
Cephalanthera longifolia	Eryuan	2860	9.37	745.16	68.27	1210.94	1.63	Terrestrial	Grassy slope	Non-endemic
Cephalanthera longifolia	Weixi	2900	8.03	970.70	69.95	1021.03	1.05	Terrestrial	Dense forest	Non-endemic
Cephalanthera longifolia	Lijiang	2900	9.67	982.53	63.24	1077.65	1.10	Terrestrial	Shrubland	Non-endemic
Cephalanthera longifolia	Lijiang	2920	9.55	982.53	63.24	1077.65	1.10	Terrestrial	Dense forest	Non-endemic
Cephalanthera longifolia	Zhongdian	3000	7.44	641.73	69.05	914.89	1.43	Terrestrial	Dense forest	Non-endemic
Cephalanthera longifolia	Deqin	3000	7.17	639.48	70.85	896.66	1.40	Terrestrial	Sparse forest	Non-endemic
Cephalanthera longifolia	Dali	3050	8.52	1082.70	68.61	1256.27	1.16	Terrestrial	Shrubland	Non-endemic
Cephalanthera longifolia	Zhongdian	3070	7.02	641.73	69.05	914.89	1.43	Terrestrial	Open site	Non-endemic
Cephalanthera longifolia	Zhongdian	3100	6.84	641.73	69.05	914.89	1.43	Terrestrial	Dense forest	Non-endemic
Cephalanthera longifolia	Yunlong	3300	6.25	1195.57	70.63	911.65	0.76	Terrestrial	Grassy slope	Non-endemic
Cephalanthera longifolia	Deqin	3300	5.37	639.48	70.85	896.66	1.40	Terrestrial	Shrubland	Non-endemic
Cephalanthera yunnanensis	Anning	1890	14.69	896.67	71.23	1167.91	1.30	Terrestrial	Dense forest	Endemic
Cephalanthera yunnanensis	Luquan	2100	13.12	965.09	73.60	1160.89	1.20	Terrestrial	Sparse forest	Endemic
Cephalanthera yunnanensis	Luquan	2500	10.72	965.09	73.60	1160.89	1.20	Terrestrial	Dense forest	Endemic
Cephalanthera yunnanensis	Lijiang	2800	10.27	982.53	63.24	1077.65	1.10	Terrestrial	Dense forest	Endemic
Cephalantheropsis calanthoides	Gongshan	1200	16.88	1738.42	78.47	860.43	0.50	Terrestrial	Dense forest	Non-endemic
Cephalantheropsis gracilis	Mengla	800	20.40	1514.70	84.25	1146.68	0.76	Terrestrial	Dense forest	Non-endemic
Cephalantheropsis gracilis	Pingbian	1280	17.14	1648.57	86.34	990.16	0.60	Terrestrial	Dense forest	Non-endemic
Cephalantheropsis gracilis	Pingbian	1400	16.42	1648.57	86.34	990.16	0.60	Terrestrial	Dense forest	Non-endemic
Ceratostylis himalaica	Zhenkang	1290	17.10	1602.96	81.14	1089.09	0.68	Epiphyte	Dense forest	Non-endemic
Ceratostylis himalaica	Pingbian	1300	17.02	1648.57	86.34	990.16	0.60	Epiphyte	Dense forest	Non-endemic

Chamaegastrodia inverta	Tengchong	2200	11.80	1501.45	78.06	857.93	0.57	Saprophyte	Dense forest	Non-endemic
Chamaegastrodia inverta	Kunming	2600	10.68	1019.14	72.30	1197.62	1.18	Saprophyte	Dense forest	Non-endemic
Chamaegastrodia poilanei	Hekou	950	18.05	1768.58	84.25	1166.26	0.66	Saprophyte	Dense forest	Non-endemic
Cheirostylis chinensis	Guangnan	1100	17.65	1044.26	78.94	1090.35	1.04	Epiphyte	Open site	Non-endemic
Cheirostylis pingbianensis	Hekou	2000	11.75	1768.58	84.25	1166.26	0.66	Terrestrial	Dense forest	Endemic
Cheirostylis pingbianensis	Hekou	2050	11.45	1768.58	84.25	1166.26	0.66	Terrestrial	Dense forest	Endemic
Cheirostylis pingbianensis	Yongde	2360	12.97	1266.25	69.00	1283.63	1.01	Terrestrial	Open site	Endemic
Cheirostylis pingbianensis	Yongde	2400	12.73	1266.25	69.00	1283.63	1.01	Terrestrial	Forest edge	Endemic
Cheirostylis yunnanensis	Funing	600	20.02	1161.58	79.07	1147.17	0.99	Epiphyte	Open site	Non-endemic
Cheirostylis yunnanensis	Xichou	1000	18.93	1267.54	82.98	985.97	0.78	Epiphyte	Dense forest	Non-endemic
Cheirostylis yunnanensis	Xichou	1300	17.13	1267.54	82.98	985.97	0.78	Epiphyte	Forest edge	Non-endemic
Cheirostylis yunnanensis	Wenshan	1442	17.01	988.87	76.70	1272.98	1.29	Epiphyte	Open site	Non-endemic
Cheirostylis yunnanensis	Zhenkang	1640	15.00	1602.96	81.14	1089.09	0.68	Epiphyte	Dense forest	Non-endemic
Cheirostylis yunnanensis	Jingdong	1700	15.38	1128.40	76.80	1142.11	1.01	Epiphyte	Open site	Non-endemic
Cheirostylis yunnanensis	Wenshan	1800	14.86	988.87	76.70	1272.98	1.29	Epiphyte	Forest edge	Non-endemic
Chiloschista griffithii	Wuding	2260	11.83	988.68	74.74	1188.19	1.20	Epiphyte	Dense forest	Non-endemic
Chiloschista yunnanensis	Menghai	1300	17.76	1314.38	80.83	1150.91	0.88	Epiphyte	Sparse forest	Non-endemic
Chiloschista yunnanensis	Menghai	1350	17.46	1314.38	80.83	1150.91	0.88	Epiphyte	Dense forest	Non-endemic
Chiloschista yunnanensis	Lancang	1500	16.70	1596.50	77.88	1183.80	0.74	Epiphyte	Sparse forest	Non-endemic
Chiloschista yunnanensis	Menghai	2000	13.56	1314.38	80.83	1150.91	0.88	Epiphyte	Dense forest	Non-endemic
Chrysoglossum ornatum	Lincang	1500	17.52	1165.84	72.08	1167.73	1.00	Terrestrial	Dense forest	Non-endemic
Chusua pauciflora	Zhongdian	3190	6.30	641.73	69.05	914.89	1.43	Terrestrial	Dense forest	Non-endemic
Chusua pauciflora	Zhongdian	3930	1.86	641.73	69.05	914.89	1.43	Terrestrial	Dense forest	Non-endemic
Cleisostoma filiforme	Mengla	620	21.48	1514.70	84.25	1146.68	0.76	Epiphyte	Forest edge	Non-endemic
Cleisostoma fuerstenbergianum	Jinping	700	21.29	2305.17	83.81	1030.46	0.45	Epiphyte	Dense forest	Non-endemic
Cleisostoma fuerstenbergianum	Menghai	1030	19.38	1314.38	80.83	1150.91	0.88	Epiphyte	Dense forest	Non-endemic
Cleisostoma fuerstenbergianum	Menghai	1300	17.76	1314.38	80.83	1150.91	0.88	Epiphyte	Dense forest	Non-endemic
Cleisostoma fuerstenbergianum	Zhenkang	1500	15.84	1602.96	81.14	1089.09	0.68	Epiphyte	Dense forest	Non-endemic
Cleisostoma fuerstenbergianum	Menghai	1500	16.56	1314.38	80.83	1150.91	0.88	Epiphyte	Dense forest	Non-endemic
Cleisostoma fuerstenbergianum	Fengqing	1700	16.00	1352.80	73.24	1172.52	0.87	Epiphyte	Dense forest	Non-endemic
Cleisostoma fuerstenbergianum	Lancang	1800	14.90	1596.50	77.88	1183.80	0.74	Epiphyte	Dense forest	Non-endemic
Cleisostoma fuerstenbergianum	Fengqing	1900	14.80	1352.80	73.24	1172.52	0.87	Epiphyte	Dense forest	Non-endemic
Cleisostoma fuerstenbergianum	Fengqing	2000	14.20	1352.80	73.24	1172.52	0.87	Epiphyte	Dense forest	Non-endemic
Cleisostoma menghaiense	Jinghong	900	20.32	1161.08	80.46	1256.19	1.08	Epiphyte	Shrubland	Endemic
Cleisostoma menghaiense	Menghai	2000	13.56	1314.38	80.83	1150.91	0.88	Epiphyte	Dense forest	Endemic
Cleisostoma menghaiense	Yongde	2700	10.93	1266.25	69.00	1283.63	1.01	Epiphyte	Dense forest	Endemic

Cleisostoma paniculatum	Maguan	1200	17.81	1330.52	83.25	1086.43	0.82	Epiphyte	Dense forest	Non-endemic
Cleisostoma paniculatum	Pingbian	1240	17.38	1648.57	86.34	990.16	0.60	Epiphyte	Dense forest	Non-endemic
Cleisostoma paniculatum	Luoping	1300	16.24	1686.24	82.51	1040.03	0.62	Epiphyte	Dense forest	Non-endemic
Cleisostoma paniculatum	Guangnan	1300	16.45	1044.26	78.94	1090.35	1.04	Epiphyte	Shrubland	Non-endemic
Cleisostoma racemiferum	Xinping	600	22.80	957.31	74.08	1291.86	1.35	Epiphyte	Dense forest	Non-endemic
Cleisostoma racemiferum	Menghai	1770	14.94	1314.38	80.83	1150.91	0.88	Epiphyte	Dense forest	Non-endemic
Cleisostoma rostratum	Lancang	1500	16.70	1596.50	77.88	1183.80	0.74	Epiphyte	Dense forest	Non-endemic
Cleisostoma sagittiforme	Jinghong	540	22.48	1161.08	80.46	1256.19	1.08	Epiphyte	Dense forest	Non-endemic
Cleisostoma sagittiforme	Jinghong	650	21.82	1161.08	80.46	1256.19	1.08	Epiphyte	Sparse forest	Non-endemic
Cleisostoma sagittiforme	Jinghong	850	20.62	1161.08	80.46	1256.19	1.08	Epiphyte	Shrubland	Non-endemic
Cleisostoma sagittiforme	Menghai	1500	16.56	1314.38	80.83	1150.91	0.88	Epiphyte	Dense forest	Non-endemic
Cleisostoma sagittiforme	Menghai	1530	16.38	1314.38	80.83	1150.91	0.88	Epiphyte	Dense forest	Non-endemic
Cleisostoma sagittiforme	Menghai	1530	16.38	1314.38	80.83	1150.91	0.88	Epiphyte	Shrubland	Non-endemic
Cleisostoma simondii	Jinghong	1100	19.12	1161.08	80.46	1256.19	1.08	Epiphyte	Shrubland	Non-endemic
Cleisostoma simondii	Yongde	2000	15.13	1266.25	69.00	1283.63	1.01	Epiphyte	Dense forest	Non-endemic
Cleisostoma striatum	Xichou	1300	17.13	1267.54	82.98	985.97	0.78	Epiphyte	Dense forest	Non-endemic
Cleisostoma striatum	Malipo	1400	15.86	1063.54	85.83	1053.97	0.99	Epiphyte	Dense forest	Non-endemic
Cleisostoma striatum	Zhenkang	1600	15.24	1602.96	81.14	1089.09	0.68	Epiphyte	Dense forest	Non-endemic
Cleisostoma williamsonii	Funing	700	19.42	1161.58	79.07	1147.17	0.99	Epiphyte	Dense forest	Non-endemic
Cleisostoma williamsonii	Funing	700	19.42	1161.58	79.07	1147.17	0.99	Epiphyte	Forest edge	Non-endemic
Cleisostoma williamsonii	Jinping	1260	17.93	2305.17	83.81	1030.46	0.45	Epiphyte	Sparse forest	Non-endemic
Cleisostoma williamsonii	Pingbian	1400	16.42	1648.57	86.34	990.16	0.60	Epiphyte	Dense forest	Non-endemic
Cleisostoma williamsonii	Wenshan	1500	16.66	988.87	76.70	1272.98	1.29	Epiphyte	Dense forest	Non-endemic
Cleisostoma williamsonii	Yuxi	1600	16.17	928.04	74.41	1159.15	1.25	Epiphyte	Dense forest	Non-endemic
Cleisostoma williamsonii	Jinping	1700	15.29	2305.17	83.81	1030.46	0.45	Epiphyte	Dense forest	Non-endemic
Cleisostoma williamsonii	Fengqing	2200	13.00	1352.80	73.24	1172.52	0.87	Epiphyte	Dense forest	Non-endemic
Coeloglossum viride	Zhongdian	3100	6.84	641.73	69.05	914.89	1.43	Terrestrial	Dense forest	Non-endemic
Coeloglossum viride	Weixi	3100	6.83	970.70	69.95	1021.03	1.05	Terrestrial	Meadow	Non-endemic
Coeloglossum viride	Weixi	3500	4.43	970.70	69.95	1021.03	1.05	Terrestrial	Dense forest	Non-endemic
Coeloglossum viride	Zhongdian	3700	3.24	641.73	69.05	914.89	1.43	Terrestrial	Meadow	Non-endemic
Coeloglossum viride	Zhongdian	3800	2.64	641.73	69.05	914.89	1.43	Terrestrial	Dense forest	Non-endemic
Coelogyne barbata	Menghai	1080	19.08	1314.38	80.83	1150.91	0.88	Epiphyte	Shrubland	Non-endemic
Coelogyne barbata	Menghai	1080	19.08	1314.38	80.83	1150.91	0.88	Epiphyte	Shrubland	Non-endemic
Coelogyne barbata	Gongshan	1200	16.88	1738.42	78.47	860.43	0.50	Epiphyte	Dense forest	Non-endemic
Coelogyne barbata	Pingbian	1240	17.38	1648.57	86.34	990.16	0.60	Epiphyte	Dense forest	Non-endemic
Coelogyne barbata	Zhenkang	1640	15.00	1602.96	81.14	1089.09	0.68	epiphyte	Shrubland	Non-endemic

Coelogyne barbata	Gengma	1670	10.67	1327.70	77.28	1181.78	0.89	Epiphyte	Dense forest	Non-endemic
Coelogyne barbata	Eryuan	2460	11.77	745.16	68.27	1210.94	1.63	Epiphyte	Forest edge	Non-endemic
Coelogyne barbata	Fugong	2800	7.26	1441.43	79.99	906.18	0.63	Epiphyte	Dense forest	Non-endemic
Coelogyne calcicola	Zhenkang	1465	16.05	1602.96	81.14	1089.09	0.68	Epiphyte	Dense forest	Non-endemic
Coelogyne corymbosa	Yongde	2240	13.69	1266.25	69.00	1283.63	1.01	epiphyte	Forest edge	Non-endemic
Coelogyne corymbosa	Yongde	2460	12.37	1266.25	69.00	1283.63	1.01	Epiphyte	Dense forest	Non-endemic
Coelogyne corymbosa	Yongde	2480	12.25	1266.25	69.00	1283.63	1.01	Epiphyte	Forest edge	Non-endemic
Coelogyne corymbosa	Yongde	2520	12.01	1266.25	69.00	1283.63	1.01	Epiphyte	Forest edge	Non-endemic
Coelogyne corymbosa	Yongde	2700	10.93	1266.25	69.00	1283.63	1.01	Epiphyte	Dense forest	Non-endemic
Coelogyne corymbosa	Yongde	2900	9.73	1266.25	69.00	1283.63	1.01	Epiphyte	Shrubland	Non-endemic
Coelogyne fimbriata	Yanshan	1200	18.26	1003.57	79.42	1172.21	1.17	Epiphyte	Dense forest	Non-endemic
Coelogyne fimbriata	Gongshan	1380	15.80	1738.42	78.47	860.43	0.50	Epiphyte	Forest edge	Non-endemic
Coelogyne fimbriata	Weixi	1900	14.03	970.70	69.95	1021.03	1.05	Epiphyte	Forest edge	Non-endemic
Coelogyne fimbriata	Gongshan	2300	10.28	1738.42	78.47	860.43	0.50	Epiphyte	Dense forest	Non-endemic
Coelogyne flaccida	Zhenkang	1600	15.24	1602.96	81.14	1089.09	0.68	Epiphyte	Dense forest	Non-endemic
Coelogyne flaccida	Xichou	1700	14.73	1267.54	82.98	985.97	0.78	Epiphyte	Dense forest	Non-endemic
Coelogyne flaccida	Jingdong	1700	15.38	1128.40	76.80	1142.11	1.01	Epiphyte	Forest edge	Non-endemic
Coelogyne gongshanensis	Gongshan	2800	7.28	1738.42	78.47	860.43	0.50	Epiphyte	Dense forest	Endemic
Coelogyne gongshanensis	Gongshan	3000	6.08	1738.42	78.47	860.43	0.50	Epiphyte	Dense forest	Endemic
Coelogyne gongshanensis	Gongshan	3200	4.88	1738.42	78.47	860.43	0.50	Epiphyte	Shrubland	Endemic
Coelogyne leucantha	Xichou	1400	16.53	1267.54	82.98	985.97	0.78	Epiphyte	Dense forest	Non-endemic
Coelogyne leucantha	Jingdong	2070	13.16	1128.40	76.80	1142.11	1.01	Epiphyte	Dense forest	Non-endemic
Coelogyne leucantha	Nanjian	2560	12.21	735.93	63.17	1535.84	2.09	Epiphyte	Dense forest	Non-endemic
Coelogyne longipes	Xichou	850	19.83	1267.54	82.98	985.97	0.78	Epiphyte	Dense forest	Non-endemic
Coelogyne longipes	Xichou	900	19.53	1267.54	82.98	985.97	0.78	Epiphyte	Dense forest	Non-endemic
Coelogyne longipes	Xichou	1000	18.93	1267.54	82.98	985.97	0.78	Epiphyte	Dense forest	Non-endemic
Coelogyne longipes	Xichou	1100	18.33	1267.54	82.98	985.97	0.78	Epiphyte	Dense forest	Non-endemic
Coelogyne longipes	Gongshan	1380	15.80	1738.42	78.47	860.43	0.50	Epiphyte	Dense forest	Non-endemic
Coelogyne longipes	Gongshan	1400	15.68	1738.42	78.47	860.43	0.50	Epiphyte	Dense forest	Non-endemic
Coelogyne longipes	Gongshan	1400	15.68	1738.42	78.47	860.43	0.50	Epiphyte	Dense forest	Non-endemic
Coelogyne longipes	Gongshan	1400	15.68	1738.42	78.47	860.43	0.50	Epiphyte	Dense forest	Non-endemic
Coelogyne longipes	Xichou	1550	15.63	1267.54	82.98	985.97	0.78	Epiphyte	Dense forest	Non-endemic
Coelogyne longipes	Pingbian	1600	15.22	1648.57	86.34	990.16	0.60	Epiphyte	Dense forest	Non-endemic
Coelogyne longipes	Xichou	1600	15.33	1267.54	82.98	985.97	0.78	Epiphyte	Dense forest	Non-endemic
Coelogyne longipes	Pingbian	1600	15.22	1648.57	86.34	990.16	0.60	Epiphyte	Dense forest	Non-endemic
Coelogyne longipes	Gongshan	1700	13.88	1738.42	78.47	860.43	0.50	Epiphyte	Dense forest	Non-endemic

Coelogyne longipes	Xichou	1700	14.73	1267.54	82.98	985.97	0.78	Epiphyte	Dense forest	Non-endemic
Coelogyne longipes	Jinping	1750	14.99	2305.17	83.81	1030.46	0.45	Epiphyte	Dense forest	Non-endemic
Coelogyne longipes	Jinping	1800	14.69	2305.17	83.81	1030.46	0.45	Epiphyte	Dense forest	Non-endemic
Coelogyne longipes	Pingbian	1900	13.42	1648.57	86.34	990.16	0.60	Epiphyte	Dense forest	Non-endemic
Coelogyne longipes	Pingbian	1900	13.42	1648.57	86.34	990.16	0.60	Epiphyte	Dense forest	Non-endemic
Coelogyne longipes	Pingbian	1900	13.42	1648.57	86.34	990.16	0.60	Epiphyte	Dense forest	Non-endemic
Coelogyne longipes	Gongshan	2000	12.08	1738.42	78.47	860.43	0.50	Epiphyte	Dense forest	Non-endemic
Coelogyne longipes	Jingdong	2100	12.98	1128.40	76.80	1142.11	1.01	Epiphyte	Forest edge	Non-endemic
Coelogyne longipes	Jinping	2200	12.29	2305.17	83.81	1030.46	0.45	Epiphyte	Dense forest	Non-endemic
Coelogyne longipes	Fuming	2200	12.77	871.62	71.93	1209.56	1.39	Epiphyte	Forest edge	Non-endemic
Coelogyne longipes	Jingdong	2400	11.18	1128.40	76.80	1142.11	1.01	Epiphyte	Dense forest	Non-endemic
Coelogyne longipes	Lincang	2500	11.52	1165.84	72.08	1167.73	1.00	Epiphyte	Dense forest	Non-endemic
Coelogyne longipes	Jingdong	2600	9.98	1128.40	76.80	1142.11	1.01	Epiphyte	Dense forest	Non-endemic
Coelogyne nitida	Yangbi	2800	9.19	1044.23	72.19	1161.15	1.11	Epiphyte	Dense forest	Non-endemic
Coelogyne nitida	Dali	3100	8.22	1082.70	68.61	1256.27	1.16	epiphyte	Shrubland	Non-endemic
Coelogyne occultata	Gongshan	1300	16.28	1738.42	78.47	860.43	0.50	Epiphyte	Dense forest	Non-endemic
Coelogyne occultata	Gongshan	2100	11.48	1738.42	78.47	860.43	0.50	Epiphyte	Dense forest	Non-endemic
Coelogyne occultata	Lushui	2100	13.45	1195.57	70.63	911.65	0.76	Epiphyte	Dense forest	Non-endemic
Coelogyne occultata	Lushui	2300	12.25	1195.57	70.63	911.65	0.76	Epiphyte	Dense forest	Non-endemic
Coelogyne occultata	Gongshan	2300	10.28	1738.42	78.47	860.43	0.50	Epiphyte	Dense forest	Non-endemic
Coelogyne occultata	Lushui	2400	11.65	1195.57	70.63	911.65	0.76	Epiphyte	Dense forest	Non-endemic
Coelogyne occultata	Yongde	2400	12.73	1266.25	69.00	1283.63	1.01	Epiphyte	Shrubland	Non-endemic
Coelogyne occultata	Longling	2500	9.12	2098.66	84.65	973.45	0.46	Epiphyte	Dense forest	Non-endemic
Coelogyne occultata	Gongshan	2500	9.08	1738.42	78.47	860.43	0.50	Epiphyte	Dense forest	Non-endemic
Coelogyne occultata	Fugong	2700	7.86	1441.43	79.99	906.18	0.63	Epiphyte	Dense forest	Non-endemic
Coelogyne occultata	Yongde	2800	10.33	1266.25	69.00	1283.63	1.01	Epiphyte	Dense forest	Non-endemic
Coelogyne occultata	Jingdong	2800	8.78	1128.40	76.80	1142.11	1.01	Epiphyte	Sparse forest	Non-endemic
Coelogyne occultata	Fugong	3000	6.06	1441.43	79.99	906.18	0.63	Epiphyte	Dense forest	Non-endemic
Coelogyne ovalis	Malipo	1080	17.78	1063.54	85.83	1053.97	0.99	Epiphyte	Dense forest	Non-endemic
Coelogyne ovalis	Gongshan	1200	16.88	1738.42	78.47	860.43	0.50	Epiphyte	Dense forest	Non-endemic
Coelogyne ovalis	Malipo	1300	16.46	1063.54	85.83	1053.97	0.99	Epiphyte	Dense forest	Non-endemic
Coelogyne ovalis	Mengla	1300	17.40	1514.70	84.25	1146.68	0.76	Epiphyte	Dense forest	Non-endemic
Coelogyne ovalis	Jinghong	1300	17.92	1161.08	80.46	1256.19	1.08	Epiphyte	Dense forest	Non-endemic
Coelogyne ovalis	Gongshan	1350	15.98	1738.42	78.47	860.43	0.50	Epiphyte	Shrubland	Non-endemic
Coelogyne ovalis	Gongshan	1400	15.68	1738.42	78.47	860.43	0.50	Epiphyte	Dense forest	Non-endemic
Coelogyne ovalis	Jinghong	1415	17.23	1161.08	80.46	1256.19	1.08	Epiphyte	Shrubland	Non-endemic

Coelogyne ovalis	Xichou	1500	15.93	1267.54	82.98	985.97	0.78	Epiphyte	Dense forest	Non-endemic
Coelogyne ovalis	Gongshan	1600	14.48	1738.42	78.47	860.43	0.50	Epiphyte	Dense forest	Non-endemic
Coelogyne ovalis	Lushui	1600	16.45	1195.57	70.63	911.65	0.76	Epiphyte	Dense forest	Non-endemic
Coelogyne ovalis	Gongshan	1600	14.48	1738.42	78.47	860.43	0.50	Epiphyte	Dense forest	Non-endemic
Coelogyne ovalis	Gongshan	1800	13.28	1738.42	78.47	860.43	0.50	Epiphyte	Dense forest	Non-endemic
Coelogyne ovalis	Fugong	2500	9.06	1441.43	79.99	906.18	0.63	Epiphyte	Dense forest	Non-endemic
Coelogyne ovalis	Fugong	2700	7.86	1441.43	79.99	906.18	0.63	Epiphyte	Dense forest	Non-endemic
Coelogyne ovalis	Fugong	2800	7.26	1441.43	79.99	906.18	0.63	Epiphyte	Dense forest	Non-endemic
Coelogyne ovalis	Fugong	2800	7.26	1441.43	79.99	906.18	0.63	Epiphyte	Dense forest	Non-endemic
Coelogyne ovalis	Gongshan	3000	6.08	1738.42	78.47	860.43	0.50	Epiphyte	Dense forest	Non-endemic
Coelogyne ovalis	Fugong	3200	4.86	1441.43	79.99	906.18	0.63	Epiphyte	Meadow	Non-endemic
Coelogyne prolifera	Xichou	870	19.71	1267.54	82.98	985.97	0.78	Epiphyte	Dense forest	Non-endemic
Coelogyne prolifera	Xichou	1100	18.33	1267.54	82.98	985.97	0.78	Epiphyte	Dense forest	Non-endemic
Coelogyne prolifera	Gongshan	1400	15.68	1738.42	78.47	860.43	0.50	Epiphyte	Dense forest	Non-endemic
Coelogyne prolifera	Gongshan	1500	15.08	1738.42	78.47	860.43	0.50	Epiphyte	Dense forest	Non-endemic
Coelogyne prolifera	Gongshan	1540	14.84	1738.42	78.47	860.43	0.50	Epiphyte	Dense forest	Non-endemic
Coelogyne prolifera	Xichou	1550	15.63	1267.54	82.98	985.97	0.78	Epiphyte	Dense forest	Non-endemic
Coelogyne prolifera	Xichou	1600	15.33	1267.54	82.98	985.97	0.78	Epiphyte	Dense forest	Non-endemic
Coelogyne prolifera	Menghai	1600	15.96	1314.38	80.83	1150.91	0.88	Epiphyte	Shrubland	Non-endemic
Coelogyne prolifera	Gongshan	1700	13.88	1738.42	78.47	860.43	0.50	Epiphyte	Dense forest	Non-endemic
Coelogyne prolifera	Menghai	1760	15.00	1314.38	80.83	1150.91	0.88	Epiphyte	Dense forest	Non-endemic
Coelogyne prolifera	Menghai	2000	13.56	1314.38	80.83	1150.91	0.88	Epiphyte	Dense forest	Non-endemic
Coelogyne prolifera	Fugong	2000	12.06	1441.43	79.99	906.18	0.63	Epiphyte	Dense forest	Non-endemic
Coelogyne prolifera	Lijiang	2100	14.47	982.53	63.24	1077.65	1.10	Epiphyte	Sparse forest	Non-endemic
Coelogyne prolifera	Lijiang	2300	13.27	982.53	63.24	1077.65	1.10	Epiphyte	Dense forest	Non-endemic
Coelogyne prolifera	Lijiang	2500	12.07	982.53	63.24	1077.65	1.10	Epiphyte	Sparse forest	Non-endemic
Coelogyne punctulata	Dali	1300	19.02	1082.70	68.61	1256.27	1.16	Epiphyte	Dense forest	Non-endemic
Coelogyne punctulata	Gongshan	1300	16.28	1738.42	78.47	860.43	0.50	Epiphyte	Dense forest	Non-endemic
Coelogyne punctulata	Gongshan	1300	16.28	1738.42	78.47	860.43	0.50	Epiphyte	Dense forest	Non-endemic
Coelogyne punctulata	Hekou	1300	15.95	1768.58	84.25	1166.26	0.66	Epiphyte	Dense forest	Non-endemic
Coelogyne punctulata	Gongshan	1310	16.22	1738.42	78.47	860.43	0.50	Epiphyte	Dense forest	Non-endemic
Coelogyne punctulata	Gongshan	1320	16.16	1738.42	78.47	860.43	0.50	Epiphyte	Dense forest	Non-endemic
Coelogyne punctulata	Gongshan	1350	15.98	1738.42	78.47	860.43	0.50	Epiphyte	Dense forest	Non-endemic
Coelogyne punctulata	Gongshan	1350	15.98	1738.42	78.47	860.43	0.50	Epiphyte	Dense forest	Non-endemic
Coelogyne punctulata	Gongshan	1380	15.80	1738.42	78.47	860.43	0.50	Epiphyte	Dense forest	Non-endemic
Coelogyne punctulata	Gongshan	1400	15.68	1738.42	78.47	860.43	0.50	Epiphyte	Dense forest	Non-endemic

Coelogyne punctulata	Gongshan	1400	15.68	1738.42	78.47	860.43	0.50	Epiphyte	Dense forest	Non-endemic
Coelogyne punctulata	Xichou	1550	15.63	1267.54	82.98	985.97	0.78	Epiphyte	Dense forest	Non-endemic
Coelogyne punctulata	Gongshan	1600	14.48	1738.42	78.47	860.43	0.50	Epiphyte	Dense forest	Non-endemic
Coelogyne punctulata	Gengma	1700	15.65	1327.70	77.28	1181.78	0.89	Epiphyte	Dense forest	Non-endemic
Coelogyne punctulata	Gongshan	1950	12.38	1738.42	78.47	860.43	0.50	Epiphyte	Dense forest	Non-endemic
Coelogyne punctulata	Lancang	2000	13.70	1596.50	77.88	1183.80	0.74	Epiphyte	Dense forest	Non-endemic
Coelogyne punctulata	Gongshan	2000	12.08	1738.42	78.47	860.43	0.50	Epiphyte	Dense forest	Non-endemic
Coelogyne punctulata	Gongshan	2000	12.08	1738.42	78.47	860.43	0.50	Epiphyte	Dense forest	Non-endemic
Coelogyne punctulata	Fugong	2000	12.06	1441.43	79.99	906.18	0.63	Epiphyte	Dense forest	Non-endemic
Coelogyne punctulata	Gongshan	2000	12.08	1738.42	78.47	860.43	0.50	Epiphyte	Dense forest	Non-endemic
Coelogyne punctulata	Maguan	2050	12.71	1330.52	83.25	1086.43	0.82	Epiphyte	Dense forest	Non-endemic
Coelogyne punctulata	Gongshan	2080	11.60	1738.42	78.47	860.43	0.50	Epiphyte	Dense forest	Non-endemic
Coelogyne punctulata	Gongshan	2100	11.48	1738.42	78.47	860.43	0.50	Epiphyte	Dense forest	Non-endemic
Coelogyne punctulata	Jinping	2200	12.29	2305.17	83.81	1030.46	0.45	Epiphyte	Dense forest	Non-endemic
Coelogyne punctulata	Jingdong	2200	12.38	1128.40	76.80	1142.11	1.01	Epiphyte	Dense forest	Non-endemic
Coelogyne punctulata	Wenshan	2200	12.46	988.87	76.70	1272.98	1.29	Epiphyte	Dense forest	Non-endemic
Coelogyne punctulata	Lushui	2300	12.25	1195.57	70.63	911.65	0.76	Epiphyte	Dense forest	Non-endemic
Coelogyne punctulata	Jingdong	2400	11.18	1128.40	76.80	1142.11	1.01	Epiphyte	Shrubland	Non-endemic
Coelogyne punctulata	Jingdong	2500	10.58	1128.40	76.80	1142.11	1.01	Epiphyte	Dense forest	Non-endemic
Coelogyne punctulata	Longling	2500	9.12	2098.66	84.65	973.45	0.46	Epiphyte	Dense forest	Non-endemic
Coelogyne punctulata	Jingdong	2520	10.46	1128.40	76.80	1142.11	1.01	Epiphyte	Dense forest	Non-endemic
Coelogyne punctulata	Deqin	2600	9.57	639.48	70.85	896.66	1.40	Epiphyte	Dense forest	Non-endemic
Coelogyne punctulata	Gongshan	2650	8.18	1738.42	78.47	860.43	0.50	Epiphyte	Dense forest	Non-endemic
Coelogyne punctulata	Gongshan	2650	8.18	1738.42	78.47	860.43	0.50	Epiphyte	Dense forest	Non-endemic
Coelogyne punctulata	Yongde	2650	11.23	1266.25	69.00	1283.63	1.01	Epiphyte	Forest edge	Non-endemic
Coelogyne punctulata	Yongde	2650	11.23	1266.25	69.00	1283.63	1.01	Epiphyte	Forest edge	Non-endemic
Coelogyne punctulata	Zhenkang	2700	8.64	1602.96	81.14	1089.09	0.68	Epiphyte	Dense forest	Non-endemic
Coelogyne punctulata	Gongshan	2700	7.88	1738.42	78.47	860.43	0.50	Epiphyte	Dense forest	Non-endemic
Coelogyne punctulata	Gongshan	2700	7.88	1738.42	78.47	860.43	0.50	Epiphyte	Dense forest	Non-endemic
Coelogyne punctulata	Fengqing	2700	10.00	1352.80	73.24	1172.52	0.87	Epiphyte	Dense forest	Non-endemic
Coelogyne punctulata	Zhenkang	2750	8.34	1602.96	81.14	1089.09	0.68	Epiphyte	Dense forest	Non-endemic
Coelogyne punctulata	Jingdong	2800	8.78	1128.40	76.80	1142.11	1.01	Epiphyte	Dense forest	Non-endemic
Coelogyne punctulata	Dali	2800	10.02	1082.70	68.61	1256.27	1.16	Epiphyte	Dense forest	Non-endemic
Coelogyne punctulata	Jingdong	2800	8.78	1128.40	76.80	1142.11	1.01	Epiphyte	Forest edge	Non-endemic
Coelogyne punctulata	Weixi	2900	8.03	970.70	69.95	1021.03	1.05	Epiphyte	Dense forest	Non-endemic
Coelogyne punctulata	Deqin	2900	7.77	639.48	70.85	896.66	1.40	Epiphyte	Dense forest	Non-endemic

Coelogyne punctulata	Jingdong	2900	8.18	1128.40	76.80	1142.11	1.01	Epiphyte	Sparse forest	Non-endemic
Coelogyne sanderae	Zhenkang	1000	18.84	1602.96	81.14	1089.09	0.68	Epiphyte	Dense forest	Non-endemic
Coelogyne sanderae	Zhenkang	1200	17.64	1602.96	81.14	1089.09	0.68	Epiphyte	Dense forest	Non-endemic
Coelogyne sanderae	Hekou	1300	15.95	1768.58	84.25	1166.26	0.66	Epiphyte	Dense forest	Non-endemic
Coelogyne sanderae	Lancang	1400	17.30	1596.50	77.88	1183.80	0.74	Epiphyte	Dense forest	Non-endemic
Coelogyne sanderae	Zhenkang	1600	15.24	1602.96	81.14	1089.09	0.68	Epiphyte	Dense forest	Non-endemic
Coelogyne sanderae	Zhenkang	1640	15.00	1602.96	81.14	1089.09	0.68	Epiphyte	Dense forest	Non-endemic
Coelogyne sanderae	Gengma	1700	15.47	1327.70	77.28	1181.78	0.89	Epiphyte	Dense forest	Non-endemic
Coelogyne sanderae	Luxi	1750	14.58	1650.33	79.33	1183.39	0.72	Epiphyte	Dense forest	Non-endemic
Coelogyne sanderae	Cangyuan	1800	14.42	1733.34	81.63	1060.82	0.61	Epiphyte	Dense forest	Non-endemic
Coelogyne sanderae	Fengqing	2250	12.70	1352.80	73.24	1172.52	0.87	Epiphyte	Dense forest	Non-endemic
Coelogyne schultesii	Gongshan	1700	13.88	1738.42	78.47	860.43	0.50	Epiphyte	Shrubland	Non-endemic
Coelogyne suaveolens	Mengla	600	21.60	1514.70	84.25	1146.68	0.76	Epiphyte	Dense forest	Non-endemic
Coelogyne viscosa	Shuangjiang	750	21.48	1006.64	75.26	1272.13	1.26	Epiphyte	Sparse forest	Non-endemic
Coelogyne viscosa	Mengla	1000	19.20	1514.70	84.25	1146.68	0.76	Epiphyte	Dense forest	Non-endemic
Coelogyne viscosa	Jinghong	1440	17.08	1161.08	80.46	1256.19	1.08	Epiphyte	Sparse forest	Non-endemic
Coelogyne viscosa	Zhenyuan	2000	14.24	1254.48	77.40	1138.06	0.91	Epiphyte	Dense forest	Non-endemic
Coelogyne wrymbosa	Yongde	1090	20.59	1266.25	69.00	1283.63	1.01	Epiphyte	Dense forest	Non-endemic
Collabium chinense	Pingbian	840	19.78	1648.57	86.34	990.16	0.60	Epiphyte	Dense forest	Non-endemic
Collabium formosanum	Luchun	1600	17.10	2013.54	78.64	1151.56	0.57	Epiphyte	Dense forest	Non-endemic
Collabium formosanum	Pingbian	1600	15.22	1648.57	86.34	990.16	0.60	Terrestrial	Dense forest	Non-endemic
Collabium formosanum	Xichou	1600	15.33	1267.54	82.98	985.97	0.78	Terrestrial	Dense forest	Non-endemic
Collabium formosanum	Xichou	1750	14.43	1267.54	82.98	985.97	0.78	Terrestrial	Dense forest	Non-endemic
Collabium formosanum	Lushui	2200	12.85	1195.57	70.63	911.65	0.76	Terrestrial	Dense forest	Non-endemic
Collabium formosanum	Lushui	2400	11.65	1195.57	70.63	911.65	0.76	Terrestrial	Dense forest	Non-endemic
Corybas taliensis	Yongde	2700	10.93	1266.25	69.00	1283.63	1.01	Terrestrial	Sparse forest	Non-endemic
Corybas taliensis	Yongde	3060	8.77	1266.25	69.00	1283.63	1.01	Terrestrial	Sparse forest	Non-endemic
Corymborkis veratrifolia	Hekou	150	22.85	1768.58	84.25	1166.26	0.66	Terrestrial	Dense forest	Non-endemic
Corymborkis veratrifolia	Cangyuan	650	21.32	1733.34	81.63	1060.82	0.61	Terrestrial	Dense forest	Non-endemic
Corymborkis veratrifolia	Maguan	900	19.61	1330.52	83.25	1086.43	0.82	Terrestrial	Dense forest	Non-endemic
Corymborkis veratrifolia	Jinghong	1000	19.72	1161.08	80.46	1256.19	1.08	Terrestrial	Dense forest	Non-endemic
Cremastra appendiculata	Jinghong	540	22.48	1161.08	80.46	1256.19	1.08	Terrestrial	Grassy slope	Non-endemic
Cremastra appendiculata	Fengqing	1200	19.00	1352.80	73.24	1172.52	0.87	Terrestrial	Dense forest	Non-endemic
Cremastra appendiculata	Suijiang	1500	11.33	917.85	78.74	923.40	1.01	Terrestrial	Dense forest	Non-endemic
Cremastra appendiculata	Yongshan	1900	10.30	665.40	74.22	968.77	1.46	Terrestrial	Dense forest	Non-endemic
Cremastra appendiculata	Gongshan	2000	12.08	1738.42	78.47	860.43	0.50	Terrestrial	Dense forest	Non-endemic

Cremastra appendiculata	Eryuan	2100	13.93	745.16	68.27	1210.94	1.63	Terrestrial	Dense forest	Non-endemic
Cremastra appendiculata	Heqing	2200	13.59	977.00	65.16	1211.84	1.24	Terrestrial	Dense forest	Non-endemic
Cremastra appendiculata	Yangbi	2300	12.19	1044.23	72.19	1161.15	1.11	Terrestrial	Dense forest	Non-endemic
Cremastra appendiculata	Eryuan	2400	12.13	745.16	68.27	1210.94	1.63	Terrestrial	Dense forest	Non-endemic
Cremastra appendiculata	Tengchong	2600	9.40	1501.45	78.06	857.93	0.57	Terrestrial	Dense forest	Non-endemic
Cryptochilus luteus	Malipo	1000	18.26	1063.54	85.83	1053.97	0.99	Epiphyte	Dense forest	Non-endemic
Cryptochilus luteus	Pingbian	1600	15.22	1648.57	86.34	990.16	0.60	Epiphyte	Dense forest	Non-endemic
Cryptochilus luteus	Jinping	1660	15.53	2305.17	83.81	1030.46	0.45	Epiphyte	Dense forest	Non-endemic
Cryptochilus luteus	Jinping	1730	15.11	2305.17	83.81	1030.46	0.45	Epiphyte	Dense forest	Non-endemic
Cryptochilus luteus	Malipo	1800	13.46	1063.54	85.83	1053.97	0.99	Epiphyte	Dense forest	Non-endemic
Cryptochilus luteus	Pingbian	1900	13.42	1648.57	86.34	990.16	0.60	Epiphyte	Dense forest	Non-endemic
Cryptochilus luteus	Gongshan	2040	11.84	1738.42	78.47	860.43	0.50	Epiphyte	Dense forest	Non-endemic
Cryptochilus luteus	Luchun	2300	12.90	2013.54	78.64	1151.56	0.57	Epiphyte	Dense forest	Non-endemic
Cymbidium aloifolium	Hekou	140	22.91	1768.58	84.25	1166.26	0.66	Epiphyte	Dense forest	Non-endemic
Cymbidium aloifolium	Hekou	150	22.85	1768.58	84.25	1166.26	0.66	Epiphyte	Dense forest	Non-endemic
Cymbidium aloifolium	Pingbian	340	22.78	1648.57	86.34	990.16	0.60	Epiphyte	Dense forest	Non-endemic
Cymbidium aloifolium	Jinghong	600	22.12	1161.08	80.46	1256.19	1.08	Epiphyte	Dense forest	Non-endemic
Cymbidium aloifolium	Mengla	680	21.12	1514.70	84.25	1146.68	0.76	Epiphyte	Dense forest	Non-endemic
Cymbidium aloifolium	Jinghong	710	21.46	1161.08	80.46	1256.19	1.08	Epiphyte	Dense forest	Non-endemic
Cymbidium aloifolium	Jinghong	840	20.68	1161.08	80.46	1256.19	1.08	Epiphyte	Dense forest	Non-endemic
Cymbidium aloifolium	Jinghong	850	20.62	1161.08	80.46	1256.19	1.08	Epiphyte	Sparse forest	Non-endemic
Cymbidium aloifolium	Menghai	1000	19.56	1314.38	80.83	1150.91	0.88	Epiphyte	Dense forest	Non-endemic
Cymbidium bicolor subsp.obtusum	Hekou	180	22.67	1768.58	84.25	1166.26	0.66	Epiphyte	Forest edge	Non-endemic
Cymbidium bicolor subsp.obtusum	Hekou	220	22.43	1768.58	84.25	1166.26	0.66	Epiphyte	Dense forest	Non-endemic
Cymbidium bicolor subsp.obtusum	Jinping	350	23.39	2305.17	83.81	1030.46	0.45	Epiphyte	Dense forest	Non-endemic
Cymbidium bicolor subsp.obtusum	Mengla	560	21.84	1514.70	84.25	1146.68	0.76	Epiphyte	Sparse forest	Non-endemic
Cymbidium bicolor subsp.obtusum	Jinghong	650	21.82	1161.08	80.46	1256.19	1.08	Epiphyte	Forest edge	Non-endemic
Cymbidium bicolor subsp.obtusum	Jinghong	700	21.52	1161.08	80.46	1256.19	1.08	Epiphyte	Shrubland	Non-endemic
Cymbidium bicolor subsp.obtusum	Jinghong	850	20.62	1161.08	80.46	1256.19	1.08	Epiphyte	Dense forest	Non-endemic
Cymbidium bicolor subsp.obtusum	Jinghong	850	20.62	1161.08	80.46	1256.19	1.08	Epiphyte	Dense forest	Non-endemic
Cymbidium bicolor subsp.obtusum	Jinghong	1020	19.60	1161.08	80.46	1256.19	1.08	Epiphyte	Dense forest	Non-endemic
Cymbidium bicolor subsp.obtusum	Jinghong	1100	19.12	1161.08	80.46	1256.19	1.08	Epiphyte	Dense forest	Non-endemic
Cymbidium bicolor subsp.obtusum	Menghai	1180	18.48	1314.38	80.83	1150.91	0.88	Epiphyte	Dense forest	Non-endemic
Cymbidium bicolor subsp.obtusum	Luchun	1200	19.50	2013.54	78.64	1151.56	0.57	Epiphyte	Forest edge	Non-endemic
Cymbidium bicolor subsp.obtusum	Jingdong	1500	16.58	1128.40	76.80	1142.11	1.01	Epiphyte	Forest edge	Non-endemic
Cymbidium bicolor subsp.obtusum	Zhenkang	1600	15.24	1602.96	81.14	1089.09	0.68	Epiphyte	Dense forest	Non-endemic

Cymbidium bicolor subsp.obtusum	Menghai	1600	15.96	1314.38	80.83	1150.91	0.88	Epiphyte	Shrubland	Non-endemic
Cymbidium cyperifolium	Funing	750	19.12	1161.58	79.07	1147.17	0.99	Epiphyte	Dense forest	Non-endemic
Cymbidium cyperifolium	Funing	950	17.92	1161.58	79.07	1147.17	0.99	Epiphyte	Dense forest	Non-endemic
Cymbidium cyperifolium	Xichou	1000	18.93	1267.54	82.98	985.97	0.78	Epiphyte	Dense forest	Non-endemic
Cymbidium cyperifolium	Xichou	1000	18.93	1267.54	82.98	985.97	0.78	Epiphyte	Sparse forest	Non-endemic
Cymbidium cyperifolium	Yanshan	1200	18.26	1003.57	79.42	1172.21	1.17	Epiphyte	Dense forest	Non-endemic
Cymbidium cyperifolium	Malipo	1200	17.06	1063.54	85.83	1053.97	0.99	Epiphyte	Open site	Non-endemic
Cymbidium cyperifolium	Guangnan	1270	16.63	1044.26	78.94	1090.35	1.04	Epiphyte	Open site	Non-endemic
Cymbidium cyperifolium	Maguan	1300	17.21	1330.52	83.25	1086.43	0.82	Epiphyte	Dense forest	Non-endemic
Cymbidium cyperifolium	Pingbian	1400	16.42	1648.57	86.34	990.16	0.60	Epiphyte	Dense forest	Non-endemic
Cymbidium cyperifolium	Malipo	1400	15.86	1063.54	85.83	1053.97	0.99	Epiphyte	Dense forest	Non-endemic
Cymbidium cyperifolium	Xichou	1450	16.23	1267.54	82.98	985.97	0.78	Epiphyte	Dense forest	Non-endemic
Cymbidium cyperifolium	Pingbian	1500	15.82	1648.57	86.34	990.16	0.60	Epiphyte	Dense forest	Non-endemic
Cymbidium cyperifolium	Malipo	1500	15.26	1063.54	85.83	1053.97	0.99	Epiphyte	Dense forest	Non-endemic
Cymbidium cyperifolium	Xichou	1550	15.63	1267.54	82.98	985.97	0.78	Epiphyte	Dense forest	Non-endemic
Cymbidium cyperifolium	Malipo	1600	14.66	1063.54	85.83	1053.97	0.99	Epiphyte	Dense forest	Non-endemic
Cymbidium cyperifolium	Mengzi	1600	16.86	855.35	71.82	1434.56	1.68	Epiphyte	Open site	Non-endemic
Cymbidium cyperifolium	Malipo	1700	14.06	1063.54	85.83	1053.97	0.99	Epiphyte	Sparse forest	Non-endemic
Cymbidium cyperifolium	Mengzi	1745	15.99	855.35	71.82	1434.56	1.68	Epiphyte	Dense forest	Non-endemic
Cymbidium cyperifolium	Nanjian	2378	13.31	735.93	63.17	1535.84	2.09	Epiphyte	Dense forest	Non-endemic
Cymbidium dayanum	Gongshan	1700	13.88	1738.42	78.47	860.43	0.50	Epiphyte	Sparse forest	Non-endemic
Cymbidium dayanum	Yongde	2690	10.99	1266.25	69.00	1283.63	1.01	Epiphyte	Sparse forest	Non-endemic
Cymbidium eburneum	Fengqing	2000	14.20	1352.80	73.24	1172.52	0.87	Epiphyte	Forest edge	Non-endemic
Cymbidium eburneum	Jingdong	2100	12.98	1128.40	76.80	1142.11	1.01	Epiphyte	Forest edge	Non-endemic
Cymbidium elegans	Gongshan	1700	13.88	1738.42	78.47	860.43	0.50	Epiphyte	Dense forest	Non-endemic
Cymbidium elegans	Wenshan	1700	15.46	988.87	76.70	1272.98	1.29	Epiphyte	Sparse forest	Non-endemic
Cymbidium elegans	Gongshan	1800	13.28	1738.42	78.47	860.43	0.50	Epiphyte	Sparse forest	Non-endemic
Cymbidium elegans	Jingdong	1900	14.18	1128.40	76.80	1142.11	1.01	Epiphyte	Dense forest	Non-endemic
Cymbidium elegans	Gongshan	1900	12.68	1738.42	78.47	860.43	0.50	Epiphyte	Dense forest	Non-endemic
Cymbidium elegans	Fugong	2000	12.06	1441.43	79.99	906.18	0.63	Epiphyte	Dense forest	Non-endemic
Cymbidium elegans	Luchun	2100	14.10	2013.54	78.64	1151.56	0.57	Epiphyte	Dense forest	Non-endemic
Cymbidium elegans	Pingbian	2100	12.22	1648.57	86.34	990.16	0.60	Epiphyte	Sparse forest	Non-endemic
Cymbidium elegans	Gongshan	2200	10.88	1738.42	78.47	860.43	0.50	Epiphyte	Dense forest	Non-endemic
Cymbidium elegans	Gongshan	2200	10.88	1738.42	78.47	860.43	0.50	Epiphyte	Dense forest	Non-endemic
Cymbidium elegans	Jinping	2400	11.09	2305.17	83.81	1030.46	0.45	Epiphyte	Dense forest	Non-endemic
Cymbidium elegans	Fugong	2800	7.26	1441.43	79.99	906.18	0.63	Epiphyte	Dense forest	Non-endemic

Cymbidium elegans	Jingdong	2900	8.18	1128.40	76.80	1142.11	1.01	Epiphyte	Dense forest	Non-endemic
Cymbidium elegans	Jingdong	2600	9.98	1128.40	76.80	1142.11	1.01	Epiphyte	Sparse forest	Non-endemic
Cymbidium elegans	Lincang	2650	10.62	1165.84	72.08	1167.73	1.00	Epiphyte	Dense forest	Non-endemic
Cymbidium ensifolium	Mengla	500	22.20	1514.70	84.25	1146.68	0.76	Terrestrial	Dense forest	Non-endemic
Cymbidium ensifolium	Mengla	600	21.60	1514.70	84.25	1146.68	0.76	Terrestrial	Dense forest	Non-endemic
Cymbidium ensifolium	Funing	700	19.42	1161.58	79.07	1147.17	0.99	Terrestrial	Dense forest	Non-endemic
Cymbidium ensifolium	Menghai	1100	18.96	1314.38	80.83	1150.91	0.88	Terrestrial	Dense forest	Non-endemic
Cymbidium ensifolium	Menghai	1180	18.48	1314.38	80.83	1150.91	0.88	Terrestrial	Dense forest	Non-endemic
Cymbidium ensifolium	Menghai	1200	18.36	1314.38	80.83	1150.91	0.88	Terrestrial	Sparse forest	Non-endemic
Cymbidium ensifolium	Guangnan	1250	16.75	1044.26	78.94	1090.35	1.04	Terrestrial	Shrubland	Non-endemic
Cymbidium ensifolium	Guangnan	1250	16.75	1044.26	78.94	1090.35	1.04	Terrestrial	Shrubland	Non-endemic
Cymbidium ensifolium	Menghai	1300	17.76	1314.38	80.83	1150.91	0.88	Terrestrial	Dense forest	Non-endemic
Cymbidium ensifolium	Menghai	1300	17.76	1314.38	80.83	1150.91	0.88	Terrestrial	Sparse forest	Non-endemic
Cymbidium ensifolium	Pingbian	1400	16.42	1648.57	86.34	990.16	0.60	Terrestrial	Dense forest	Non-endemic
Cymbidium ensifolium	Jingdong	1600	15.98	1128.40	76.80	1142.11	1.01	Terrestrial	Dense forest	Non-endemic
Cymbidium ensifolium	Jingdong	2100	12.98	1128.40	76.80	1142.11	1.01	Terrestrial	Dense forest	Non-endemic
Cymbidium ensifolium	Jingdong	2500	10.58	1128.40	76.80	1142.11	1.01	Terrestrial	Dense forest	Non-endemic
Cymbidium ensifolium	Jingdong	2500	10.58	1128.40	76.80	1142.11	1.01	Terrestrial	Grassy slope	Non-endemic
Cymbidium ensifolium	Deqin	3300	5.37	639.48	70.85	896.66	1.40	Terrestrial	Grassy slope	Non-endemic
Cymbidium erythraeum	Xichou	1490	15.99	1267.54	82.98	985.97	0.78	Epiphyte	Dense forest	Non-endemic
Cymbidium erythraeum	Pingbian	1500	15.82	1648.57	86.34	990.16	0.60	Epiphyte	Grassy slope	Non-endemic
Cymbidium erythraeum	Gongshan	1700	13.88	1738.42	78.47	860.43	0.50	Epiphyte	Sparse forest	Non-endemic
Cymbidium erythraeum	Jiangchuan	1750	15.50	872.74	74.72	1181.99	1.35	Epiphyte	Dense forest	Non-endemic
Cymbidium erythraeum	Jinghong	1800	14.92	1161.08	80.46	1256.19	1.08	Epiphyte	Dense forest	Non-endemic
Cymbidium erythraeum	Eshan	1800	14.41	938.39	78.62	1123.48	1.20	Epiphyte	Forest edge	Non-endemic
Cymbidium erythraeum	Yuanjiang	1850	15.08	805.59	69.08	1652.32	2.05	Epiphyte	Dense forest	Non-endemic
Cymbidium erythraeum	Fuming	2100	13.37	871.62	71.93	1209.56	1.39	Epiphyte	Dense forest	Non-endemic
Cymbidium erythraeum	Yongde	2400	12.73	1266.25	69.00	1283.63	1.01	Epiphyte	Grassy slope	Non-endemic
Cymbidium erythraeum	Lincang	2800	9.72	1165.84	72.08	1167.73	1.00	Epiphyte	Dense forest	Non-endemic
Cymbidium faberi	Gongshan	2000	12.08	1738.42	78.47	860.43	0.50	Terrestrial	Dense forest	Non-endemic
Cymbidium faberi	Gongshan	2120	11.36	1738.42	78.47	860.43	0.50	Terrestrial	Dense forest	Non-endemic
Cymbidium faberi	Lijiang	2160	14.11	982.53	63.24	1077.65	1.10	Terrestrial	Dense forest	Non-endemic
Cymbidium faberi	Kunming	2200	13.08	1019.14	72.30	1197.62	1.18	Terrestrial	Dense forest	Non-endemic
Cymbidium faberi	Weixi	2560	10.07	970.70	69.95	1021.03	1.05	Terrestrial	Dense forest	Non-endemic
Cymbidium faberi	Deqin	2700	8.97	639.48	70.85	896.66	1.40	Terrestrial	Dense forest	Non-endemic
Cymbidium faberi	Weixi	3000	7.43	970.70	69.95	1021.03	1.05	Terrestrial	Dense forest	Non-endemic

Cymbidium faberi	Zhongdian	3240	6.00	641.73	69.05	914.89	1.43	Terrestrial	Shrubland	Non-endemic
Cymbidium floribundum	Guangnan	800	19.45	1044.26	78.94	1090.35	1.04	Epiphyte	Shrubland	Non-endemic
Cymbidium floribundum	Funing	1000	17.62	1161.58	79.07	1147.17	0.99	Epiphyte	Sparse forest	Non-endemic
Cymbidium floribundum	Yanshan	1200	18.26	1003.57	79.42	1172.21	1.17	Epiphyte	Dense forest	Non-endemic
Cymbidium floribundum	Menghai	1300	17.76	1314.38	80.83	1150.91	0.88	Epiphyte	Sparse forest	Non-endemic
Cymbidium floribundum	Xichou	1300	17.13	1267.54	82.98	985.97	0.78	Epiphyte	Sparse forest	Non-endemic
Cymbidium floribundum	Guangnan	1360	16.09	1044.26	78.94	1090.35	1.04	Epiphyte	Sparse forest	Non-endemic
Cymbidium floribundum	Malipo	1500	15.26	1063.54	85.83	1053.97	0.99	Epiphyte	Dense forest	Non-endemic
Cymbidium floribundum	Xichou	1500	15.93	1267.54	82.98	985.97	0.78	Epiphyte	Dense forest	Non-endemic
Cymbidium floribundum	Wenshan	1950	13.96	988.87	76.70	1272.98	1.29	Epiphyte	Dense forest	Non-endemic
Cymbidium floribundum	Deqin	2000	13.17	639.48	70.85	896.66	1.40	Epiphyte	Dense forest	Non-endemic
Cymbidium floribundum	Fugong	2000	12.06	1441.43	79.99	906.18	0.63	Epiphyte	Dense forest	Non-endemic
Cymbidium floribundum	Weixi	2000	13.43	970.70	69.95	1021.03	1.05	Epiphyte	Dense forest	Non-endemic
Cymbidium floribundum	Gongshan	2000	12.08	1738.42	78.47	860.43	0.50	Epiphyte	Shrubland	Non-endemic
Cymbidium floribundum	Weixi	2000	13.43	970.70	69.95	1021.03	1.05	Epiphyte	Sparse forest	Non-endemic
Cymbidium floribundum	Gongshan	2300	10.28	1738.42	78.47	860.43	0.50	Epiphyte	Dense forest	Non-endemic
Cymbidium floribundum	Weixi	2300	11.63	970.70	69.95	1021.03	1.05	Epiphyte	Dense forest	Non-endemic
Cymbidium floribundum	Lijiang	2400	12.67	982.53	63.24	1077.65	1.10	Epiphyte	Open site	Non-endemic
Cymbidium floribundum	Zhongdian	2450	10.74	641.73	69.05	914.89	1.43	Epiphyte	Dense forest	Non-endemic
Cymbidium floribundum	Gongshan	2500	9.08	1738.42	78.47	860.43	0.50	Epiphyte	Dense forest	Non-endemic
Cymbidium floribundum	Gongshan	2500	9.08	1738.42	78.47	860.43	0.50	Epiphyte	Dense forest	Non-endemic
Cymbidium floribundum	Gongshan	2600	8.48	1738.42	78.47	860.43	0.50	Epiphyte	Dense forest	Non-endemic
Cymbidium floribundum	Deqin	2600	9.57	639.48	70.85	896.66	1.40	Epiphyte	Dense forest	Non-endemic
Cymbidium floribundum	Deqin	2600	9.57	639.48	70.85	896.66	1.40	Epiphyte	Sparse forest	Non-endemic
Cymbidium floribundum	Gongshan	2700	7.88	1738.42	78.47	860.43	0.50	Epiphyte	Dense forest	Non-endemic
Cymbidium floribundum	Gongshan	2900	6.68	1738.42	78.47	860.43	0.50	Epiphyte	Dense forest	Non-endemic
Cymbidium goeringii	Guangnan	1330	16.27	1044.26	78.94	1090.35	1.04	Terrestrial	Dense forest	Non-endemic
Cymbidium goeringii	Yongde	1600	17.53	1266.25	69.00	1283.63	1.01	Terrestrial	Dense forest	Non-endemic
Cymbidium goeringii	Nanjian	1980	15.69	735.93	63.17	1535.84	2.09	Terrestrial	Dense forest	Non-endemic
Cymbidium goeringii var.serratum	Kunming	1900	14.88	1019.14	72.30	1197.62	1.18	Terrestrial	Dense forest	Non-endemic
Cymbidium hookerianum	Pingbian	1040	18.58	1648.57	86.34	990.16	0.60	Epiphyte	Sparse forest	Non-endemic
Cymbidium hookerianum	Jinping	1060	19.13	2305.17	83.81	1030.46	0.45	Epiphyte	Dense forest	Non-endemic
Cymbidium hookerianum	Hekou	1200	16.55	1768.58	84.25	1166.26	0.66	Epiphyte	Sparse forest	Non-endemic
Cymbidium hookerianum	Pingbian	1300	17.02	1648.57	86.34	990.16	0.60	Epiphyte	Forest edge	Non-endemic
Cymbidium hookerianum	Menghai	1300	17.76	1314.38	80.83	1150.91	0.88	Epiphyte	Forest edge	Non-endemic
Cymbidium hookerianum	Menghai	1350	17.46	1314.38	80.83	1150.91	0.88	Epiphyte	Dense forest	Non-endemic

Cymbidium hookerianum	Gongshan	1400	15.68	1738.42	78.47	860.43	0.50	Epiphyte	Dense forest	Non-endemic
Cymbidium hookerianum	Shuangjiang	1400	17.58	1006.64	75.26	1272.13	1.26	Epiphyte	Dense forest	Non-endemic
Cymbidium hookerianum	Gongshan	1400	15.68	1738.42	78.47	860.43	0.50	Epiphyte	Dense forest	Non-endemic
Cymbidium hookerianum	Cangyuan	1500	16.22	1733.34	81.63	1060.82	0.61	Epiphyte	Dense forest	Non-endemic
Cymbidium hookerianum	Gongshan	1500	15.08	1738.42	78.47	860.43	0.50	Epiphyte	Dense forest	Non-endemic
Cymbidium hookerianum	Gongshan	1700	13.88	1738.42	78.47	860.43	0.50	Epiphyte	Shrubland	Non-endemic
Cymbidium hookerianum	Longling	1800	13.32	2098.66	84.65	973.45	0.46	Epiphyte	Dense forest	Non-endemic
Cymbidium hookerianum	Gongshan	1800	13.28	1738.42	78.47	860.43	0.50	Epiphyte	Dense forest	Non-endemic
Cymbidium hookerianum	Gongshan	1800	13.28	1738.42	78.47	860.43	0.50	Epiphyte	Dense forest	Non-endemic
Cymbidium hookerianum	Wenshan	1800	14.86	988.87	76.70	1272.98	1.29	Epiphyte	Sparse forest	Non-endemic
Cymbidium hookerianum	Gongshan	1880	12.80	1738.42	78.47	860.43	0.50	Epiphyte	Dense forest	Non-endemic
Cymbidium hookerianum	Jingdong	1900	14.18	1128.40	76.80	1142.11	1.01	Epiphyte	Dense forest	Non-endemic
Cymbidium hookerianum	Jingdong	2100	12.98	1128.40	76.80	1142.11	1.01	Epiphyte	Dense forest	Non-endemic
Cymbidium hookerianum	Gongshan	2100	11.48	1738.42	78.47	860.43	0.50	Epiphyte	Dense forest	Non-endemic
Cymbidium hookerianum	Nanjian	2171	14.55	735.93	63.17	1535.84	2.09	Epiphyte	Sparse forest	Non-endemic
Cymbidium hookerianum	Pingbian	2180	11.74	1648.57	86.34	990.16	0.60	Epiphyte	Shrubland	Non-endemic
Cymbidium hookerianum	Gongshan	2200	10.88	1738.42	78.47	860.43	0.50	Epiphyte	Dense forest	Non-endemic
Cymbidium hookerianum	Gongshan	2200	10.88	1738.42	78.47	860.43	0.50	Epiphyte	Dense forest	Non-endemic
Cymbidium hookerianum	Gongshan	2300	10.28	1738.42	78.47	860.43	0.50	Epiphyte	Dense forest	Non-endemic
Cymbidium hookerianum	Yongde	2600	11.53	1266.25	69.00	1283.63	1.01	Epiphyte	Dense forest	Non-endemic
Cymbidium hookerianum	Gongshan	3000	6.08	1738.42	78.47	860.43	0.50	Epiphyte	Dense forest	Non-endemic
Cymbidium iridioides	Luchun	750	22.20	2013.54	78.64	1151.56	0.57	Epiphyte	Dense forest	Non-endemic
Cymbidium iridioides	Yanshan	1100	18.86	1003.57	79.42	1172.21	1.17	Epiphyte	Dense forest	Non-endemic
Cymbidium iridioides	Cangyuan	1150	18.32	1733.34	81.63	1060.82	0.61	Epiphyte	Dense forest	Non-endemic
Cymbidium iridioides	Pingbian	1300	17.02	1648.57	86.34	990.16	0.60	Epiphyte	Dense forest	Non-endemic
Cymbidium iridioides	Yuanyang	1300	18.51	1100.28	77.18	1242.17	1.13	Epiphyte	Sparse forest	Non-endemic
Cymbidium iridioides	Malipo	1500	15.26	1063.54	85.83	1053.97	0.99	Epiphyte	Dense forest	Non-endemic
Cymbidium iridioides	Longling	1600	14.52	2098.66	84.65	973.45	0.46	Epiphyte	Dense forest	Non-endemic
Cymbidium iridioides	Gongshan	1600	14.48	1738.42	78.47	860.43	0.50	Epiphyte	Forest edge	Non-endemic
Cymbidium iridioides	Zhenkang	1640	15.00	1602.96	81.14	1089.09	0.68	Epiphyte	Sparse forest	Non-endemic
Cymbidium iridioides	Menghai	1650	15.66	1314.38	80.83	1150.91	0.88	Epiphyte	Sparse forest	Non-endemic
Cymbidium iridioides	Pingbian	1700	14.62	1648.57	86.34	990.16	0.60	Epiphyte	Dense forest	Non-endemic
Cymbidium iridioides	Tengchong	1750	14.50	1501.45	78.06	857.93	0.57	Epiphyte	Shrubland	Non-endemic
Cymbidium iridioides	Tengchong	1800	14.20	1501.45	78.06	857.93	0.57	Epiphyte	Sparse forest	Non-endemic
Cymbidium iridioides	Jianshui	2000	14.55	793.10	71.73	1413.81	1.78	Epiphyte	Dense forest	Non-endemic
Cymbidium iridioides	Gongshan	2000	12.08	1738.42	78.47	860.43	0.50	Epiphyte	Dense forest	Non-endemic

Cymbidium iridioides	Lijiang	2100	14.47	982.53	63.24	1077.65	1.10	Epiphyte	Dense forest	Non-endemic
Cymbidium iridioides	Jingdong	2100	12.98	1128.40	76.80	1142.11	1.01	Epiphyte	Sparse forest	Non-endemic
Cymbidium iridioides	Gongshan	2200	10.88	1738.42	78.47	860.43	0.50	Epiphyte	Dense forest	Non-endemic
Cymbidium iridioides	Gongshan	2200	10.88	1738.42	78.47	860.43	0.50	Epiphyte	Forest edge	Non-endemic
Cymbidium iridioides	Gongshan	2200	10.88	1738.42	78.47	860.43	0.50	Epiphyte	Forest edge	Non-endemic
Cymbidium iridioides	Gongshan	2200	10.88	1738.42	78.47	860.43	0.50	Epiphyte	Sparse forest	Non-endemic
Cymbidium iridioides	Fugong	2300	10.26	1441.43	79.99	906.18	0.63	Epiphyte	Sparse forest	Non-endemic
Cymbidium iridioides	Gongshan	2500	9.08	1738.42	78.47	860.43	0.50	Epiphyte	Forest edge	Non-endemic
Cymbidium iridioides	Jingdong	2600	9.98	1128.40	76.80	1142.11	1.01	Epiphyte	Grassy slope	Non-endemic
Cymbidium iridioides	Deqin	2700	8.97	639.48	70.85	896.66	1.40	Epiphyte	Forest edge	Non-endemic
Cymbidium kanran	Pingbian	1500	15.82	1648.57	86.34	990.16	0.60	Terrestrial	Dense forest	Non-endemic
Cymbidium kanran	Gongshan	2000	12.08	1738.42	78.47	860.43	0.50	Terrestrial	Dense forest	Non-endemic
Cymbidium kanran	Gongshan	2100	11.48	1738.42	78.47	860.43	0.50	Terrestrial	Dense forest	Non-endemic
Cymbidium kanran	Kunming	2300	12.48	1019.14	72.30	1197.62	1.18	Terrestrial	Dense forest	Non-endemic
Cymbidium lancifolium	Xichou	870	19.71	1267.54	82.98	985.97	0.78	Terrestrial	Dense forest	Non-endemic
Cymbidium lancifolium	Yanshan	1100	18.86	1003.57	79.42	1172.21	1.17	Terrestrial	Dense forest	Non-endemic
Cymbidium lancifolium	Yanshan	1200	18.26	1003.57	79.42	1172.21	1.17	Terrestrial	Dense forest	Non-endemic
Cymbidium lancifolium	Yongde	1300	19.33	1266.25	69.00	1283.63	1.01	Terrestrial	Dense forest	Non-endemic
Cymbidium lancifolium	Yuanyang	1300	18.51	1100.28	77.18	1242.17	1.13	Terrestrial	Dense forest	Non-endemic
Cymbidium lancifolium	Pingbian	1400	16.42	1648.57	86.34	990.16	0.60	Terrestrial	Dense forest	Non-endemic
Cymbidium lancifolium	Gongshan	1450	15.38	1738.42	78.47	860.43	0.50	Terrestrial	Shrubland	Non-endemic
Cymbidium lancifolium	Xichou	1500	15.93	1267.54	82.98	985.97	0.78	terrestrial	Dense forest	Non-endemic
Cymbidium lancifolium	Xichou	1500	15.93	1267.54	82.98	985.97	0.78	Terrestrial	Dense forest	Non-endemic
Cymbidium lancifolium	Xichou	1600	15.33	1267.54	82.98	985.97	0.78	Terrestrial	Dense forest	Non-endemic
Cymbidium lancifolium	Gongshan	2000	12.08	1738.42	78.47	860.43	0.50	Terrestrial	Dense forest	Non-endemic
Cymbidium lancifolium	Weixi	2200	12.23	970.70	69.95	1021.03	1.05	Terrestrial	Dense forest	Non-endemic
Cymbidium lowianum	Menghai	1540	16.32	1314.38	80.83	1150.91	0.88	Epiphyte	Dense forest	Non-endemic
Cymbidium lowianum	Mengla	1800	14.40	1514.70	84.25	1146.68	0.76	Epiphyte	Dense forest	Non-endemic
Cymbidium lowianum	Wenshan	1950	13.96	988.87	76.70	1272.98	1.29	Epiphyte	Dense forest	Non-endemic
Cymbidium macrorhizon	Dongchuan	1700	15.63	1021.73	71.65	1186.61	1.16	Saprophyte	Dense forest	Non-endemic
Cymbidium mastersii	Jinghong	1650	15.82	1161.08	80.46	1256.19	1.08	Epiphyte	Sparse forest	Non-endemic
Cymbidium qiubeiense	Xichou	1500	15.93	1267.54	82.98	985.97	0.78	Epiphyte	Dense forest	Endemic
Cymbidium qiubeiense	Wenshan	1800	14.86	988.87	76.70	1272.98	1.29	Epiphyte	Dense forest	Endemic
Cymbidium sinense	Jinghong	800	20.92	1161.08	80.46	1256.19	1.08	Terrestrial	Dense forest	Non-endemic
Cymbidium sinense	Jinghong	1000	19.72	1161.08	80.46	1256.19	1.08	Terrestrial	Dense forest	Non-endemic
Cymbidium sinense	Xichou	1000	18.93	1267.54	82.98	985.97	0.78	Terrestrial	Shrubland	Non-endemic

Cymbidium sinense	Xichou	1200	17.73	1267.54	82.98	985.97	0.78	Terrestrial	Dense forest	Non-endemic
Cymbidium sinense	Xichou	1200	17.73	1267.54	82.98	985.97	0.78	Terrestrial	Dense forest	Non-endemic
Cymbidium sinense	Funing	1260	16.06	1161.58	79.07	1147.17	0.99	Terrestrial	Dense forest	Non-endemic
Cymbidium sinense	Xichou	1600	15.33	1267.54	82.98	985.97	0.78	Terrestrial	Dense forest	Non-endemic
Cymbidium tracyanum	Gongshan	1700	13.88	1738.42	78.47	860.43	0.50	Epiphyte	Dense forest	Non-endemic
Cymbidium tracyanum	Pingbian	1800	14.02	1648.57	86.34	990.16	0.60	Epiphyte	Dense forest	Non-endemic
Cymbidium tracyanum	Jingdong	2100	12.98	1128.40	76.80	1142.11	1.01	Epiphyte	Dense forest	Non-endemic
Cymbidium wilsonii	Jingdong	2500	10.58	1128.40	76.80	1142.11	1.01	Epiphyte	Shrubland	Endemic
Cypripedium bardolphianum	Weixi	3600	3.83	970.70	69.95	1021.03	1.05	Terrestrial	Grassy slope	Non-endemic
Cypripedium bardolphianum	Zhongdian	3700	3.24	641.73	69.05	914.89	1.43	Terrestrial	Sparse forest	Non-endemic
Cypripedium bardolphianum	Zhongdian	3900	2.04	641.73	69.05	914.89	1.43	Terrestrial	Grassy slope	Non-endemic
Cypripedium corrugatum	Lijiang	2500	12.07	982.53	63.24	1077.65	1.10	Terrestrial	Grassy slope	Non-endemic
Cypripedium corrugatum	Lijiang	2600	11.47	982.53	63.24	1077.65	1.10	Terrestrial	Forest edge	Non-endemic
Cypripedium corrugatum	Lijiang	2700	10.87	982.53	63.24	1077.65	1.10	Terrestrial	Grassy slope	Non-endemic
Cypripedium corrugatum	Zhongdian	2900	8.04	641.73	69.05	914.89	1.43	Terrestrial	Shrubland	Non-endemic
Cypripedium corrugatum	Lijiang	3000	9.07	982.53	63.24	1077.65	1.10	Terrestrial	Grassy slope	Non-endemic
Cypripedium corrugatum	Lijiang	3260	7.51	982.53	63.24	1077.65	1.10	Terrestrial	Forest edge	Non-endemic
Cypripedium corrugatum	Lijiang	3400	6.67	982.53	63.24	1077.65	1.10	Terrestrial	Meadow	Non-endemic
Cypripedium corrugatum	Zhongdian	3500	4.44	641.73	69.05	914.89	1.43	Terrestrial	Grassy slope	Non-endemic
Cypripedium corrugatum	Gongshan	3650	2.18	1738.42	78.47	860.43	0.50	Terrestrial	Meadow	Non-endemic
Cypripedium corrugatum	Zhongdian	3900	2.04	641.73	69.05	914.89	1.43	Terrestrial	Grassy slope	Non-endemic
Cypripedium corrugatum	Zhongdian	4000	1.44	641.73	69.05	914.89	1.43	Terrestrial	Shrubland	Non-endemic
Cypripedium dahenise	Lijiang	2800	10.27	982.53	63.24	1077.65	1.10	Terrestrial	Open site	Non-endemic
Cypripedium dahenise	Lijiang	3000	9.07	982.53	63.24	1077.65	1.10	Terrestrial	Open site	Non-endemic
Cypripedium dahenise	Zhongdian	3500	4.44	641.73	69.05	914.89	1.43	Terrestrial	Sparse forest	Non-endemic
Cypripedium elegans	Lijiang	3600	5.47	982.53	63.24	1077.65	1.10	Terrestrial	Meadow	Non-endemic
Cypripedium farreri	Lijiang	3100	8.47	982.53	63.24	1077.65	1.10	Terrestrial	Grassy slope	Non-endemic
Cypripedium flavum	Lijiang	2600	11.47	982.53	63.24	1077.65	1.10	Terrestrial	Open site	Non-endemic
Cypripedium flavum	Weixi	2600	9.83	970.70	69.95	1021.03	1.05	Terrestrial	Sparse forest	Non-endemic
Cypripedium flavum	Lijiang	2850	9.97	982.53	63.24	1077.65	1.10	Terrestrial	Meadow	Non-endemic
Cypripedium flavum	Dali	2850	9.72	1082.70	68.61	1256.27	1.16	Terrestrial	Shrubland	Non-endemic
Cypripedium flavum	Lijiang	2900	9.67	982.53	63.24	1077.65	1.10	Terrestrial	Grassy slope	Non-endemic
Cypripedium flavum	Gongshan	2950	6.38	1738.42	78.47	860.43	0.50	Terrestrial	Forest edge	Non-endemic
Cypripedium flavum	Gongshan	3000	6.08	1738.42	78.47	860.43	0.50	Terrestrial	Grassy slope	Non-endemic
Cypripedium flavum	Deqin	3000	7.17	639.48	70.85	896.66	1.40	Terrestrial	Grassy slope	Non-endemic
Cypripedium flavum	Lijiang	3000	9.07	982.53	63.24	1077.65	1.10	Terrestrial	Grassy slope	Non-endemic

Cypripedium flavum	Zhongdian	3000	7.44	641.73	69.05	914.89	1.43	Terrestrial	Shrubland	Non-endemic
Cypripedium flavum	Lijiang	3100	8.47	982.53	63.24	1077.65	1.10	Terrestrial	Grassy slope	Non-endemic
Cypripedium flavum	Zhongdian	3100	6.84	641.73	69.05	914.89	1.43	Terrestrial	Shrubland	Non-endemic
Cypripedium flavum	Lijiang	3200	7.87	982.53	63.24	1077.65	1.10	Terrestrial	Grassy slope	Non-endemic
Cypripedium flavum	Lijiang	3200	7.87	982.53	63.24	1077.65	1.10	Terrestrial	Meadow	Non-endemic
Cypripedium flavum	Lijiang	3260	7.51	982.53	63.24	1077.65	1.10	Terrestrial	Forest edge	Non-endemic
Cypripedium flavum	Zhongdian	3300	5.64	641.73	69.05	914.89	1.43	Terrestrial	Dense forest	Non-endemic
Cypripedium flavum	Deqin	3300	5.37	639.48	70.85	896.66	1.40	Terrestrial	Dense forest	Non-endemic
Cypripedium flavum	Lijiang	3300	7.27	982.53	63.24	1077.65	1.10	Terrestrial	Forest edge	Non-endemic
Cypripedium flavum	Zhongdian	3350	5.34	641.73	69.05	914.89	1.43	Terrestrial	Grassy slope	Non-endemic
Cypripedium flavum	Zhongdian	3400	5.04	641.73	69.05	914.89	1.43	Terrestrial	Grassy slope	Non-endemic
Cypripedium flavum	Zhongdian	3400	5.04	641.73	69.05	914.89	1.43	Terrestrial	Shrubland	Non-endemic
Cypripedium flavum	Zhongdian	3400	5.04	641.73	69.05	914.89	1.43	Terrestrial	Shrubland	Non-endemic
Cypripedium flavum	Zhongdian	3420	4.92	641.73	69.05	914.89	1.43	Terrestrial	Sparse forest	Non-endemic
Cypripedium flavum	Zhongdian	3450	4.74	641.73	69.05	914.89	1.43	Terrestrial	Dense forest	Non-endemic
Cypripedium flavum	Zhongdian	3500	4.44	641.73	69.05	914.89	1.43	Terrestrial	Shrubland	Non-endemic
Cypripedium forrestii	Zhongdian	3512	4.37	641.73	69.05	914.89	1.43	Terrestrial	Shrubland	Endemic
Cypripedium forrestii	Zhongdian	3650	3.54	641.73	69.05	914.89	1.43	Terrestrial	Dense forest	Endemic
Cypripedium franchetii	Zhongdian	3620	3.72	641.73	69.05	914.89	1.43	Terrestrial	Shrubland	Non-endemic
Cypripedium guttatum	Lijiang	2800	10.27	982.53	63.24	1077.65	1.10	Terrestrial	Grassy slope	Non-endemic
Cypripedium guttatum	Lijiang	2900	9.67	982.53	63.24	1077.65	1.10	Terrestrial	Shrubland	Non-endemic
Cypripedium guttatum	Zhongdian	3000	7.44	641.73	69.05	914.89	1.43	Terrestrial	Dense forest	Non-endemic
Cypripedium guttatum	Deqin	3200	5.97	639.48	70.85	896.66	1.40	Terrestrial	Dense forest	Non-endemic
Cypripedium guttatum	Zhongdian	3210	6.18	641.73	69.05	914.89	1.43	Terrestrial	Dense forest	Non-endemic
Cypripedium guttatum	Deqin	3250	5.67	639.48	70.85	896.66	1.40	Terrestrial	Dense forest	Non-endemic
Cypripedium guttatum	Zhongdian	3300	5.64	641.73	69.05	914.89	1.43	Terrestrial	Dense forest	Non-endemic
Cypripedium guttatum	Zhongdian	3500	4.44	641.73	69.05	914.89	1.43	Terrestrial	Dense forest	Non-endemic
Cypripedium guttatum	Lijiang	3500	6.07	982.53	63.24	1077.65	1.10	Terrestrial	Meadow	Non-endemic
Cypripedium guttatum	Zhongdian	3600	3.84	641.73	69.05	914.89	1.43	Terrestrial	Grassy slope	Non-endemic
Cypripedium guttatum	Zhongdian	3700	3.24	641.73	69.05	914.89	1.43	Terrestrial	Forest edge	Non-endemic
Cypripedium guttatum	Zhongdian	3700	3.24	641.73	69.05	914.89	1.43	Terrestrial	Grassy slope	Non-endemic
Cypripedium henryi	Gongshan	1900	12.68	1738.42	78.47	860.43	0.50	Terrestrial	Shrubland	Non-endemic
Cypripedium henryi	Gongshan	2000	12.08	1738.42	78.47	860.43	0.50	Terrestrial	Sparse forest	Non-endemic
Cypripedium henryi	Weixi	2100	12.83	970.70	69.95	1021.03	1.05	Terrestrial	Dense forest	Non-endemic
Cypripedium henryi	Weixi	2300	11.63	970.70	69.95	1021.03	1.05	Terrestrial	Sparse forest	Non-endemic
Cypripedium henryi	Gongshan	2450	9.38	1738.42	78.47	860.43	0.50	Terrestrial	Dense forest	Non-endemic

Cypripedium henryi	Weixi	2500	10.43	970.70	69.95	1021.03	1.05	Terrestrial	Dense forest	Non-endemic
Cypripedium henryi	Gongshan	2800	7.28	1738.42	78.47	860.43	0.50	Terrestrial	Forest edge	Non-endemic
Cypripedium lichiangense	Lijiang	2600	11.47	982.53	63.24	1077.65	1.10	Terrestrial	Shrubland	Endemic
Cypripedium margaritaceum	Lijiang	2600	11.47	982.53	63.24	1077.65	1.10	Terrestrial	Shrubland	Non-endemic
Cypripedium margaritaceum	Qiaojia	2700	7.42	878.15	76.01	1034.97	1.18	Terrestrial	Dense forest	Non-endemic
Cypripedium margaritaceum	Dali	2700	10.62	1082.70	68.61	1256.27	1.16	Terrestrial	Shrubland	Non-endemic
Cypripedium margaritaceum	Zhongdian	2800	8.64	641.73	69.05	914.89	1.43	Terrestrial	Shrubland	Non-endemic
Cypripedium margaritaceum	Lijiang	2800	10.27	982.53	63.24	1077.65	1.10	Terrestrial	Shrubland	Non-endemic
Cypripedium margaritaceum	Lijiang	2850	9.97	982.53	63.24	1077.65	1.10	Terrestrial	Dense forest	Non-endemic
Cypripedium margaritaceum	Lijiang	2900	9.67	982.53	63.24	1077.65	1.10	Terrestrial	Dense forest	Non-endemic
Cypripedium margaritaceum	Lijiang	3000	9.07	982.53	63.24	1077.65	1.10	Terrestrial	Dense forest	Non-endemic
Cypripedium margaritaceum	Qiaojia	3000	5.62	878.15	76.01	1034.97	1.18	Terrestrial	Dense forest	Non-endemic
Cypripedium margaritaceum	Dali	3000	8.82	1082.70	68.61	1256.27	1.16	Terrestrial	Meadow	Non-endemic
Cypripedium margaritaceum	Lijiang	3000	9.07	982.53	63.24	1077.65	1.10	Terrestrial	Shrubland	Non-endemic
Cypripedium margaritaceum	Lijiang	3100	8.47	982.53	63.24	1077.65	1.10	Terrestrial	Dense forest	Non-endemic
Cypripedium margaritaceum	Dali	3100	8.22	1082.70	68.61	1256.27	1.16	Terrestrial	Shrubland	Non-endemic
Cypripedium margaritaceum	Lijiang	3150	8.17	982.53	63.24	1077.65	1.10	Terrestrial	Forest edge	Non-endemic
Cypripedium margaritaceum	Dali	3400	6.42	1082.70	68.61	1256.27	1.16	Terrestrial	Dense forest	Non-endemic
Cypripedium margaritaceum	Zhongdian	3450	4.74	641.73	69.05	914.89	1.43	Terrestrial	Sparse forest	Non-endemic
Cypripedium margaritaceum	Lijiang	3500	6.07	982.53	63.24	1077.65	1.10	Terrestrial	Shrubland	Non-endemic
Cypripedium plectrochilum	Luquan	2100	13.12	965.09	73.60	1160.89	1.20	Terrestrial	Sparse forest	Non-endemic
Cypripedium plectrochilum	Fuming	2200	12.77	871.62	71.93	1209.56	1.39	Terrestrial	Grassy slope	Non-endemic
Cypripedium plectrochilum	Songming	2400	11.19	1010.18	74.14	1134.24	1.12	Terrestrial	Dense forest	Non-endemic
Cypripedium plectrochilum	Eryuan	2500	11.53	745.16	68.27	1210.94	1.63	Terrestrial	Grassy slope	Non-endemic
Cypripedium plectrochilum	Lijiang	2650	11.17	982.53	63.24	1077.65	1.10	Terrestrial	Dense forest	Non-endemic
Cypripedium plectrochilum	Zhongdian	2700	9.24	641.73	69.05	914.89	1.43	Terrestrial	Meadow	Non-endemic
Cypripedium plectrochilum	Lijiang	2800	10.27	982.53	63.24	1077.65	1.10	Terrestrial	Dense forest	Non-endemic
Cypripedium plectrochilum	Zhongdian	2800	8.64	641.73	69.05	914.89	1.43	Terrestrial	Grassy slope	Non-endemic
Cypripedium plectrochilum	Dayao	2850	9.83	810.80	65.01	1427.38	1.76	Terrestrial	Dense forest	Non-endemic
Cypripedium plectrochilum	Zhongdian	2900	8.04	641.73	69.05	914.89	1.43	Terrestrial	Dense forest	Non-endemic
Cypripedium plectrochilum	Lijiang	2920	9.55	982.53	63.24	1077.65	1.10	Terrestrial	Dense forest	Non-endemic
Cypripedium plectrochilum	Zhongdian	3000	7.44	641.73	69.05	914.89	1.43	Terrestrial	Forest edge	Non-endemic
Cypripedium plectrochilum	Ninglang	3100	7.46	928.64	69.17	1136.01	1.22	Terrestrial	Dense forest	Non-endemic
Cypripedium plectrochilum	Zhongdian	3100	6.84	641.73	69.05	914.89	1.43	Terrestrial	Dense forest	Non-endemic
Cypripedium plectrochilum	Zhongdian	3100	6.84	641.73	69.05	914.89	1.43	Terrestrial	Shrubland	Non-endemic
Cypripedium plectrochilum	Lijiang	3170	8.05	982.53	63.24	1077.65	1.10	Terrestrial	Dense forest	Non-endemic

Cypripedium plectrochilum	Zhongdian	3210	6.18	641.73	69.05	914.89	1.43	Terrestrial	Dense forest	Non-endemic
Cypripedium plectrochilum	Lijiang	3250	7.57	982.53	63.24	1077.65	1.10	Terrestrial	Dense forest	Non-endemic
Cypripedium plectrochilum	Zhongdian	3250	5.94	641.73	69.05	914.89	1.43	Terrestrial	Open site	Non-endemic
Cypripedium plectrochilum	Lijiang	3250	7.57	982.53	63.24	1077.65	1.10	Terrestrial	Open site	Non-endemic
Cypripedium plectrochilum	Zhongdian	3400	5.04	641.73	69.05	914.89	1.43	Terrestrial	Dense forest	Non-endemic
Cypripedium plectrochilum	Zhongdian	3500	4.44	641.73	69.05	914.89	1.43	Terrestrial	Dense forest	Non-endemic
Cypripedium plectrochilum	Zhongdian	3600	3.84	641.73	69.05	914.89	1.43	Terrestrial	Dense forest	Non-endemic
Cypripedium tibeticum	Lijiang	2600	11.47	982.53	63.24	1077.65	1.10	Terrestrial	Forest edge	Non-endemic
Cypripedium tibeticum	Lijiang	2650	11.17	982.53	63.24	1077.65	1.10	Terrestrial	Shrubland	Non-endemic
Cypripedium tibeticum	Lijiang	2800	10.27	982.53	63.24	1077.65	1.10	Terrestrial	Shrubland	Non-endemic
Cypripedium tibeticum	Weixi	2840	8.39	970.70	69.95	1021.03	1.05	Terrestrial	Grassy slope	Non-endemic
Cypripedium tibeticum	Lijiang	2850	9.97	982.53	63.24	1077.65	1.10	Terrestrial	Meadow	Non-endemic
Cypripedium tibeticum	Lijiang	2900	9.67	982.53	63.24	1077.65	1.10	Terrestrial	Meadow	Non-endemic
Cypripedium tibeticum	Zhongdian	3000	7.44	641.73	69.05	914.89	1.43	Terrestrial	Dense forest	Non-endemic
Cypripedium tibeticum	Lijiang	3100	8.47	982.53	63.24	1077.65	1.10	Terrestrial	Grassy slope	Non-endemic
Cypripedium tibeticum	Lijiang	3100	8.47	982.53	63.24	1077.65	1.10	Terrestrial	Grassy slope	Non-endemic
Cypripedium tibeticum	Lijiang	3140	8.23	982.53	63.24	1077.65	1.10	Terrestrial	Meadow	Non-endemic
Cypripedium tibeticum	Weixi	3200	6.23	970.70	69.95	1021.03	1.05	Terrestrial	Dense forest	Non-endemic
Cypripedium tibeticum	Weixi	3200	6.23	970.70	69.95	1021.03	1.05	Terrestrial	Grassy slope	Non-endemic
Cypripedium tibeticum	Lijiang	3200	7.87	982.53	63.24	1077.65	1.10	Terrestrial	Meadow	Non-endemic
Cypripedium tibeticum	Lijiang	3200	7.87	982.53	63.24	1077.65	1.10	Terrestrial	Meadow	Non-endemic
Cypripedium tibeticum	Yongde	3240	7.69	1266.25	69.00	1283.63	1.01	Terrestrial	Grassy slope	Non-endemic
Cypripedium tibeticum	Zhongdian	3250	5.94	641.73	69.05	914.89	1.43	Terrestrial	Dense forest	Non-endemic
Cypripedium tibeticum	Lijiang	3250	7.57	982.53	63.24	1077.65	1.10	Terrestrial	Grassy slope	Non-endemic
Cypripedium tibeticum	Lijiang	3250	7.57	982.53	63.24	1077.65	1.10	Terrestrial	Grassy slope	Non-endemic
Cypripedium tibeticum	Zhongdian	3300	5.64	641.73	69.05	914.89	1.43	Terrestrial	Dense forest	Non-endemic
Cypripedium tibeticum	Zhongdian	3400	5.04	641.73	69.05	914.89	1.43	Terrestrial	Dense forest	Non-endemic
Cypripedium tibeticum	Zhongdian	3400	5.04	641.73	69.05	914.89	1.43	Terrestrial	Dense forest	Non-endemic
Cypripedium tibeticum	Yangbi	3400	5.59	1044.23	72.19	1161.15	1.11	Terrestrial	Forest edge	Non-endemic
Cypripedium tibeticum	Zhongdian	3400	5.04	641.73	69.05	914.89	1.43	Terrestrial	Grassy slope	Non-endemic
Cypripedium tibeticum	Zhongdian	3450	4.74	641.73	69.05	914.89	1.43	Terrestrial	Dense forest	Non-endemic
Cypripedium tibeticum	Zhongdian	3450	4.74	641.73	69.05	914.89	1.43	Terrestrial	Dense forest	Non-endemic
Cypripedium tibeticum	Zhongdian	3500	4.44	641.73	69.05	914.89	1.43	Terrestrial	Shrubland	Non-endemic
Cypripedium tibeticum	Gongshan	3600	2.48	1738.42	78.47	860.43	0.50	Terrestrial	Forest edge	Non-endemic
Cypripedium tibeticum	Gongshan	3650	2.18	1738.42	78.47	860.43	0.50	Terrestrial	Dense forest	Non-endemic
Cypripedium tibeticum	Zhongdian	3700	3.24	641.73	69.05	914.89	1.43	Terrestrial	Open site	Non-endemic

Cypripedium tibeticum	Zhongdian	3750	2.94	641.73	69.05	914.89	1.43	Terrestrial	Dense forest	Non-endemic
Cypripedium wardii	Deqin	3250	5.67	639.48	70.85	896.66	1.40	Terrestrial	Dense forest	Non-endemic
Cypripedium yunnanense	Zhongdian	3350	5.34	641.73	69.05	914.89	1.43	Terrestrial	Meadow	Endemic
Dendrobium acinaciforme	Mengla	850	20.10	1514.70	84.25	1146.68	0.76	Epiphyte	Dense forest	Non-endemic
Dendrobium acinaciforme	Jinghong	950	20.02	1161.08	80.46	1256.19	1.08	Epiphyte	Sparse forest	Non-endemic
Dendrobium acinaciforme	Jinghong	1270	18.10	1161.08	80.46	1256.19	1.08	Epiphyte	Dense forest	Non-endemic
Dendrobium acinaciforme	Fugong	1320	16.14	1441.43	79.99	906.18	0.63	Epiphyte	Dense forest	Non-endemic
Dendrobium aduncum	Jinping	380	23.21	2305.17	83.81	1030.46	0.45	Epiphyte	Dense forest	Non-endemic
Dendrobium aduncum	Xichou	830	19.95	1267.54	82.98	985.97	0.78	Epiphyte	Dense forest	Non-endemic
Dendrobium aduncum	Xichou	870	19.71	1267.54	82.98	985.97	0.78	Epiphyte	Dense forest	Non-endemic
Dendrobium aduncum	Xichou	1460	16.17	1267.54	82.98	985.97	0.78	Epiphyte	Dense forest	Non-endemic
Dendrobium aduncum	Yangbi	2200	12.79	1044.23	72.19	1161.15	1.11	Epiphyte	Dense forest	Non-endemic
Dendrobium aphyllum	Funing	200	22.42	1161.58	79.07	1147.17	0.99	Epiphyte	Shrubland	Non-endemic
Dendrobium aphyllum	Lushui	900	20.65	1195.57	70.63	911.65	0.76	Epiphyte	Dense forest	Non-endemic
Dendrobium aphyllum	Jinghong	950	20.02	1161.08	80.46	1256.19	1.08	Epiphyte	Sparse forest	Non-endemic
Dendrobium aphyllum	Zhenkang	1000	18.84	1602.96	81.14	1089.09	0.68	Epiphyte	Dense forest	Non-endemic
Dendrobium aphyllum	Zhenkang	1000	18.84	1602.96	81.14	1089.09	0.68	Epiphyte	Dense forest	Non-endemic
Dendrobium aphyllum	Yongde	1000	21.13	1266.25	69.00	1283.63	1.01	Epiphyte	Dense forest	Non-endemic
Dendrobium aphyllum	Zhenkang	1100	18.24	1602.96	81.14	1089.09	0.68	Epiphyte	Dense forest	Non-endemic
Dendrobium aphyllum	Puer	1200	19.06	1429.78	79.51	1123.79	0.79	Epiphyte	Dense forest	Non-endemic
Dendrobium aphyllum	Menghai	1350	17.46	1314.38	80.83	1150.91	0.88	Epiphyte	Sparse forest	Non-endemic
Dendrobium aphyllum	Zhenkang	1500	15.84	1602.96	81.14	1089.09	0.68	Epiphyte	Dense forest	Non-endemic
Dendrobium aphyllum	Zhenkang	1600	15.24	1602.96	81.14	1089.09	0.68	Epiphyte	Dense forest	Non-endemic
Dendrobium aphyllum	Zhenkang	1700	14.64	1602.96	81.14	1089.09	0.68	Epiphyte	Dense forest	Non-endemic
Dendrobium aphyllum	Longling	1800	13.32	2098.66	84.65	973.45	0.46	Epiphyte	Dense forest	Non-endemic
Dendrobium aurantiacum var.auranticum	Gongshan	1500	15.08	1738.42	78.47	860.43	0.50	Epiphyte	Dense forest	Non-endemic
Dendrobium aurantiacum var.denneanum	Xichou	1320	17.01	1267.54	82.98	985.97	0.78	Epiphyte	Dense forest	Non-endemic
Dendrobium aurantiacum var.denneanum	Xichou	1460	16.17	1267.54	82.98	985.97	0.78	Epiphyte	Dense forest	Non-endemic
Dendrobium aurantiacum var.denneanum	Menghai	1540	16.32	1314.38	80.83	1150.91	0.88	Epiphyte	Shrubland	Non-endemic
Dendrobium aurantiacum var.denneanum	Xichou	1600	15.33	1267.54	82.98	985.97	0.78	Epiphyte	Dense forest	Non-endemic

Dendrobium aurantiacum var.denneanum	Gongshan	1600	14.48	1738.42	78.47	860.43	0.50	Epiphyte	Forest edge	Non-endemic
Dendrobium aurantiacum var.denneanum	Xichou	1600	15.33	1267.54	82.98	985.97	0.78	Epiphyte	Sparse forest	Non-endemic
Dendrobium aurantiacum var.denneanum	Weixi	1800	14.63	970.70	69.95	1021.03	1.05	Epiphyte	Dense forest	Non-endemic
Dendrobium aurantiacum var.denneanum	Fengqing	1900	14.80	1352.80	73.24	1172.52	0.87	Epiphyte	Dense forest	Non-endemic
Dendrobium aurantiacum var.denneanum	Weixi	2000	13.43	970.70	69.95	1021.03	1.05	Epiphyte	Dense forest	Non-endemic
Dendrobium aurantiacum var.denneanum	Weixi	2015	13.34	970.70	69.95	1021.03	1.05	Epiphyte	Dense forest	Non-endemic
Dendrobium aurantiacum var.denneanum	Gongshan	2100	11.48	1738.42	78.47	860.43	0.50	Epiphyte	Dense forest	Non-endemic
Dendrobium aurantiacum var.denneanum	Dali	2200	13.62	1082.70	68.61	1256.27	1.16	Epiphyte	Shrubland	Non-endemic
Dendrobium aurantiacum var.denneanum	Lijiang	2400	12.67	982.53	63.24	1077.65	1.10	Epiphyte	Dense forest	Non-endemic
Dendrobium aurantiacum var.denneanum	Lijiang	2500	12.07	982.53	63.24	1077.65	1.10	Epiphyte	Dense forest	Non-endemic
Dendrobium aurantiacum var.denneanum	Zhongdian	3250	5.94	641.73	69.05	914.89	1.43	Epiphyte	Dense forest	Non-endemic
Dendrobium bellatulum	Pingbian	1250	17.32	1648.57	86.34	990.16	0.60	Epiphyte	Dense forest	Non-endemic
Dendrobium bellatulum	Zhenkang	1600	15.24	1602.96	81.14	1089.09	0.68	Epiphyte	Dense forest	Non-endemic
Dendrobium bellatulum	Yongde	1600	17.53	1266.25	69.00	1283.63	1.01	Epiphyte	Dense forest	Non-endemic
Dendrobium bellatulum	Lancang	1800	14.90	1596.50	77.88	1183.80	0.74	Epiphyte	Dense forest	Non-endemic
Dendrobium bellatulum	Jingdong	1950	13.88	1128.40	76.80	1142.11	1.01	Epiphyte	Dense forest	Non-endemic
Dendrobium bellatulum	Jingdong	2050	13.28	1128.40	76.80	1142.11	1.01	Epiphyte	Dense forest	Non-endemic
Dendrobium bellatulum	Fengqing	2100	13.60	1352.80	73.24	1172.52	0.87	Epiphyte	Dense forest	Non-endemic
Dendrobium bellatulum	Jingdong	2100	12.98	1128.40	76.80	1142.11	1.01	Epiphyte	Dense forest	Non-endemic
Dendrobium bellatulum	Jingdong	2100	12.98	1128.40	76.80	1142.11	1.01	Epiphyte	Dense forest	Non-endemic
Dendrobium brymerianum	Yongde	1600	17.53	1266.25	69.00	1283.63	1.01	Epiphyte	Dense forest	Non-endemic
Dendrobium brymerianum	Menghai	1900	14.16	1314.38	80.83	1150.91	0.88	Epiphyte	Dense forest	Non-endemic
Dendrobium capillipes	Jinghong	900	20.32	1161.08	80.46	1256.19	1.08	Epiphyte	Dense forest	Non-endemic
Dendrobium cariniferum	Zhenkang	1100	18.24	1602.96	81.14	1089.09	0.68	Epiphyte	Dense forest	Non-endemic
Dendrobium cariniferum	Cangyuan	1200	18.02	1733.34	81.63	1060.82	0.61	Epiphyte	Dense forest	Non-endemic

Dendrobium cariniferum	Zhenkang	1640	15.00	1602.96	81.14	1089.09	0.68	Epiphyte	Dense forest	Non-endemic
Dendrobium chrysanthum	Mengla	700	21.00	1514.70	84.25	1146.68	0.76	Epiphyte	Dense forest	Non-endemic
Dendrobium chrysanthum	Malipo	1000	18.26	1063.54	85.83	1053.97	0.99	Epiphyte	Sparse forest	Non-endemic
Dendrobium chrysanthum	Yanshan	1100	18.86	1003.57	79.42	1172.21	1.17	Epiphyte	Dense forest	Non-endemic
Dendrobium chrysanthum	Fugong	1200	16.86	1441.43	79.99	906.18	0.63	Epiphyte	Forest edge	Non-endemic
Dendrobium chrysanthum	Pingbian	1250	17.32	1648.57	86.34	990.16	0.60	Epiphyte	Dense forest	Non-endemic
Dendrobium chrysanthum	Lincang	1300	18.72	1165.84	72.08	1167.73	1.00	Epiphyte	Dense forest	Non-endemic
Dendrobium chrysanthum	Jingdong	1450	16.88	1128.40	76.80	1142.11	1.01	Epiphyte	Dense forest	Non-endemic
Dendrobium chrysanthum	Xichou	1500	15.93	1267.54	82.98	985.97	0.78	Epiphyte	Dense forest	Non-endemic
Dendrobium chrysanthum	Luchun	1580	17.22	2013.54	78.64	1151.56	0.57	Epiphyte	Dense forest	Non-endemic
Dendrobium chrysanthum	Fugong	1700	13.86	1441.43	79.99	906.18	0.63	Epiphyte	Sparse forest	Non-endemic
Dendrobium chrysanthum	Lancang	1900	14.30	1596.50	77.88	1183.80	0.74	Epiphyte	Dense forest	Non-endemic
Dendrobium chrysanthum	Fugong	2000	12.06	1441.43	79.99	906.18	0.63	Epiphyte	Dense forest	Non-endemic
Dendrobium chrysanthum	Fugong	2500	9.06	1441.43	79.99	906.18	0.63	Epiphyte	Dense forest	Non-endemic
Dendrobium chrysanthum	Fugong	2500	9.06	1441.43	79.99	906.18	0.63	Epiphyte	Dense forest	Non-endemic
Dendrobium chrysotoxum	Jinghong	700	21.52	1161.08	80.46	1256.19	1.08	Epiphyte	Sparse forest	Non-endemic
Dendrobium chrysotoxum	Jinghong	1400	17.32	1161.08	80.46	1256.19	1.08	Epiphyte	Dense forest	Non-endemic
Dendrobium chrysotoxum	Cangyuan	1500	16.22	1733.34	81.63	1060.82	0.61	Epiphyte	Dense forest	Non-endemic
Dendrobium chrysotoxum	Lancang	1550	16.40	1596.50	77.88	1183.80	0.74	Epiphyte	Dense forest	Non-endemic
Dendrobium compactum	Jinghong	800	20.92	1161.08	80.46	1256.19	1.08	Epiphyte	Shrubland	Non-endemic
Dendrobium compactum	Menghai	1100	18.96	1314.38	80.83	1150.91	0.88	Epiphyte	Dense forest	Non-endemic
Dendrobium compactum	Mengla	1150	18.30	1514.70	84.25	1146.68	0.76	Epiphyte	Dense forest	Non-endemic
Dendrobium compactum	Menghai	1530	16.38	1314.38	80.83	1150.91	0.88	Epiphyte	Dense forest	Non-endemic
Dendrobium compactum	Jinghong	1600	16.12	1161.08	80.46	1256.19	1.08	Epiphyte	Shrubland	Non-endemic
Dendrobium compactum	Jinghong	1850	14.62	1161.08	80.46	1256.19	1.08	Epiphyte	Dense forest	Non-endemic
Dendrobium compactum	Fengqing	2000	14.20	1352.80	73.24	1172.52	0.87	Epiphyte	Dense forest	Non-endemic
Dendrobium compressicaule	Mengla	720	20.88	1514.70	84.25	1146.68	0.76	Epiphyte	Dense forest	Non-endemic
Dendrobium crepidatum	Zhenkang	1200	17.64	1602.96	81.14	1089.09	0.68	Epiphyte	Dense forest	Non-endemic
Dendrobium crystallinum	Mengla	810	20.34	1514.70	84.25	1146.68	0.76	Epiphyte	Dense forest	Non-endemic
Dendrobium devonianum	Jinghong	750	21.22	1161.08	80.46	1256.19	1.08	Epiphyte	Sparse forest	Non-endemic
Dendrobium devonianum	Jinghong	950	20.02	1161.08	80.46	1256.19	1.08	Epiphyte	Sparse forest	Non-endemic
Dendrobium devonianum	Pingbian	1000	18.82	1648.57	86.34	990.16	0.60	Epiphyte	Sparse forest	Non-endemic
Dendrobium devonianum	Ruili	1200	17.80	1436.05	78.54	1237.71	0.86	Epiphyte	Dense forest	Non-endemic
Dendrobium devonianum	Jinghong	1200	18.52	1161.08	80.46	1256.19	1.08	Epiphyte	Dense forest	Non-endemic
Dendrobium devonianum	Hekou	1270	16.13	1768.58	84.25	1166.26	0.66	Epiphyte	Sparse forest	Non-endemic
Dendrobium devonianum	Pingbian	1300	17.02	1648.57	86.34	990.16	0.60	Epiphyte	Dense forest	Non-endemic

Dendrobium devonianum	Lancang	1400	17.30	1596.50	77.88	1183.80	0.74	Epiphyte	Dense forest	Non-endemic
Dendrobium devonianum	Menghai	1530	16.38	1314.38	80.83	1150.91	0.88	Epiphyte	Shrubland	Non-endemic
Dendrobium devonianum	Jingdong	1670	15.56	1128.40	76.80	1142.11	1.01	Epiphyte	Dense forest	Non-endemic
Dendrobium devonianum	Menghai	1800	14.76	1314.38	80.83	1150.91	0.88	Epiphyte	Dense forest	Non-endemic
Dendrobium devonianum	Fengqing	2000	14.20	1352.80	73.24	1172.52	0.87	Epiphyte	Dense forest	Non-endemic
Dendrobium dixanthum	Jinghong	1200	18.52	1161.08	80.46	1256.19	1.08	Epiphyte	Dense forest	Non-endemic
Dendrobium ellipsophyllum	Menghai	1100	18.96	1314.38	80.83	1150.91	0.88	Epiphyte	Dense forest	Non-endemic
Dendrobium exile	Mengla	500	22.20	1514.70	84.25	1146.68	0.76	Epiphyte	Dense forest	Non-endemic
Dendrobium exile	Mengla	650	21.30	1514.70	84.25	1146.68	0.76	Epiphyte	Dense forest	Non-endemic
Dendrobium exile	Mengla	710	20.94	1514.70	84.25	1146.68	0.76	Epiphyte	Dense forest	Non-endemic
Dendrobium falconeri	Jingdong	1540	16.34	1128.40	76.80	1142.11	1.01	Epiphyte	Dense forest	Non-endemic
Dendrobium falconeri	Zhenkang	1600	15.24	1602.96	81.14	1089.09	0.68	Epiphyte	Dense forest	Non-endemic
Dendrobium falconeri	Jingdong	1600	15.98	1128.40	76.80	1142.11	1.01	Epiphyte	Forest edge	Non-endemic
Dendrobium falconeri	Luchun	1620	16.98	2013.54	78.64	1151.56	0.57	Epiphyte	Dense forest	Non-endemic
Dendrobium fimbriatum	Jinghong	650	21.82	1161.08	80.46	1256.19	1.08	Epiphyte	Sparse forest	Non-endemic
Dendrobium fimbriatum	Xichou	870	19.71	1267.54	82.98	985.97	0.78	Epiphyte	Dense forest	Non-endemic
Dendrobium fimbriatum	Hekou	900	18.35	1768.58	84.25	1166.26	0.66	Epiphyte	Forest edge	Non-endemic
Dendrobium fimbriatum	Funing	1000	17.62	1161.58	79.07	1147.17	0.99	Epiphyte	Sparse forest	Non-endemic
Dendrobium fimbriatum	Fugong	1050	17.76	1441.43	79.99	906.18	0.63	Epiphyte	Forest edge	Non-endemic
Dendrobium fimbriatum	Jinghong	1150	18.82	1161.08	80.46	1256.19	1.08	Epiphyte	Dense forest	Non-endemic
Dendrobium fimbriatum	Yanshan	1200	18.26	1003.57	79.42	1172.21	1.17	Epiphyte	Dense forest	Non-endemic
Dendrobium fimbriatum	Zhenkang	1400	16.44	1602.96	81.14	1089.09	0.68	Epiphyte	Dense forest	Non-endemic
Dendrobium fimbriatum	Xichou	1500	15.93	1267.54	82.98	985.97	0.78	Epiphyte	Open site	Non-endemic
Dendrobium fimbriatum	Gongshan	1550	14.78	1738.42	78.47	860.43	0.50	Epiphyte	Dense forest	Non-endemic
Dendrobium fimbriatum	Zhenkang	1600	15.24	1602.96	81.14	1089.09	0.68	Epiphyte	Dense forest	Non-endemic
Dendrobium fimbriatum	Xichou	1600	15.33	1267.54	82.98	985.97	0.78	Epiphyte	Dense forest	Non-endemic
Dendrobium fimbriatum	Zhenkang	1640	15.00	1602.96	81.14	1089.09	0.68	Epiphyte	Dense forest	Non-endemic
Dendrobium fimbriatum	Fugong	3100	5.46	1441.43	79.99	906.18	0.63	Epiphyte	Dense forest	Non-endemic
Dendrobium findlayanum	Mengla	1000	19.20	1514.70	84.25	1146.68	0.76	Epiphyte	Dense forest	Non-endemic
Dendrobium guangxiense	Xichou	1530	15.75	1267.54	82.98	985.97	0.78	Epiphyte	Sparse forest	Non-endemic
Dendrobium hancockii	Funing	700	19.42	1161.58	79.07	1147.17	0.99	Epiphyte	Dense forest	Non-endemic
Dendrobium harveyanum	Yongde	1600	17.53	1266.25	69.00	1283.63	1.01	Epiphyte	Dense forest	Non-endemic
Dendrobium henryi	Malipo	550	20.96	1063.54	85.83	1053.97	0.99	Epiphyte	Shrubland	Non-endemic
Dendrobium henryi	Menglian	910	20.12	1357.68	80.47	1185.94	0.87	Epiphyte	Sparse forest	Non-endemic
Dendrobium henryi	Pingbian	1100	18.22	1648.57	86.34	990.16	0.60	Epiphyte	Dense forest	Non-endemic
Dendrobium henryi	Pingbian	1250	17.32	1648.57	86.34	990.16	0.60	Epiphyte	Sparse forest	Non-endemic

Dendrobium henryi	Pingbian	1360	16.66	1648.57	86.34	990.16	0.60	Epiphyte	Dense forest	Non-endemic
Dendrobium henryi	Hekou	1460	14.99	1768.58	84.25	1166.26	0.66	Epiphyte	Dense forest	Non-endemic
Dendrobium henryi	Xichou	1500	15.93	1267.54	82.98	985.97	0.78	epiphyte	Forest edge	Non-endemic
Dendrobium henryi	Menglian	1700	15.38	1357.68	80.47	1185.94	0.87	Epiphyte	Dense forest	Non-endemic
Dendrobium hercoglossum	Maguan	800	20.21	1330.52	83.25	1086.43	0.82	Epiphyte	Dense forest	Non-endemic
Dendrobium hercoglossum	Pingbian	1200	17.62	1648.57	86.34	990.16	0.60	Epiphyte	Dense forest	Non-endemic
Dendrobium hercoglossum	Jinping	1260	17.93	2305.17	83.81	1030.46	0.45	Epiphyte	Sparse forest	Non-endemic
Dendrobium heterocarpum	Zhenkang	1600	15.24	1602.96	81.14	1089.09	0.68	Epiphyte	Sparse forest	Non-endemic
Dendrobium heterocarpum	Zhenkang	1600	15.24	1602.96	81.14	1089.09	0.68	Epiphyte	Dense forest	Non-endemic
Dendrobium heterocarpum	Luxi	1750	14.58	1650.33	79.33	1183.39	0.72	Epiphyte	Dense forest	Non-endemic
Dendrobium hookerianum	Fugong	1100	17.46	1441.43	79.99	906.18	0.63	Epiphyte	Dense forest	Non-endemic
Dendrobium hookerianum	Gongshan	1300	16.28	1738.42	78.47	860.43	0.50	Epiphyte	Dense forest	Non-endemic
Dendrobium hookerianum	Gongshan	1400	15.68	1738.42	78.47	860.43	0.50	Epiphyte	Dense forest	Non-endemic
Dendrobium hookerianum	Gongshan	1570	14.66	1738.42	78.47	860.43	0.50	Epiphyte	Forest edge	Non-endemic
Dendrobium hookerianum	Gongshan	1600	14.48	1738.42	78.47	860.43	0.50	Epiphyte	Forest edge	Non-endemic
Dendrobium hookerianum	Gongshan	1600	14.48	1738.42	78.47	860.43	0.50	Epiphyte	Forest edge	Non-endemic
Dendrobium hookerianum	Gongshan	1700	13.88	1738.42	78.47	860.43	0.50	Epiphyte	Dense forest	Non-endemic
Dendrobium hookerianum	Gongshan	1800	13.28	1738.42	78.47	860.43	0.50	Epiphyte	Dense forest	Non-endemic
Dendrobium hookerianum	Gongshan	1800	13.28	1738.42	78.47	860.43	0.50	Epiphyte	Dense forest	Non-endemic
Dendrobium hookerianum	Lushui	1900	14.65	1195.57	70.63	911.65	0.76	Epiphyte	Dense forest	Non-endemic
Dendrobium hookerianum	Fugong	3300	4.26	1441.43	79.99	906.18	0.63	Epiphyte	Dense forest	Non-endemic
Dendrobium infundibulum	Mengla	2070	12.78	1514.70	84.25	1146.68	0.76	Epiphyte	Dense forest	Non-endemic
Dendrobium jenkinsii	Jinghong	1150	18.82	1161.08	80.46	1256.19	1.08	Epiphyte	Dense forest	Non-endemic
Dendrobium jenkinsii	Menghai	1180	18.48	1314.38	80.83	1150.91	0.88	Epiphyte	Dense forest	Non-endemic
Dendrobium jenkinsii	Jinghong	1400	17.32	1161.08	80.46	1256.19	1.08	Epiphyte	Shrubland	Non-endemic
Dendrobium jenkinsii	Jinghong	1400	17.32	1161.08	80.46	1256.19	1.08	Epiphyte	Sparse forest	Non-endemic
Dendrobium jenkinsii	Menghai	1500	16.56	1314.38	80.83	1150.91	0.88	Epiphyte	Dense forest	Non-endemic
Dendrobium jenkinsii	Lancang	1500	16.70	1596.50	77.88	1183.80	0.74	Epiphyte	Dense forest	Non-endemic
Dendrobium jenkinsii	Menghai	1530	16.38	1314.38	80.83	1150.91	0.88	Epiphyte	Dense forest	Non-endemic
Dendrobium jenkinsii	Cangyuan	1550	15.92	1733.34	81.63	1060.82	0.61	Epiphyte	Dense forest	Non-endemic
Dendrobium lindleyi	Hekou	270	22.13	1768.58	84.25	1166.26	0.66	Epiphyte	Dense forest	Non-endemic
Dendrobium lindleyi	Xichou	500	21.93	1267.54	82.98	985.97	0.78	Epiphyte	Dense forest	Non-endemic
Dendrobium lindleyi	Menghai	1400	17.16	1314.38	80.83	1150.91	0.88	Epiphyte	Dense forest	Non-endemic
Dendrobium lituiflorum	Jinghong	540	22.48	1161.08	80.46	1256.19	1.08	Epiphyte	Dense forest	Non-endemic
Dendrobium lituiflorum	Lushui	900	20.65	1195.57	70.63	911.65	0.76	Epiphyte	Dense forest	Non-endemic
Dendrobium lituiflorum	Jinghong	1100	19.12	1161.08	80.46	1256.19	1.08	Epiphyte	Dense forest	Non-endemic

Dendrobium lituiflorum	Zhenkang	1600	15.24	1602.96	81.14	1089.09	0.68	Epiphyte	Dense forest	Non-endemic
Dendrobium lituiflorum	Lancang	1700	15.50	1596.50	77.88	1183.80	0.74	Epiphyte	Dense forest	Non-endemic
Dendrobium loddigesii	Funing	700	19.42	1161.58	79.07	1147.17	0.99	Epiphyte	Dense forest	Non-endemic
Dendrobium loddigesii	Guangnan	1340	16.21	1044.26	78.94	1090.35	1.04	Epiphyte	Dense forest	Non-endemic
Dendrobium loddigesii	Wenshan	1500	16.66	988.87	76.70	1272.98	1.29	Epiphyte	Dense forest	Non-endemic
Dendrobium lohohense	Xichou	1100	18.33	1267.54	82.98	985.97	0.78	Epiphyte	Sparse forest	Non-endemic
Dendrobium lohohense	Xichou	1350	16.83	1267.54	82.98	985.97	0.78	Epiphyte	Sparse forest	Non-endemic
Dendrobium lohohense	Yongde	2700	10.93	1266.25	69.00	1283.63	1.01	Epiphyte	Dense forest	Non-endemic
Dendrobium longicornu	Gongshan	1200	16.88	1738.42	78.47	860.43	0.50	Epiphyte	Dense forest	Non-endemic
Dendrobium longicornu	Pingbian	1400	16.42	1648.57	86.34	990.16	0.60	Epiphyte	Grassy slope	Non-endemic
Dendrobium longicornu	Xichou	1550	15.63	1267.54	82.98	985.97	0.78	Epiphyte	Dense forest	Non-endemic
Dendrobium longicornu	Luchun	1900	15.30	2013.54	78.64	1151.56	0.57	Epiphyte	Dense forest	Non-endemic
Dendrobium longicornu	Tengchong	2100	12.40	1501.45	78.06	857.93	0.57	Epiphyte	Forest edge	Non-endemic
Dendrobium longicornu	Yongde	2340	13.09	1266.25	69.00	1283.63	1.01	Epiphyte	Dense forest	Non-endemic
Dendrobium longicornu	Longling	2400	9.72	2098.66	84.65	973.45	0.46	Epiphyte	Dense forest	Non-endemic
Dendrobium longicornu	Yongde	2400	12.73	1266.25	69.00	1283.63	1.01	Epiphyte	Dense forest	Non-endemic
Dendrobium longicornu	Luchun	2500	11.70	2013.54	78.64	1151.56	0.57	Epiphyte	Dense forest	Non-endemic
Dendrobium longicornu	Yongde	2560	11.77	1266.25	69.00	1283.63	1.01	Epiphyte	Dense forest	Non-endemic
Dendrobium longicornu	Yongde	2600	11.53	1266.25	69.00	1283.63	1.01	Epiphyte	Dense forest	Non-endemic
Dendrobium longicornu	Yunlong	2800	9.25	1195.57	70.63	911.65	0.76	Epiphyte	Dense forest	Non-endemic
Dendrobium minutiflorum	Hekou	1840	12.71	1768.58	84.25	1166.26	0.66	Epiphyte	Dense forest	Non-endemic
Dendrobium moniliforme	Xichou	1620	15.21	1267.54	82.98	985.97	0.78	Epiphyte	Dense forest	Non-endemic
Dendrobium moniliforme	Gengma	1700	15.47	1327.70	77.28	1181.78	0.89	Epiphyte	Dense forest	Non-endemic
Dendrobium moniliforme	Pingbian	1800	14.02	1648.57	86.34	990.16	0.60	Epiphyte	Dense forest	Non-endemic
Dendrobium moniliforme	Wenshan	1900	14.26	988.87	76.70	1272.98	1.29	Epiphyte	Dense forest	Non-endemic
Dendrobium moniliforme	Pingbian	2000	12.82	1648.57	86.34	990.16	0.60	Epiphyte	Dense forest	Non-endemic
Dendrobium moniliforme	Jingdong	2000	13.58	1128.40	76.80	1142.11	1.01	Epiphyte	Dense forest	Non-endemic
Dendrobium moniliforme	Yangbi	2510	10.93	1044.23	72.19	1161.15	1.11	Epiphyte	Dense forest	Non-endemic
Dendrobium moniliforme	Gongshan	3000	6.08	1738.42	78.47	860.43	0.50	Epiphyte	Dense forest	Non-endemic
Dendrobium moschatum	Jinghong	1300	17.92	1161.08	80.46	1256.19	1.08	Epiphyte	Dense forest	Non-endemic
Dendrobium nobile	Xichou	850	19.83	1267.54	82.98	985.97	0.78	Epiphyte	Open site	Non-endemic
Dendrobium nobile	Jinghong	1000	19.72	1161.08	80.46	1256.19	1.08	Epiphyte	Dense forest	Non-endemic
Dendrobium nobile	Fugong	1050	17.76	1441.43	79.99	906.18	0.63	Epiphyte	Forest edge	Non-endemic
Dendrobium nobile	Fugong	1100	17.46	1441.43	79.99	906.18	0.63	Epiphyte	Sparse forest	Non-endemic
Dendrobium nobile	Menghai	1300	17.76	1314.38	80.83	1150.91	0.88	Epiphyte	Sparse forest	Non-endemic
Dendrobium nobile	Xichou	1350	16.83	1267.54	82.98	985.97	0.78	Epiphyte	Sparse forest	Non-endemic

Dendrobium nobile	Cangyuan	1500	16.22	1733.34	81.63	1060.82	0.61	Epiphyte	Dense forest	Non-endemic
Dendrobium nobile	Wenshan	1500	16.66	988.87	76.70	1272.98	1.29	Epiphyte	Sparse forest	Non-endemic
Dendrobium nobile	Gongshan	1600	14.48	1738.42	78.47	860.43	0.50	Epiphyte	Sparse forest	Non-endemic
Dendrobium nobile	Lijiang	1700	16.87	982.53	63.24	1077.65	1.10	Epiphyte	Forest edge	Non-endemic
Dendrobium officinale	Gongshan	2200	10.88	1738.42	78.47	860.43	0.50	Epiphyte	Forest edge	Non-endemic
Dendrobium officinale	Gongshan	2350	9.98	1738.42	78.47	860.43	0.50	Epiphyte	Dense forest	Non-endemic
Dendrobium parciflorum	Jinghong	1500	16.72	1161.08	80.46	1256.19	1.08	Epiphyte	Dense forest	Non-endemic
Dendrobium pendulum	Mengla	1200	18.00	1514.70	84.25	1146.68	0.76	Epiphyte	Dense forest	Non-endemic
Dendrobium porphyrochilum	Tengchong	2700	8.80	1501.45	78.06	857.93	0.57	Epiphyte	Dense forest	Non-endemic
Dendrobium primulinum	Longling	1800	13.32	2098.66	84.65	973.45	0.46	Epiphyte	Dense forest	Non-endemic
Dendrobium salaccense	Mengla	680	21.12	1514.70	84.25	1146.68	0.76	Epiphyte	Dense forest	Non-endemic
Dendrobium sinense	Mengla	520	22.08	1514.70	84.25	1146.68	0.76	Epiphyte	Dense forest	Non-endemic
Dendrobium spatella	Mengla	830	20.22	1514.70	84.25	1146.68	0.76	Epiphyte	Dense forest	Non-endemic
Dendrobium strongylanthum	Longchuan	1000	18.55	1604.50	80.46	1149.57	0.72	Epiphyte	Shrubland	Non-endemic
Dendrobium strongylanthum	Hekou	1300	15.95	1768.58	84.25	1166.26	0.66	Epiphyte	Dense forest	Non-endemic
Dendrobium strongylanthum	Jinghong	1800	14.92	1161.08	80.46	1256.19	1.08	Epiphyte	Dense forest	Non-endemic
Dendrobium strongylanthum	Luchun	1900	15.30	2013.54	78.64	1151.56	0.57	Epiphyte	Dense forest	Non-endemic
Dendrobium strongylanthum	Shuangjiang	2000	13.98	1006.64	75.26	1272.13	1.26	Epiphyte	Dense forest	Non-endemic
Dendrobium strongylanthum	Jingdong	2100	12.98	1128.40	76.80	1142.11	1.01	Epiphyte	Dense forest	Non-endemic
Dendrobium strongylanthum	Jingdong	2100	12.98	1128.40	76.80	1142.11	1.01	Epiphyte	Dense forest	Non-endemic
Dendrobium strongylanthum	Jingdong	2100	12.98	1128.40	76.80	1142.11	1.01	Epiphyte	Dense forest	Non-endemic
Dendrobium strongylanthum	Jingdong	2100	12.98	1128.40	76.80	1142.11	1.01	Epiphyte	Dense forest	Non-endemic
Dendrobium strongylanthum	Luchun	2100	14.10	2013.54	78.64	1151.56	0.57	Epiphyte	Sparse forest	Non-endemic
Dendrobium strongylanthum	Tengchong	2300	11.20	1501.45	78.06	857.93	0.57	Epiphyte	Dense forest	Non-endemic
Dendrobium stuposum	Menghai	1760	15.00	1314.38	80.83	1150.91	0.88	Epiphyte	Dense forest	Non-endemic
Dendrobium sulcatum	Mengla	950	19.50	1514.70	84.25	1146.68	0.76	Epiphyte	Dense forest	Non-endemic
Dendrobium terminale	Mengla	1080	18.72	1514.70	84.25	1146.68	0.76	Epiphyte	Dense forest	Non-endemic
Dendrobium thyrsiflorum	Cangyuan	800	20.42	1733.34	81.63	1060.82	0.61	Epiphyte	Dense forest	Non-endemic
Dendrobium thyrsiflorum	Maguan	800	20.21	1330.52	83.25	1086.43	0.82	Epiphyte	Dense forest	Non-endemic
Dendrobium thyrsiflorum	Malipo	900	18.86	1063.54	85.83	1053.97	0.99	Epiphyte	Shrubland	Non-endemic
Dendrobium thyrsiflorum	Jinghong	1000	19.72	1161.08	80.46	1256.19	1.08	Epiphyte	Dense forest	Non-endemic
Dendrobium thyrsiflorum	Jinghong	1100	19.12	1161.08	80.46	1256.19	1.08	Epiphyte	Dense forest	Non-endemic
Dendrobium thyrsiflorum	Jinghong	1200	18.52	1161.08	80.46	1256.19	1.08	Epiphyte	Dense forest	Non-endemic
Dendrobium thyrsiflorum	Pingbian	1200	17.62	1648.57	86.34	990.16	0.60	Epiphyte	Dense forest	Non-endemic
Dendrobium thyrsiflorum	Jinghong	1400	17.32	1161.08	80.46	1256.19	1.08	Epiphyte	Dense forest	Non-endemic
Dendrobium thyrsiflorum	Cangyuan	1500	16.22	1733.34	81.63	1060.82	0.61	Epiphyte	Dense forest	Non-endemic

Dendrobium thyrsiflorum	Cangyuan	1500	16.22	1733.34	81.63	1060.82	0.61	Epiphyte	Dense forest	Non-endemic
Dendrobium thyrsiflorum	Menghai	1550	16.26	1314.38	80.83	1150.91	0.88	Epiphyte	Dense forest	Non-endemic
Dendrobium thyrsiflorum	Gengma	1620	15.47	1327.70	77.28	1181.78	0.89	Epiphyte	Dense forest	Non-endemic
Dendrobium thyrsiflorum	Menghai	1800	14.76	1314.38	80.83	1150.91	0.88	Epiphyte	Dense forest	Non-endemic
Dendrobium thyrsiflorum	Jingdong	2000	13.58	1128.40	76.80	1142.11	1.01	Epiphyte	Dense forest	Non-endemic
Dendrobium thyrsiflorum	Jingdong	2400	11.18	1128.40	76.80	1142.11	1.01	Epiphyte	Dense forest	Non-endemic
Dendrobium thyrsiflorum	Jingdong	2400	11.18	1128.40	76.80	1142.11	1.01	Epiphyte	Dense forest	Non-endemic
Dendrobium thyrsiflorum	Jinghong	900	20.32	1161.08	80.46	1256.19	1.08	Epiphyte	Dense forest	Non-endemic
Dendrobium trigonopus	Menghai	1150	18.66	1314.38	80.83	1150.91	0.88	Epiphyte	Dense forest	Non-endemic
Dendrobium wardianum	Luchun	1500	17.70	2013.54	78.64	1151.56	0.57	Epiphyte	Dense forest	Non-endemic
Dendrobium wardianum	Jingdong	1800	14.78	1128.40	76.80	1142.11	1.01	Epiphyte	Dense forest	Non-endemic
Dendrobium wardianum	Jingdong	1900	14.18	1128.40	76.80	1142.11	1.01	Epiphyte	Dense forest	Non-endemic
Dendrobium wardianum	Jingdong	2200	12.38	1128.40	76.80	1142.11	1.01	Epiphyte	Sparse forest	Non-endemic
Dendrobium williamsonii	Yongde	2400	12.73	1266.25	69.00	1283.63	1.01	Epiphyte	Shrubland	Non-endemic
Dendrobium wilsonii	Xichou	1620	15.21	1267.54	82.98	985.97	0.78	Epiphyte	Dense forest	Non-endemic
Diglyphosa latifolia	Pingbian	1200	17.62	1648.57	86.34	990.16	0.60	Terrestrial	Dense forest	Non-endemic
Diphylax contigua	Gongshan	3000	6.08	1738.42	78.47	860.43	0.50	Terrestrial	Shrubland	Endemic
Diphylax uniformis	Yongde	2500	12.13	1266.25	69.00	1283.63	1.01	Terrestrial	Forest edge	Non-endemic
Diphylax uniformis	Zhongdian	3930	1.86	641.73	69.05	914.89	1.43	Terrestrial	Dense forest	Non-endemic
Diphylax urceolata	Yiliang	1750	11.78	751.45	71.67	1097.64	1.46	Terrestrial	Dense forest	Non-endemic
Diphylax urceolata	Gongshan	3100	5.48	1738.42	78.47	860.43	0.50	Terrestrial	Grassy slope	Non-endemic
Diplomeris pulchella	Gongshan	1350	15.98	1738.42	78.47	860.43	0.50	Terrestrial	Dense forest	Non-endemic
Diplomeris pulchella	Gongshan	1420	15.56	1738.42	78.47	860.43	0.50	Terrestrial	Dense forest	Non-endemic
Diploprora championii	Pingbian	760	20.26	1648.57	86.34	990.16	0.60	Epiphyte	Sparse forest	Non-endemic
Diploprora championii	Malipo	900	18.86	1063.54	85.83	1053.97	0.99	Epiphyte	Dense forest	Non-endemic
Diploprora championii	Pingbian	1000	18.82	1648.57	86.34	990.16	0.60	Epiphyte	Dense forest	Non-endemic
Diploprora championii	Malipo	1100	17.66	1063.54	85.83	1053.97	0.99	Epiphyte	Shrubland	Non-endemic
Diploprora championii	Maguan	1200	17.81	1330.52	83.25	1086.43	0.82	Epiphyte	Dense forest	Non-endemic
Diploprora championii	Pingbian	1250	17.32	1648.57	86.34	990.16	0.60	Epiphyte	Dense forest	Non-endemic
Diploprora championii	Pingbian	1320	16.90	1648.57	86.34	990.16	0.60	Epiphyte	Dense forest	Non-endemic
Epigeneium amplum	Malipo	1000	18.26	1063.54	85.83	1053.97	0.99	Epiphyte	Dense forest	Non-endemic
Epigeneium amplum	Xichou	1000	18.93	1267.54	82.98	985.97	0.78	Epiphyte	Dense forest	Non-endemic
Epigeneium amplum	Malipo	1080	17.78	1063.54	85.83	1053.97	0.99	Epiphyte	Dense forest	Non-endemic
Epigeneium amplum	Gongshan	1320	16.16	1738.42	78.47	860.43	0.50	Epiphyte	Forest edge	Non-endemic
Epigeneium amplum	Pingbian	1400	16.42	1648.57	86.34	990.16	0.60	Epiphyte	Dense forest	Non-endemic
Epigeneium amplum	Xichou	1500	15.93	1267.54	82.98	985.97	0.78	Epiphyte	Dense forest	Non-endemic

Epigeneium amplum	Xichou	1500	15.93	1267.54	82.98	985.97	0.78	Epiphyte	Dense forest	Non-endemic
Epigeneium clemensiae	Yongde	2360	12.97	1266.25	69.00	1283.63	1.01	Epiphyte	Shrubland	Non-endemic
Epigeneium fargesii	Pingbian	1300	17.02	1648.57	86.34	990.16	0.60	Epiphyte	Dense forest	Non-endemic
Epigeneium fuscescens	Jingdong	1350	17.48	1128.40	76.80	1142.11	1.01	Epiphyte	Dense forest	Non-endemic
Epigeneium fuscescens	Jingdong	1700	15.38	1128.40	76.80	1142.11	1.01	Epiphyte	Dense forest	Non-endemic
Epigeneium rotundatum	Dali	1300	19.02	1082.70	68.61	1256.27	1.16	Epiphyte	Dense forest	Non-endemic
Epigeneium rotundatum	Binchuan	1580	17.22	573.87	62.93	1418.42	2.47	Epiphyte	Dense forest	Non-endemic
Epigeneium rotundatum	Pingbian	1650	14.92	1648.57	86.34	990.16	0.60	Epiphyte	Dense forest	Non-endemic
Epigeneium rotundatum	Gongshan	1900	12.68	1738.42	78.47	860.43	0.50	Epiphyte	Dense forest	Non-endemic
Epigeneium rotundatum	Weixi	1900	14.03	970.70	69.95	1021.03	1.05	Epiphyte	Dense forest	Non-endemic
Epigeneium rotundatum	Fugong	2000	12.06	1441.43	79.99	906.18	0.63	Epiphyte	Dense forest	Non-endemic
Epigeneium rotundatum	Gongshan	2300	10.28	1738.42	78.47	860.43	0.50	Epiphyte	Dense forest	Non-endemic
Epigeneium rotundatum	Jingdong	2300	11.78	1128.40	76.80	1142.11	1.01	Epiphyte	Shrubland	Non-endemic
Epigeneium rotundatum	Lushui	2650	10.15	1195.57	70.63	911.65	0.76	Epiphyte	Dense forest	Non-endemic
Epigeneium yunnanense	Gongshan	1380	15.80	1738.42	78.47	860.43	0.50	Epiphyte	Dense forest	Endemic
Epigeneium yunnanense	Gongshan	2200	10.88	1738.42	78.47	860.43	0.50	Epiphyte	Dense forest	Endemic
Epigeneium yunnanense	Lijiang	2300	13.27	982.53	63.24	1077.65	1.10	Epiphyte	Dense forest	Endemic
Epigeneium yunnanense	Gongshan	2400	9.68	1738.42	78.47	860.43	0.50	Epiphyte	Dense forest	Endemic
Epigeneium yunnanense	Luchun	2500	11.70	2013.54	78.64	1151.56	0.57	Epiphyte	Dense forest	Endemic
Epipactis helleborine	Gongshan	1000	18.08	1738.42	78.47	860.43	0.50	Terrestrial	Open site	Non-endemic
Epipactis helleborine	Jiangchuan	1700	15.80	872.74	74.72	1181.99	1.35	Terrestrial	Dense forest	Non-endemic
Epipactis helleborine	Yiliang	1900	10.88	751.45	71.67	1097.64	1.46	Terrestrial	Dense forest	Non-endemic
Epipactis helleborine	Gongshan	2000	12.08	1738.42	78.47	860.43	0.50	Terrestrial	Forest edge	Non-endemic
Epipactis helleborine	Yuliang	2020	13.40	905.45	75.03	1218.48	1.35	Terrestrial	Dense forest	Non-endemic
Epipactis helleborine	Jingdong	2080	13.10	1128.40	76.80	1142.11	1.01	Terrestrial	Dense forest	Non-endemic
Epipactis helleborine	Hekou	2080	11.27	1768.58	84.25	1166.26	0.66	Terrestrial	Open site	Non-endemic
Epipactis helleborine	Jingdong	2100	12.98	1128.40	76.80	1142.11	1.01	Terrestrial	Dense forest	Non-endemic
Epipactis helleborine	Kunming	2100	13.68	1019.14	72.30	1197.62	1.18	Terrestrial	Dense forest	Non-endemic
Epipactis helleborine	Yangbi	2300	12.19	1044.23	72.19	1161.15	1.11	Terrestrial	Dense forest	Non-endemic
Epipactis helleborine	Songming	2300	11.79	1010.18	74.14	1134.24	1.12	Terrestrial	Dense forest	Non-endemic
Epipactis helleborine	Gongshan	2300	10.28	1738.42	78.47	860.43	0.50	Terrestrial	Grassy slope	Non-endemic
Epipactis helleborine	Kunming	2350	12.18	1019.14	72.30	1197.62	1.18	Terrestrial	Grassy slope	Non-endemic
Epipactis helleborine	Yangbi	2400	11.59	1044.23	72.19	1161.15	1.11	Terrestrial	Dense forest	Non-endemic
Epipactis helleborine	Lushui	2500	11.05	1195.57	70.63	911.65	0.76	Terrestrial	Forest edge	Non-endemic
Epipactis helleborine	Luquan	2500	10.72	965.09	73.60	1160.89	1.20	Terrestrial	Sparse forest	Non-endemic
Epipactis helleborine	Yongde	2560	11.77	1266.25	69.00	1283.63	1.01	Terrestrial	Forest edge	Non-endemic

Epipactis helleborine	Zhongdian	2690	9.30	641.73	69.05	914.89	1.43	Terrestrial	Dense forest	Non-endemic
Epipactis helleborine	Deqin	2700	8.97	639.48	70.85	896.66	1.40	Terrestrial	Dense forest	Non-endemic
Epipactis helleborine	Deqin	2700	8.97	639.48	70.85	896.66	1.40	Terrestrial	Grassy slope	Non-endemic
Epipactis helleborine	Zhongdian	2780	8.76	641.73	69.05	914.89	1.43	Terrestrial	Grassy slope	Non-endemic
Epipactis helleborine	Dongchuan	2800	9.03	1021.73	71.65	1186.61	1.16	Terrestrial	Shrubland	Non-endemic
Epipactis helleborine	Zhongdian	2800	8.64	641.73	69.05	914.89	1.43	Terrestrial	Sparse forest	Non-endemic
Epipactis helleborine	Lijiang	2900	9.67	982.53	63.24	1077.65	1.10	Terrestrial	Dense forest	Non-endemic
Epipactis helleborine	Lijiang	2900	9.67	982.53	63.24	1077.65	1.10	Terrestrial	Open site	Non-endemic
Epipactis helleborine	Yongde	2990	9.19	1266.25	69.00	1283.63	1.01	Terrestrial	Dense forest	Non-endemic
Epipactis helleborine	Weixi	3000	7.43	970.70	69.95	1021.03	1.05	Terrestrial	Forest edge	Non-endemic
Epipactis helleborine	Gongshan	3000	6.08	1738.42	78.47	860.43	0.50	Terrestrial	Grassy slope	Non-endemic
Epipactis helleborine	Deqin	3000	7.17	639.48	70.85	896.66	1.40	Terrestrial	Grassy slope	Non-endemic
Epipactis helleborine	Eryuan	3000	8.53	745.16	68.27	1210.94	1.63	Terrestrial	Meadow	Non-endemic
Epipactis helleborine	Dali	3100	8.22	1082.70	68.61	1256.27	1.16	Terrestrial	Grassy slope	Non-endemic
Epipactis helleborine	Dali	3100	8.22	1082.70	68.61	1256.27	1.16	Terrestrial	Open site	Non-endemic
Epipactis helleborine	Zhongdian	3190	6.30	641.73	69.05	914.89	1.43	Terrestrial	Dense forest	Non-endemic
Epipactis helleborine	Luquan	3190	6.58	965.09	73.60	1160.89	1.20	Terrestrial	Shrubland	Non-endemic
Epipactis helleborine	Zhongdian	3200	6.24	641.73	69.05	914.89	1.43	Terrestrial	Grassy slope	Non-endemic
Epipactis helleborine	Zhongdian	3280	5.76	641.73	69.05	914.89	1.43	Terrestrial	Dense forest	Non-endemic
Epipactis helleborine	Zhongdian	3300	5.64	641.73	69.05	914.89	1.43	Terrestrial	Grassy slope	Non-endemic
Epipactis helleborine	Deqin	3500	4.17	639.48	70.85	896.66	1.40	Terrestrial	Dense forest	Non-endemic
Epipactis helleborine	Zhongdian	3800	2.64	641.73	69.05	914.89	1.43	Terrestrial	Dense forest	Non-endemic
Epipactis mairei	Zhenxiong	1500	12.44	899.34	83.70	852.28	0.95	Terrestrial	Dense forest	Non-endemic
Epipactis mairei	Luoping	2100	11.44	1686.24	82.51	1040.03	0.62	Terrestrial	Shrubland	Non-endemic
Epipactis mairei	Eryuan	2400	12.13	745.16	68.27	1210.94	1.63	Terrestrial	Sparse forest	Non-endemic
Epipactis mairei	Lushui	2500	11.05	1195.57	70.63	911.65	0.76	Terrestrial	Grassy slope	Non-endemic
Epipactis mairei	Heqing	2550	11.49	977.00	65.16	1211.84	1.24	Terrestrial	Open site	Non-endemic
Epipactis mairei	Yongde	2560	11.77	1266.25	69.00	1283.63	1.01	Terrestrial	Grassy slope	Non-endemic
Epipactis mairei	Zhenkang	2580	9.36	1602.96	81.14	1089.09	0.68	Terrestrial	Grassy slope	Non-endemic
Epipactis mairei	Lijiang	2600	11.47	982.53	63.24	1077.65	1.10	Terrestrial	Grassy slope	Non-endemic
Epipactis mairei	Dali	2600	11.22	1082.70	68.61	1256.27	1.16	Terrestrial	Grassy slope	Non-endemic
Epipactis mairei	Eryuan	2740	10.09	745.16	68.27	1210.94	1.63	Terrestrial	Grassy slope	Non-endemic
Epipactis mairei	Lijiang	2800	10.27	982.53	63.24	1077.65	1.10	Terrestrial	Dense forest	Non-endemic
Epipactis mairei	Lijiang	2900	9.67	982.53	63.24	1077.65	1.10	Terrestrial	Forest edge	Non-endemic
Epipactis mairei	Zhenkang	2900	7.44	1602.96	81.14	1089.09	0.68	Terrestrial	Grassy slope	Non-endemic
Epipactis mairei	Weixi	3000	7.43	970.70	69.95	1021.03	1.05	Terrestrial	Dense forest	Non-endemic

Epipactis mairei	Lijiang	3000	9.07	982.53	63.24	1077.65	1.10	Terrestrial	Grassy slope	Non-endemic
Epipactis mairei	Deqin	3000	7.17	639.48	70.85	896.66	1.40	Terrestrial	Grassy slope	Non-endemic
Epipactis mairei	Dongchuan	3000	7.83	1021.73	71.65	1186.61	1.16	Terrestrial	Grassy slope	Non-endemic
Epipactis mairei	Dongchuan	3000	7.83	1021.73	71.65	1186.61	1.16	Terrestrial	Open site	Non-endemic
Epipactis mairei	Weixi	3200	6.23	970.70	69.95	1021.03	1.05	Terrestrial	Dense forest	Non-endemic
Epipactis mairei	Zhongdian	3400	5.04	641.73	69.05	914.89	1.43	Terrestrial	Open site	Non-endemic
Epipactis yunnanensis	Lijiang	3300	7.27	982.53	63.24	1077.65	1.10	Terrestrial	Meadow	Non-endemic
Epipogium aphyllum	Gongshan	2350	9.98	1738.42	78.47	860.43	0.50	Saprophyte	Dense forest	Non-endemic
Epipogium aphyllum	Gongshan	3400	3.68	1738.42	78.47	860.43	0.50	Saprophyte	Dense forest	Non-endemic
Epipogium roseum	Jinping	500	22.49	2305.17	83.81	1030.46	0.45	Saprophyte	Dense forest	Non-endemic
Epipogium roseum	Funing	700	19.42	1161.58	79.07	1147.17	0.99	Saprophyte	Dense forest	Non-endemic
Epipogium roseum	Pingbian	1250	17.32	1648.57	86.34	990.16	0.60	Saprophyte	Dense forest	Non-endemic
Epipogium roseum	Lancang	1300	17.90	1596.50	77.88	1183.80	0.74	Saprophyte	Dense forest	Non-endemic
Epipogium roseum	Xichou	1420	16.41	1267.54	82.98	985.97	0.78	Saprophyte	Dense forest	Non-endemic
Eria acervata	Jinghong	1100	19.12	1161.08	80.46	1256.19	1.08	Epiphyte	Shrubland	Non-endemic
Eria amica	Cangyuan	1250	17.72	1733.34	81.63	1060.82	0.61	Epiphyte	Dense forest	Non-endemic
Eria amica	Menghai	1300	17.76	1314.38	80.83	1150.91	0.88	Epiphyte	Forest edge	Non-endemic
Eria amica	Menghai	1300	17.76	1314.38	80.83	1150.91	0.88	Epiphyte	Sparse forest	Non-endemic
Eria amica	Menghai	1540	16.32	1314.38	80.83	1150.91	0.88	Epiphyte	Shrubland	Non-endemic
Eria bambusifolia	Luchun	600	23.10	2013.54	78.64	1151.56	0.57	Epiphyte	Dense forest	Non-endemic
Eria bambusifolia	Luchun	780	22.02	2013.54	78.64	1151.56	0.57	Epiphyte	Dense forest	Non-endemic
Eria bambusifolia	Longling	1510	15.06	2098.66	84.65	973.45	0.46	Epiphyte	Dense forest	Non-endemic
Eria bambusifolia	Luchun	1580	17.22	2013.54	78.64	1151.56	0.57	Epiphyte	Dense forest	Non-endemic
Eria clausa	Jinghong	1100	19.12	1161.08	80.46	1256.19	1.08	Epiphyte	Shrubland	Non-endemic
Eria clausa	Malipo	1200	17.06	1063.54	85.83	1053.97	0.99	Epiphyte	Dense forest	Non-endemic
Eria clausa	Cangyuan	1200	18.02	1733.34	81.63	1060.82	0.61	Epiphyte	Dense forest	Non-endemic
Eria clausa	Menghai	1300	17.76	1314.38	80.83	1150.91	0.88	Epiphyte	Dense forest	Non-endemic
Eria clausa	Maguan	1400	16.61	1330.52	83.25	1086.43	0.82	Epiphyte	Sparse forest	Non-endemic
Eria clausa	Shuangjiang	1480	17.10	1006.64	75.26	1272.13	1.26	Epiphyte	Dense forest	Non-endemic
Eria clausa	Zhenkang	1640	15.00	1602.96	81.14	1089.09	0.68	Epiphyte	Dense forest	Non-endemic
Eria clausa	Lancang	1700	15.50	1596.50	77.88	1183.80	0.74	Epiphyte	Dense forest	Non-endemic
Eria clausa	Lancang	1700	15.50	1596.50	77.88	1183.80	0.74	Epiphyte	Dense forest	Non-endemic
Eria corneri	Mengla	680	21.12	1514.70	84.25	1146.68	0.76	Epiphyte	Dense forest	Non-endemic
Eria corneri	Funing	700	19.42	1161.58	79.07	1147.17	0.99	Epiphyte	Open site	Non-endemic
Eria corneri	Luchun	800	21.90	2013.54	78.64	1151.56	0.57	Epiphyte	Dense forest	Non-endemic
Eria corneri	Mengla	800	20.40	1514.70	84.25	1146.68	0.76	Epiphyte	Dense forest	Non-endemic

Eria corneri	Xichou	1000	18.93	1267.54	82.98	985.97	0.78	Epiphyte	Dense forest	Non-endemic
Eria corneri	Pingbian	1100	18.22	1648.57	86.34	990.16	0.60	Epiphyte	Dense forest	Non-endemic
Eria corneri	Jinghong	1100	19.12	1161.08	80.46	1256.19	1.08	Epiphyte	Dense forest	Non-endemic
Eria corneri	Malipo	1200	17.06	1063.54	85.83	1053.97	0.99	Epiphyte	Shrubland	Non-endemic
Eria corneri	Hekou	1209	16.50	1768.58	84.25	1166.26	0.66	Epiphyte	Dense forest	Non-endemic
Eria corneri	Xichou	1300	17.13	1267.54	82.98	985.97	0.78	Epiphyte	Dense forest	Non-endemic
Eria corneri	Xichou	1500	15.93	1267.54	82.98	985.97	0.78	Epiphyte	Dense forest	Non-endemic
Eria corneri	Hekou	1300	15.95	1768.58	84.25	1166.26	0.66	Epiphyte	Dense forest	Non-endemic
Eria coronaria	Hekou	900	18.35	1768.58	84.25	1166.26	0.66	Epiphyte	Dense forest	Non-endemic
Eria coronaria	Yanshan	1100	18.86	1003.57	79.42	1172.21	1.17	Epiphyte	Dense forest	Non-endemic
Eria coronaria	Yanshan	1200	18.26	1003.57	79.42	1172.21	1.17	Epiphyte	Dense forest	Non-endemic
Eria coronaria	Malipo	1200	17.06	1063.54	85.83	1053.97	0.99	Epiphyte	Shrubland	Non-endemic
Eria coronaria	Gongshan	1320	16.16	1738.42	78.47	860.43	0.50	Epiphyte	Dense forest	Non-endemic
Eria coronaria	Gongshan	1400	15.68	1738.42	78.47	860.43	0.50	Epiphyte	Dense forest	Non-endemic
Eria coronaria	Xichou	1400	16.53	1267.54	82.98	985.97	0.78	Epiphyte	Dense forest	Non-endemic
Eria coronaria	Gongshan	1400	15.68	1738.42	78.47	860.43	0.50	Epiphyte	Dense forest	Non-endemic
Eria coronaria	Gongshan	1400	15.68	1738.42	78.47	860.43	0.50	Epiphyte	Shrubland	Non-endemic
Eria coronaria	Xichou	1450	16.23	1267.54	82.98	985.97	0.78	Epiphyte	Dense forest	Non-endemic
Eria coronaria	Xichou	1450	16.23	1267.54	82.98	985.97	0.78	Epiphyte	Open site	Non-endemic
Eria coronaria	Xichou	1500	15.93	1267.54	82.98	985.97	0.78	Epiphyte	Dense forest	Non-endemic
Eria coronaria	Xichou	1500	15.93	1267.54	82.98	985.97	0.78	Epiphyte	Sparse forest	Non-endemic
Eria coronaria	Gongshan	1580	14.60	1738.42	78.47	860.43	0.50	Epiphyte	Dense forest	Non-endemic
Eria coronaria	Pingbian	1800	14.02	1648.57	86.34	990.16	0.60	Epiphyte	Dense forest	Non-endemic
Eria coronaria	Jingdong	1950	13.88	1128.40	76.80	1142.11	1.01	Epiphyte	Dense forest	Non-endemic
Eria coronaria	Fugong	2000	12.06	1441.43	79.99	906.18	0.63	Epiphyte	Dense forest	Non-endemic
Eria coronaria	Hekou	2020	11.63	1768.58	84.25	1166.26	0.66	Epiphyte	Dense forest	Non-endemic
Eria coronaria	Malipo	2100	11.66	1063.54	85.83	1053.97	0.99	Epiphyte	Shrubland	Non-endemic
Eria coronaria	Pingbian	2200	11.62	1648.57	86.34	990.16	0.60	Epiphyte	Dense forest	Non-endemic
Eria coronaria	Jingdong	2500	10.58	1128.40	76.80	1142.11	1.01	Epiphyte	Shrubland	Non-endemic
Eria coronaria	Zhenkang	2700	8.64	1602.96	81.14	1089.09	0.68	Epiphyte	Dense forest	Non-endemic
Eria dasyphylla	Menghai	1350	17.46	1314.38	80.83	1150.91	0.88	Epiphyte	Dense forest	Non-endemic
Eria dasyphylla	Zhenkang	1600	15.24	1602.96	81.14	1089.09	0.68	Epiphyte	Dense forest	Non-endemic
Eria dasyphylla	Jinghong	1950	14.02	1161.08	80.46	1256.19	1.08	Epiphyte	Dense forest	Non-endemic
Eria donnaiensis	Maguan	1000	19.01	1330.52	83.25	1086.43	0.82	Epiphyte	Shrubland	Non-endemic
Eria donnaiensis	Menghai	1500	16.56	1314.38	80.83	1150.91	0.88	Epiphyte	Shrubland	Non-endemic
Eria graminifolia	Gongshan	1500	15.08	1738.42	78.47	860.43	0.50	Epiphyte	Dense forest	Non-endemic

Eria graminifolia	Gongshan	1700	13.88	1738.42	78.47	860.43	0.50	Epiphyte	Dense forest	Non-endemic
Eria graminifolia	Gongshan	1800	13.28	1738.42	78.47	860.43	0.50	Epiphyte	Dense forest	Non-endemic
Eria graminifolia	Lushui	1900	14.65	1195.57	70.63	911.65	0.76	Epiphyte	Dense forest	Non-endemic
Eria graminifolia	Gongshan	2000	12.08	1738.42	78.47	860.43	0.50	Epiphyte	Sparse forest	Non-endemic
Eria graminifolia	Lushui	2270	12.43	1195.57	70.63	911.65	0.76	Epiphyte	Dense forest	Non-endemic
Eria graminifolia	Baoshan	2400	11.36	992.38	73.87	1160.15	1.17	Epiphyte	Dense forest	Non-endemic
Eria graminifolia	Fugong	2400	9.66	1441.43	79.99	906.18	0.63	Epiphyte	Dense forest	Non-endemic
Eria graminifolia	Lushui	2500	11.05	1195.57	70.63	911.65	0.76	Epiphyte	Dense forest	Non-endemic
Eria graminifolia	Gongshan	2600	8.48	1738.42	78.47	860.43	0.50	Epiphyte	Dense forest	Non-endemic
Eria graminifolia	Gongshan	2700	7.88	1738.42	78.47	860.43	0.50	Epiphyte	Dense forest	Non-endemic
Eria graminifolia	Gongshan	3500	3.08	1738.42	78.47	860.43	0.50	Epiphyte	Dense forest	Non-endemic
Eria hainanensis	Menghai	1350	17.46	1314.38	80.83	1150.91	0.88	Epiphyte	Sparse forest	Non-endemic
Eria javanica	Jinghong	1000	19.72	1161.08	80.46	1256.19	1.08	Epiphyte	Dense forest	Non-endemic
Eria javanica	Jinghong	1150	18.82	1161.08	80.46	1256.19	1.08	Epiphyte	Dense forest	Non-endemic
Eria marginata	Gongshan	1300	16.28	1738.42	78.47	860.43	0.50	Epiphyte	Dense forest	Non-endemic
Eria marginata	Gongshan	1400	15.68	1738.42	78.47	860.43	0.50	Epiphyte	Dense forest	Non-endemic
Eria marginata	Menghai	1530	16.38	1314.38	80.83	1150.91	0.88	Epiphyte	Dense forest	Non-endemic
Eria marginata	Luxi	1750	14.58	1650.33	79.33	1183.39	0.72	Epiphyte	Dense forest	Non-endemic
Eria marginata	Zhenkang	2000	12.84	1602.96	81.14	1089.09	0.68	Epiphyte	Dense forest	Non-endemic
Eria marginata	Luxi	2000	13.08	1650.33	79.33	1183.39	0.72	Epiphyte	Dense forest	Non-endemic
Eria muscicola	Menghai	1750	15.06	1314.38	80.83	1150.91	0.88	Epiphyte	Dense forest	Non-endemic
Eria muscicola	Jinghong	1800	14.92	1161.08	80.46	1256.19	1.08	Epiphyte	Dense forest	Non-endemic
Eria muscicola	Menghai	1800	14.76	1314.38	80.83	1150.91	0.88	Epiphyte	Dense forest	Non-endemic
Eria muscicola	Lincang	2500	11.52	1165.84	72.08	1167.73	1.00	Epiphyte	Dense forest	Non-endemic
Eria obvia	Menglian	1050	19.28	1357.68	80.47	1185.94	0.87	Epiphyte	Dense forest	Non-endemic
Eria obvia	Menghai	1340	17.52	1314.38	80.83	1150.91	0.88	Epiphyte	Shrubland	Non-endemic
Eria obvia	Menghai	1500	16.56	1314.38	80.83	1150.91	0.88	Epiphyte	Shrubland	Non-endemic
Eria pannea	Menghai	640	21.72	1314.38	80.83	1150.91	0.88	Epiphyte	Dense forest	Non-endemic
Eria pannea	Mengla	900	19.80	1514.70	84.25	1146.68	0.76	Epiphyte	Sparse forest	Non-endemic
Eria pannea	Jinghong	1100	19.12	1161.08	80.46	1256.19	1.08	Epiphyte	Sparse forest	Non-endemic
Eria pannea	Menghai	1150	18.66	1314.38	80.83	1150.91	0.88	Epiphyte	Dense forest	Non-endemic
Eria pannea	Menghai	1300	17.76	1314.38	80.83	1150.91	0.88	Epiphyte	Sparse forest	Non-endemic
Eria pannea	Menghai	1300	17.76	1314.38	80.83	1150.91	0.88	Epiphyte	Sparse forest	Non-endemic
Eria pannea	Jinghong	1349	17.63	1161.08	80.46	1256.19	1.08	Epiphyte	Dense forest	Non-endemic
Eria pannea	Jinghong	1400	17.32	1161.08	80.46	1256.19	1.08	Epiphyte	Dense forest	Non-endemic
Eria pannea	Jinping	1450	16.79	2305.17	83.81	1030.46	0.45	Epiphyte	Sparse forest	Non-endemic

Eria pannea	Menghai	1530	16.38	1314.38	80.83	1150.91	0.88	Epiphyte	Dense forest	Non-endemic
Eria pannea	Zhenkang	1600	15.24	1602.96	81.14	1089.09	0.68	Epiphyte	Dense forest	Non-endemic
Eria pannea	Yongde	1700	16.93	1266.25	69.00	1283.63	1.01	Epiphyte	Dense forest	Non-endemic
Eria pannea	Shuangjiang	2200	12.78	1006.64	75.26	1272.13	1.26	Epiphyte	Dense forest	Non-endemic
Eria pudica	Jinghong	1530	16.54	1161.08	80.46	1256.19	1.08	Epiphyte	Shrubland	Non-endemic
Eria pulchella	Menghai	1100	18.96	1314.38	80.83	1150.91	0.88	Epiphyte	Dense forest	Non-endemic
Eria pulchella	Menghai	1600	15.96	1314.38	80.83	1150.91	0.88	Epiphyte	Shrubland	Non-endemic
Eria pulchella	Lancang	1700	15.50	1596.50	77.88	1183.80	0.74	Epiphyte	Dense forest	Non-endemic
Eria pusilla	Malipo	1500	15.26	1063.54	85.83	1053.97	0.99	Epiphyte	Sparse forest	Non-endemic
Eria rhomboidalia	Funing	700	19.42	1161.58	79.07	1147.17	0.99	Epiphyte	Dense forest	Non-endemic
Eria rhomboidalia	Xichou	870	19.71	1267.54	82.98	985.97	0.78	Epiphyte	Open site	Non-endemic
Eria rhomboidalia	Funing	1100	17.02	1161.58	79.07	1147.17	0.99	Epiphyte	Sparse forest	Non-endemic
Eria rhomboidalia	Xichou	1650	15.03	1267.54	82.98	985.97	0.78	Epiphyte	Sparse forest	Non-endemic
Eria spicata	Xichou	1200	17.73	1267.54	82.98	985.97	0.78	Epiphyte	Dense forest	Non-endemic
Eria spicata	Yanshan	1200	18.26	1003.57	79.42	1172.21	1.17	Epiphyte	Dense forest	Non-endemic
Eria spicata	Xichou	1300	17.13	1267.54	82.98	985.97	0.78	Epiphyte	Dense forest	Non-endemic
Eria spicata	Jinping	1690	15.35	2305.17	83.81	1030.46	0.45	Epiphyte	Dense forest	Non-endemic
Eria spicata	Jinghong	1800	14.92	1161.08	80.46	1256.19	1.08	Epiphyte	Dense forest	Non-endemic
Eria spicata	Yongde	1800	16.33	1266.25	69.00	1283.63	1.01	Epiphyte	Grassy slope	Non-endemic
Eria spicata	Jingdong	2100	12.98	1128.40	76.80	1142.11	1.01	Epiphyte	Dense forest	Non-endemic
Eria spicata	Jingdong	2400	11.18	1128.40	76.80	1142.11	1.01	Epiphyte	Dense forest	Non-endemic
Eria spicata	Luchun	2500	11.70	2013.54	78.64	1151.56	0.57	Epiphyte	Dense forest	Non-endemic
Eria spicata	Gongshan	2500	9.08	1738.42	78.47	860.43	0.50	Epiphyte	Dense forest	Non-endemic
Eria spicata	Yongde	2500	12.13	1266.25	69.00	1283.63	1.01	Epiphyte	Dense forest	Non-endemic
Eria spicata	Yongde	2500	12.13	1266.25	69.00	1283.63	1.01	Epiphyte	Dense forest	Non-endemic
Eria spicata	Lincang	2800	9.72	1165.84	72.08	1167.73	1.00	Epiphyte	Dense forest	Non-endemic
Eria stricta	Zhenkang	1100	18.24	1602.96	81.14	1089.09	0.68	Epiphyte	Dense forest	Non-endemic
Eria stricta	Xichou	1300	17.13	1267.54	82.98	985.97	0.78	Epiphyte	Dense forest	Non-endemic
Eria stricta	Malipo	1500	15.26	1063.54	85.83	1053.97	0.99	Epiphyte	Dense forest	Non-endemic
Eria stricta	Luchun	1500	17.70	2013.54	78.64	1151.56	0.57	Epiphyte	Dense forest	Non-endemic
Eria stricta	Longling	1600	14.52	2098.66	84.65	973.45	0.46	Epiphyte	Dense forest	Non-endemic
Eria stricta	Luxi	1750	14.58	1650.33	79.33	1183.39	0.72	Epiphyte	Dense forest	Non-endemic
Eria stricta	Luxi	1750	14.58	1650.33	79.33	1183.39	0.72	Epiphyte	Dense forest	Non-endemic
Eria stricta	Luxi	1750	14.58	1650.33	79.33	1183.39	0.72	Epiphyte	Dense forest	Non-endemic
Eria stricta	Luxi	1750	14.58	1650.33	79.33	1183.39	0.72	Epiphyte	Dense forest	Non-endemic
Eria stricta	Longling	1800	13.32	2098.66	84.65	973.45	0.46	Epiphyte	Dense forest	Non-endemic

Eria stricta	Luxi	2000	13.08	1650.33	79.33	1183.39	0.72	Epiphyte	Dense forest	Non-endemic
Eria szetschuanica	Weixi	2300	11.63	970.70	69.95	1021.03	1.05	Epiphyte	Forest edge	Non-endemic
Eria tomentosa	Luchun	600	23.10	2013.54	78.64	1151.56	0.57	Epiphyte	Forest edge	Non-endemic
Eria tomentosa	Mengla	650	21.30	1514.70	84.25	1146.68	0.76	Epiphyte	Dense forest	Non-endemic
Eria tomentosa	Jinghong	700	21.52	1161.08	80.46	1256.19	1.08	Epiphyte	Sparse forest	Non-endemic
Eria tomentosa	Cangyuan	800	20.42	1733.34	81.63	1060.82	0.61	Epiphyte	Dense forest	Non-endemic
Eria tomentosa	Mengla	800	20.40	1514.70	84.25	1146.68	0.76	Epiphyte	Forest edge	Non-endemic
Eria tomentosa	Jinghong	900	20.32	1161.08	80.46	1256.19	1.08	Epiphyte	Dense forest	Non-endemic
Eria tomentosa	Mengla	900	19.80	1514.70	84.25	1146.68	0.76	Epiphyte	Forest edge	Non-endemic
Eria tomentosa	Jinghong	1050	19.42	1161.08	80.46	1256.19	1.08	Epiphyte	Dense forest	Non-endemic
Eria tomentosa	Menghai	1100	18.96	1314.38	80.83	1150.91	0.88	Epiphyte	Dense forest	Non-endemic
Eria tomentosa	Menghai	1180	18.48	1314.38	80.83	1150.91	0.88	Epiphyte	Dense forest	Non-endemic
Eria tomentosa	Menghai	1500	16.56	1314.38	80.83	1150.91	0.88	Epiphyte	Dense forest	Non-endemic
Eria tomentosa	Menghai	1250	18.06	1314.38	80.83	1150.91	0.88	Epiphyte	Sparse forest	Non-endemic
Eria yanshanensis	Yanshan	1100	18.86	1003.57	79.42	1172.21	1.17	Epiphyte	Dense forest	Endemic
Eria yunnanensis	Jinghong	1500	16.72	1161.08	80.46	1256.19	1.08	Epiphyte	Dense forest	Endemic
Erythrodes chinensis	Mengla	1750	14.70	1514.70	84.25	1146.68	0.76	Terrestrial	Dense forest	Non-endemic
Esmeralda clarkei	Jingdong	1700	15.38	1128.40	76.80	1142.11	1.01	Epiphyte	Dense forest	Non-endemic
Esmeralda clarkei	Jingdong	2100	12.98	1128.40	76.80	1142.11	1.01	Epiphyte	Forest edge	Non-endemic
Eulophia bracteosa	Pingbian	1300	17.02	1648.57	86.34	990.16	0.60	Terrestrial	Shrubland	Non-endemic
Eulophia spectabilis	Hekou	200	22.55	1768.58	84.25	1166.26	0.66	Terrestrial	Dense forest	Non-endemic
Eulophia spectabilis	Jianshui	1430	17.97	793.10	71.73	1413.81	1.78	Terrestrial	Forest edge	Non-endemic
Eulophia spectabilis	Lancang	1500	16.70	1596.50	77.88	1183.80	0.74	Terrestrial	Dense forest	Non-endemic
Eulophia spectabilis	Lancang	1550	16.40	1596.50	77.88	1183.80	0.74	Terrestrial	Grassy slope	Non-endemic
Flickingeria albopurpurea	Gongshan	1600	14.48	1738.42	78.47	860.43	0.50	Epiphyte	Dense forest	Non-endemic
Flickingeria bicolor	Mengla	900	19.80	1514.70	84.25	1146.68	0.76	Epiphyte	Dense forest	Endemic
Flickingeria concolor	Jinghong	670	21.70	1161.08	80.46	1256.19	1.08	Epiphyte	Dense forest	Endemic
Flickingeria fimbriata	Hekou	200	22.55	1768.58	84.25	1166.26	0.66	Epiphyte	Dense forest	Non-endemic
Flickingeria fimbriata	Maguan	1050	18.71	1330.52	83.25	1086.43	0.82	Epiphyte	Sparse forest	Non-endemic
Flickingeria fimbriata	Malipo	1200	17.06	1063.54	85.83	1053.97	0.99	Epiphyte	Dense forest	Non-endemic
Flickingeria tricarinata	Mengla	800	20.40	1514.70	84.25	1146.68	0.76	Epiphyte	Dense forest	Endemic
Galeola faberi	Jinping	1880	14.21	2305.17	83.81	1030.46	0.45	Saprophyte	Dense forest	Non-endemic
Galeola faberi	Gongshan	2100	11.48	1738.42	78.47	860.43	0.50	Saprophyte	Dense forest	Non-endemic
Galeola faberi	Pingbian	2200	11.62	1648.57	86.34	990.16	0.60	Saprophyte	Dense forest	Non-endemic
Galeola faberi	Weixi	2300	11.63	970.70	69.95	1021.03	1.05	Saprophyte	Dense forest	Non-endemic
Galeola faberi	Weixi	2500	10.43	970.70	69.95	1021.03	1.05	Saprophyte	Dense forest	Non-endemic

Galeola faberi	Fugong	3100	5.46	1441.43	79.99	906.18	0.63	Saprophyte	Dense forest	Non-endemic
Galeola lindleyana	Gongshan	1250	16.58	1738.42	78.47	860.43	0.50	Saprophyte	Dense forest	Non-endemic
Galeola lindleyana	Hekou	1250	16.25	1768.58	84.25	1166.26	0.66	Saprophyte	Dense forest	Non-endemic
Galeola lindleyana	Gongshan	1250	16.58	1738.42	78.47	860.43	0.50	Saprophyte	Dense forest	Non-endemic
Galeola lindleyana	Malipo	1300	16.46	1063.54	85.83	1053.97	0.99	Saprophyte	Dense forest	Non-endemic
Galeola lindleyana	Pingbian	1300	17.02	1648.57	86.34	990.16	0.60	Saprophyte	Dense forest	Non-endemic
Galeola lindleyana	Fugong	1300	16.26	1441.43	79.99	906.18	0.63	Saprophyte	Dense forest	Non-endemic
Galeola lindleyana	Xichou	1300	17.13	1267.54	82.98	985.97	0.78	Saprophyte	Sparse forest	Non-endemic
Galeola lindleyana	Gongshan	1350	15.98	1738.42	78.47	860.43	0.50	Saprophyte	Dense forest	Non-endemic
Galeola lindleyana	Gongshan	1400	15.68	1738.42	78.47	860.43	0.50	Saprophyte	Dense forest	Non-endemic
Galeola lindleyana	Gongshan	1450	15.38	1738.42	78.47	860.43	0.50	Saprophyte	Dense forest	Non-endemic
Galeola lindleyana	Hekou	1480	14.87	1768.58	84.25	1166.26	0.66	Saprophyte	Dense forest	Non-endemic
Galeola lindleyana	Gongshan	1500	15.08	1738.42	78.47	860.43	0.50	Saprophyte	Dense forest	Non-endemic
Galeola lindleyana	Gongshan	1500	15.08	1738.42	78.47	860.43	0.50	Saprophyte	Dense forest	Non-endemic
Galeola lindleyana	Pingbian	1500	15.82	1648.57	86.34	990.16	0.60	Saprophyte	Dense forest	Non-endemic
Galeola lindleyana	Gongshan	1550	14.78	1738.42	78.47	860.43	0.50	Saprophyte	Sparse forest	Non-endemic
Galeola lindleyana	Malipo	1600	14.66	1063.54	85.83	1053.97	0.99	Saprophyte	Dense forest	Non-endemic
Galeola lindleyana	Pingbian	1700	14.62	1648.57	86.34	990.16	0.60	Saprophyte	Dense forest	Non-endemic
Galeola lindleyana	Gongshan	1700	13.88	1738.42	78.47	860.43	0.50	Saprophyte	Dense forest	Non-endemic
Galeola lindleyana	Maguan	1700	14.81	1330.52	83.25	1086.43	0.82	Saprophyte	Dense forest	Non-endemic
Galeola lindleyana	Pingbian	1700	14.62	1648.57	86.34	990.16	0.60	Saprophyte	Dense forest	Non-endemic
Galeola lindleyana	Zhenxiong	1780	10.76	899.34	83.70	852.28	0.95	Saprophyte	Dense forest	Non-endemic
Galeola lindleyana	Daguan	1800	11.02	987.00	80.05	896.37	0.91	Saprophyte	Dense forest	Non-endemic
Galeola lindleyana	Pingbian	1840	13.78	1648.57	86.34	990.16	0.60	Saprophyte	Shrubland	Non-endemic
Galeola lindleyana	Jingdong	1900	14.18	1128.40	76.80	1142.11	1.01	Saprophyte	Dense forest	Non-endemic
Galeola lindleyana	Gongshan	1900	12.68	1738.42	78.47	860.43	0.50	Saprophyte	Dense forest	Non-endemic
Galeola lindleyana	Luchun	1900	15.30	2013.54	78.64	1151.56	0.57	Saprophyte	Dense forest	Non-endemic
Galeola lindleyana	Gongshan	1900	12.68	1738.42	78.47	860.43	0.50	Saprophyte	Forest edge	Non-endemic
Galeola lindleyana	Gongshan	1900	12.68	1738.42	78.47	860.43	0.50	Saprophyte	Shrubland	Non-endemic
Galeola lindleyana	Gongshan	1900	12.68	1738.42	78.47	860.43	0.50	Saprophyte	Shrubland	Non-endemic
Galeola lindleyana	Jingdong	1950	13.88	1128.40	76.80	1142.11	1.01	Saprophyte	Dense forest	Non-endemic
Galeola lindleyana	Fugong	2000	12.06	1441.43	79.99	906.18	0.63	Saprophyte	Dense forest	Non-endemic
Galeola lindleyana	Gongshan	2000	12.08	1738.42	78.47	860.43	0.50	Saprophyte	Dense forest	Non-endemic
Galeola lindleyana	Gongshan	2000	12.08	1738.42	78.47	860.43	0.50	Saprophyte	Dense forest	Non-endemic
Galeola lindleyana	Tengchong	2070	12.58	1501.45	78.06	857.93	0.57	Saprophyte	Dense forest	Non-endemic
Galeola lindleyana	Jingdong	2100	12.98	1128.40	76.80	1142.11	1.01	Saprophyte	Dense forest	Non-endemic

Galeola lindleyana	Jingdong	2100	12.98	1128.40	76.80	1142.11	1.01	Saprophyte	Dense forest	Non-endemic
Galeola lindleyana	Fugong	2100	11.46	1441.43	79.99	906.18	0.63	Saprophyte	Dense forest	Non-endemic
Galeola lindleyana	Lushui	2500	11.05	1195.57	70.63	911.65	0.76	Saprophyte	Dense forest	Non-endemic
Galeola lindleyana	Lijiang	2500	12.07	982.53	63.24	1077.65	1.10	Saprophyte	Dense forest	Non-endemic
Galeola lindleyana	Gongshan	2500	9.08	1738.42	78.47	860.43	0.50	Saprophyte	Dense forest	Non-endemic
Galeola lindleyana	Tengchong	2700	8.80	1501.45	78.06	857.93	0.57	Saprophyte	Forest edge	Non-endemic
Galeola lindleyana	Lijiang	3000	9.07	982.53	63.24	1077.65	1.10	Saprophyte	Dense forest	Non-endemic
Gastrochilus calceolaris	Jinghong	860	20.56	1161.08	80.46	1256.19	1.08	Epiphyte	Dense forest	Non-endemic
Gastrochilus calceolaris	Lushui	1040	19.81	1195.57	70.63	911.65	0.76	Epiphyte	Dense forest	Non-endemic
Gastrochilus calceolaris	Maguan	1200	17.81	1330.52	83.25	1086.43	0.82	Epiphyte	Dense forest	Non-endemic
Gastrochilus calceolaris	Gongshan	1300	16.28	1738.42	78.47	860.43	0.50	Epiphyte	Dense forest	Non-endemic
Gastrochilus calceolaris	Menghai	1300	17.76	1314.38	80.83	1150.91	0.88	Epiphyte	Forest edge	Non-endemic
Gastrochilus calceolaris	Gongshan	1320	16.16	1738.42	78.47	860.43	0.50	Epiphyte	Dense forest	Non-endemic
Gastrochilus calceolaris	Gongshan	1350	15.98	1738.42	78.47	860.43	0.50	Epiphyte	Dense forest	Non-endemic
Gastrochilus calceolaris	Gongshan	1350	15.98	1738.42	78.47	860.43	0.50	Epiphyte	Dense forest	Non-endemic
Gastrochilus calceolaris	Gongshan	1500	15.08	1738.42	78.47	860.43	0.50	Epiphyte	Dense forest	Non-endemic
Gastrochilus calceolaris	Jinghong	1500	16.72	1161.08	80.46	1256.19	1.08	Epiphyte	Dense forest	Non-endemic
Gastrochilus calceolaris	Jingdong	1600	15.98	1128.40	76.80	1142.11	1.01	Epiphyte	Dense forest	Non-endemic
Gastrochilus calceolaris	Fengqing	1700	16.00	1352.80	73.24	1172.52	0.87	Epiphyte	Dense forest	Non-endemic
Gastrochilus calceolaris	Gengma	1700	15.95	1327.70	77.28	1181.78	0.89	Epiphyte	Dense forest	Non-endemic
Gastrochilus calceolaris	Longling	1800	13.32	2098.66	84.65	973.45	0.46	Epiphyte	Dense forest	Non-endemic
Gastrochilus calceolaris	Longling	1800	13.32	2098.66	84.65	973.45	0.46	Epiphyte	Dense forest	Non-endemic
Gastrochilus calceolaris	Longling	1800	13.32	2098.66	84.65	973.45	0.46	Epiphyte	Dense forest	Non-endemic
Gastrochilus calceolaris	Longling	1800	13.32	2098.66	84.65	973.45	0.46	Epiphyte	Dense forest	Non-endemic
Gastrochilus calceolaris	Jingdong	1850	14.48	1128.40	76.80	1142.11	1.01	Epiphyte	Dense forest	Non-endemic
Gastrochilus calceolaris	Jingdong	1900	14.18	1128.40	76.80	1142.11	1.01	Epiphyte	Dense forest	Non-endemic
Gastrochilus calceolaris	Shuangjiang	1920	14.46	1006.64	75.26	1272.13	1.26	Epiphyte	Dense forest	Non-endemic
Gastrochilus calceolaris	Zhenkang	2000	12.84	1602.96	81.14	1089.09	0.68	Epiphyte	Dense forest	Non-endemic
Gastrochilus calceolaris	Luchun	2000	14.70	2013.54	78.64	1151.56	0.57	Epiphyte	Forest edge	Non-endemic
Gastrochilus calceolaris	Jingdong	2100	12.98	1128.40	76.80	1142.11	1.01	Epiphyte	Dense forest	Non-endemic
Gastrochilus calceolaris	Gongshan	2100	11.48	1738.42	78.47	860.43	0.50	Epiphyte	Dense forest	Non-endemic
Gastrochilus calceolaris	Lushui	2100	13.45	1195.57	70.63	911.65	0.76	Epiphyte	Dense forest	Non-endemic
Gastrochilus calceolaris	Jingdong	2100	12.98	1128.40	76.80	1142.11	1.01	Epiphyte	Forest edge	Non-endemic
Gastrochilus calceolaris	Zhongdian	2200	12.24	641.73	69.05	914.89	1.43	Epiphyte	Dense forest	Non-endemic
Gastrochilus calceolaris	Jingdong	2400	11.18	1128.40	76.80	1142.11	1.01	Epiphyte	Dense forest	Non-endemic
Gastrochilus calceolaris	Lincang	2700	10.32	1165.84	72.08	1167.73	1.00	Epiphyte	Dense forest	Non-endemic

Gastrochilus dasypogon	Jinghong	950	20.02	1161.08	80.46	1256.19	1.08	Epiphyte	Shrubland	Non-endemic
Gastrochilus diannanensis	Mengla	750	20.70	1514.70	84.25	1146.68	0.76	Epiphyte	Dense forest	Non-endemic
Gastrochilus distichus	Jingdong	2000	13.58	1128.40	76.80	1142.11	1.01	Epiphyte	Dense forest	Non-endemic
Gastrochilus distichus	Hekou	2000	11.75	1768.58	84.25	1166.26	0.66	Epiphyte	Dense forest	Non-endemic
Gastrochilus distichus	Eryuan	2100	13.93	745.16	68.27	1210.94	1.63	Epiphyte	Dense forest	Non-endemic
Gastrochilus distichus	Dali	2200	13.62	1082.70	68.61	1256.27	1.16	Epiphyte	Dense forest	Non-endemic
Gastrochilus distichus	Yongde	2326	13.17	1266.25	69.00	1283.63	1.01	Epiphyte	Dense forest	Non-endemic
Gastrochilus distichus	Dali	2350	12.72	1082.70	68.61	1256.27	1.16	Epiphyte	Dense forest	Non-endemic
Gastrochilus distichus	Longling	2400	9.72	2098.66	84.65	973.45	0.46	Epiphyte	Dense forest	Non-endemic
Gastrochilus distichus	Jingdong	2500	10.58	1128.40	76.80	1142.11	1.01	Epiphyte	Dense forest	Non-endemic
Gastrochilus distichus	Yongde	2600	11.53	1266.25	69.00	1283.63	1.01	Epiphyte	Dense forest	Non-endemic
Gastrochilus distichus	Zhenkang	2700	8.64	1602.96	81.14	1089.09	0.68	Epiphyte	Dense forest	Non-endemic
Gastrochilus distichus	Dali	2700	10.62	1082.70	68.61	1256.27	1.16	Epiphyte	Dense forest	Non-endemic
Gastrochilus distichus	Yangbi	2800	9.19	1044.23	72.19	1161.15	1.11	Epiphyte	Dense forest	Non-endemic
Gastrochilus distichus	Dali	1500	17.82	1082.70	68.61	1256.27	1.16	Epiphyte	Sparse forest	Non-endemic
Gastrochilus intermedius	Lancang	1500	16.70	1596.50	77.88	1183.80	0.74	Epiphyte	Dense forest	Non-endemic
Gastrochilus obliquus	Mengla	500	22.20	1514.70	84.25	1146.68	0.76	Epiphyte	Dense forest	Non-endemic
Gastrochilus obliquus	Mengla	600	21.60	1514.70	84.25	1146.68	0.76	Epiphyte	Dense forest	Non-endemic
Gastrochilus obliquus	Mengla	850	20.10	1514.70	84.25	1146.68	0.76	Epiphyte	Sparse forest	Non-endemic
Gastrochilus obliquus	Jinghong	860	20.56	1161.08	80.46	1256.19	1.08	Epiphyte	Dense forest	Non-endemic
Gastrochilus obliquus	Jinghong	860	20.56	1161.08	80.46	1256.19	1.08	Epiphyte	Dense forest	Non-endemic
Gastrochilus obliquus	Jinghong	950	20.02	1161.08	80.46	1256.19	1.08	Epiphyte	Shrubland	Non-endemic
Gastrochilus pseudodistichus	Xichou	1000	18.93	1267.54	82.98	985.97	0.78	Epiphyte	Dense forest	Non-endemic
Gastrochilus pseudodistichus	Malipo	1100	17.66	1063.54	85.83	1053.97	0.99	Epiphyte	Sparse forest	Non-endemic
Gastrochilus pseudodistichus	Xichou	1350	16.83	1267.54	82.98	985.97	0.78	Epiphyte	Dense forest	Non-endemic
Gastrochilus pseudodistichus	Xichou	1500	15.93	1267.54	82.98	985.97	0.78	Epiphyte	Dense forest	Non-endemic
Gastrochilus pseudodistichus	Malipo	1500	15.26	1063.54	85.83	1053.97	0.99	Epiphyte	Shrubland	Non-endemic
Gastrochilus pseudodistichus	Malipo	1600	14.66	1063.54	85.83	1053.97	0.99	Epiphyte	Dense forest	Non-endemic
Gastrochilus pseudodistichus	Pingbian	1800	14.02	1648.57	86.34	990.16	0.60	Epiphyte	Dense forest	Non-endemic
Gastrochilus pseudodistichus	Lancang	2200	12.50	1596.50	77.88	1183.80	0.74	Epiphyte	Dense forest	Non-endemic
Gastrochilus pseudodistichus	Pingbian	2200	11.62	1648.57	86.34	990.16	0.60	Epiphyte	Dense forest	Non-endemic
Gastrochilus pseudodistichus	Gongshan	2500	9.08	1738.42	78.47	860.43	0.50	Epiphyte	Dense forest	Non-endemic
Gastrochilus subpapillosus	Menghai	1180	18.48	1314.38	80.83	1150.91	0.88	Epiphyte	Dense forest	Endemic
Gastrochilus subpapillosus	Jinghong	1400	17.32	1161.08	80.46	1256.19	1.08	Epiphyte	Dense forest	Endemic
Gastrodia albiflora	Songming	1900	14.19	1010.18	74.14	1134.24	1.12	saprophyte	Dense forest	Non-endemic
Gastrodia elata	Shiping	1700	16.36	919.48	75.04	1244.05	1.35	saprophyte	Shrubland	Non-endemic

Gastrodia elata	Daguan	1950	10.12	987.00	80.05	896.37	0.91	saprophyte	Dense forest	Non-endemic
Gastrodia elata	Yiliang	1950	10.58	751.45	71.67	1097.64	1.46	saprophyte	Shrubland	Non-endemic
Gastrodia elata	Weixi	2020	13.31	970.70	69.95	1021.03	1.05	saprophyte	Dense forest	Non-endemic
Gastrodia elata	Yiliang	2100	9.68	751.45	71.67	1097.64	1.46	saprophyte	Dense forest	Non-endemic
Gastrodia elata	Dali	2300	13.02	1082.70	68.61	1256.27	1.16	saprophyte	Dense forest	Non-endemic
Gastrodia elata	Lijiang	2400	12.67	982.53	63.24	1077.65	1.10	saprophyte	Dense forest	Non-endemic
Gastrodia elata	Zhongdian	2900	8.04	641.73	69.05	914.89	1.43	saprophyte	Grassy slope	Non-endemic
Gastrodia elata	Lijiang	3000	9.07	982.53	63.24	1077.65	1.10	saprophyte	Open site	Non-endemic
Gastrodia elata	Zhongdian	3100	6.84	641.73	69.05	914.89	1.43	saprophyte	Dense forest	Non-endemic
Gastrodia menghaiensis	Menghai	1200	18.36	1314.38	80.83	1150.91	0.88	saprophyte	Dense forest	Endemic
Gastrodia tuberculata	Kunming	1900	14.88	1019.14	72.30	1197.62	1.18	saprophyte	Shrubland	Endemic
Geodorum attenuatum	Menghai	1350	17.46	1314.38	80.83	1150.91	0.88	Terrestrial	Dense forest	Non-endemic
Geodorum densiflorum	Yuanyang	400	23.91	1100.28	77.18	1242.17	1.13	Terrestrial	Dense forest	Non-endemic
Geodorum densiflorum	Yuanjiang	650	22.28	805.59	69.08	1652.32	2.05	Terrestrial	Grassy slope	Non-endemic
Geodorum densiflorum	Heqing	2000	14.79	977.00	65.16	1211.84	1.24	Terrestrial	Grassy slope	Non-endemic
Geodorum pulchellum	Pingbian	400	22.42	1648.57	86.34	990.16	0.60	Terrestrial	Grassy slope	Non-endemic
Geodorum recurvum	Shiping	1300	18.76	919.48	75.04	1244.05	1.35	Terrestrial	Shrubland	Non-endemic
Goodyera biflora	Wenshan	1500	16.66	988.87	76.70	1272.98	1.29	Epiphyte	Dense forest	Non-endemic
Goodyera biflora	Wenshan	2100	13.06	988.87	76.70	1272.98	1.29	Epiphyte	Dense forest	Non-endemic
Goodyera brachystegia	Wenshan	1469	16.85	988.87	76.70	1272.98	1.29	Terrestrial	Dense forest	Non-endemic
Goodyera foliosa	Gongshan	1300	16.28	1738.42	78.47	860.43	0.50	Terrestrial	Dense forest	Non-endemic
Goodyera foliosa	Gongshan	1720	13.76	1738.42	78.47	860.43	0.50	Terrestrial	Dense forest	Non-endemic
Goodyera foliosa	Malipo	1800	13.46	1063.54	85.83	1053.97	0.99	Terrestrial	Dense forest	Non-endemic
Goodyera foliosa	Fugong	2000	12.06	1441.43	79.99	906.18	0.63	Terrestrial	Dense forest	Non-endemic
Goodyera foliosa	Jingdong	2100	12.98	1128.40	76.80	1142.11	1.01	Terrestrial	Dense forest	Non-endemic
Goodyera foliosa	Fugong	2500	9.06	1441.43	79.99	906.18	0.63	Terrestrial	Dense forest	Non-endemic
Goodyera foliosa	Luchun	2600	11.10	2013.54	78.64	1151.56	0.57	Terrestrial	Dense forest	Non-endemic
Goodyera foliosa	Wuding	2600	9.79	988.68	74.74	1188.19	1.20	Terrestrial	Dense forest	Non-endemic
Goodyera foliosa	Fugong	2800	7.26	1441.43	79.99	906.18	0.63	Terrestrial	Dense forest	Non-endemic
Goodyera foliosa	Fugong	2800	7.26	1441.43	79.99	906.18	0.63	Terrestrial	Dense forest	Non-endemic
Goodyera foliosa	Yongde	2820	10.21	1266.25	69.00	1283.63	1.01	Terrestrial	Grassy slope	Non-endemic
Goodyera foliosa	Yongde	2900	9.73	1266.25	69.00	1283.63	1.01	Terrestrial	Dense forest	Non-endemic
Goodyera foliosa	Yongde	3100	8.53	1266.25	69.00	1283.63	1.01	Terrestrial	Meadow	Non-endemic
Goodyera foliosa	Yongde	3200	7.93	1266.25	69.00	1283.63	1.01	Terrestrial	Meadow	Non-endemic
Goodyera fumata	Jinghong	750	21.22	1161.08	80.46	1256.19	1.08	Terrestrial	Shrubland	Non-endemic
Goodyera fumata	Jinghong	900	20.32	1161.08	80.46	1256.19	1.08	Terrestrial	Dense forest	Non-endemic

Goodyera fusca	Eryuan	3780	3.85	745.16	68.27	1210.94	1.63	Terrestrial	Shrubland	Non-endemic
Goodyera fusca	Deqin	4500	-1.83	639.48	70.85	896.66	1.40	Terrestrial	Shrubland	Non-endemic
Goodyera henryi	Guangnan	1550	14.95	1044.26	78.94	1090.35	1.04	Terrestrial	Grassy slope	Non-endemic
Goodyera henryi	Xichou	1800	14.13	1267.54	82.98	985.97	0.78	Terrestrial	Dense forest	Non-endemic
Goodyera henryi	Jinghong	1800	14.92	1161.08	80.46	1256.19	1.08	Terrestrial	Forest edge	Non-endemic
Goodyera henryi	Yongshan	1850	10.60	665.40	74.22	968.77	1.46	Terrestrial	Dense forest	Non-endemic
Goodyera henryi	Yongde	2000	15.13	1266.25	69.00	1283.63	1.01	Terrestrial	Dense forest	Non-endemic
Goodyera henryi	Wenshan	2300	11.86	988.87	76.70	1272.98	1.29	Terrestrial	Dense forest	Non-endemic
Goodyera henryi	Fugong	2400	9.66	1441.43	79.99	906.18	0.63	Terrestrial	Dense forest	Non-endemic
Goodyera henryi	Lushui	2500	11.05	1195.57	70.63	911.65	0.76	Terrestrial	Dense forest	Non-endemic
Goodyera procera	Funing	500	20.62	1161.58	79.07	1147.17	0.99	Terrestrial	Dense forest	Non-endemic
Goodyera procera	Funing	550	20.32	1161.58	79.07	1147.17	0.99	Terrestrial	Forest edge	Non-endemic
Goodyera procera	Pingbian	680	20.74	1648.57	86.34	990.16	0.60	Terrestrial	Dense forest	Non-endemic
Goodyera procera	Jinping	800	20.69	2305.17	83.81	1030.46	0.45	Terrestrial	Dense forest	Non-endemic
Goodyera procera	Lushui	900	20.65	1195.57	70.63	911.65	0.76	Terrestrial	Dense forest	Non-endemic
Goodyera procera	Jinghong	1100	19.12	1161.08	80.46	1256.19	1.08	Terrestrial	Dense forest	Non-endemic
Goodyera procera	Wenshan	1350	17.56	988.87	76.70	1272.98	1.29	Terrestrial	Grassy slope	Non-endemic
Goodyera procera	Pingbian	1460	16.06	1648.57	86.34	990.16	0.60	Terrestrial	Grassy slope	Non-endemic
Goodyera procera	Lancang	1550	16.40	1596.50	77.88	1183.80	0.74	Terrestrial	Grassy slope	Non-endemic
Goodyera procera	Pingbian	1730	14.44	1648.57	86.34	990.16	0.60	Terrestrial	Dense forest	Non-endemic
Goodyera procera	Yongde	2000	15.13	1266.25	69.00	1283.63	1.01	Terrestrial	Dense forest	Non-endemic
Goodyera repens	Deqin	3000	7.17	639.48	70.85	896.66	1.40	Epiphyte	Sparse forest	Non-endemic
Goodyera repens	Dongchuan	2850	8.73	1021.73	71.65	1186.61	1.16	Epiphyte	Dense forest	Non-endemic
Goodyera repens	Yongde	2900	9.73	1266.25	69.00	1283.63	1.01	Epiphyte	Shrubland	Non-endemic
Goodyera repens	Dongchuan	3200	6.63	1021.73	71.65	1186.61	1.16	Epiphyte	Dense forest	Non-endemic
Goodyera repens	Dongchuan	3300	6.03	1021.73	71.65	1186.61	1.16	Epiphyte	Shrubland	Non-endemic
Goodyera repens	Jingdong	2160	12.62	1128.40	76.80	1142.11	1.01	Terrestrial	Dense forest	Non-endemic
Goodyera repens	Gongshan	2500	9.08	1738.42	78.47	860.43	0.50	Terrestrial	Dense forest	Non-endemic
Goodyera repens	Dali	2700	10.62	1082.70	68.61	1256.27	1.16	Terrestrial	Dense forest	Non-endemic
Goodyera repens	Fugong	2900	6.66	1441.43	79.99	906.18	0.63	Terrestrial	Dense forest	Non-endemic
Goodyera repens	Yangbi	2900	8.59	1044.23	72.19	1161.15	1.11	Terrestrial	Dense forest	Non-endemic
Goodyera repens	Dongchuan	2940	8.19	1021.73	71.65	1186.61	1.16	Terrestrial	Dense forest	Non-endemic
Goodyera repens	Yongde	2950	9.43	1266.25	69.00	1283.63	1.01	Terrestrial	Dense forest	Non-endemic
Goodyera repens	Yunlong	2980	8.17	1195.57	70.63	911.65	0.76	Terrestrial	Dense forest	Non-endemic
Goodyera repens	Yunlong	3000	8.05	1195.57	70.63	911.65	0.76	Terrestrial	Dense forest	Non-endemic
Goodyera repens	Zhongdian	3100	6.84	641.73	69.05	914.89	1.43	Terrestrial	Dense forest	Non-endemic

Goodyera repens	Luquan	3190	6.58	965.09	73.60	1160.89	1.20	Terrestrial	Shrubland	Non-endemic
Goodyera repens	Weixi	3300	5.63	970.70	69.95	1021.03	1.05	Terrestrial	Dense forest	Non-endemic
Goodyera repens	Zhongdian	3500	4.44	641.73	69.05	914.89	1.43	Terrestrial	Dense forest	Non-endemic
Goodyera repens	Deqin	3800	2.37	639.48	70.85	896.66	1.40	Terrestrial	Dense forest	Non-endemic
Goodyera revutina	Wenshan	1870	14.44	988.87	76.70	1272.98	1.29	Terrestrial	Dense forest	Non-endemic
Goodyera robusta	Luchun	2100	14.10	2013.54	78.64	1151.56	0.57	Terrestrial	Dense forest	Non-endemic
Goodyera schlechtendaliana	Xichou	1450	16.23	1267.54	82.98	985.97	0.78	Terrestrial	Dense forest	Non-endemic
Goodyera schlechtendaliana	Zhenxiong	1850	10.34	899.34	83.70	852.28	0.95	Terrestrial	Dense forest	Non-endemic
Goodyera schlechtendaliana	Gongshan	2000	12.08	1738.42	78.47	860.43	0.50	Terrestrial	Forest edge	Non-endemic
Goodyera schlechtendaliana	Pingbian	2100	12.22	1648.57	86.34	990.16	0.60	Terrestrial	Sparse forest	Non-endemic
Goodyera schlechtendaliana	Deqin	2300	11.37	639.48	70.85	896.66	1.40	Terrestrial	Shrubland	Non-endemic
Goodyera schlechtendaliana	Lijiang	2700	10.87	982.53	63.24	1077.65	1.10	Terrestrial	Dense forest	Non-endemic
Goodyera schlechtendaliana	Jingdong	2700	9.38	1128.40	76.80	1142.11	1.01	Terrestrial	Grassy slope	Non-endemic
Goodyera schlechtendaliana	Yangbi	2800	9.19	1044.23	72.19	1161.15	1.11	Terrestrial	Dense forest	Non-endemic
Goodyera schlechtendaliana	Jingdong	2830	8.60	1128.40	76.80	1142.11	1.01	Terrestrial	Dense forest	Non-endemic
Goodyera velutina	Yiliang	1850	11.18	751.45	71.67	1097.64	1.46	Terrestrial	Dense forest	Non-endemic
Goodyera viridiflora	Yanshan	1100	18.86	1003.57	79.42	1172.21	1.17	Terrestrial	Dense forest	Non-endemic
Goodyera viridiflora	Yongde	1600	17.53	1266.25	69.00	1283.63	1.01	Terrestrial	Dense forest	Non-endemic
Goodyera viridiflora	Yongde	1600	17.53	1266.25	69.00	1283.63	1.01	Terrestrial	Forest edge	Non-endemic
Goodyera viridiflora	Yongde	3200	7.93	1266.25	69.00	1283.63	1.01	Terrestrial	Meadow	Non-endemic
Goodyera yunnanensis	Deqin	3800	2.37	639.48	70.85	896.66	1.40	Terrestrial	Dense forest	Non-endemic
Gymnadenia conopsea	Zhongdian	3400	5.04	641.73	69.05	914.89	1.43	Terrestrial	Dense forest	Non-endemic
Gymnadenia conopsea	Zhongdian	3930	1.86	641.73	69.05	914.89	1.43	Terrestrial	Dense forest	Non-endemic
Gymnadenia crassinervis	Gongshan	2000	12.08	1738.42	78.47	860.43	0.50	Terrestrial	Grassy slope	Non-endemic
Gymnadenia crassinervis	Weixi	3600	3.83	970.70	69.95	1021.03	1.05	Terrestrial	Grassy slope	Non-endemic
Gymnadenia orchidis	Heqing	2270	13.17	977.00	65.16	1211.84	1.24	Terrestrial	Meadow	Non-endemic
Gymnadenia orchidis	Lijiang	2650	11.17	982.53	63.24	1077.65	1.10	Terrestrial	Meadow	Non-endemic
Gymnadenia orchidis	Deqin	2700	8.97	639.48	70.85	896.66	1.40	Terrestrial	Grassy slope	Non-endemic
Gymnadenia orchidis	Lijiang	2700	10.87	982.53	63.24	1077.65	1.10	Terrestrial	Meadow	Non-endemic
Gymnadenia orchidis	Lijiang	2800	10.27	982.53	63.24	1077.65	1.10	Terrestrial	Dense forest	Non-endemic
Gymnadenia orchidis	Zhongdian	2800	8.64	641.73	69.05	914.89	1.43	Terrestrial	Grassy slope	Non-endemic
Gymnadenia orchidis	Deqin	3000	7.17	639.48	70.85	896.66	1.40	Terrestrial	Dense forest	Non-endemic
Gymnadenia orchidis	Zhongdian	3000	7.44	641.73	69.05	914.89	1.43	Terrestrial	Grassy slope	Non-endemic
Gymnadenia orchidis	Lijiang	3000	9.07	982.53	63.24	1077.65	1.10	Terrestrial	Grassy slope	Non-endemic
Gymnadenia orchidis	Zhongdian	3100	6.84	641.73	69.05	914.89	1.43	Terrestrial	Grassy slope	Non-endemic
Gymnadenia orchidis	Lijiang	3140	8.23	982.53	63.24	1077.65	1.10	Terrestrial	Shrubland	Non-endemic

Gymnadenia orchidis	Deqin	3170	6.15	639.48	70.85	896.66	1.40	Terrestrial	Grassy slope	Non-endemic
Gymnadenia orchidis	Zhongdian	3200	6.24	641.73	69.05	914.89	1.43	Terrestrial	Dense forest	Non-endemic
Gymnadenia orchidis	Weixi	3200	6.23	970.70	69.95	1021.03	1.05	Terrestrial	Grassy slope	Non-endemic
Gymnadenia orchidis	Dali	3200	7.62	1082.70	68.61	1256.27	1.16	Terrestrial	Meadow	Non-endemic
Gymnadenia orchidis	Lijiang	3300	7.27	982.53	63.24	1077.65	1.10	Terrestrial	Dense forest	Non-endemic
Gymnadenia orchidis	Zhongdian	3400	5.04	641.73	69.05	914.89	1.43	Terrestrial	Dense forest	Non-endemic
Gymnadenia orchidis	Lijiang	3400	6.67	982.53	63.24	1077.65	1.10	Terrestrial	Meadow	Non-endemic
Gymnadenia orchidis	Weixi	3500	4.43	970.70	69.95	1021.03	1.05	Terrestrial	Forest edge	Non-endemic
Gymnadenia orchidis	Weixi	3500	4.43	970.70	69.95	1021.03	1.05	Terrestrial	Grassy slope	Non-endemic
Gymnadenia orchidis	Zhongdian	3500	4.44	641.73	69.05	914.89	1.43	Terrestrial	Meadow	Non-endemic
Gymnadenia orchidis	Fugong	3500	3.06	1441.43	79.99	906.18	0.63	Terrestrial	Meadow	Non-endemic
Gymnadenia orchidis	Gongshan	3600	2.48	1738.42	78.47	860.43	0.50	Terrestrial	Forest edge	Non-endemic
Gymnadenia orchidis	Weixi	3600	3.83	970.70	69.95	1021.03	1.05	Terrestrial	Grassy slope	Non-endemic
Gymnadenia orchidis	Lijiang	3650	5.17	982.53	63.24	1077.65	1.10	Terrestrial	Grassy slope	Non-endemic
Gymnadenia orchidis	Zhongdian	3700	3.24	641.73	69.05	914.89	1.43	Terrestrial	Meadow	Non-endemic
Gymnadenia orchidis	Zhongdian	3700	3.24	641.73	69.05	914.89	1.43	Terrestrial	Shrubland	Non-endemic
Gymnadenia orchidis	Zhongdian	3700	3.24	641.73	69.05	914.89	1.43	Terrestrial	Shrubland	Non-endemic
Gymnadenia orchidis	Deqin	3800	2.37	639.48	70.85	896.66	1.40	Terrestrial	Grassy slope	Non-endemic
Gymnadenia orchidis	Zhongdian	3800	2.64	641.73	69.05	914.89	1.43	Terrestrial	Meadow	Non-endemic
Gymnadenia orchidis	Deqin	3800	2.37	639.48	70.85	896.66	1.40	Terrestrial	Meadow	Non-endemic
Gymnadenia orchidis	Lijiang	3800	4.27	982.53	63.24	1077.65	1.10	Terrestrial	Open site	Non-endemic
Gymnadenia orchidis	Zhongdian	3800	2.64	641.73	69.05	914.89	1.43	Terrestrial	Shrubland	Non-endemic
Gymnadenia orchidis	Zhongdian	3800	2.64	641.73	69.05	914.89	1.43	Terrestrial	Shrubland	Non-endemic
Gymnadenia orchidis	Zhongdian	3850	2.34	641.73	69.05	914.89	1.43	Terrestrial	Meadow	Non-endemic
Gymnadenia orchidis	Weixi	3900	2.03	970.70	69.95	1021.03	1.05	Terrestrial	Dense forest	Non-endemic
Gymnadenia orchidis	Deqin	3900	1.77	639.48	70.85	896.66	1.40	Terrestrial	Grassy slope	Non-endemic
Gymnadenia orchidis	Lijiang	3900	3.67	982.53	63.24	1077.65	1.10	Terrestrial	Meadow	Non-endemic
Gymnadenia orchidis	Lijiang	3900	3.67	982.53	63.24	1077.65	1.10	Terrestrial	Open site	Non-endemic
Gymnadenia orchidis	Fugong	4000	0.06	1441.43	79.99	906.18	0.63	Terrestrial	Dense forest	Non-endemic
Gymnadenia orchidis	Weixi	4000	1.43	970.70	69.95	1021.03	1.05	Terrestrial	Grassy slope	Non-endemic
Gymnadenia orchidis	Zhongdian	4000	1.44	641.73	69.05	914.89	1.43	Terrestrial	Grassy slope	Non-endemic
Gymnadenia orchidis	Lijiang	4000	3.07	982.53	63.24	1077.65	1.10	Terrestrial	Meadow	Non-endemic
Gymnadenia orchidis	Fugong	4000	0.06	1441.43	79.99	906.18	0.63	Terrestrial	Shrubland	Non-endemic
Gymnadenia orchidis	Weixi	4000	1.43	970.70	69.95	1021.03	1.05	Terrestrial	Shrubland	Non-endemic
Gymnadenia orchidis	Gongshan	4100	-0.52	1738.42	78.47	860.43	0.50	Terrestrial	Dense forest	Non-endemic
Gymnadenia orchidis	Dongchuan	4150	0.93	1021.73	71.65	1186.61	1.16	Terrestrial	Meadow	Non-endemic

Habenaria acuifera	Yuanmou	1700	18.08	657.16	56.45	1629.23	2.48	Terrestrial	Shrubland	Non-endemic
Habenaria acuifera	Fengqing	1750	15.70	1352.80	73.24	1172.52	0.87	Terrestrial	Shrubland	Non-endemic
Habenaria acuifera	Yangbi	2000	13.99	1044.23	72.19	1161.15	1.11	Terrestrial	Shrubland	Non-endemic
Habenaria aitchisonii	Heqing	1550	17.49	977.00	65.16	1211.84	1.24	Terrestrial	Grassy slope	Non-endemic
Habenaria aitchisonii	Anning	2100	13.43	896.67	71.23	1167.91	1.30	Terrestrial	Shrubland	Non-endemic
Habenaria aitchisonii	Anning	2200	12.83	896.67	71.23	1167.91	1.30	Terrestrial	Dense forest	Non-endemic
Habenaria aitchisonii	Kunming	2400	11.88	1019.14	72.30	1197.62	1.18	Terrestrial	Open site	Non-endemic
Habenaria aitchisonii	Yangbi	2500	10.99	1044.23	72.19	1161.15	1.11	Terrestrial	Dense forest	Non-endemic
Habenaria aitchisonii	Zhongdian	2500	10.44	641.73	69.05	914.89	1.43	Terrestrial	Shrubland	Non-endemic
Habenaria aitchisonii	Heqing	2650	10.89	977.00	65.16	1211.84	1.24	Terrestrial	Dense forest	Non-endemic
Habenaria aitchisonii	Lijiang	2650	11.17	982.53	63.24	1077.65	1.10	Terrestrial	Dense forest	Non-endemic
Habenaria aitchisonii	Yongde	2660	11.17	1266.25	69.00	1283.63	1.01	Terrestrial	Shrubland	Non-endemic
Habenaria aitchisonii	Deqin	2800	8.37	639.48	70.85	896.66	1.40	Terrestrial	Dense forest	Non-endemic
Habenaria aitchisonii	Zhongdian	2800	8.64	641.73	69.05	914.89	1.43	Terrestrial	Grassy slope	Non-endemic
Habenaria aitchisonii	Dongchuan	3000	7.83	1021.73	71.65	1186.61	1.16	Terrestrial	Grassy slope	Non-endemic
Habenaria aitchisonii	Deqin	3000	7.17	639.48	70.85	896.66	1.40	Terrestrial	Shrubland	Non-endemic
Habenaria aitchisonii	Zhongdian	3050	7.14	641.73	69.05	914.89	1.43	Terrestrial	Dense forest	Non-endemic
Habenaria aitchisonii	Zhongdian	3200	6.24	641.73	69.05	914.89	1.43	Terrestrial	Shrubland	Non-endemic
Habenaria aitchisonii	Deqin	3300	5.37	639.48	70.85	896.66	1.40	Terrestrial	Dense forest	Non-endemic
Habenaria aitchisonii	Luquan	3358	5.57	965.09	73.60	1160.89	1.20	Terrestrial	Shrubland	Non-endemic
Habenaria aitchisonii	Lijiang	3400	6.67	982.53	63.24	1077.65	1.10	Terrestrial	Shrubland	Non-endemic
Habenaria aitchisonii	Lijiang	3550	5.77	982.53	63.24	1077.65	1.10	Terrestrial	Dense forest	Non-endemic
Habenaria aitchisonii	Lijiang	3700	4.87	982.53	63.24	1077.65	1.10	Terrestrial	Shrubland	Non-endemic
Habenaria aitchisonii	Heqing	3800	3.99	977.00	65.16	1211.84	1.24	Terrestrial	Grassy slope	Non-endemic
Habenaria aitchisonii	Heqing	3800	3.99	977.00	65.16	1211.84	1.24	Terrestrial	Grassy slope	Non-endemic
Habenaria aitchisonii	Heqing	3800	3.99	977.00	65.16	1211.84	1.24	Terrestrial	Grassy slope	Non-endemic
Habenaria aitchisonii	Dongchuan	4000	1.83	1021.73	71.65	1186.61	1.16	Terrestrial	Meadow	Non-endemic
Habenaria aitchisonii	Dongchuan	4000	1.83	1021.73	71.65	1186.61	1.16	Terrestrial	Meadow	Non-endemic
Habenaria balfouriana	Lijiang	3000	9.07	982.53	63.24	1077.65	1.10	Terrestrial	Grassy slope	Non-endemic
Habenaria balfouriana	Zhongdian	3000	7.44	641.73	69.05	914.89	1.43	Terrestrial	Meadow	Non-endemic
Habenaria balfouriana	Heqing	3100	8.19	977.00	65.16	1211.84	1.24	Terrestrial	Meadow	Non-endemic
Habenaria balfouriana	Zhongdian	3300	5.64	641.73	69.05	914.89	1.43	Terrestrial	Meadow	Non-endemic
Habenaria balfouriana	Lijiang	3400	6.67	982.53	63.24	1077.65	1.10	Terrestrial	Meadow	Non-endemic
Habenaria balfouriana	Lijiang	3500	6.07	982.53	63.24	1077.65	1.10	Terrestrial	Grassy slope	Non-endemic
Habenaria balfouriana	Weixi	3600	3.83	970.70	69.95	1021.03	1.05	Terrestrial	Grassy slope	Non-endemic
Habenaria balfouriana	Zhongdian	3700	3.24	641.73	69.05	914.89	1.43	Terrestrial	Dense forest	Non-endemic

Habenaria ciliolaris	Mengla	550	21.90	1514.70	84.25	1146.68	0.76	Terrestrial	Dense forest	Non-endemic
Habenaria ciliolaris	Jinghong	650	21.82	1161.08	80.46	1256.19	1.08	Terrestrial	Forest edge	Non-endemic
Habenaria ciliolaris	Mengla	700	21.00	1514.70	84.25	1146.68	0.76	Terrestrial	Sparse forest	Non-endemic
Habenaria ciliolaris	Mengla	720	20.88	1514.70	84.25	1146.68	0.76	Terrestrial	Dense forest	Non-endemic
Habenaria ciliolaris	Mengla	920	19.68	1514.70	84.25	1146.68	0.76	Terrestrial	Dense forest	Non-endemic
Habenaria ciliolaris	Jinghong	920	20.20	1161.08	80.46	1256.19	1.08	Terrestrial	Dense forest	Non-endemic
Habenaria ciliolaris	Jinghong	920	20.20	1161.08	80.46	1256.19	1.08	Terrestrial	Forest edge	Non-endemic
Habenaria ciliolaris	Jinghong	1000	19.72	1161.08	80.46	1256.19	1.08	Terrestrial	Dense forest	Non-endemic
Habenaria ciliolaris	Luquan	2025	13.57	965.09	73.60	1160.89	1.20	Terrestrial	Dense forest	Non-endemic
Habenaria davidii	Eryuan	1840	15.49	745.16	68.27	1210.94	1.63	Terrestrial	Grassy slope	Non-endemic
Habenaria davidii	Jiangchuan	1950	14.30	872.74	74.72	1181.99	1.35	Terrestrial	Shrubland	Non-endemic
Habenaria davidii	Kunming	1970	14.46	1019.14	72.30	1197.62	1.18	Terrestrial	Grassy slope	Non-endemic
Habenaria davidii	Tonghai	2000	14.61	891.56	72.93	1238.30	1.39	Terrestrial	Meadow	Non-endemic
Habenaria davidii	Kunming	2030	14.10	1019.14	72.30	1197.62	1.18	Terrestrial	Dense forest	Non-endemic
Habenaria davidii	Luoping	2100	11.44	1686.24	82.51	1040.03	0.62	Terrestrial	Grassy slope	Non-endemic
Habenaria davidii	Kunming	2100	13.68	1019.14	72.30	1197.62	1.18	Terrestrial	Meadow	Non-endemic
Habenaria davidii	Yongde	2250	13.63	1266.25	69.00	1283.63	1.01	Terrestrial	Grassy slope	Non-endemic
Habenaria davidii	Fengqing	2300	12.40	1352.80	73.24	1172.52	0.87	Terrestrial	Grassy slope	Non-endemic
Habenaria davidii	Yongde	2300	13.33	1266.25	69.00	1283.63	1.01	Terrestrial	Grassy slope	Non-endemic
Habenaria davidii	Yongde	2400	12.73	1266.25	69.00	1283.63	1.01	Terrestrial	Grassy slope	Non-endemic
Habenaria davidii	Zhongdian	2470	10.62	641.73	69.05	914.89	1.43	Terrestrial	Grassy slope	Non-endemic
Habenaria davidii	Lijiang	2500	12.07	982.53	63.24	1077.65	1.10	Terrestrial	Shrubland	Non-endemic
Habenaria davidii	Eryuan	2560	11.17	745.16	68.27	1210.94	1.63	Terrestrial	Grassy slope	Non-endemic
Habenaria davidii	Lijiang	2700	10.87	982.53	63.24	1077.65	1.10	Terrestrial	Grassy slope	Non-endemic
Habenaria davidii	Lijiang	2800	10.27	982.53	63.24	1077.65	1.10	Terrestrial	Grassy slope	Non-endemic
Habenaria davidii	Zhongdian	2900	8.04	641.73	69.05	914.89	1.43	Terrestrial	Meadow	Non-endemic
Habenaria davidii	Lijiang	3020	8.95	982.53	63.24	1077.65	1.10	Terrestrial	Open site	Non-endemic
Habenaria davidii	Luquan	3190	6.58	965.09	73.60	1160.89	1.20	Terrestrial	Shrubland	Non-endemic
Habenaria davidii	Weixi	3200	6.23	970.70	69.95	1021.03	1.05	Terrestrial	Grassy slope	Non-endemic
Habenaria davidii	Dongchuan	3250	6.33	1021.73	71.65	1186.61	1.16	Terrestrial	Shrubland	Non-endemic
Habenaria delavayi	Mengzi	1720	16.14	855.35	71.82	1434.56	1.68	Terrestrial	Meadow	Non-endemic
Habenaria delavayi	Dali	2010	14.76	1082.70	68.61	1256.27	1.16	Terrestrial	Grassy slope	Non-endemic
Habenaria delavayi	Binchuan	2100	14.10	573.87	62.93	1418.42	2.47	Terrestrial	Meadow	Non-endemic
Habenaria delavayi	Kunming	2200	13.08	1019.14	72.30	1197.62	1.18	Terrestrial	Dense forest	Non-endemic
Habenaria delavayi	Anning	2250	12.53	896.67	71.23	1167.91	1.30	Terrestrial	Shrubland	Non-endemic
Habenaria delavayi	Eryuan	2280	12.85	745.16	68.27	1210.94	1.63	Terrestrial	Sparse forest	Non-endemic

Habenaria delavayi	Kunming	2300	12.48	1019.14	72.30	1197.62	1.18	Terrestrial	Grassy slope	Non-endemic
Habenaria delavayi	Eryuan	2360	12.37	745.16	68.27	1210.94	1.63	Terrestrial	Dense forest	Non-endemic
Habenaria delavayi	Kunming	2400	11.88	1019.14	72.30	1197.62	1.18	Terrestrial	Grassy slope	Non-endemic
Habenaria delavayi	Lijiang	2420	12.55	982.53	63.24	1077.65	1.10	Terrestrial	Meadow	Non-endemic
Habenaria delavayi	Lijiang	2500	12.07	982.53	63.24	1077.65	1.10	Terrestrial	Shrubland	Non-endemic
Habenaria delavayi	Lijiang	2600	11.47	982.53	63.24	1077.65	1.10	Terrestrial	Meadow	Non-endemic
Habenaria delavayi	Lijiang	2690	10.93	982.53	63.24	1077.65	1.10	Terrestrial	Meadow	Non-endemic
Habenaria delavayi	Zhongdian	2800	8.64	641.73	69.05	914.89	1.43	Terrestrial	Dense forest	Non-endemic
Habenaria delavayi	Lijiang	2800	10.27	982.53	63.24	1077.65	1.10	Terrestrial	Grassy slope	Non-endemic
Habenaria delavayi	Eryuan	2900	9.13	745.16	68.27	1210.94	1.63	Terrestrial	Dense forest	Non-endemic
Habenaria delavayi	Lijiang	3080	8.59	982.53	63.24	1077.65	1.10	Terrestrial	Meadow	Non-endemic
Habenaria delavayi	Lijiang	3100	8.47	982.53	63.24	1077.65	1.10	Terrestrial	Grassy slope	Non-endemic
Habenaria dentata	Funing	250	22.12	1161.58	79.07	1147.17	0.99	Terrestrial	Grassy slope	Non-endemic
Habenaria dentata	Yuanjiang	800	21.38	805.59	69.08	1652.32	2.05	Terrestrial	Shrubland	Non-endemic
Habenaria dentata	Menghai	900	20.16	1314.38	80.83	1150.91	0.88	Terrestrial	Dense forest	Non-endemic
Habenaria dentata	Cangyuan	1200	18.02	1733.34	81.63	1060.82	0.61	Terrestrial	Dense forest	Non-endemic
Habenaria dentata	Yanshan	1200	18.26	1003.57	79.42	1172.21	1.17	Terrestrial	Dense forest	Non-endemic
Habenaria dentata	Gongshan	1200	16.88	1738.42	78.47	860.43	0.50	Terrestrial	Grassy slope	Non-endemic
Habenaria dentata	Pingbian	1250	17.32	1648.57	86.34	990.16	0.60	Terrestrial	Grassy slope	Non-endemic
Habenaria dentata	Luchun	1300	18.90	2013.54	78.64	1151.56	0.57	Terrestrial	Dense forest	Non-endemic
Habenaria dentata	Mengzi	1300	18.66	855.35	71.82	1434.56	1.68	Terrestrial	Grassy slope	Non-endemic
Habenaria dentata	Mengzi	1300	18.66	855.35	71.82	1434.56	1.68	Terrestrial	Shrubland	Non-endemic
Habenaria dentata	Mengla	1350	17.10	1514.70	84.25	1146.68	0.76	Terrestrial	Dense forest	Non-endemic
Habenaria dentata	Jingdong	1460	16.82	1128.40	76.80	1142.11	1.01	Terrestrial	Shrubland	Non-endemic
Habenaria dentata	Fugong	1500	15.06	1441.43	79.99	906.18	0.63	Terrestrial	Open site	Non-endemic
Habenaria dentata	Jingdong	1520	16.46	1128.40	76.80	1142.11	1.01	Terrestrial	Dense forest	Non-endemic
Habenaria dentata	Jingdong	1550	16.28	1128.40	76.80	1142.11	1.01	Terrestrial	Sparse forest	Non-endemic
Habenaria dentata	Fengqing	1600	16.60	1352.80	73.24	1172.52	0.87	Terrestrial	Grassy slope	Non-endemic
Habenaria dentata	Shuangbai	1660	16.92	942.53	72.21	1300.52	1.38	Terrestrial	Dense forest	Non-endemic
Habenaria dentata	Gongshan	1700	13.88	1738.42	78.47	860.43	0.50	Terrestrial	Dense forest	Non-endemic
Habenaria dentata	Jingdong	1700	15.38	1128.40	76.80	1142.11	1.01	Terrestrial	Grassy slope	Non-endemic
Habenaria dentata	Gongshan	1700	13.88	1738.42	78.47	860.43	0.50	Terrestrial	Grassy slope	Non-endemic
Habenaria dentata	Fugong	1700	13.86	1441.43	79.99	906.18	0.63	Terrestrial	Open site	Non-endemic
Habenaria dentata	Longchuan	1700	14.35	1604.50	80.46	1149.57	0.72	Terrestrial	Open site	Non-endemic
Habenaria dentata	Tengchong	1740	14.56	1501.45	78.06	857.93	0.57	Terrestrial	Grassy slope	Non-endemic
Habenaria dentata	Eshan	1890	13.87	938.39	78.62	1123.48	1.20	Terrestrial	Grassy slope	Non-endemic

Habenaria dentata	Kunming	1900	14.88	1019.14	72.30	1197.62	1.18	Terrestrial	Shrubland	Non-endemic
Habenaria dentata	Eryuan	2100	13.93	745.16	68.27	1210.94	1.63	Terrestrial	Grassy slope	Non-endemic
Habenaria dentata	Kunming	2130	13.50	1019.14	72.30	1197.62	1.18	Terrestrial	Grassy slope	Non-endemic
Habenaria dentata	Kunming	2200	13.08	1019.14	72.30	1197.62	1.18	Terrestrial	Open site	Non-endemic
Habenaria dentata	Gongshan	2300	10.28	1738.42	78.47	860.43	0.50	Terrestrial	Grassy slope	Non-endemic
Habenaria diceras	Deqin	3310	5.31	639.48	70.85	896.66	1.40	Terrestrial	Shrubland	Non-endemic
Habenaria diphylla	Menghai	1300	17.76	1314.38	80.83	1150.91	0.88	Terrestrial	Dense forest	Non-endemic
Habenaria diplonema	Lijiang	2800	10.27	982.53	63.24	1077.65	1.10	Terrestrial	Dense forest	Endemic
Habenaria finetiana	Kunming	1900	14.88	1019.14	72.30	1197.62	1.18	Terrestrial	Shrubland	Non-endemic
Habenaria finetiana	Lincang	2000	14.52	1165.84	72.08	1167.73	1.00	Terrestrial	Dense forest	Non-endemic
Habenaria finetiana	Eryuan	2100	13.93	745.16	68.27	1210.94	1.63	Terrestrial	Grassy slope	Non-endemic
Habenaria finetiana	Heqing	2200	13.59	977.00	65.16	1211.84	1.24	Terrestrial	Grassy slope	Non-endemic
Habenaria finetiana	Anning	2200	12.83	896.67	71.23	1167.91	1.30	Terrestrial	Shrubland	Non-endemic
Habenaria finetiana	Lijiang	2400	12.67	982.53	63.24	1077.65	1.10	Terrestrial	Open site	Non-endemic
Habenaria finetiana	Zhongdian	2500	10.44	641.73	69.05	914.89	1.43	Terrestrial	Grassy slope	Non-endemic
Habenaria finetiana	Zhongdian	2500	10.44	641.73	69.05	914.89	1.43	Terrestrial	Grassy slope	Non-endemic
Habenaria fordii	Mengla	900	19.80	1514.70	84.25	1146.68	0.76	Terrestrial	Dense forest	Non-endemic
Habenaria fordii	Yongde	1300	19.33	1266.25	69.00	1283.63	1.01	Terrestrial	Dense forest	Non-endemic
Habenaria furcifera	Yongde	1000	21.13	1266.25	69.00	1283.63	1.01	Terrestrial	Grassy slope	Non-endemic
Habenaria glaucifolia	Lijiang	2600	11.47	982.53	63.24	1077.65	1.10	Terrestrial	Meadow	Non-endemic
Habenaria glaucifolia	Zhongdian	2800	8.64	641.73	69.05	914.89	1.43	Terrestrial	Grassy slope	Non-endemic
Habenaria glaucifolia	Lijiang	2800	10.27	982.53	63.24	1077.65	1.10	Terrestrial	Meadow	Non-endemic
Habenaria glaucifolia	Eryuan	2900	9.13	745.16	68.27	1210.94	1.63	Terrestrial	Sparse forest	Non-endemic
Habenaria glaucifolia	Lijiang	2950	9.37	982.53	63.24	1077.65	1.10	Terrestrial	Shrubland	Non-endemic
Habenaria glaucifolia	Weixi	3000	7.43	970.70	69.95	1021.03	1.05	Terrestrial	Dense forest	Non-endemic
Habenaria glaucifolia	Eryuan	3000	8.53	745.16	68.27	1210.94	1.63	Terrestrial	Forest edge	Non-endemic
Habenaria glaucifolia	Zhongdian	3050	7.14	641.73	69.05	914.89	1.43	Terrestrial	Grassy slope	Non-endemic
Habenaria glaucifolia	Lijiang	3070	8.65	982.53	63.24	1077.65	1.10	Terrestrial	Meadow	Non-endemic
Habenaria glaucifolia	Zhongdian	3100	6.84	641.73	69.05	914.89	1.43	Terrestrial	Meadow	Non-endemic
Habenaria glaucifolia	Lijiang	3100	8.47	982.53	63.24	1077.65	1.10	Terrestrial	Meadow	Non-endemic
Habenaria glaucifolia	Zhongdian	3100	6.84	641.73	69.05	914.89	1.43	Terrestrial	Shrubland	Non-endemic
Habenaria glaucifolia	Weixi	3200	6.23	970.70	69.95	1021.03	1.05	Terrestrial	Grassy slope	Non-endemic
Habenaria glaucifolia	Zhongdian	3260	5.88	641.73	69.05	914.89	1.43	Terrestrial	Meadow	Non-endemic
Habenaria glaucifolia	Zhongdian	3300	5.64	641.73	69.05	914.89	1.43	Terrestrial	Meadow	Non-endemic
Habenaria glaucifolia	Zhongdian	3300	5.64	641.73	69.05	914.89	1.43	Terrestrial	Shrubland	Non-endemic
Habenaria glaucifolia	Deqin	3320	5.25	639.48	70.85	896.66	1.40	Terrestrial	Meadow	Non-endemic

Habenaria glaucifolia	Zhongdian	3600	3.84	641.73	69.05	914.89	1.43	Terrestrial	Meadow	Non-endemic
Habenaria glaucifolia	Zhongdian	3900	2.04	641.73	69.05	914.89	1.43	Terrestrial	Dense forest	Non-endemic
Habenaria intermedia	Zhongdian	3000	7.44	641.73	69.05	914.89	1.43	Terrestrial	Sparse forest	Non-endemic
Habenaria limprichtii	Heqing	1700	16.59	977.00	65.16	1211.84	1.24	Terrestrial	Grassy slope	Non-endemic
Habenaria limprichtii	Kunming	1900	14.88	1019.14	72.30	1197.62	1.18	Terrestrial	Shrubland	Non-endemic
Habenaria limprichtii	Kunming	1900	14.88	1019.14	72.30	1197.62	1.18	Terrestrial	Shrubland	Non-endemic
Habenaria limprichtii	Zhaotong	1920	11.80	708.66	74.85	1046.05	1.48	Terrestrial	Grassy slope	Non-endemic
Habenaria limprichtii	Jiangchuan	1950	14.30	872.74	74.72	1181.99	1.35	Terrestrial	Dense forest	Non-endemic
Habenaria limprichtii	Jingdong	2100	12.98	1128.40	76.80	1142.11	1.01	Terrestrial	Dense forest	Non-endemic
Habenaria limprichtii	Kunming	2100	13.68	1019.14	72.30	1197.62	1.18	Terrestrial	Grassy slope	Non-endemic
Habenaria limprichtii	Kunming	2300	12.48	1019.14	72.30	1197.62	1.18	Terrestrial	Grassy slope	Non-endemic
Habenaria limprichtii	Kunming	2310	12.42	1019.14	72.30	1197.62	1.18	Terrestrial	Grassy slope	Non-endemic
Habenaria limprichtii	Dali	2350	12.72	1082.70	68.61	1256.27	1.16	Terrestrial	Shrubland	Non-endemic
Habenaria limprichtii	Songming	2350	11.49	1010.18	74.14	1134.24	1.12	Terrestrial	Shrubland	Non-endemic
Habenaria limprichtii	Kunming	2380	12.00	1019.14	72.30	1197.62	1.18	Terrestrial	Grassy slope	Non-endemic
Habenaria limprichtii	Dali	2420	12.30	1082.70	68.61	1256.27	1.16	Terrestrial	Dense forest	Non-endemic
Habenaria limprichtii	Dali	2500	11.82	1082.70	68.61	1256.27	1.16	Terrestrial	Meadow	Non-endemic
Habenaria limprichtii	Wuding	2600	9.79	988.68	74.74	1188.19	1.20	Terrestrial	Grassy slope	Non-endemic
Habenaria limprichtii	Lijiang	2650	11.17	982.53	63.24	1077.65	1.10	Terrestrial	Grassy slope	Non-endemic
Habenaria limprichtii	Lijiang	2650	11.17	982.53	63.24	1077.65	1.10	Terrestrial	Meadow	Non-endemic
Habenaria limprichtii	Lijiang	2730	10.69	982.53	63.24	1077.65	1.10	Terrestrial	Meadow	Non-endemic
Habenaria limprichtii	Lijiang	2750	10.57	982.53	63.24	1077.65	1.10	Terrestrial	Meadow	Non-endemic
Habenaria limprichtii	Zhongdian	2900	8.04	641.73	69.05	914.89	1.43	Terrestrial	Meadow	Non-endemic
Habenaria limprichtii	Yangbi	3000	7.99	1044.23	72.19	1161.15	1.11	Terrestrial	Grassy slope	Non-endemic
Habenaria limprichtii	Lijiang	3000	9.07	982.53	63.24	1077.65	1.10	Terrestrial	Grassy slope	Non-endemic
Habenaria limprichtii	Lijiang	3000	9.07	982.53	63.24	1077.65	1.10	Terrestrial	Shrubland	Non-endemic
Habenaria limprichtii	Lijiang	3020	8.95	982.53	63.24	1077.65	1.10	Terrestrial	Open site	Non-endemic
Habenaria limprichtii	Lijiang	3200	7.87	982.53	63.24	1077.65	1.10	Terrestrial	Grassy slope	Non-endemic
Habenaria limprichtii	Lijiang	3430	6.49	982.53	63.24	1077.65	1.10	Terrestrial	Shrubland	Non-endemic
Habenaria linguella	Tonghai	1400	18.21	891.56	72.93	1238.30	1.39	Terrestrial	Open site	Non-endemic
Habenaria linguella	Menghai	1450	16.86	1314.38	80.83	1150.91	0.88	Terrestrial	Dense forest	Non-endemic
Habenaria linguella	Menglian	1600	15.98	1357.68	80.47	1185.94	0.87	Terrestrial	Meadow	Non-endemic
Habenaria linguella	Menghai	1800	14.76	1314.38	80.83	1150.91	0.88	Terrestrial	Meadow	Non-endemic
Habenaria linguella	Heqing	2590	11.25	977.00	65.16	1211.84	1.24	Terrestrial	Meadow	Non-endemic
Habenaria lucida	Jinghong	150	24.82	1161.08	80.46	1256.19	1.08	Terrestrial	Dense forest	Non-endemic
Habenaria lucida	Mengla	850	20.10	1514.70	84.25	1146.68	0.76	Terrestrial	Dense forest	Non-endemic

Habenaria lucida	Jinghong	900	20.32	1161.08	80.46	1256.19	1.08	Terrestrial	Shrubland	Non-endemic
Habenaria lucida	Jinghong	1000	19.72	1161.08	80.46	1256.19	1.08	Terrestrial	Dense forest	Non-endemic
Habenaria lucida	Jinghong	1000	19.72	1161.08	80.46	1256.19	1.08	Terrestrial	Dense forest	Non-endemic
Habenaria lucida	Jinghong	1100	19.12	1161.08	80.46	1256.19	1.08	Terrestrial	Dense forest	Non-endemic
Habenaria mairei	Zhongdian	3100	6.84	641.73	69.05	914.89	1.43	Terrestrial	Meadow	Non-endemic
Habenaria mairei	Zhongdian	3350	5.34	641.73	69.05	914.89	1.43	Terrestrial	Meadow	Non-endemic
Habenaria mairei	Zhongdian	3500	4.44	641.73	69.05	914.89	1.43	Terrestrial	Grassy slope	Non-endemic
Habenaria malintana	Mengla	560	21.84	1514.70	84.25	1146.68	0.76	Terrestrial	Open site	Non-endemic
Habenaria malintana	Mengla	800	20.40	1514.70	84.25	1146.68	0.76	Terrestrial	Shrubland	Non-endemic
Habenaria malintana	Mengla	850	20.10	1514.70	84.25	1146.68	0.76	Terrestrial	Dense forest	Non-endemic
Habenaria malintana	Mengla	900	19.80	1514.70	84.25	1146.68	0.76	Terrestrial	Dense forest	Non-endemic
Habenaria malintana	Mengla	1040	18.96	1514.70	84.25	1146.68	0.76	Terrestrial	Grassy slope	Non-endemic
Habenaria malintana	Tengchong	1320	17.08	1501.45	78.06	857.93	0.57	Terrestrial	Dense forest	Non-endemic
Habenaria malintana	Tengchong	1320	17.08	1501.45	78.06	857.93	0.57	Terrestrial	Dense forest	Non-endemic
Habenaria marginata	Mengla	500	22.20	1514.70	84.25	1146.68	0.76	Terrestrial	Grassy slope	Non-endemic
Habenaria marginata	Jinghong	800	20.92	1161.08	80.46	1256.19	1.08	Terrestrial	Meadow	Non-endemic
Habenaria marginata	Jinghong	950	20.02	1161.08	80.46	1256.19	1.08	Terrestrial	Dense forest	Non-endemic
Habenaria marginata	Jinghong	1000	19.72	1161.08	80.46	1256.19	1.08	Terrestrial	Open site	Non-endemic
Habenaria marginata	Mengla	1040	18.96	1514.70	84.25	1146.68	0.76	Terrestrial	Grassy slope	Non-endemic
Habenaria marginata	Mengla	1200	18.00	1514.70	84.25	1146.68	0.76	Terrestrial	Open site	Non-endemic
Habenaria medioflexa	Mengla	700	21.00	1514.70	84.25	1146.68	0.76	Terrestrial	Dense forest	Non-endemic
Habenaria medioflexa	Mengla	800	20.40	1514.70	84.25	1146.68	0.76	Terrestrial	Dense forest	Non-endemic
Habenaria nematocerata	Mengla	1000	19.20	1514.70	84.25	1146.68	0.76	Terrestrial	Dense forest	Endemic
Habenaria petelotii	Xichou	1500	15.93	1267.54	82.98	985.97	0.78	Terrestrial	Dense forest	Non-endemic
Habenaria petelotii	Malipo	1600	14.66	1063.54	85.83	1053.97	0.99	Terrestrial	Dense forest	Non-endemic
Habenaria plurifoliata	Jinghong	800	20.92	1161.08	80.46	1256.19	1.08	Terrestrial	Dense forest	Non-endemic
Habenaria remiformis	Yuliang	1960	13.76	905.45	75.03	1218.48	1.35	Terrestrial	Dense forest	Non-endemic
Habenaria rostellifera	Yuliang	1800	14.72	905.45	75.03	1218.48	1.35	Terrestrial	Grassy slope	Non-endemic
Habenaria rostellifera	Kunming	1900	14.88	1019.14	72.30	1197.62	1.18	Terrestrial	Grassy slope	Non-endemic
Habenaria rostellifera	Kunming	2000	14.28	1019.14	72.30	1197.62	1.18	Terrestrial	Open site	Non-endemic
Habenaria rostellifera	Kunming	2050	13.98	1019.14	72.30	1197.62	1.18	Terrestrial	Grassy slope	Non-endemic
Habenaria rostellifera	Kunming	2100	13.68	1019.14	72.30	1197.62	1.18	Terrestrial	Grassy slope	Non-endemic
Habenaria rostellifera	Dali	2170	13.80	1082.70	68.61	1256.27	1.16	Terrestrial	Grassy slope	Non-endemic
Habenaria rostellifera	Songming	2200	12.39	1010.18	74.14	1134.24	1.12	Terrestrial	Dense forest	Non-endemic
Habenaria rostellifera	Kunming	2200	13.08	1019.14	72.30	1197.62	1.18	Terrestrial	Open site	Non-endemic
Habenaria rostellifera	Kunming	2300	12.48	1019.14	72.30	1197.62	1.18	Terrestrial	Dense forest	Non-endemic

Habenaria rostellifera	Kunming	2380	12.00	1019.14	72.30	1197.62	1.18	Terrestrial	Grassy slope	Non-endemic
Habenaria schindleri	Jingdong	2120	12.86	1128.40	76.80	1142.11	1.01	Terrestrial	Dense forest	Non-endemic
Habenaria szechuanica	Lijiang	2900	9.67	982.53	63.24	1077.65	1.10	Terrestrial	Dense forest	Non-endemic
Habenaria tonkinensis	Jinghong	840	20.68	1161.08	80.46	1256.19	1.08	Terrestrial	Dense forest	Non-endemic
Habenaria yuana	Jinghong	1800	14.92	1161.08	80.46	1256.19	1.08	Terrestrial	Grassy slope	Non-endemic
Hancockia uniflora	Xichou	1300	17.13	1267.54	82.98	985.97	0.78	Terrestrial	Dense forest	Non-endemic
Hancockia uniflora	Pingbian	1560	15.46	1648.57	86.34	990.16	0.60	Terrestrial	Sparse forest	Non-endemic
Hancockia uniflora	Pingbian	1650	14.92	1648.57	86.34	990.16	0.60	Terrestrial	Dense forest	Non-endemic
Hemipilia cruciata	Jianchuan	2200	12.35	753.13	70.16	1154.48	1.53	Terrestrial	Grassy slope	Non-endemic
Hemipilia cruciata	Yaoan	2400	12.14	784.85	68.90	1295.94	1.65	Terrestrial	Shrubland	Non-endemic
Hemipilia cruciata	Lijiang	2700	10.87	982.53	63.24	1077.65	1.10	Terrestrial	Grassy slope	Non-endemic
Hemipilia flabellata	Jiangchuan	1760	15.44	872.74	74.72	1181.99	1.35	Terrestrial	Dense forest	Non-endemic
Hemipilia flabellata	Jiangchuan	1780	15.32	872.74	74.72	1181.99	1.35	Terrestrial	Dense forest	Non-endemic
Hemipilia flabellata	Tonghai	1850	15.51	891.56	72.93	1238.30	1.39	Terrestrial	Dense forest	Non-endemic
Hemipilia flabellata	Anning	1860	14.87	896.67	71.23	1167.91	1.30	Terrestrial	Dense forest	Non-endemic
Hemipilia flabellata	Dali	2040	14.58	1082.70	68.61	1256.27	1.16	Terrestrial	Shrubland	Non-endemic
Hemipilia flabellata	Yangbi	2100	13.39	1044.23	72.19	1161.15	1.11	Terrestrial	Grassy slope	Non-endemic
Hemipilia flabellata	Eryuan	2120	13.81	745.16	68.27	1210.94	1.63	Terrestrial	Shrubland	Non-endemic
Hemipilia flabellata	Lijiang	2200	13.87	982.53	63.24	1077.65	1.10	Terrestrial	Dense forest	Non-endemic
Hemipilia flabellata	Zhongdian	2200	12.24	641.73	69.05	914.89	1.43	Terrestrial	Dense forest	Non-endemic
Hemipilia flabellata	Kunming	2200	13.08	1019.14	72.30	1197.62	1.18	Terrestrial	Open site	Non-endemic
Hemipilia flabellata	Dali	2230	13.44	1082.70	68.61	1256.27	1.16	Terrestrial	Shrubland	Non-endemic
Hemipilia flabellata	Binchuan	2300	12.90	573.87	62.93	1418.42	2.47	Terrestrial	Dense forest	Non-endemic
Hemipilia flabellata	Longling	2400	9.72	2098.66	84.65	973.45	0.46	Terrestrial	Dense forest	Non-endemic
Hemipilia flabellata	Wuding	2400	10.99	988.68	74.74	1188.19	1.20	Terrestrial	Dense forest	Non-endemic
Hemipilia flabellata	Lijiang	2400	12.67	982.53	63.24	1077.65	1.10	Terrestrial	Dense forest	Non-endemic
Hemipilia flabellata	Heqing	2480	11.91	977.00	65.16	1211.84	1.24	Terrestrial	Shrubland	Non-endemic
Hemipilia flabellata	Weixi	2500	10.43	970.70	69.95	1021.03	1.05	Terrestrial	Dense forest	Non-endemic
Hemipilia flabellata	Heqing	2500	11.79	977.00	65.16	1211.84	1.24	Terrestrial	Dense forest	Non-endemic
Hemipilia flabellata	Lijiang	2500	12.07	982.53	63.24	1077.65	1.10	Terrestrial	Shrubland	Non-endemic
Hemipilia flabellata	Gongshan	2700	7.88	1738.42	78.47	860.43	0.50	Terrestrial	Dense forest	Non-endemic
Hemipilia flabellata	Zhongdian	2700	9.24	641.73	69.05	914.89	1.43	Terrestrial	Shrubland	Non-endemic
Hemipilia flabellata	Zhongdian	2800	8.64	641.73	69.05	914.89	1.43	Terrestrial	Dense forest	Non-endemic
Hemipilia flabellata	Zhongdian	2900	8.04	641.73	69.05	914.89	1.43	Terrestrial	Dense forest	Non-endemic
Hemipilia flabellata	Zhongdian	3900	2.04	641.73	69.05	914.89	1.43	Terrestrial	Dense forest	Non-endemic
Hemipilia forrestii	Lijiang	2250	13.57	982.53	63.24	1077.65	1.10	Terrestrial	Dense forest	Non-endemic

Hemipilia kwangsiensis	Yongde	2300	13.33	1266.25	69.00	1283.63	1.01	Terrestrial	Dense forest	Non-endemic
Hemipilia limprichtii	Chuxiong	1500	17.57	874.28	69.99	1192.36	1.36	Terrestrial	Shrubland	Non-endemic
Hemipilia limprichtii	Dali	2050	14.52	1082.70	68.61	1256.27	1.16	Terrestrial	Shrubland	Non-endemic
Hemipilia limprichtii	Fuyuan	2500	9.87	1084.02	74.80	1096.10	1.01	Terrestrial	Shrubland	Non-endemic
Hemipilia quinquangularis	Weixi	1600	15.83	970.70	69.95	1021.03	1.05	Terrestrial	Dense forest	Non-endemic
Hemipilia quinquangularis	Deqin	1950	13.47	639.48	70.85	896.66	1.40	Terrestrial	Grassy slope	Non-endemic
Hemipilia quinquangularis	Weixi	2300	11.63	970.70	69.95	1021.03	1.05	Terrestrial	Dense forest	Non-endemic
Hemipilia quinquangularis	Gongshan	2500	9.08	1738.42	78.47	860.43	0.50	Terrestrial	Shrubland	Non-endemic
Hemipilia quinquangularis	Lijiang	2650	11.17	982.53	63.24	1077.65	1.10	Terrestrial	Dense forest	Non-endemic
Hemipilia quinquangularis	Deqin	2900	7.77	639.48	70.85	896.66	1.40	Terrestrial	Dense forest	Non-endemic
Herminium alaschanicum	Lijiang	2800	10.27	982.53	63.24	1077.65	1.10	Terrestrial	Grassy slope	Non-endemic
Herminium alaschanicum	Yongde	1700	16.93	1266.25	69.00	1283.63	1.01	Terrestrial	Dense forest	Non-endemic
Herminium alaschanicum	Deqin	2700	8.97	639.48	70.85	896.66	1.40	Terrestrial	Grassy slope	Non-endemic
Herminium alaschanicum	Zhongdian	2900	8.04	641.73	69.05	914.89	1.43	Terrestrial	Grassy slope	Non-endemic
Herminium alaschanicum	Zhongdian	3300	5.64	641.73	69.05	914.89	1.43	Terrestrial	Meadow	Non-endemic
Herminium alaschanicum	Zhongdian	3380	5.16	641.73	69.05	914.89	1.43	Terrestrial	Meadow	Non-endemic
Herminium angustilabre	Gongshan	3500	3.08	1738.42	78.47	860.43	0.50	Terrestrial	Shrubland	Non-endemic
Herminium carnosilabre	Gongshan	3200	4.88	1738.42	78.47	860.43	0.50	Terrestrial	Shrubland	Endemic
Herminium chloranthum	Lijiang	2500	12.07	982.53	63.24	1077.65	1.10	Terrestrial	Grassy slope	Non-endemic
Herminium chloranthum	Lijiang	2800	10.27	982.53	63.24	1077.65	1.10	Terrestrial	Grassy slope	Non-endemic
Herminium chloranthum	Deqin	3400	4.77	639.48	70.85	896.66	1.40	Terrestrial	Grassy slope	Non-endemic
Herminium chloranthum	Lijiang	3550	5.77	982.53	63.24	1077.65	1.10	Terrestrial	Dense forest	Non-endemic
Herminium chloranthum	Weixi	3600	3.83	970.70	69.95	1021.03	1.05	Terrestrial	Grassy slope	Non-endemic
Herminium chloranthum	Zhongdian	3800	2.64	641.73	69.05	914.89	1.43	Terrestrial	Grassy slope	Non-endemic
Herminium chloranthum	Deqin	4020	1.05	639.48	70.85	896.66	1.40	Terrestrial	Grassy slope	Non-endemic
Herminium chloranthum	Deqin	4100	0.57	639.48	70.85	896.66	1.40	Terrestrial	Shrubland	Non-endemic
Herminium coiloglossum	Jingdong	1660	15.62	1128.40	76.80	1142.11	1.01	Terrestrial	Dense forest	Non-endemic
Herminium coiloglossum	Kunming	2300	12.48	1019.14	72.30	1197.62	1.18	Terrestrial	Dense forest	Non-endemic
Herminium coiloglossum	Yangbi	2900	8.59	1044.23	72.19	1161.15	1.11	Terrestrial	Dense forest	Non-endemic
Herminium coiloglossum	Zhaotong	3040	5.08	708.66	74.85	1046.05	1.48	Terrestrial	Meadow	Non-endemic
Herminium ecalcaratum	Chuxiong	2200	13.37	874.28	69.99	1192.36	1.36	Terrestrial	Dense forest	Non-endemic
Herminium ecalcaratum	Yangbi	2500	10.99	1044.23	72.19	1161.15	1.11	Terrestrial	Dense forest	Non-endemic
Herminium ecalcaratum	Heqing	2600	11.19	977.00	65.16	1211.84	1.24	Terrestrial	Grassy slope	Non-endemic
Herminium glossophyllum	Lijiang	2760	10.51	982.53	63.24	1077.65	1.10	Terrestrial	Grassy slope	Non-endemic
Herminium glossophyllum	Zhongdian	3000	7.44	641.73	69.05	914.89	1.43	Terrestrial	Grassy slope	Non-endemic
Herminium glossophyllum	Zhongdian	3430	4.86	641.73	69.05	914.89	1.43	Terrestrial	Dense forest	Non-endemic

Herminium josephi	Zhongdian	2800	8.64	641.73	69.05	914.89	1.43	Terrestrial	Grassy slope	Non-endemic
Herminium josephi	Dongchuan	3000	7.83	1021.73	71.65	1186.61	1.16	Terrestrial	Shrubland	Non-endemic
Herminium josephi	Dongchuan	3100	7.23	1021.73	71.65	1186.61	1.16	Terrestrial	Shrubland	Non-endemic
Herminium josephi	Dayao	3120	8.21	810.80	65.01	1427.38	1.76	Terrestrial	Meadow	Non-endemic
Herminium josephi	Huize	3400	4.97	799.98	71.46	1179.17	1.47	Terrestrial	Meadow	Non-endemic
Herminium josephi	Lijiang	3700	4.87	982.53	63.24	1077.65	1.10	Terrestrial	Meadow	Non-endemic
Herminium josephi	Fugong	4000	0.06	1441.43	79.99	906.18	0.63	Terrestrial	Meadow	Non-endemic
Herminium josephi	Fugong	4000	0.06	1441.43	79.99	906.18	0.63	Terrestrial	Meadow	Non-endemic
Herminium lanceum	Yongde	1300	19.33	1266.25	69.00	1283.63	1.01	Terrestrial	Dense forest	Non-endemic
Herminium lanceum	Gongshan	1600	14.48	1738.42	78.47	860.43	0.50	Terrestrial	Dense forest	Non-endemic
Herminium lanceum	Jiangchuan	1700	15.80	872.74	74.72	1181.99	1.35	Terrestrial	Dense forest	Non-endemic
Herminium lanceum	Jiangchuan	1750	15.50	872.74	74.72	1181.99	1.35	Terrestrial	Dense forest	Non-endemic
Herminium lanceum	Lushui	1790	15.31	1195.57	70.63	911.65	0.76	Terrestrial	Forest edge	Non-endemic
Herminium lanceum	Fugong	1800	13.26	1441.43	79.99	906.18	0.63	Terrestrial	Dense forest	Non-endemic
Herminium lanceum	Jinghong	1800	14.92	1161.08	80.46	1256.19	1.08	Terrestrial	Grassy slope	Non-endemic
Herminium lanceum	Yuliang	1800	14.72	905.45	75.03	1218.48	1.35	Terrestrial	Grassy slope	Non-endemic
Herminium lanceum	Deqin	1900	13.77	639.48	70.85	896.66	1.40	Terrestrial	Grassy slope	Non-endemic
Herminium lanceum	Yangbi	1980	14.11	1044.23	72.19	1161.15	1.11	Terrestrial	Grassy slope	Non-endemic
Herminium lanceum	Jingdong	2000	13.58	1128.40	76.80	1142.11	1.01	Terrestrial	Forest edge	Non-endemic
Herminium lanceum	Dayao	2000	14.93	810.80	65.01	1427.38	1.76	Terrestrial	Shrubland	Non-endemic
Herminium lanceum	Kunming	2030	14.10	1019.14	72.30	1197.62	1.18	Terrestrial	Grassy slope	Non-endemic
Herminium lanceum	Songming	2100	12.99	1010.18	74.14	1134.24	1.12	Terrestrial	Grassy slope	Non-endemic
Herminium lanceum	Jingdong	2120	12.86	1128.40	76.80	1142.11	1.01	Terrestrial	Dense forest	Non-endemic
Herminium lanceum	Jingdong	2120	12.86	1128.40	76.80	1142.11	1.01	Terrestrial	Dense forest	Non-endemic
Herminium lanceum	Kunming	2120	13.56	1019.14	72.30	1197.62	1.18	Terrestrial	Grassy slope	Non-endemic
Herminium lanceum	Kunming	2200	13.08	1019.14	72.30	1197.62	1.18	Terrestrial	Dense forest	Non-endemic
Herminium lanceum	Anning	2200	12.83	896.67	71.23	1167.91	1.30	Terrestrial	Shrubland	Non-endemic
Herminium lanceum	Songming	2200	12.39	1010.18	74.14	1134.24	1.12	Terrestrial	Shrubland	Non-endemic
Herminium lanceum	Eryuan	2230	13.15	745.16	68.27	1210.94	1.63	Terrestrial	Dense forest	Non-endemic
Herminium lanceum	Zhenkang	2250	11.34	1602.96	81.14	1089.09	0.68	Terrestrial	Grassy slope	Non-endemic
Herminium lanceum	Zhenkang	2250	11.34	1602.96	81.14	1089.09	0.68	Terrestrial	Grassy slope	Non-endemic
Herminium lanceum	Gongshan	2300	10.28	1738.42	78.47	860.43	0.50	Terrestrial	Grassy slope	Non-endemic
Herminium lanceum	Eryuan	2350	12.43	745.16	68.27	1210.94	1.63	Terrestrial	Dense forest	Non-endemic
Herminium lanceum	Eryuan	2350	12.43	745.16	68.27	1210.94	1.63	Terrestrial	Grassy slope	Non-endemic
Herminium lanceum	Songming	2380	11.31	1010.18	74.14	1134.24	1.12	Terrestrial	Grassy slope	Non-endemic
Herminium lanceum	Dali	2400	12.42	1082.70	68.61	1256.27	1.16	Terrestrial	Dense forest	Non-endemic

Herminium lanceum	Fengqing	2400	11.80	1352.80	73.24	1172.52	0.87	Terrestrial	Grassy slope	Non-endemic
Herminium lanceum	Kunming	2400	11.88	1019.14	72.30	1197.62	1.18	Terrestrial	Grassy slope	Non-endemic
Herminium lanceum	Jingdong	2400	11.18	1128.40	76.80	1142.11	1.01	Terrestrial	Grassy slope	Non-endemic
Herminium lanceum	Dali	2470	12.00	1082.70	68.61	1256.27	1.16	Terrestrial	Grassy slope	Non-endemic
Herminium lanceum	Gongshan	2500	9.08	1738.42	78.47	860.43	0.50	Terrestrial	Dense forest	Non-endemic
Herminium lanceum	Lijiang	2500	12.07	982.53	63.24	1077.65	1.10	Terrestrial	Grassy slope	Non-endemic
Herminium lanceum	Dali	2600	11.22	1082.70	68.61	1256.27	1.16	Terrestrial	Grassy slope	Non-endemic
Herminium lanceum	Yongde	2660	11.17	1266.25	69.00	1283.63	1.01	Terrestrial	Grassy slope	Non-endemic
Herminium lanceum	Lijiang	2700	10.87	982.53	63.24	1077.65	1.10	Terrestrial	Dense forest	Non-endemic
Herminium lanceum	Gongshan	2700	7.88	1738.42	78.47	860.43	0.50	Terrestrial	Grassy slope	Non-endemic
Herminium lanceum	Yongde	2700	10.93	1266.25	69.00	1283.63	1.01	Terrestrial	Grassy slope	Non-endemic
Herminium lanceum	Gongshan	2700	7.88	1738.42	78.47	860.43	0.50	Terrestrial	Meadow	Non-endemic
Herminium lanceum	Lijiang	2770	10.45	982.53	63.24	1077.65	1.10	Terrestrial	Grassy slope	Non-endemic
Herminium lanceum	Longling	2800	7.32	2098.66	84.65	973.45	0.46	Terrestrial	Meadow	Non-endemic
Herminium lanceum	Wenshan	2800	8.86	988.87	76.70	1272.98	1.29	Terrestrial	Shrubland	Non-endemic
Herminium lanceum	Yongde	2810	10.27	1266.25	69.00	1283.63	1.01	Terrestrial	Meadow	Non-endemic
Herminium lanceum	Lijiang	2850	9.97	982.53	63.24	1077.65	1.10	Terrestrial	Dense forest	Non-endemic
Herminium lanceum	Jingdong	2850	8.48	1128.40	76.80	1142.11	1.01	Terrestrial	Grassy slope	Non-endemic
Herminium lanceum	Dali	2900	9.42	1082.70	68.61	1256.27	1.16	Terrestrial	Meadow	Non-endemic
Herminium lanceum	Zhongdian	2900	8.04	641.73	69.05	914.89	1.43	Terrestrial	Meadow	Non-endemic
Herminium lanceum	Lijiang	2950	9.37	982.53	63.24	1077.65	1.10	Terrestrial	Shrubland	Non-endemic
Herminium lanceum	Heqing	3000	8.79	977.00	65.16	1211.84	1.24	Terrestrial	Dense forest	Non-endemic
Herminium lanceum	Weixi	3000	7.43	970.70	69.95	1021.03	1.05	Terrestrial	Dense forest	Non-endemic
Herminium lanceum	Zhongdian	3000	7.44	641.73	69.05	914.89	1.43	Terrestrial	Grassy slope	Non-endemic
Herminium lanceum	Dongchuan	3000	7.83	1021.73	71.65	1186.61	1.16	Terrestrial	Grassy slope	Non-endemic
Herminium lanceum	Huaping	3050	8.85	1092.50	61.93	1424.09	1.30	Terrestrial	Grassy slope	Non-endemic
Herminium lanceum	Dali	3100	8.22	1082.70	68.61	1256.27	1.16	Terrestrial	Meadow	Non-endemic
Herminium lanceum	Weixi	3200	6.23	970.70	69.95	1021.03	1.05	Terrestrial	Dense forest	Non-endemic
Herminium lanceum	Weixi	3300	5.63	970.70	69.95	1021.03	1.05	Terrestrial	Grassy slope	Non-endemic
Herminium lanceum	Weixi	3300	5.63	970.70	69.95	1021.03	1.05	Terrestrial	Grassy slope	Non-endemic
Herminium lanceum	Zhongdian	3300	5.64	641.73	69.05	914.89	1.43	Terrestrial	Grassy slope	Non-endemic
Herminium lanceum	Zhongdian	3300	5.64	641.73	69.05	914.89	1.43	Terrestrial	Meadow	Non-endemic
Herminium lanceum	Zhongdian	3440	4.80	641.73	69.05	914.89	1.43	Terrestrial	Dense forest	Non-endemic
Herminium lanceum	Deqin	3500	4.17	639.48	70.85	896.66	1.40	Terrestrial	Grassy slope	Non-endemic
Herminium lanceum	Dongchuan	3500	4.83	1021.73	71.65	1186.61	1.16	Terrestrial	Meadow	Non-endemic
Herminium lanceum	Zhongdian	3520	4.32	641.73	69.05	914.89	1.43	Terrestrial	Dense forest	Non-endemic

Herminium lanceum	Lijiang	3610	5.41	982.53	63.24	1077.65	1.10	Terrestrial	Meadow	Non-endemic
Herminium monorchis	Zhongdian	3000	7.44	641.73	69.05	914.89	1.43	Terrestrial	Dense forest	Non-endemic
Herminium monorchis	Zhongdian	4300	-0.36	641.73	69.05	914.89	1.43	Terrestrial	Shrubland	Non-endemic
Herminium ophioglossoides	Zhongdian	2300	11.64	641.73	69.05	914.89	1.43	Terrestrial	Shrubland	Non-endemic
Herminium ophioglossoides	Dali	2400	12.42	1082.70	68.61	1256.27	1.16	Terrestrial	Grassy slope	Non-endemic
Herminium ophioglossoides	Yaoan	2400	12.14	784.85	68.90	1295.94	1.65	Terrestrial	Shrubland	Non-endemic
Herminium ophioglossoides	Lijiang	2420	12.55	982.53	63.24	1077.65	1.10	Terrestrial	Grassy slope	Non-endemic
Herminium ophioglossoides	Lijiang	2600	11.47	982.53	63.24	1077.65	1.10	Terrestrial	Grassy slope	Non-endemic
Herminium ophioglossoides	Lijiang	2700	10.87	982.53	63.24	1077.65	1.10	Terrestrial	Shrubland	Non-endemic
Herminium ophioglossoides	Lijiang	2800	10.27	982.53	63.24	1077.65	1.10	Terrestrial	Meadow	Non-endemic
Herminium ophioglossoides	Lijiang	2870	9.85	982.53	63.24	1077.65	1.10	Terrestrial	Dense forest	Non-endemic
Herminium ophioglossoides	Zhongdian	3100	6.84	641.73	69.05	914.89	1.43	Terrestrial	Dense forest	Non-endemic
Herminium ophioglossoides	Zhongdian	3100	6.84	641.73	69.05	914.89	1.43	Terrestrial	Grassy slope	Non-endemic
Herminium ophioglossoides	Zhongdian	3200	6.24	641.73	69.05	914.89	1.43	Terrestrial	Meadow	Non-endemic
Herminium ophioglossoides	Lijiang	3200	7.87	982.53	63.24	1077.65	1.10	Terrestrial	Meadow	Non-endemic
Herminium ophioglossoides	Lijiang	3200	7.87	982.53	63.24	1077.65	1.10	Terrestrial	Meadow	Non-endemic
Herminium ophioglossoides	Zhongdian	3230	6.06	641.73	69.05	914.89	1.43	Terrestrial	Dense forest	Non-endemic
Herminium ophioglossoides	Zhongdian	3420	4.92	641.73	69.05	914.89	1.43	Terrestrial	Grassy slope	Non-endemic
Herminium ophioglossoides	Zhongdian	3500	4.44	641.73	69.05	914.89	1.43	Terrestrial	Open site	Non-endemic
Herminium quinquelobum	Gongshan	2200	10.88	1738.42	78.47	860.43	0.50	Terrestrial	Dense forest	Non-endemic
Herminium singulum	Fugong	2700	7.86	1441.43	79.99	906.18	0.63	Terrestrial	Dense forest	Non-endemic
Herminium souliei	Qiaojia	1400	15.22	878.15	76.01	1034.97	1.18	Terrestrial	Grassy slope	Non-endemic
Herminium souliei	Binchuan	1890	15.36	573.87	62.93	1418.42	2.47	Terrestrial	Dense forest	Non-endemic
Herminium souliei	Eryuan	2140	13.69	745.16	68.27	1210.94	1.63	Terrestrial	Grassy slope	Non-endemic
Herminium souliei	Lijiang	2800	10.27	982.53	63.24	1077.65	1.10	Terrestrial	Grassy slope	Non-endemic
Herminium souliei	Zhongdian	2900	8.04	641.73	69.05	914.89	1.43	Terrestrial	Dense forest	Non-endemic
Herminium souliei	Menglian	2900	8.18	1357.68	80.47	1185.94	0.87	Terrestrial	Meadow	Non-endemic
Herminium souliei	Lijiang	3000	9.07	982.53	63.24	1077.65	1.10	Terrestrial	Meadow	Non-endemic
Herminium souliei	Dongchuan	3050	7.53	1021.73	71.65	1186.61	1.16	Terrestrial	Grassy slope	Non-endemic
Herminium souliei	Zhongdian	3100	6.84	641.73	69.05	914.89	1.43	Terrestrial	Meadow	Non-endemic
Herminium souliei	Dali	3100	8.22	1082.70	68.61	1256.27	1.16	Terrestrial	Open site	Non-endemic
Herminium souliei	Lushui	3150	7.15	1195.57	70.63	911.65	0.76	Terrestrial	Meadow	Non-endemic
Herminium souliei	Zhongdian	3240	6.00	641.73	69.05	914.89	1.43	Terrestrial	Meadow	Non-endemic
Herminium souliei	Luquan	3250	6.22	965.09	73.60	1160.89	1.20	Terrestrial	Meadow	Non-endemic
Herminium souliei	Luquan	3250	6.22	965.09	73.60	1160.89	1.20	Terrestrial	Meadow	Non-endemic
Herminium souliei	Zhongdian	3300	5.64	641.73	69.05	914.89	1.43	Terrestrial	Meadow	Non-endemic

Herminium souliei	Zhongdian	3400	5.04	641.73	69.05	914.89	1.43	Terrestrial	Meadow	Non-endemic
Herminium souliei	Lijiang	3550	5.77	982.53	63.24	1077.65	1.10	Terrestrial	Grassy slope	Non-endemic
Herminium souliei	Lijiang	3550	5.77	982.53	63.24	1077.65	1.10	Terrestrial	Meadow	Non-endemic
Herminium souliei	Dali	3900	3.42	1082.70	68.61	1256.27	1.16	Terrestrial	Grassy slope	Non-endemic
Herminium souliei	Luquan	4000	1.72	965.09	73.60	1160.89	1.20	Terrestrial	Grassy slope	Non-endemic
Herminium souliei	Luquan	4200	0.52	965.09	73.60	1160.89	1.20	Terrestrial	Shrubland	Non-endemic
Herminium yunnanense	Yangbi	1730	15.61	1044.23	72.19	1161.15	1.11	Terrestrial	Grassy slope	Endemic
Herminium yunnanense	Chuxiong	2200	13.37	874.28	69.99	1192.36	1.36	Terrestrial	Dense forest	Endemic
Hetaeria rubens	Mengla	570	21.78	1514.70	84.25	1146.68	0.76	Terrestrial	Dense forest	Non-endemic
Hetaeria rubens	Jinghong	640	21.88	1161.08	80.46	1256.19	1.08	Terrestrial	Dense forest	Non-endemic
Hetaeria rubens	Jinghong	750	21.22	1161.08	80.46	1256.19	1.08	Terrestrial	Dense forest	Non-endemic
Hetaeria rubens	Jinghong	820	20.80	1161.08	80.46	1256.19	1.08	Terrestrial	Dense forest	Non-endemic
Hetaeria rubens	Malipo	1000	18.26	1063.54	85.83	1053.97	0.99	Terrestrial	Dense forest	Non-endemic
Holcoglossum amesianum	Cangyuan	1250	17.72	1733.34	81.63	1060.82	0.61	Epiphyte	Dense forest	Non-endemic
Holcoglossum amesianum	Menghai	1350	17.46	1314.38	80.83	1150.91	0.88	Epiphyte	Sparse forest	Non-endemic
Holcoglossum amesianum	Zhenkang	1640	15.00	1602.96	81.14	1089.09	0.68	Epiphyte	Dense forest	Non-endemic
Holcoglossum amesianum	Jingdong	1800	14.78	1128.40	76.80	1142.11	1.01	Epiphyte	Dense forest	Non-endemic
Holcoglossum flavescens	Yongde	2500	12.13	1266.25	69.00	1283.63	1.01	Epiphyte	Dense forest	Non-endemic
Holcoglossum kimballianum	Xichou	1000	18.93	1267.54	82.98	985.97	0.78	Epiphyte	Dense forest	Non-endemic
Holcoglossum kimballianum	Zhenyuan	1150	19.34	1254.48	77.40	1138.06	0.91	Epiphyte	Dense forest	Non-endemic
Holcoglossum kimballianum	Jianchuan	1430	16.97	753.13	70.16	1154.48	1.53	Epiphyte	Dense forest	Non-endemic
Holcoglossum kimballianum	Menghai	1630	15.78	1314.38	80.83	1150.91	0.88	Epiphyte	Sparse forest	Non-endemic
Holcoglossum kimballianum	Yongde	2500	12.13	1266.25	69.00	1283.63	1.01	Epiphyte	Dense forest	Non-endemic
Holcoglossum lingulatum	Malipo	1000	18.26	1063.54	85.83	1053.97	0.99	Epiphyte	Sparse forest	Non-endemic
Holcoglossum rupestre	Zhongdian	2200	12.24	641.73	69.05	914.89	1.43	Epiphyte	Dense forest	Endemic
Holcoglossum rupestre	Fugong	2400	9.66	1441.43	79.99	906.18	0.63	Epiphyte	Dense forest	Endemic
Holcoglossum rupestre	Yangbi	2570	10.57	1044.23	72.19	1161.15	1.11	Epiphyte	Dense forest	Endemic
Holcoglossum sinicum	Yangbi	1740	15.55	1044.23	72.19	1161.15	1.11	Epiphyte	Dense forest	Non-endemic
Holcoglossum sinicum	Yangbi	2710	9.73	1044.23	72.19	1161.15	1.11	Epiphyte	Dense forest	Non-endemic
Holcoglossum sinicum	Yangbi	3200	6.79	1044.23	72.19	1161.15	1.11	Epiphyte	Dense forest	Non-endemic
Holcoglossum wangii	Hekou	800	18.95	1768.58	84.25	1166.26	0.66	Epiphyte	Dense forest	Non-endemic
Hygrochilus parlshii	Menghai	1030	19.38	1314.38	80.83	1150.91	0.88	Epiphyte	Dense forest	Non-endemic
Hygrochilus parlshii	Menghai	1100	18.96	1314.38	80.83	1150.91	0.88	Epiphyte	Forest edge	Non-endemic
Ischnogyne mandarinanum	Jingdong	2600	9.98	1128.40	76.80	1142.11	1.01	Epiphyte	Dense forest	Non-endemic
Kingidium braceanum	Menghai	1500	16.56	1314.38	80.83	1150.91	0.88	Epiphyte	Dense forest	Non-endemic
Kingidium deliciosum	Pingbian	330	22.84	1648.57	86.34	990.16	0.60	Epiphyte	Dense forest	Non-endemic

Kingidium deliciosum	Menghai	1550	16.26	1314.38	80.83	1150.91	0.88	Epiphyte	Dense forest	Non-endemic
Kingidium taenialis	Jingdong	1100	18.98	1128.40	76.80	1142.11	1.01	Epiphyte	Dense forest	Non-endemic
Kingidium taenialis	Menghai	1280	17.88	1314.38	80.83	1150.91	0.88	Epiphyte	Shrubland	Non-endemic
Kingidium taenialis	Menghai	1350	17.46	1314.38	80.83	1150.91	0.88	Epiphyte	Dense forest	Non-endemic
Kingidium taenialis	Pingbian	1400	16.42	1648.57	86.34	990.16	0.60	Epiphyte	Dense forest	Non-endemic
Kingidium taenialis	Jingdong	1500	16.58	1128.40	76.80	1142.11	1.01	Epiphyte	Dense forest	Non-endemic
Kingidium taenialis	Menghai	1500	16.56	1314.38	80.83	1150.91	0.88	Epiphyte	Dense forest	Non-endemic
Kingidium taenialis	Fengqing	2100	13.60	1352.80	73.24	1172.52	0.87	Epiphyte	Dense forest	Non-endemic
Kingidium taenialis	Zhongdian	2200	12.24	641.73	69.05	914.89	1.43	Epiphyte	Dense forest	Non-endemic
Lecanorchis japonica	Luchun	2500	11.70	2013.54	78.64	1151.56	0.57	Saprophyte	Dense forest	Non-endemic
Liparis assamica	Jinghong	800	20.92	1161.08	80.46	1256.19	1.08	Epiphyte	Dense forest	Non-endemic
Liparis assamica	Gongshan	1300	16.28	1738.42	78.47	860.43	0.50	Epiphyte	Dense forest	Non-endemic
Liparis assamica	Tengchong	1800	14.20	1501.45	78.06	857.93	0.57	Epiphyte	Dense forest	Non-endemic
Liparis assamica	Luchun	2100	14.10	2013.54	78.64	1151.56	0.57	Epiphyte	Dense forest	Non-endemic
Liparis balansae	Pingbian	1400	16.42	1648.57	86.34	990.16	0.60	Epiphyte	Dense forest	Non-endemic
Liparis balansae	Xichou	1550	15.63	1267.54	82.98	985.97	0.78	Epiphyte	Dense forest	Non-endemic
Liparis balansae	Xichou	1600	15.33	1267.54	82.98	985.97	0.78	Epiphyte	Dense forest	Non-endemic
Liparis balansae	Malipo	1800	13.46	1063.54	85.83	1053.97	0.99	Epiphyte	Dense forest	Non-endemic
Liparis balansae	Lincang	2000	14.52	1165.84	72.08	1167.73	1.00	Epiphyte	Dense forest	Non-endemic
Liparis balansae	Luchun	2100	14.10	2013.54	78.64	1151.56	0.57	Epiphyte	Dense forest	Non-endemic
Liparis balansae	Luchun	2200	13.50	2013.54	78.64	1151.56	0.57	Epiphyte	Dense forest	Non-endemic
Liparis bautingensis	Malipo	1200	17.06	1063.54	85.83	1053.97	0.99	Epiphyte	Sparse forest	Non-endemic
Liparis bistriata	Wenshan	1020	19.54	988.87	76.70	1272.98	1.29	Epiphyte	Dense forest	Non-endemic
Liparis bootanensis	Malipo	1300	16.46	1063.54	85.83	1053.97	0.99	Epiphyte	Dense forest	Non-endemic
Liparis bootanensis	Malipo	1400	15.86	1063.54	85.83	1053.97	0.99	Epiphyte	Dense forest	Non-endemic
Liparis bootanensis	Gongshan	1400	15.68	1738.42	78.47	860.43	0.50	Epiphyte	Dense forest	Non-endemic
Liparis bootanensis	Gongshan	1400	15.68	1738.42	78.47	860.43	0.50	Epiphyte	Dense forest	Non-endemic
Liparis bootanensis	Gongshan	1400	15.68	1738.42	78.47	860.43	0.50	Epiphyte	Dense forest	Non-endemic
Liparis bootanensis	Gongshan	1420	15.56	1738.42	78.47	860.43	0.50	Epiphyte	Dense forest	Non-endemic
Liparis bootanensis	Gongshan	1450	15.38	1738.42	78.47	860.43	0.50	Epiphyte	Dense forest	Non-endemic
Liparis bootanensis	Gongshan	1500	15.08	1738.42	78.47	860.43	0.50	Epiphyte	Dense forest	Non-endemic
Liparis bootanensis	Pingbian	1600	15.22	1648.57	86.34	990.16	0.60	Epiphyte	Dense forest	Non-endemic
Liparis bootanensis	Pingbian	1600	15.22	1648.57	86.34	990.16	0.60	Epiphyte	Dense forest	Non-endemic
Liparis bootanensis	Gongshan	1600	14.48	1738.42	78.47	860.43	0.50	Epiphyte	Dense forest	Non-endemic
Liparis bootanensis	Xichou	1700	14.73	1267.54	82.98	985.97	0.78	Epiphyte	Dense forest	Non-endemic
Liparis caespitosa	Menglian	1000	19.58	1357.68	80.47	1185.94	0.87	Epiphyte	Dense forest	Non-endemic

Liparis caespitosa	Menghai	1100	18.96	1314.38	80.83	1150.91	0.88	Epiphyte	Dense forest	Non-endemic
Liparis caespitosa	Gongshan	1380	15.80	1738.42	78.47	860.43	0.50	Epiphyte	Dense forest	Non-endemic
Liparis caespitosa	Gongshan	1400	15.68	1738.42	78.47	860.43	0.50	Epiphyte	Dense forest	Non-endemic
Liparis caespitosa	Menghai	1400	17.16	1314.38	80.83	1150.91	0.88	Epiphyte	Dense forest	Non-endemic
Liparis caespitosa	Gongshan	1680	14.00	1738.42	78.47	860.43	0.50	Epiphyte	Dense forest	Non-endemic
Liparis caespitosa	Gongshan	1700	13.88	1738.42	78.47	860.43	0.50	Epiphyte	Dense forest	Non-endemic
Liparis caespitosa	Jinghong	1700	15.52	1161.08	80.46	1256.19	1.08	Epiphyte	Dense forest	Non-endemic
Liparis caespitosa	Gongshan	1800	13.28	1738.42	78.47	860.43	0.50	Epiphyte	Dense forest	Non-endemic
Liparis caespitosa	Gongshan	1950	12.38	1738.42	78.47	860.43	0.50	Epiphyte	Dense forest	Non-endemic
Liparis caespitosa	Gongshan	2000	12.08	1738.42	78.47	860.43	0.50	Epiphyte	Dense forest	Non-endemic
Liparis caespitosa	Gongshan	2000	12.08	1738.42	78.47	860.43	0.50	Epiphyte	Dense forest	Non-endemic
Liparis caespitosa	Gongshan	2000	12.08	1738.42	78.47	860.43	0.50	Epiphyte	Dense forest	Non-endemic
Liparis caespitosa	Yongde	2400	12.73	1266.25	69.00	1283.63	1.01	Epiphyte	Shrubland	Non-endemic
Liparis campylostalix	Fengqing	2650	10.30	1352.80	73.24	1172.52	0.87	Epiphyte	Dense forest	Non-endemic
Liparis cathcartii	Kunming	2200	13.08	1019.14	72.30	1197.62	1.18	Epiphyte	Dense forest	Non-endemic
Liparis cespitosa	Jinghong	800	20.92	1161.08	80.46	1256.19	1.08	Epiphyte	Dense forest	Non-endemic
Liparis chapaensis	Gongshan	1400	15.68	1738.42	78.47	860.43	0.50	Epiphyte	Dense forest	Non-endemic
Liparis chapaensis	Gongshan	1400	15.68	1738.42	78.47	860.43	0.50	Epiphyte	Dense forest	Non-endemic
Liparis chapaensis	Gongshan	2500	9.08	1738.42	78.47	860.43	0.50	Epiphyte	Dense forest	Non-endemic
Liparis cordifolia	Pingbian	1300	17.02	1648.57	86.34	990.16	0.60	Epiphyte	Dense forest	Non-endemic
Liparis delicatula	Jinghong	900	20.32	1161.08	80.46	1256.19	1.08	Epiphyte	Dense forest	Non-endemic
Liparis delicatula	Menghai	1030	19.38	1314.38	80.83	1150.91	0.88	Epiphyte	Dense forest	Non-endemic
Liparis delicatula	Menghai	1250	18.06	1314.38	80.83	1150.91	0.88	Epiphyte	Dense forest	Non-endemic
Liparis delicatula	Menghai	1300	17.76	1314.38	80.83	1150.91	0.88	Epiphyte	Dense forest	Non-endemic
Liparis distans	Funing	1000	17.62	1161.58	79.07	1147.17	0.99	Epiphyte	Dense forest	Non-endemic
Liparis distans	Malipo	1000	18.26	1063.54	85.83	1053.97	0.99	Epiphyte	Dense forest	Non-endemic
Liparis distans	Malipo	1100	17.66	1063.54	85.83	1053.97	0.99	Epiphyte	Dense forest	Non-endemic
Liparis distans	Yanshan	1200	18.26	1003.57	79.42	1172.21	1.17	Epiphyte	Dense forest	Non-endemic
Liparis distans	Yanshan	1200	18.26	1003.57	79.42	1172.21	1.17	Epiphyte	Dense forest	Non-endemic
Liparis distans	Pingbian	1200	17.62	1648.57	86.34	990.16	0.60	Epiphyte	Sparse forest	Non-endemic
Liparis distans	Malipo	1250	16.76	1063.54	85.83	1053.97	0.99	Epiphyte	Sparse forest	Non-endemic
Liparis distans	Jinghong	1270	18.10	1161.08	80.46	1256.19	1.08	Epiphyte	Dense forest	Non-endemic
Liparis distans	Gongshan	1300	16.28	1738.42	78.47	860.43	0.50	Epiphyte	Dense forest	Non-endemic
Liparis distans	Malipo	1300	16.46	1063.54	85.83	1053.97	0.99	Epiphyte	Dense forest	Non-endemic
Liparis distans	Xichou	1300	17.13	1267.54	82.98	985.97	0.78	Epiphyte	Dense forest	Non-endemic
Liparis distans	Xichou	1300	17.13	1267.54	82.98	985.97	0.78	Epiphyte	Dense forest	Non-endemic

Liparis distans	Xichou	1300	17.13	1267.54	82.98	985.97	0.78	Epiphyte	Dense forest	Non-endemic
Liparis distans	Xichou	1300	17.13	1267.54	82.98	985.97	0.78	Epiphyte	Dense forest	Non-endemic
Liparis distans	Xichou	1350	16.83	1267.54	82.98	985.97	0.78	Epiphyte	Sparse forest	Non-endemic
Liparis distans	Pingbian	1400	16.42	1648.57	86.34	990.16	0.60	Epiphyte	Dense forest	Non-endemic
Liparis distans	Pingbian	1400	16.42	1648.57	86.34	990.16	0.60	Epiphyte	Dense forest	Non-endemic
Liparis distans	Gongshan	1470	15.26	1738.42	78.47	860.43	0.50	Epiphyte	Dense forest	Non-endemic
Liparis distans	Xichou	1480	16.05	1267.54	82.98	985.97	0.78	Epiphyte	Dense forest	Non-endemic
Liparis distans	Malipo	1500	15.26	1063.54	85.83	1053.97	0.99	Epiphyte	Dense forest	Non-endemic
Liparis distans	Pingbian	1500	15.82	1648.57	86.34	990.16	0.60	Epiphyte	Dense forest	Non-endemic
Liparis distans	Xichou	1500	15.93	1267.54	82.98	985.97	0.78	Epiphyte	Shrubland	Non-endemic
Liparis distans	Malipo	1600	14.66	1063.54	85.83	1053.97	0.99	Epiphyte	Dense forest	Non-endemic
Liparis distans	Xichou	1600	15.33	1267.54	82.98	985.97	0.78	Epiphyte	Dense forest	Non-endemic
Liparis distans	Malipo	1600	14.66	1063.54	85.83	1053.97	0.99	Epiphyte	Dense forest	Non-endemic
Liparis distans	Pingbian	1700	14.62	1648.57	86.34	990.16	0.60	Epiphyte	Shrubland	Non-endemic
Liparis distans	Fugong	1800	13.26	1441.43	79.99	906.18	0.63	Epiphyte	Dense forest	Non-endemic
Liparis distans	Gongshan	1800	13.28	1738.42	78.47	860.43	0.50	Epiphyte	Dense forest	Non-endemic
Liparis distans	Gongshan	1800	13.28	1738.42	78.47	860.43	0.50	Epiphyte	Dense forest	Non-endemic
Liparis distans	Gongshan	1850	12.98	1738.42	78.47	860.43	0.50	Epiphyte	Sparse forest	Non-endemic
Liparis distans	Fugong	1900	12.66	1441.43	79.99	906.18	0.63	Epiphyte	Dense forest	Non-endemic
Liparis distans	Luchun	1900	15.30	2013.54	78.64	1151.56	0.57	Epiphyte	Dense forest	Non-endemic
Liparis distans	Gongshan	2000	12.08	1738.42	78.47	860.43	0.50	Epiphyte	Dense forest	Non-endemic
Liparis distans	Fugong	2000	12.06	1441.43	79.99	906.18	0.63	Epiphyte	Dense forest	Non-endemic
Liparis distans	Gongshan	2100	11.48	1738.42	78.47	860.43	0.50	Epiphyte	Dense forest	Non-endemic
Liparis distans	Gongshan	2300	10.28	1738.42	78.47	860.43	0.50	Epiphyte	Dense forest	Non-endemic
Liparis distans	Weixi	2400	11.03	970.70	69.95	1021.03	1.05	Epiphyte	Dense forest	Non-endemic
Liparis distans	Luchun	2400	12.30	2013.54	78.64	1151.56	0.57	Epiphyte	Dense forest	Non-endemic
Liparis distans	Fugong	2800	7.26	1441.43	79.99	906.18	0.63	Epiphyte	Dense forest	Non-endemic
Liparis elliptica	Mengla	700	21.00	1514.70	84.25	1146.68	0.76	Epiphyte	Dense forest	Non-endemic
Liparis elliptica	Menghai	1300	17.76	1314.38	80.83	1150.91	0.88	Epiphyte	Dense forest	Non-endemic
Liparis elliptica	Gongshan	1300	16.28	1738.42	78.47	860.43	0.50	Epiphyte	Dense forest	Non-endemic
Liparis elliptica	Jinping	1500	16.49	2305.17	83.81	1030.46	0.45	Epiphyte	Dense forest	Non-endemic
Liparis elliptica	Menghai	1520	16.44	1314.38	80.83	1150.91	0.88	Epiphyte	Shrubland	Non-endemic
Liparis elliptica	Menghai	1530	16.38	1314.38	80.83	1150.91	0.88	Epiphyte	Dense forest	Non-endemic
Liparis elliptica	Menghai	1630	15.78	1314.38	80.83	1150.91	0.88	Terrestrial	Sparse forest	Non-endemic
Liparis glossula	Wenshan	2200	12.46	988.87	76.70	1272.98	1.29	Terrestrial	Dense forest	Non-endemic
Liparis japonica	Jingdong	1670	15.56	1128.40	76.80	1142.11	1.01	Terrestrial	Grassy slope	Non-endemic

Liparis japonica	Yangbi	1770	15.37	1044.23	72.19	1161.15	1.11	Terrestrial	Dense forest	Non-endemic
Liparis japonica	Yiliang	1900	10.88	751.45	71.67	1097.64	1.46	Terrestrial	Dense forest	Non-endemic
Liparis japonica	Lincang	1900	15.12	1165.84	72.08	1167.73	1.00	Terrestrial	Grassy slope	Non-endemic
Liparis japonica	Zhenxiong	1900	10.04	899.34	83.70	852.28	0.95	Terrestrial	Shrubland	Non-endemic
Liparis japonica	Gongshan	2070	11.66	1738.42	78.47	860.43	0.50	Terrestrial	Dense forest	Non-endemic
Liparis japonica	Yongde	2350	13.03	1266.25	69.00	1283.63	1.01	Terrestrial	Grassy slope	Non-endemic
Liparis japonica	Songming	2400	11.19	1010.18	74.14	1134.24	1.12	Terrestrial	Dense forest	Non-endemic
Liparis japonica	Yongde	2400	12.73	1266.25	69.00	1283.63	1.01	Terrestrial	Grassy slope	Non-endemic
Liparis japonica	Yongde	2400	12.73	1266.25	69.00	1283.63	1.01	Terrestrial	Grassy slope	Non-endemic
Liparis japonica	Dongchuan	3000	7.83	1021.73	71.65	1186.61	1.16	Terrestrial	Grassy slope	Non-endemic
Liparis latilabris	Maguan	1400	16.61	1330.52	83.25	1086.43	0.82	Epiphyte	Dense forest	Non-endemic
Liparis latilabris	Maguan	1400	16.61	1330.52	83.25	1086.43	0.82	Epiphyte	Dense forest	Non-endemic
Liparis latilabris	Anning	2100	13.43	896.67	71.23	1167.91	1.30	Epiphyte	Dense forest	Non-endemic
Liparis mannii	Mengla	820	20.28	1514.70	84.25	1146.68	0.76	Epiphyte	Dense forest	Non-endemic
Liparis nervosa	Hekou	320	21.83	1768.58	84.25	1166.26	0.66	Terrestrial	Dense forest	Non-endemic
Liparis nervosa	Hekou	480	20.87	1768.58	84.25	1166.26	0.66	Terrestrial	Dense forest	Non-endemic
Liparis nervosa	Mengla	500	22.20	1514.70	84.25	1146.68	0.76	Terrestrial	Open site	Non-endemic
Liparis nervosa	Mengla	800	20.40	1514.70	84.25	1146.68	0.76	Terrestrial	Dense forest	Non-endemic
Liparis nervosa	Shizong	850	19.95	1204.89	79.59	1053.84	0.88	Terrestrial	Grassy slope	Non-endemic
Liparis nervosa	Shizong	900	19.65	1204.89	79.59	1053.84	0.88	Terrestrial	Grassy slope	Non-endemic
Liparis nervosa	Yanshan	1200	18.26	1003.57	79.42	1172.21	1.17	Terrestrial	Dense forest	Non-endemic
Liparis nervosa	Gongshan	1200	16.88	1738.42	78.47	860.43	0.50	Terrestrial	Dense forest	Non-endemic
Liparis nervosa	Malipo	1200	17.06	1063.54	85.83	1053.97	0.99	Terrestrial	Sparse forest	Non-endemic
Liparis nervosa	Gongshan	1380	15.80	1738.42	78.47	860.43	0.50	Terrestrial	Dense forest	Non-endemic
Liparis nervosa	Xichou	1500	15.93	1267.54	82.98	985.97	0.78	Terrestrial	Dense forest	Non-endemic
Liparis nervosa	Eshan	1500	16.21	938.39	78.62	1123.48	1.20	Terrestrial	Dense forest	Non-endemic
Liparis nervosa	Pingbian	1600	15.22	1648.57	86.34	990.16	0.60	Terrestrial	Dense forest	Non-endemic
Liparis nervosa	Yongde	1600	17.53	1266.25	69.00	1283.63	1.01	Terrestrial	Dense forest	Non-endemic
Liparis nervosa	Lincang	1700	16.32	1165.84	72.08	1167.73	1.00	Terrestrial	Dense forest	Non-endemic
Liparis nervosa	Yangbi	1730	15.61	1044.23	72.19	1161.15	1.11	Terrestrial	Dense forest	Non-endemic
Liparis nervosa	Malipo	2000	12.26	1063.54	85.83	1053.97	0.99	Terrestrial	Dense forest	Non-endemic
Liparis nervosa	Tengchong	2020	12.88	1501.45	78.06	857.93	0.57	Terrestrial	Dense forest	Non-endemic
Liparis nervosa	Fengqing	2100	13.60	1352.80	73.24	1172.52	0.87	Terrestrial	Dense forest	Non-endemic
Liparis nervosa	Jingdong	2200	12.38	1128.40	76.80	1142.11	1.01	Terrestrial	Shrubland	Non-endemic
Liparis nigra	Yongde	1400	18.73	1266.25	69.00	1283.63	1.01	Terrestrial	Grassy slope	Non-endemic
Liparis odorata	Jinghong	550	22.42	1161.08	80.46	1256.19	1.08	Terrestrial	Sparse forest	Non-endemic

Liparis odorata	Jinghong	900	20.32	1161.08	80.46	1256.19	1.08	Terrestrial	Dense forest	Non-endemic
Liparis odorata	Pingbian	1400	16.42	1648.57	86.34	990.16	0.60	Terrestrial	Dense forest	Non-endemic
Liparis odorata	Yongren	1630	16.97	865.52	65.40	1457.77	1.68	Terrestrial	Forest edge	Non-endemic
Liparis odorata	Anning	1820	15.11	896.67	71.23	1167.91	1.30	Terrestrial	Dense forest	Non-endemic
Liparis odorata	Kunming	1900	14.88	1019.14	72.30	1197.62	1.18	Terrestrial	Dense forest	Non-endemic
Liparis odorata	Jingdong	2100	12.98	1128.40	76.80	1142.11	1.01	Terrestrial	Dense forest	Non-endemic
Liparis odorata	Anning	2100	13.43	896.67	71.23	1167.91	1.30	Terrestrial	Sparse forest	Non-endemic
Liparis odorata	Heqing	2200	13.59	977.00	65.16	1211.84	1.24	Terrestrial	Grassy slope	Non-endemic
Liparis pauliana	Jingdong	1900	14.18	1128.40	76.80	1142.11	1.01	Terrestrial	Dense forest	Non-endemic
Liparis pauliana	Kunming	1950	14.58	1019.14	72.30	1197.62	1.18	Terrestrial	Dense forest	Non-endemic
Liparis pauliana	Kunming	2100	13.68	1019.14	72.30	1197.62	1.18	Terrestrial	Dense forest	Non-endemic
Liparis pauliana	Kunming	2200	13.08	1019.14	72.30	1197.62	1.18	Terrestrial	Dense forest	Non-endemic
Liparis pauliana	Kunming	2300	12.48	1019.14	72.30	1197.62	1.18	Terrestrial	Grassy slope	Non-endemic
Liparis petiolata	Mengla	1000	19.20	1514.70	84.25	1146.68	0.76	Terrestrial	Dense forest	Non-endemic
Liparis petiolata	Menglian	2400	11.18	1357.68	80.47	1185.94	0.87	Terrestrial	Shrubland	Non-endemic
Liparis petiolata	Lincang	2900	9.12	1165.84	72.08	1167.73	1.00	Terrestrial	Dense forest	Non-endemic
Liparis platyrachis	Dali	1500	17.82	1082.70	68.61	1256.27	1.16	Epiphyte	Dense forest	Non-endemic
Liparis regnieri	Jingdong	980	19.70	1128.40	76.80	1142.11	1.01	Terrestrial	Dense forest	Non-endemic
Liparis resupinata	Gongshan	1320	16.16	1738.42	78.47	860.43	0.50	Epiphyte	Dense forest	Non-endemic
Liparis resupinata	Gongshan	1400	15.68	1738.42	78.47	860.43	0.50	Epiphyte	Dense forest	Non-endemic
Liparis resupinata	Gongshan	1450	15.38	1738.42	78.47	860.43	0.50	Epiphyte	Dense forest	Non-endemic
Liparis resupinata	Gongshan	1450	15.38	1738.42	78.47	860.43	0.50	Epiphyte	Dense forest	Non-endemic
Liparis resupinata	Malipo	1500	15.26	1063.54	85.83	1053.97	0.99	Epiphyte	Dense forest	Non-endemic
Liparis resupinata	Gongshan	1500	15.08	1738.42	78.47	860.43	0.50	Epiphyte	Dense forest	Non-endemic
Liparis resupinata	Gongshan	1500	15.08	1738.42	78.47	860.43	0.50	Epiphyte	Dense forest	Non-endemic
Liparis resupinata	Malipo	1560	14.90	1063.54	85.83	1053.97	0.99	Epiphyte	Dense forest	Non-endemic
Liparis resupinata	Gongshan	1600	14.48	1738.42	78.47	860.43	0.50	Epiphyte	Dense forest	Non-endemic
Liparis resupinata	Gongshan	1600	14.48	1738.42	78.47	860.43	0.50	Epiphyte	Dense forest	Non-endemic
Liparis resupinata	Gongshan	1600	14.48	1738.42	78.47	860.43	0.50	Epiphyte	Dense forest	Non-endemic
Liparis resupinata	Gongshan	1750	13.58	1738.42	78.47	860.43	0.50	Epiphyte	Dense forest	Non-endemic
Liparis resupinata	Gongshan	1800	13.28	1738.42	78.47	860.43	0.50	Epiphyte	Dense forest	Non-endemic
Liparis resupinata	Gongshan	1800	13.28	1738.42	78.47	860.43	0.50	Epiphyte	Shrubland	Non-endemic
Liparis resupinata	Jingdong	2100	12.98	1128.40	76.80	1142.11	1.01	Epiphyte	Dense forest	Non-endemic
Liparis resupinata	Jingdong	2100	12.98	1128.40	76.80	1142.11	1.01	Epiphyte	Dense forest	Non-endemic
Liparis resupinata	Luchun	2200	13.50	2013.54	78.64	1151.56	0.57	Epiphyte	Dense forest	Non-endemic
Liparis resupinata	Jingdong	2400	11.18	1128.40	76.80	1142.11	1.01	Epiphyte	Dense forest	Non-endemic

Liparis resupinata	Lincang	2500	11.52	1165.84	72.08	1167.73	1.00	Epiphyte	Dense forest	Non-endemic
Liparis siamensis	Jinghong	700	21.52	1161.08	80.46	1256.19	1.08	Epiphyte	Dense forest	Non-endemic
Liparis stricklandiana	Malipo	1350	16.16	1063.54	85.83	1053.97	0.99	Epiphyte	Forest edge	Non-endemic
Liparis stricklandiana	Gongshan	1400	15.68	1738.42	78.47	860.43	0.50	Epiphyte	Dense forest	Non-endemic
Liparis stricklandiana	Gongshan	1480	15.20	1738.42	78.47	860.43	0.50	Epiphyte	Dense forest	Non-endemic
Liparis stricklandiana	Malipo	1500	15.26	1063.54	85.83	1053.97	0.99	Epiphyte	Dense forest	Non-endemic
Liparis stricklandiana	Gongshan	1580	14.60	1738.42	78.47	860.43	0.50	Epiphyte	Dense forest	Non-endemic
Liparis stricklandiana	Gongshan	1620	14.36	1738.42	78.47	860.43	0.50	Epiphyte	Dense forest	Non-endemic
Liparis stricklandiana	Gongshan	1720	13.76	1738.42	78.47	860.43	0.50	Epiphyte	Dense forest	Non-endemic
Liparis stricklandiana	Gongshan	1800	13.28	1738.42	78.47	860.43	0.50	Epiphyte	Dense forest	Non-endemic
Liparis stricklandiana	Gongshan	1800	13.28	1738.42	78.47	860.43	0.50	Epiphyte	Dense forest	Non-endemic
Liparis stricklandiana	Gongshan	2000	12.08	1738.42	78.47	860.43	0.50	Epiphyte	Dense forest	Non-endemic
Liparis tschangii	Jinghong	1100	19.12	1161.08	80.46	1256.19	1.08	Terrestrial	Dense forest	Non-endemic
Liparis viridiflora	Mengla	550	21.90	1514.70	84.25	1146.68	0.76	Epiphyte	Dense forest	Non-endemic
Liparis viridiflora	Mengla	700	21.00	1514.70	84.25	1146.68	0.76	Epiphyte	Dense forest	Non-endemic
Liparis viridiflora	Mengla	720	20.88	1514.70	84.25	1146.68	0.76	Epiphyte	Dense forest	Non-endemic
Liparis viridiflora	Menghai	720	21.24	1314.38	80.83	1150.91	0.88	Epiphyte	Dense forest	Non-endemic
Liparis viridiflora	Mengla	720	20.88	1514.70	84.25	1146.68	0.76	Epiphyte	Dense forest	Non-endemic
Liparis viridiflora	Mengla	750	20.70	1514.70	84.25	1146.68	0.76	Epiphyte	Dense forest	Non-endemic
Liparis viridiflora	Mengla	800	20.40	1514.70	84.25	1146.68	0.76	Epiphyte	Forest edge	Non-endemic
Liparis viridiflora	Jinghong	800	20.92	1161.08	80.46	1256.19	1.08	Epiphyte	Shrubland	Non-endemic
Liparis viridiflora	Jinghong	840	20.68	1161.08	80.46	1256.19	1.08	Epiphyte	Dense forest	Non-endemic
Liparis viridiflora	Jinghong	840	20.68	1161.08	80.46	1256.19	1.08	Epiphyte	Shrubland	Non-endemic
Liparis viridiflora	Mengla	850	20.10	1514.70	84.25	1146.68	0.76	Epiphyte	Dense forest	Non-endemic
Liparis viridiflora	Mengla	850	20.10	1514.70	84.25	1146.68	0.76	Epiphyte	Forest edge	Non-endemic
Liparis viridiflora	Mengla	880	19.92	1514.70	84.25	1146.68	0.76	Epiphyte	Dense forest	Non-endemic
Liparis viridiflora	Xichou	1000	18.93	1267.54	82.98	985.97	0.78	Epiphyte	Dense forest	Non-endemic
Liparis viridiflora	Xichou	1000	18.93	1267.54	82.98	985.97	0.78	Epiphyte	Dense forest	Non-endemic
Liparis viridiflora	Malipo	1000	18.26	1063.54	85.83	1053.97	0.99	Epiphyte	Dense forest	Non-endemic
Liparis viridiflora	Lushui	1000	20.05	1195.57	70.63	911.65	0.76	Epiphyte	Dense forest	Non-endemic
Liparis viridiflora	Mengla	1000	19.20	1514.70	84.25	1146.68	0.76	Epiphyte	Dense forest	Non-endemic
Liparis viridiflora	Mengla	1000	19.20	1514.70	84.25	1146.68	0.76	Epiphyte	Dense forest	Non-endemic
Liparis viridiflora	Mengla	1080	18.72	1514.70	84.25	1146.68	0.76	Epiphyte	Dense forest	Non-endemic
Liparis viridiflora	Malipo	1100	17.66	1063.54	85.83	1053.97	0.99	Epiphyte	Shrubland	Non-endemic
Liparis viridiflora	Zhenyuan	1150	19.34	1254.48	77.40	1138.06	0.91	Epiphyte	Dense forest	Non-endemic
Liparis viridiflora	Menghai	1150	18.66	1314.38	80.83	1150.91	0.88	Epiphyte	Dense forest	Non-endemic

Liparis viridiflora	Mengla	1200	18.00	1514.70	84.25	1146.68	0.76	Epiphyte	Dense forest	Non-endemic
Liparis viridiflora	Mengla	1200	18.00	1514.70	84.25	1146.68	0.76	Epiphyte	Dense forest	Non-endemic
Liparis viridiflora	Menghai	1200	18.36	1314.38	80.83	1150.91	0.88	Epiphyte	Dense forest	Non-endemic
Liparis viridiflora	Xichou	1300	17.13	1267.54	82.98	985.97	0.78	Epiphyte	Dense forest	Non-endemic
Liparis viridiflora	Xichou	1300	17.13	1267.54	82.98	985.97	0.78	Epiphyte	Dense forest	Non-endemic
Liparis viridiflora	Menghai	1300	17.76	1314.38	80.83	1150.91	0.88	Epiphyte	Sparse forest	Non-endemic
Liparis viridiflora	Yuxi	1370	17.55	928.04	74.41	1159.15	1.25	Epiphyte	Dense forest	Non-endemic
Liparis viridiflora	Jingdong	1400	17.18	1128.40	76.80	1142.11	1.01	Epiphyte	Dense forest	Non-endemic
Liparis viridiflora	Lincang	1400	18.12	1165.84	72.08	1167.73	1.00	Epiphyte	Dense forest	Non-endemic
Liparis viridiflora	Jinghong	1400	17.32	1161.08	80.46	1256.19	1.08	Epiphyte	Shrubland	Non-endemic
Liparis viridiflora	Xichou	1500	15.93	1267.54	82.98	985.97	0.78	Epiphyte	Dense forest	Non-endemic
Liparis viridiflora	Menghai	1540	16.32	1314.38	80.83	1150.91	0.88	Epiphyte	Dense forest	Non-endemic
Liparis viridiflora	Luxi	1550	15.78	1650.33	79.33	1183.39	0.72	Epiphyte	Dense forest	Non-endemic
Liparis viridiflora	Malipo	1800	13.46	1063.54	85.83	1053.97	0.99	Epiphyte	Dense forest	Non-endemic
Liparis viridiflora	Malipo	2000	12.26	1063.54	85.83	1053.97	0.99	Epiphyte	Shrubland	Non-endemic
Liparis viridiflora	Luchun	2200	13.50	2013.54	78.64	1151.56	0.57	Epiphyte	Dense forest	Non-endemic
Liparis viridiflora	Weixi	2300	11.63	970.70	69.95	1021.03	1.05	Epiphyte	Dense forest	Non-endemic
Listera grandiflora	Gongshan	2800	7.28	1738.42	78.47	860.43	0.50	Terrestrial	Dense forest	Non-endemic
Listera grandiflora	Yongde	2950	9.43	1266.25	69.00	1283.63	1.01	Terrestrial	Dense forest	Non-endemic
Listera grandiflora	Weixi	3000	7.43	970.70	69.95	1021.03	1.05	Terrestrial	Dense forest	Non-endemic
Listera grandiflora	Weixi	3000	7.43	970.70	69.95	1021.03	1.05	Terrestrial	sparse forest	Non-endemic
Listera grandiflora	Zhongdian	3200	6.24	641.73	69.05	914.89	1.43	Terrestrial	Dense forest	Non-endemic
Listera grandiflora	Weixi	3500	4.43	970.70	69.95	1021.03	1.05	Terrestrial	Dense forest	Non-endemic
Listera mucronata	Tengchong	2400	10.60	1501.45	78.06	857.93	0.57	Terrestrial	Meadow	Non-endemic
Listera nanchuanica	Daguan	2150	8.92	987.00	80.05	896.37	0.91	Terrestrial	Sparse forest	Non-endemic
Listera pinetorum	Gongshan	2300	10.28	1738.42	78.47	860.43	0.50	Terrestrial	Dense forest	Non-endemic
Listera pinetorum	Gongshan	2300	10.28	1738.42	78.47	860.43	0.50	Terrestrial	Dense forest	Non-endemic
Listera pinetorum	Gongshan	2500	9.08	1738.42	78.47	860.43	0.50	Terrestrial	Dense forest	Non-endemic
Listera pinetorum	Gongshan	2500	9.08	1738.42	78.47	860.43	0.50	Terrestrial	Grassy slope	Non-endemic
Listera yunnanensis	Wenshan	2300	11.86	988.87	76.70	1272.98	1.29	Terrestrial	Dense forest	Endemic
Luisia brachystachys	Menghai	1180	18.48	1314.38	80.83	1150.91	0.88	Epiphyte	Dense forest	Non-endemic
Luisia filiformis	Jinping	350	23.39	2305.17	83.81	1030.46	0.45	Epiphyte	Dense forest	Non-endemic
Luisia filiformis	Jinping	800	20.69	2305.17	83.81	1030.46	0.45	Epiphyte	Dense forest	Non-endemic
Luisia filiformis	Menghai	1100	18.96	1314.38	80.83	1150.91	0.88	Epiphyte	Dense forest	Non-endemic
Luisia filiformis	Jinping	1750	14.99	2305.17	83.81	1030.46	0.45	Epiphyte	Dense forest	Non-endemic
Luisia longispica	Mengla	800	20.40	1514.70	84.25	1146.68	0.76	Epiphyte	Dense forest	Endemic

Luisia magniflora	Menghai	1900	14.16	1314.38	80.83	1150.91	0.88	Epiphyte	Dense forest	Endemic
Luisia morsei	Mengla	590	21.66	1514.70	84.25	1146.68	0.76	Epiphyte	Dense forest	Non-endemic
Luisia morsei	Maguan	800	20.21	1330.52	83.25	1086.43	0.82	Epiphyte	Dense forest	Non-endemic
Luisia morsei	Shizong	850	19.95	1204.89	79.59	1053.84	0.88	Epiphyte	Dense forest	Non-endemic
Luisia morsei	Cangyuan	1200	18.02	1733.34	81.63	1060.82	0.61	Epiphyte	Dense forest	Non-endemic
Luisia morsei	Yongde	1300	19.33	1266.25	69.00	1283.63	1.01	Epiphyte	Shrubland	Non-endemic
Luisia terelibalia	Mengla	1000	19.20	1514.70	84.25	1146.68	0.76	Epiphyte	Dense forest	Non-endemic
Luisia teres	Yanshan	1200	18.26	1003.57	79.42	1172.21	1.17	Epiphyte	Dense forest	Non-endemic
Luisia zollingeri	Mengla	950	19.50	1514.70	84.25	1146.68	0.76	Epiphyte	Dense forest	Non-endemic
Malaxis acuminata	Jinghong	550	22.42	1161.08	80.46	1256.19	1.08	Terrestrial	Sparse forest	Non-endemic
Malaxis acuminata	Funing	600	20.02	1161.58	79.07	1147.17	0.99	Terrestrial	Open site	Non-endemic
Malaxis acuminata	Menghai	1600	15.96	1314.38	80.83	1150.91	0.88	Terrestrial	Dense forest	Non-endemic
Malaxis acuminata	Menghai	1600	15.96	1314.38	80.83	1150.91	0.88	Terrestrial	Dense forest	Non-endemic
Malaxis acuminata	Lushui	1600	16.45	1195.57	70.63	911.65	0.76	Terrestrial	Grassy slope	Non-endemic
Malaxis acuminata	Lushui	1800	15.25	1195.57	70.63	911.65	0.76	Terrestrial	Sparse forest	Non-endemic
Malaxis acuminata	Fengqing	2100	13.60	1352.80	73.24	1172.52	0.87	Terrestrial	Dense forest	Non-endemic
Malaxis bahanensis	Eryuan	1870	15.31	745.16	68.27	1210.94	1.63	Terrestrial	Grassy slope	Endemic
Malaxis bahanensis	Lijiang	2250	13.57	982.53	63.24	1077.65	1.10	Terrestrial	Grassy slope	Endemic
Malaxis biaurita	Menghai	1350	17.46	1314.38	80.83	1150.91	0.88	Terrestrial	Dense forest	Non-endemic
Malaxis biaurita	Gongshan	2500	9.08	1738.42	78.47	860.43	0.50	Terrestrial	Dense forest	Non-endemic
Malaxis calophylla	Cangyuan	1200	18.02	1733.34	81.63	1060.82	0.61	Terrestrial	Dense forest	Non-endemic
Malaxis khasiana	Gongshan	1500	15.08	1738.42	78.47	860.43	0.50	Terrestrial	Dense forest	Non-endemic
Malaxis latifolia	Pingbian	600	21.22	1648.57	86.34	990.16	0.60	Terrestrial	Dense forest	Non-endemic
Malaxis latifolia	Jingdong	980	19.70	1128.40	76.80	1142.11	1.01	Terrestrial	Dense forest	Non-endemic
Malaxis latifolia	Jinghong	1100	19.12	1161.08	80.46	1256.19	1.08	Terrestrial	Dense forest	Non-endemic
Malaxis latifolia	Menghai	1150	18.66	1314.38	80.83	1150.91	0.88	Terrestrial	Open site	Non-endemic
Malaxis latifolia	Lincang	1300	18.72	1165.84	72.08	1167.73	1.00	Terrestrial	Dense forest	Non-endemic
Malaxis latifolia	Menghai	1460	16.80	1314.38	80.83	1150.91	0.88	Terrestrial	Dense forest	Non-endemic
Malaxis latifolia	Eshan	1500	16.21	938.39	78.62	1123.48	1.20	Terrestrial	Dense forest	Non-endemic
Malaxis latifolia	Yongde	1500	18.13	1266.25	69.00	1283.63	1.01	Terrestrial	Forest edge	Non-endemic
Malaxis latifolia	Eshan	1600	15.61	938.39	78.62	1123.48	1.20	Terrestrial	Shrubland	Non-endemic
Malaxis latifolia	Yongde	1800	16.33	1266.25	69.00	1283.63	1.01	Terrestrial	Forest edge	Non-endemic
Malaxis monophyllos	Gongshan	2250	10.58	1738.42	78.47	860.43	0.50	Terrestrial	Dense forest	Non-endemic
Malaxis monophyllos	Qiaojia	2300	9.82	878.15	76.01	1034.97	1.18	Terrestrial	Grassy slope	Non-endemic
Malaxis monophyllos	Yongde	2360	12.97	1266.25	69.00	1283.63	1.01	Terrestrial	Grassy slope	Non-endemic
Malaxis monophyllos	Lijiang	2500	12.07	982.53	63.24	1077.65	1.10	Terrestrial	Dense forest	Non-endemic

Malaxis monophyllos	Yongde	2700	10.93	1266.25	69.00	1283.63	1.01	Terrestrial	Grassy slope	Non-endemic
Malaxis monophyllos	Lijiang	2800	10.27	982.53	63.24	1077.65	1.10	Terrestrial	Dense forest	Non-endemic
Malaxis monophyllos	Weixi	2800	8.63	970.70	69.95	1021.03	1.05	Terrestrial	Dense forest	Non-endemic
Malaxis monophyllos	Lijiang	2800	10.27	982.53	63.24	1077.65	1.10	Terrestrial	Dense forest	Non-endemic
Malaxis monophyllos	Longling	2800	7.32	2098.66	84.65	973.45	0.46	Terrestrial	Forest edge	Non-endemic
Malaxis monophyllos	Qiaojia	2800	6.82	878.15	76.01	1034.97	1.18	Terrestrial	Grassy slope	Non-endemic
Malaxis monophyllos	Qiaojia	2830	6.64	878.15	76.01	1034.97	1.18	Terrestrial	Grassy slope	Non-endemic
Malaxis monophyllos	Lijiang	2850	9.97	982.53	63.24	1077.65	1.10	Terrestrial	Dense forest	Non-endemic
Malaxis monophyllos	Yongde	2980	9.25	1266.25	69.00	1283.63	1.01	Terrestrial	Dense forest	Non-endemic
Malaxis monophyllos	Deqin	3000	7.17	639.48	70.85	896.66	1.40	Terrestrial	Dense forest	Non-endemic
Malaxis monophyllos	Zhenkang	3000	6.84	1602.96	81.14	1089.09	0.68	Terrestrial	Shrubland	Non-endemic
Malaxis monophyllos	Zhongdian	3100	6.84	641.73	69.05	914.89	1.43	Terrestrial	Grassy slope	Non-endemic
Malaxis monophyllos	Yongde	3100	8.53	1266.25	69.00	1283.63	1.01	Terrestrial	Meadow	Non-endemic
Malaxis monophyllos	Zhongdian	3100	6.84	641.73	69.05	914.89	1.43	Terrestrial	Meadow	Non-endemic
Malaxis monophyllos	Dali	3150	7.92	1082.70	68.61	1256.27	1.16	Terrestrial	Grassy slope	Non-endemic
Malaxis monophyllos	Zhongdian	3200	6.24	641.73	69.05	914.89	1.43	Terrestrial	Dense forest	Non-endemic
Malaxis monophyllos	Deqin	3400	4.77	639.48	70.85	896.66	1.40	Terrestrial	Grassy slope	Non-endemic
Malaxis monophyllos	Yongde	3410	6.67	1266.25	69.00	1283.63	1.01	Terrestrial	Dense forest	Non-endemic
Malaxis monophyllos	Zhongdian	3450	4.74	641.73	69.05	914.89	1.43	Terrestrial	Grassy slope	Non-endemic
Malaxis monophyllos	Deqin	3450	4.47	639.48	70.85	896.66	1.40	Terrestrial	Shrubland	Non-endemic
Malaxis monophyllos	Zhenkang	3500	3.84	1602.96	81.14	1089.09	0.68	Terrestrial	Grassy slope	Non-endemic
Malaxis monophyllos	Weixi	3500	4.43	970.70	69.95	1021.03	1.05	Terrestrial	Grassy slope	Non-endemic
Malaxis monophyllos	Zhenkang	3500	3.84	1602.96	81.14	1089.09	0.68	Terrestrial	Grassy slope	Non-endemic
Malaxis monophyllos	Weixi	3500	4.43	970.70	69.95	1021.03	1.05	Terrestrial	Meadow	Non-endemic
Malaxis monophyllos	Deqin	3510	4.11	639.48	70.85	896.66	1.40	Terrestrial	Shrubland	Non-endemic
Malaxis monophyllos	Weixi	3600	3.83	970.70	69.95	1021.03	1.05	Terrestrial	Grassy slope	Non-endemic
Malaxis monophyllos	Eryuan	3780	3.85	745.16	68.27	1210.94	1.63	Terrestrial	Grassy slope	Non-endemic
Malaxis monophyllos	Deqin	3800	2.37	639.48	70.85	896.66	1.40	Terrestrial	Grassy slope	Non-endemic
Malaxis monophyllos	Gongshan	3800	1.28	1738.42	78.47	860.43	0.50	Terrestrial	Grassy slope	Non-endemic
Malaxis monophyllos	Lijiang	3800	4.27	982.53	63.24	1077.65	1.10	Terrestrial	Shrubland	Non-endemic
Malaxis monophyllos	Zhongdian	3950	1.74	641.73	69.05	914.89	1.43	Terrestrial	Meadow	Non-endemic
Malaxis orbicularis	Jinghong	1700	15.52	1161.08	80.46	1256.19	1.08	Terrestrial	Dense forest	Endemic
Malaxis ovalisepala	Mengla	850	20.10	1514.70	84.25	1146.68	0.76	Terrestrial	Dense forest	Non-endemic
Malaxis purpurea	Menghai	1300	17.76	1314.38	80.83	1150.91	0.88	Terrestrial	Dense forest	Non-endemic
Malaxis purpurea	Menghai	1800	14.76	1314.38	80.83	1150.91	0.88	Terrestrial	Dense forest	Non-endemic
Monomeria barbata	Malipo	1000	18.26	1063.54	85.83	1053.97	0.99	Epiphyte	Dense forest	Non-endemic

Monomeria barbata	Gongshan	1300	16.28	1738.42	78.47	860.43	0.50	Epiphyte	Dense forest	Non-endemic
Monomeria barbata	Malipo	1500	15.26	1063.54	85.83	1053.97	0.99	Epiphyte	Dense forest	Non-endemic
Monomeria barbata	Malipo	1500	15.26	1063.54	85.83	1053.97	0.99	Epiphyte	Dense forest	Non-endemic
Myrmechis gracilis	Jingdong	2400	11.18	1128.40	76.80	1142.11	1.01	Terrestrial	Dense forest	Non-endemic
Myrmechis japonica	Gongshan	2600	8.48	1738.42	78.47	860.43	0.50	Epiphyte	Dense forest	Non-endemic
Myrmechis japonica	Yongde	2900	9.73	1266.25	69.00	1283.63	1.01	Epiphyte	Dense forest	Non-endemic
Myrmechis japonica	Yongde	2950	9.43	1266.25	69.00	1283.63	1.01	Epiphyte	Dense forest	Non-endemic
Myrmechis pumila	Jingdong	2430	11.00	1128.40	76.80	1142.11	1.01	Terrestrial	Dense forest	Non-endemic
Myrmechis urceolata	Pingbian	2200	11.62	1648.57	86.34	990.16	0.60	Terrestrial	Dense forest	Non-endemic
Myrmechis urceolata	Qiaojia	2300	9.82	878.15	76.01	1034.97	1.18	Terrestrial	Dense forest	Non-endemic
Myrmechis urceolata	Yongde	3060	8.77	1266.25	69.00	1283.63	1.01	Terrestrial	Grassy slope	Non-endemic
Myrmechis urceolata	Zhongdian	3200	6.24	641.73	69.05	914.89	1.43	Terrestrial	Grassy slope	Non-endemic
Neogyna gardneriana	Malipo	1600	14.66	1063.54	85.83	1053.97	0.99	Epiphyte	Dense forest	Non-endemic
Neogyna gardneriana	Luchun	1600	17.10	2013.54	78.64	1151.56	0.57	Epiphyte	Dense forest	Non-endemic
Neogyna gardneriana	Lincang	1700	16.32	1165.84	72.08	1167.73	1.00	Epiphyte	Dense forest	Non-endemic
Neogyna gardneriana	Longling	1700	13.92	2098.66	84.65	973.45	0.46	Epiphyte	Dense forest	Non-endemic
Neogyna gardneriana	Jingdong	1700	15.38	1128.40	76.80	1142.11	1.01	Epiphyte	Dense forest	Non-endemic
Neogyna gardneriana	Hekou	1900	12.35	1768.58	84.25	1166.26	0.66	Epiphyte	Dense forest	Non-endemic
Neogyna gardneriana	Jingdong	2000	13.58	1128.40	76.80	1142.11	1.01	Epiphyte	Dense forest	Non-endemic
Neogyna gardneriana	Jingdong	2200	12.38	1128.40	76.80	1142.11	1.01	Epiphyte	Open site	Non-endemic
Neottia acuminata	Gongshan	2350	9.98	1738.42	78.47	860.43	0.50	Saprophyte	Dense forest	Non-endemic
Neottia acuminata	Gongshan	2800	7.28	1738.42	78.47	860.43	0.50	Saprophyte	Dense forest	Non-endemic
Neottia acuminata	Heqing	3000	8.79	977.00	65.16	1211.84	1.24	Saprophyte	Dense forest	Non-endemic
Neottia acuminata	Lijiang	3000	9.07	982.53	63.24	1077.65	1.10	Saprophyte	Grassy slope	Non-endemic
Neottia acuminata	Zhongdian	3440	4.80	641.73	69.05	914.89	1.43	Saprophyte	Dense forest	Non-endemic
Neottia acuminata	Zhongdian	3850	2.34	641.73	69.05	914.89	1.43	Saprophyte	Dense forest	Non-endemic
Neottia acuminata	Zhongdian	3960	1.68	641.73	69.05	914.89	1.43	Saprophyte	Dense forest	Non-endemic
Neottia acuminata	Zhongdian	4000	1.44	641.73	69.05	914.89	1.43	Saprophyte	Dense forest	Non-endemic
Neottia acuminata	Zhongdian	4100	0.84	641.73	69.05	914.89	1.43	Saprophyte	Dense forest	Non-endemic
Neottia listeroides	Zhongdian	2800	8.64	641.73	69.05	914.89	1.43	Saprophyte	Dense forest	Non-endemic
Neottia listeroides	Gongshan	3500	3.08	1738.42	78.47	860.43	0.50	Saprophyte	Dense forest	Non-endemic
Neottia megalochila	Lijiang	3250	7.57	982.53	63.24	1077.65	1.10	Saprophyte	Grassy slope	Non-endemic
Neottia megalochila	Lijiang	3300	7.27	982.53	63.24	1077.65	1.10	Saprophyte	Dense forest	Non-endemic
Neottia megalochila	Zhongdian	3400	5.04	641.73	69.05	914.89	1.43	Saprophyte	Dense forest	Non-endemic
Neottia megalochila	Gongshan	3500	3.08	1738.42	78.47	860.43	0.50	Saprophyte	Dense forest	Non-endemic
Neottia megalochila	Lijiang	3800	4.27	982.53	63.24	1077.65	1.10	Saprophyte	Meadow	Non-endemic

Neottia megalochila	Zhongdian	3900	2.04	641.73	69.05	914.89	1.43	Saprophyte	Open site	Non-endemic
Neottianthe calcicola	Deqin	3000	7.17	639.48	70.85	896.66	1.40	Terrestrial	Dense forest	Non-endemic
Neottianthe calcicola	Zhongdian	3100	6.84	641.73	69.05	914.89	1.43	Terrestrial	Forest edge	Non-endemic
Neottianthe calcicola	Lijiang	3170	8.05	982.53	63.24	1077.65	1.10	Terrestrial	Open site	Non-endemic
Neottianthe calcicola	Heqing	3300	6.99	977.00	65.16	1211.84	1.24	Terrestrial	Sparse forest	Non-endemic
Neottianthe calcicola	Heqing	3400	6.39	977.00	65.16	1211.84	1.24	Terrestrial	Sparse forest	Non-endemic
Neottianthe calcicola	Zhongdian	3470	4.62	641.73	69.05	914.89	1.43	Terrestrial	Grassy slope	Non-endemic
Neottianthe calcicola	Zhongdian	3500	4.44	641.73	69.05	914.89	1.43	Terrestrial	Grassy slope	Non-endemic
Neottianthe calcicola	Zhongdian	3600	3.84	641.73	69.05	914.89	1.43	Terrestrial	Meadow	Non-endemic
Neottianthe calcicola	Zhongdian	3700	3.24	641.73	69.05	914.89	1.43	Terrestrial	Forest edge	Non-endemic
Neottianthe calcicola	Heqing	3800	3.99	977.00	65.16	1211.84	1.24	Terrestrial	Grassy slope	Non-endemic
Neottianthe calcicola	Zhongdian	3800	2.64	641.73	69.05	914.89	1.43	Terrestrial	Meadow	Non-endemic
Neottianthe camptoceras	Baoshan	3100	7.16	992.38	73.87	1160.15	1.17	Terrestrial	Dense forest	Non-endemic
Neottianthe cucullata	Deqin	3400	4.77	639.48	70.85	896.66	1.40	Terrestrial	Shrubland	Non-endemic
Neottianthe gymnadeninoides	Deqin	3000	7.17	639.48	70.85	896.66	1.40	Terrestrial	Dense forest	Non-endemic
Neottianthe luteola	Gongshan	3000	6.08	1738.42	78.47	860.43	0.50	Terrestrial	Grassy slope	Endemic
Neottianthe monophylla	Lijiang	2600	11.47	982.53	63.24	1077.65	1.10	Terrestrial	Dense forest	Non-endemic
Neottianthe oblonga	Zhongdian	3100	6.84	641.73	69.05	914.89	1.43	Terrestrial	Forest edge	Non-endemic
Neottianthe secundiflora	Heqing	3400	6.39	977.00	65.16	1211.84	1.24	Terrestrial	Grassy slope	Non-endemic
Nervilia aragoana	Luquan	1960	13.96	965.09	73.60	1160.89	1.20	Terrestrial	Shrubland	Non-endemic
Nervilia aragoana	Luquan	2300	11.92	965.09	73.60	1160.89	1.20	Terrestrial	Shrubland	Non-endemic
Nervilia fordii	Hekou	1950	12.05	1768.58	84.25	1166.26	0.66	Terrestrial	Forest edge	Non-endemic
Nervilia mackinnonii	Luoping	1350	15.94	1686.24	82.51	1040.03	0.62	Terrestrial	Dense forest	Non-endemic
Nervilia mackinnonii	Xichou	1650	15.03	1267.54	82.98	985.97	0.78	Terrestrial	Dense forest	Non-endemic
Nervilia plicata	Jinghong	650	21.82	1161.08	80.46	1256.19	1.08	Terrestrial	Dense forest	Non-endemic
Nervilia plicata	Jinghong	650	21.82	1161.08	80.46	1256.19	1.08	Terrestrial	Dense forest	Non-endemic
Nervilia plicata	Menghai	1000	19.56	1314.38	80.83	1150.91	0.88	Terrestrial	Shrubland	Non-endemic
Nervilia plicata	Jingdong	1100	18.98	1128.40	76.80	1142.11	1.01	Terrestrial	Dense forest	Non-endemic
NerviliaaragoanaGaud.	Luquan	1960	13.96	965.09	73.60	1160.89	1.20	Terrestrial	Grassy slope	Non-endemic
Oberonia acaulis	Luchun	1580	17.22	2013.54	78.64	1151.56	0.57	Epiphyte	Dense forest	Non-endemic
Oberonia acaulis var.luchunensis	Dali	2100	14.22	1082.70	68.61	1256.27	1.16	Epiphyte	Dense forest	Endemic
Oberonia acaulis var.luchunensis	Luchun	2400	12.30	2013.54	78.64	1151.56	0.57	Epiphyte	Forest edge	Endemic
Oberonia caulescens	Xichou	1200	17.73	1267.54	82.98	985.97	0.78	Epiphyte	Dense forest	Non-endemic
Oberonia caulescens	Yanshan	1200	18.26	1003.57	79.42	1172.21	1.17	Epiphyte	Dense forest	Non-endemic
Oberonia caulescens	Pingbian	1600	15.22	1648.57	86.34	990.16	0.60	Epiphyte	Dense forest	Non-endemic
Oberonia caulescens	Luxi	1750	14.58	1650.33	79.33	1183.39	0.72	Epiphyte	Dense forest	Non-endemic

Oberonia caulescens	Wenshan	1750	15.16	988.87	76.70	1272.98	1.29	Epiphyte	Dense forest	Non-endemic
Oberonia caulescens	Menghai	1800	14.76	1314.38	80.83	1150.91	0.88	Epiphyte	Dense forest	Non-endemic
Oberonia caulescens	Mengzi	1800	15.66	855.35	71.82	1434.56	1.68	Epiphyte	Shrubland	Non-endemic
Oberonia caulescens	Gongshan	1900	12.68	1738.42	78.47	860.43	0.50	Epiphyte	Dense forest	Non-endemic
Oberonia caulescens	Gongshan	2000	12.08	1738.42	78.47	860.43	0.50	Epiphyte	Dense forest	Non-endemic
Oberonia caulescens	Eshan	2000	13.21	938.39	78.62	1123.48	1.20	Epiphyte	Shrubland	Non-endemic
Oberonia caulescens	Jingdong	2100	12.98	1128.40	76.80	1142.11	1.01	Epiphyte	Dense forest	Non-endemic
Oberonia caulescens	Jingdong	2200	12.38	1128.40	76.80	1142.11	1.01	Epiphyte	Dense forest	Non-endemic
Oberonia caulescens	Fengqing	2400	11.80	1352.80	73.24	1172.52	0.87	Epiphyte	Dense forest	Non-endemic
Oberonia caulescens	Lushui	2500	11.05	1195.57	70.63	911.65	0.76	Epiphyte	Dense forest	Non-endemic
Oberonia caulescens	Jingdong	2600	9.98	1128.40	76.80	1142.11	1.01	Epiphyte	Dense forest	Non-endemic
Oberonia caulescens	Deqin	2700	8.97	639.48	70.85	896.66	1.40	Epiphyte	Shrubland	Non-endemic
Oberonia ensiformis	Mengla	720	20.88	1514.70	84.25	1146.68	0.76	Epiphyte	Dense forest	Non-endemic
Oberonia ensiformis	Mengla	750	20.70	1514.70	84.25	1146.68	0.76	Epiphyte	Dense forest	Non-endemic
Oberonia ensiformis	Jinping	800	20.69	2305.17	83.81	1030.46	0.45	Epiphyte	Dense forest	Non-endemic
Oberonia ensiformis	Jinghong	950	20.02	1161.08	80.46	1256.19	1.08	Epiphyte	Dense forest	Non-endemic
Oberonia ensiformis	Jinghong	1000	19.72	1161.08	80.46	1256.19	1.08	Epiphyte	Shrubland	Non-endemic
Oberonia ensiformis	Mengla	1100	18.60	1514.70	84.25	1146.68	0.76	Epiphyte	Dense forest	Non-endemic
Oberonia ensiformis	Mengla	1100	18.60	1514.70	84.25	1146.68	0.76	Epiphyte	Dense forest	Non-endemic
Oberonia ensiformis	Jinghong	1150	18.82	1161.08	80.46	1256.19	1.08	Epiphyte	Dense forest	Non-endemic
Oberonia ensiformis	Jinghong	1150	18.82	1161.08	80.46	1256.19	1.08	Epiphyte	Dense forest	Non-endemic
Oberonia ensiformis	Malipo	1150	17.36	1063.54	85.83	1053.97	0.99	Epiphyte	Dense forest	Non-endemic
Oberonia ensiformis	Jinghong	1200	18.52	1161.08	80.46	1256.19	1.08	Epiphyte	Dense forest	Non-endemic
Oberonia ensiformis	Jinghong	1200	18.52	1161.08	80.46	1256.19	1.08	Epiphyte	Dense forest	Non-endemic
Oberonia ensiformis	Menghai	1500	16.56	1314.38	80.83	1150.91	0.88	Epiphyte	Dense forest	Non-endemic
Oberonia falconeri	Jinghong	670	21.70	1161.08	80.46	1256.19	1.08	Epiphyte	Sparse forest	Non-endemic
Oberonia falconeri	Xichou	700	20.73	1267.54	82.98	985.97	0.78	Epiphyte	Dense forest	Non-endemic
Oberonia falconeri	Jinghong	860	20.56	1161.08	80.46	1256.19	1.08	Epiphyte	Dense forest	Non-endemic
Oberonia falconeri	Mengla	860	20.04	1514.70	84.25	1146.68	0.76	Epiphyte	Sparse forest	Non-endemic
Oberonia falconeri	Jinghong	910	20.26	1161.08	80.46	1256.19	1.08	Epiphyte	Dense forest	Non-endemic
Oberonia falconeri	Jinghong	950	20.02	1161.08	80.46	1256.19	1.08	Epiphyte	Shrubland	Non-endemic
Oberonia falconeri	Jinghong	1020	19.60	1161.08	80.46	1256.19	1.08	Epiphyte	Dense forest	Non-endemic
Oberonia falconeri	Jinghong	1150	18.82	1161.08	80.46	1256.19	1.08	Epiphyte	Dense forest	Non-endemic
Oberonia falconeri	Mengla	1500	16.20	1514.70	84.25	1146.68	0.76	Epiphyte	Dense forest	Non-endemic
Oberonia falconeri	Longling	2500	9.12	2098.66	84.65	973.45	0.46	Epiphyte	Dense forest	Non-endemic
Oberonia gammiei	Jinghong	860	20.56	1161.08	80.46	1256.19	1.08	Epiphyte	Dense forest	Non-endemic

Oberonia integerrima	Yongde	1600	17.53	1266.25	69.00	1283.63	1.01	Epiphyte	Dense forest	Non-endemic
Oberonia iridifolia	Yingjiang	1300	16.59	1505.33	79.98	1158.19	0.77	Epiphyte	Dense forest	Non-endemic
Oberonia jenkinsiana	Menghai	1100	18.96	1314.38	80.83	1150.91	0.88	Epiphyte	Sparse forest	Non-endemic
Oberonia jenkinsiana	Gongshan	1300	16.28	1738.42	78.47	860.43	0.50	Epiphyte	Dense forest	Non-endemic
Oberonia jenkinsiana	Menghai	1300	17.76	1314.38	80.83	1150.91	0.88	Epiphyte	Sparse forest	Non-endemic
Oberonia jenkinsiana	Jinghong	1500	16.72	1161.08	80.46	1256.19	1.08	Epiphyte	Dense forest	Non-endemic
Oberonia jenkinsiana	Pingbian	1500	15.82	1648.57	86.34	990.16	0.60	Epiphyte	Dense forest	Non-endemic
Oberonia jenkinsiana	Menghai	1520	16.44	1314.38	80.83	1150.91	0.88	Epiphyte	Dense forest	Non-endemic
Oberonia jenkinsiana	Menghai	1530	16.38	1314.38	80.83	1150.91	0.88	Epiphyte	Dense forest	Non-endemic
Oberonia jenkinsiana	Yongde	1600	17.53	1266.25	69.00	1283.63	1.01	Epiphyte	Dense forest	Non-endemic
Oberonia jenkinsiana	Jingdong	1700	15.38	1128.40	76.80	1142.11	1.01	Epiphyte	Dense forest	Non-endemic
Oberonia jenkinsiana	Luchun	1760	16.14	2013.54	78.64	1151.56	0.57	Epiphyte	Dense forest	Non-endemic
Oberonia jenkinsiana	Gongshan	2100	11.48	1738.42	78.47	860.43	0.50	Epiphyte	Dense forest	Non-endemic
Oberonia jenkinsiana	Tengchong	2300	11.20	1501.45	78.06	857.93	0.57	Epiphyte	Dense forest	Non-endemic
Oberonia jenkinsiana	Zhenkang	2700	8.64	1602.96	81.14	1089.09	0.68	Epiphyte	Dense forest	Non-endemic
Oberonia kwangsiensis	Malipo	1150	17.36	1063.54	85.83	1053.97	0.99	Epiphyte	Dense forest	Non-endemic
Oberonia latipetala	Lancang	1500	16.70	1596.50	77.88	1183.80	0.74	Epiphyte	Dense forest	Endemic
Oberonia latipetala	Jingdong	1800	14.78	1128.40	76.80	1142.11	1.01	Epiphyte	Dense forest	Endemic
Oberonia latipetala	Jingdong	1800	14.78	1128.40	76.80	1142.11	1.01	Epiphyte	Dense forest	Endemic
Oberonia latipetala	Gongshan	1900	12.68	1738.42	78.47	860.43	0.50	Epiphyte	Dense forest	Endemic
Oberonia latipetala	Jingdong	1950	13.88	1128.40	76.80	1142.11	1.01	Epiphyte	Dense forest	Endemic
Oberonia latipetala	Jingdong	2100	12.98	1128.40	76.80	1142.11	1.01	Epiphyte	Dense forest	Endemic
Oberonia latipetala	Jingdong	2400	11.18	1128.40	76.80	1142.11	1.01	Epiphyte	Dense forest	Endemic
Oberonia mannii	Pingbian	1600	15.22	1648.57	86.34	990.16	0.60	Epiphyte	Dense forest	Non-endemic
Oberonia menghaiensis	Menghai	1800	14.76	1314.38	80.83	1150.91	0.88	Epiphyte	Dense forest	Endemic
Oberonia menglaensis	Mengla	750	20.70	1514.70	84.25	1146.68	0.76	Epiphyte	Dense forest	Endemic
Oberonia myosurus	Yanshan	1200	18.26	1003.57	79.42	1172.21	1.17	Epiphyte	Dense forest	Non-endemic
Oberonia myosurus	Menghai	1500	16.56	1314.38	80.83	1150.91	0.88	Epiphyte	Shrubland	Non-endemic
Oberonia myosurus	Xichou	1540	15.69	1267.54	82.98	985.97	0.78	Epiphyte	Dense forest	Non-endemic
Oberonia pachyrachis	Yongde	1900	15.73	1266.25	69.00	1283.63	1.01	Epiphyte	Sparse forest	Non-endemic
Oberonia pachyrachis	Lancang	2100	13.10	1596.50	77.88	1183.80	0.74	Epiphyte	Dense forest	Non-endemic
Oberonia pyrulifera	Fengqing	1700	16.00	1352.80	73.24	1172.52	0.87	Epiphyte	Dense forest	Non-endemic
Oberonia pyrulifera	Fengqing	2100	13.60	1352.80	73.24	1172.52	0.87	Epiphyte	Dense forest	Non-endemic
Oberonia pyrulifera	Zhenkang	2300	11.04	1602.96	81.14	1089.09	0.68	Epiphyte	Dense forest	Non-endemic
Orchis brevicalcarata	Dali	2700	10.62	1082.70	68.61	1256.27	1.16	Epiphyte	Dense forest	Non-endemic
Orchis brevicalcarata	Dali	1500	17.82	1082.70	68.61	1256.27	1.16	Terrestrial	Grassy slope	Non-endemic

Orchis brevicalcarata	Dali	2350	12.72	1082.70	68.61	1256.27	1.16	Terrestrial	Grassy slope	Non-endemic
Orchis brevicalcarata	Lijiang	2500	12.07	982.53	63.24	1077.65	1.10	Terrestrial	Dense forest	Non-endemic
Orchis brevicalcarata	Yangbi	2500	10.99	1044.23	72.19	1161.15	1.11	Terrestrial	Dense forest	Non-endemic
Orchis brevicalcarata	Dali	2600	11.22	1082.70	68.61	1256.27	1.16	Terrestrial	Grassy slope	Non-endemic
Orchis brevicalcarata	Lijiang	2700	10.87	982.53	63.24	1077.65	1.10	Terrestrial	Grassy slope	Non-endemic
Orchis brevicalcarata	Binchuan	2900	9.30	573.87	62.93	1418.42	2.47	Terrestrial	Meadow	Non-endemic
Orchis brevicalcarata	Lijiang	3100	8.47	982.53	63.24	1077.65	1.10	Terrestrial	Dense forest	Non-endemic
Orchis brevicalcarata	Dayao	3200	7.73	810.80	65.01	1427.38	1.76	Terrestrial	Dense forest	Non-endemic
Orchis brevicalcarata	Lijiang	3200	7.87	982.53	63.24	1077.65	1.10	Terrestrial	Grassy slope	Non-endemic
Orchis brevicalcarata	Lijiang	3300	7.27	982.53	63.24	1077.65	1.10	Terrestrial	Sparse forest	Non-endemic
Orchis brevicalcarata	Zhongdian	3500	4.44	641.73	69.05	914.89	1.43	Terrestrial	Grassy slope	Non-endemic
Orchis chrysea	Gongshan	3400	3.68	1738.42	78.47	860.43	0.50	Epiphyte	Forest edge	Non-endemic
Orchis chrysea	Gongshan	3800	1.28	1738.42	78.47	860.43	0.50	Terrestrial	Meadow	Non-endemic
Orchis chusua	Luchun	850	21.60	2013.54	78.64	1151.56	0.57	Terrestrial	Dense forest	Non-endemic
Orchis chusua	Daguan	1200	14.62	987.00	80.05	896.37	0.91	Terrestrial	Dense forest	Non-endemic
Orchis chusua	Weixi	1850	14.33	970.70	69.95	1021.03	1.05	Terrestrial	Grassy slope	Non-endemic
Orchis chusua	Kunming	1900	14.88	1019.14	72.30	1197.62	1.18	Terrestrial	Grassy slope	Non-endemic
Orchis chusua	Lijiang	2000	15.07	982.53	63.24	1077.65	1.10	Terrestrial	Meadow	Non-endemic
Orchis chusua	Yongde	2010	15.07	1266.25	69.00	1283.63	1.01	Terrestrial	Dense forest	Non-endemic
Orchis chusua	Kunming	2100	13.68	1019.14	72.30	1197.62	1.18	Terrestrial	Grassy slope	Non-endemic
Orchis chusua	Dali	2230	13.44	1082.70	68.61	1256.27	1.16	Terrestrial	Dense forest	Non-endemic
Orchis chusua	Eryuan	2230	13.15	745.16	68.27	1210.94	1.63	Terrestrial	Grassy slope	Non-endemic
Orchis chusua	Eryuan	2300	12.73	745.16	68.27	1210.94	1.63	Terrestrial	Sparse forest	Non-endemic
Orchis chusua	Yunlong	2450	11.35	1195.57	70.63	911.65	0.76	Terrestrial	Shrubland	Non-endemic
Orchis chusua	Lijiang	2500	12.07	982.53	63.24	1077.65	1.10	Terrestrial	Grassy slope	Non-endemic
Orchis chusua	Lijiang	2500	12.07	982.53	63.24	1077.65	1.10	Terrestrial	Grassy slope	Non-endemic
Orchis chusua	Lijiang	2500	12.07	982.53	63.24	1077.65	1.10	Terrestrial	Meadow	Non-endemic
Orchis chusua	Gongshan	2500	9.08	1738.42	78.47	860.43	0.50	Terrestrial	Meadow	Non-endemic
Orchis chusua	Lijiang	2560	11.71	982.53	63.24	1077.65	1.10	Terrestrial	Dense forest	Non-endemic
Orchis chusua	Heqing	2600	11.19	977.00	65.16	1211.84	1.24	Terrestrial	Dense forest	Non-endemic
Orchis chusua	Dali	2600	11.22	1082.70	68.61	1256.27	1.16	Terrestrial	Grassy slope	Non-endemic
Orchis chusua	Lijiang	2600	11.47	982.53	63.24	1077.65	1.10	Terrestrial	Meadow	Non-endemic
Orchis chusua	Zhongdian	2650	9.54	641.73	69.05	914.89	1.43	Terrestrial	Meadow	Non-endemic
Orchis chusua	Deqin	2700	8.97	639.48	70.85	896.66	1.40	Terrestrial	Grassy slope	Non-endemic
Orchis chusua	Lijiang	2700	10.87	982.53	63.24	1077.65	1.10	Terrestrial	Shrubland	Non-endemic
Orchis chusua	Gongshan	2800	7.28	1738.42	78.47	860.43	0.50	Terrestrial	Dense forest	Non-endemic

Orchis chusua	Lijiang	2800	10.27	982.53	63.24	1077.65	1.10	Terrestrial	Forest edge	Non-endemic
Orchis chusua	Lijiang	2800	10.27	982.53	63.24	1077.65	1.10	Terrestrial	Grassy slope	Non-endemic
Orchis chusua	Zhongdian	2800	8.64	641.73	69.05	914.89	1.43	Terrestrial	Grassy slope	Non-endemic
Orchis chusua	Dali	2800	10.02	1082.70	68.61	1256.27	1.16	Terrestrial	Open site	Non-endemic
Orchis chusua	Zhenkang	2850	7.74	1602.96	81.14	1089.09	0.68	Terrestrial	Grassy slope	Non-endemic
Orchis chusua	Zhongdian	2900	8.04	641.73	69.05	914.89	1.43	Terrestrial	Dense forest	Non-endemic
Orchis chusua	Zhongdian	2900	8.04	641.73	69.05	914.89	1.43	Terrestrial	Forest edge	Non-endemic
Orchis chusua	Jingdong	2900	8.18	1128.40	76.80	1142.11	1.01	Terrestrial	Grassy slope	Non-endemic
Orchis chusua	Dongchuan	2980	7.95	1021.73	71.65	1186.61	1.16	Terrestrial	Meadow	Non-endemic
Orchis chusua	Lijiang	3000	9.07	982.53	63.24	1077.65	1.10	Terrestrial	Forest edge	Non-endemic
Orchis chusua	Lijiang	3000	9.07	982.53	63.24	1077.65	1.10	Terrestrial	Grassy slope	Non-endemic
Orchis chusua	Deqin	3000	7.17	639.48	70.85	896.66	1.40	Terrestrial	Grassy slope	Non-endemic
Orchis chusua	Gongshan	3000	6.08	1738.42	78.47	860.43	0.50	Terrestrial	Grassy slope	Non-endemic
Orchis chusua	Weixi	3000	7.43	970.70	69.95	1021.03	1.05	Terrestrial	Meadow	Non-endemic
Orchis chusua	Dali	3000	8.82	1082.70	68.61	1256.27	1.16	Terrestrial	Open site	Non-endemic
Orchis chusua	Dongchuan	3000	7.83	1021.73	71.65	1186.61	1.16	Terrestrial	Shrubland	Non-endemic
Orchis chusua	Lijiang	3000	9.07	982.53	63.24	1077.65	1.10	Terrestrial	Sparse forest	Non-endemic
Orchis chusua	Eryuan	3000	8.53	745.16	68.27	1210.94	1.63	Terrestrial	Sparse forest	Non-endemic
Orchis chusua	Dayao	3100	8.33	810.80	65.01	1427.38	1.76	Terrestrial	Meadow	Non-endemic
Orchis chusua	Deqin	3100	6.57	639.48	70.85	896.66	1.40	Terrestrial	Sparse forest	Non-endemic
Orchis chusua	Lijiang	3140	8.23	982.53	63.24	1077.65	1.10	Terrestrial	Grassy slope	Non-endemic
Orchis chusua	Lijiang	3145	8.20	982.53	63.24	1077.65	1.10	Terrestrial	Shrubland	Non-endemic
Orchis chusua	Lushui	3150	7.15	1195.57	70.63	911.65	0.76	Terrestrial	Meadow	Non-endemic
Orchis chusua	Lanping	3200	6.09	989.98	74.21	939.09	0.95	Terrestrial	Grassy slope	Non-endemic
Orchis chusua	Dali	3200	7.62	1082.70	68.61	1256.27	1.16	Terrestrial	Meadow	Non-endemic
Orchis chusua	Zhongdian	3200	6.24	641.73	69.05	914.89	1.43	Terrestrial	Meadow	Non-endemic
Orchis chusua	Dongchuan	3200	6.63	1021.73	71.65	1186.61	1.16	Terrestrial	Shrubland	Non-endemic
Orchis chusua	Deqin	3300	5.37	639.48	70.85	896.66	1.40	Terrestrial	Grassy slope	Non-endemic
Orchis chusua	Deqin	3300	5.37	639.48	70.85	896.66	1.40	Terrestrial	Grassy slope	Non-endemic
Orchis chusua	Weixi	3300	5.63	970.70	69.95	1021.03	1.05	Terrestrial	Grassy slope	Non-endemic
Orchis chusua	Weixi	3400	5.03	970.70	69.95	1021.03	1.05	Terrestrial	Grassy slope	Non-endemic
Orchis chusua	Weixi	3400	5.03	970.70	69.95	1021.03	1.05	Terrestrial	Grassy slope	Non-endemic
Orchis chusua	Zhenkang	3400	4.44	1602.96	81.14	1089.09	0.68	Terrestrial	Grassy slope	Non-endemic
Orchis chusua	Zhenkang	3400	4.44	1602.96	81.14	1089.09	0.68	Terrestrial	Grassy slope	Non-endemic
Orchis chusua	Zhongdian	3400	5.04	641.73	69.05	914.89	1.43	Terrestrial	Grassy slope	Non-endemic
Orchis chusua	Zhongdian	3400	5.04	641.73	69.05	914.89	1.43	Terrestrial	Meadow	Non-endemic

Orchis chusua	Yongde	3410	6.67	1266.25	69.00	1283.63	1.01	Terrestrial	Dense forest	Non-endemic
Orchis chusua	Weixi	3500	4.43	970.70	69.95	1021.03	1.05	Terrestrial	Grassy slope	Non-endemic
Orchis chusua	Weixi	3500	4.43	970.70	69.95	1021.03	1.05	Terrestrial	Grassy slope	Non-endemic
Orchis chusua	Deqin	3500	4.17	639.48	70.85	896.66	1.40	Terrestrial	Grassy slope	Non-endemic
Orchis chusua	Lanping	3500	4.29	989.98	74.21	939.09	0.95	Terrestrial	Grassy slope	Non-endemic
Orchis chusua	Lijiang	3500	6.07	982.53	63.24	1077.65	1.10	Terrestrial	Grassy slope	Non-endemic
Orchis chusua	Dali	3500	5.82	1082.70	68.61	1256.27	1.16	Terrestrial	Grassy slope	Non-endemic
Orchis chusua	Lijiang	3500	6.07	982.53	63.24	1077.65	1.10	Terrestrial	Grassy slope	Non-endemic
Orchis chusua	Weixi	3500	4.43	970.70	69.95	1021.03	1.05	Terrestrial	Meadow	Non-endemic
Orchis chusua	Deqin	3500	4.17	639.48	70.85	896.66	1.40	Terrestrial	Open site	Non-endemic
Orchis chusua	Eryuan	3500	5.53	745.16	68.27	1210.94	1.63	Terrestrial	Shrubland	Non-endemic
Orchis chusua	Dongchuan	3500	4.83	1021.73	71.65	1186.61	1.16	Terrestrial	Shrubland	Non-endemic
Orchis chusua	Deqin	3540	3.93	639.48	70.85	896.66	1.40	Terrestrial	Open site	Non-endemic
Orchis chusua	Lijiang	3550	5.77	982.53	63.24	1077.65	1.10	Terrestrial	Meadow	Non-endemic
Orchis chusua	Deqin	3600	3.57	639.48	70.85	896.66	1.40	Terrestrial	Dense forest	Non-endemic
Orchis chusua	Weixi	3600	3.83	970.70	69.95	1021.03	1.05	Terrestrial	Grassy slope	Non-endemic
Orchis chusua	Weixi	3600	3.83	970.70	69.95	1021.03	1.05	Terrestrial	Grassy slope	Non-endemic
Orchis chusua	Weixi	3600	3.83	970.70	69.95	1021.03	1.05	Terrestrial	Grassy slope	Non-endemic
Orchis chusua	Zhongdian	3600	3.84	641.73	69.05	914.89	1.43	Terrestrial	Grassy slope	Non-endemic
Orchis chusua	Zhongdian	3600	3.84	641.73	69.05	914.89	1.43	Terrestrial	Shrubland	Non-endemic
Orchis chusua	Zhongdian	3650	3.54	641.73	69.05	914.89	1.43	Terrestrial	Grassy slope	Non-endemic
Orchis chusua	Lijiang	3650	5.17	982.53	63.24	1077.65	1.10	Terrestrial	Grassy slope	Non-endemic
Orchis chusua	Zhongdian	3650	3.54	641.73	69.05	914.89	1.43	Terrestrial	Grassy slope	Non-endemic
Orchis chusua	Lijiang	3700	4.87	982.53	63.24	1077.65	1.10	Terrestrial	Meadow	Non-endemic
Orchis chusua	Huize	3720	3.05	799.98	71.46	1179.17	1.47	Terrestrial	Meadow	Non-endemic
Orchis chusua	Zhongdian	3750	2.94	641.73	69.05	914.89	1.43	Terrestrial	Shrubland	Non-endemic
Orchis chusua	Deqin	3800	2.37	639.48	70.85	896.66	1.40	Terrestrial	Dense forest	Non-endemic
Orchis chusua	Zhongdian	3800	2.64	641.73	69.05	914.89	1.43	Terrestrial	Grassy slope	Non-endemic
Orchis chusua	Lijiang	3800	4.27	982.53	63.24	1077.65	1.10	Terrestrial	Grassy slope	Non-endemic
Orchis chusua	Zhongdian	3800	2.64	641.73	69.05	914.89	1.43	Terrestrial	Grassy slope	Non-endemic
Orchis chusua	Zhongdian	3850	2.34	641.73	69.05	914.89	1.43	Terrestrial	Forest edge	Non-endemic
Orchis chusua	Zhongdian	3890	2.10	641.73	69.05	914.89	1.43	Terrestrial	Grassy slope	Non-endemic
Orchis chusua	Deqin	3900	1.77	639.48	70.85	896.66	1.40	Terrestrial	Grassy slope	Non-endemic
Orchis chusua	Dongchuan	3900	2.43	1021.73	71.65	1186.61	1.16	Terrestrial	Meadow	Non-endemic
Orchis chusua	Fugong	4000	0.06	1441.43	79.99	906.18	0.63	Terrestrial	Dense forest	Non-endemic
Orchis chusua	Deqin	4000	1.17	639.48	70.85	896.66	1.40	Terrestrial	Grassy slope	Non-endemic

Orchis chusua	Zhongdian	4000	1.44	641.73	69.05	914.89	1.43	Terrestrial	Grassy slope	Non-endemic
Orchis chusua var.nana	Eryuan	3650	4.63	745.16	68.27	1210.94	1.63	Terrestrial	Shrubland	Non-endemic
Orchis chusua var.nana	Zhongdian	4000	1.44	641.73	69.05	914.89	1.43	Terrestrial	Shrubland	Non-endemic
Orchis crenulata	Lijiang	3300	7.27	982.53	63.24	1077.65	1.10	Terrestrial	Open site	Endemic
Orchis diantha	Zhongdian	2800	8.64	641.73	69.05	914.89	1.43	Terrestrial	Grassy slope	Non-endemic
Orchis diantha	Lijiang	3000	9.07	982.53	63.24	1077.65	1.10	Terrestrial	Grassy slope	Non-endemic
Orchis diantha	Yangbi	3100	7.39	1044.23	72.19	1161.15	1.11	Terrestrial	Forest edge	Non-endemic
Orchis diantha	Weixi	3100	6.83	970.70	69.95	1021.03	1.05	Terrestrial	Grassy slope	Non-endemic
Orchis diantha	Lijiang	3300	7.27	982.53	63.24	1077.65	1.10	Terrestrial	Meadow	Non-endemic
Orchis diantha	Zhongdian	3400	5.04	641.73	69.05	914.89	1.43	Terrestrial	Shrubland	Non-endemic
Orchis diantha	Zhongdian	3440	4.80	641.73	69.05	914.89	1.43	Terrestrial	Grassy slope	Non-endemic
Orchis diantha	Gongshan	3500	3.08	1738.42	78.47	860.43	0.50	Terrestrial	Forest edge	Non-endemic
Orchis diantha	Weixi	3700	3.23	970.70	69.95	1021.03	1.05	Terrestrial	Grassy slope	Non-endemic
Orchis diantha	Weixi	3800	2.63	970.70	69.95	1021.03	1.05	Terrestrial	Meadow	Non-endemic
Orchis diantha	Zhongdian	3850	2.34	641.73	69.05	914.89	1.43	Terrestrial	Dense forest	Non-endemic
Orchis diantha	Weixi	3900	2.03	970.70	69.95	1021.03	1.05	Terrestrial	Grassy slope	Non-endemic
Orchis diantha	Zhongdian	3900	2.04	641.73	69.05	914.89	1.43	Terrestrial	Grassy slope	Non-endemic
Orchis diantha	Gongshan	4000	0.08	1738.42	78.47	860.43	0.50	Terrestrial	Meadow	Non-endemic
Orchis diantha	Zhongdian	4000	1.44	641.73	69.05	914.89	1.43	Terrestrial	Meadow	Non-endemic
Orchis diantha	Deqin	4300	-0.63	639.48	70.85	896.66	1.40	Terrestrial	Grassy slope	Non-endemic
Orchis latifolia	Zhongdian	3350	5.34	641.73	69.05	914.89	1.43	Terrestrial	Meadow	Non-endemic
Orchis limprichtii	Lijiang	4150	2.17	982.53	63.24	1077.65	1.10	Terrestrial	Grassy slope	Non-endemic
Orchis tschiliensis	Deqin	4100	0.57	639.48	70.85	896.66	1.40	Terrestrial	Grassy slope	Non-endemic
Orchis wardii	Zhongdian	2400	11.04	641.73	69.05	914.89	1.43	Terrestrial	Grassy slope	Non-endemic
Orchis wardii	Zhongdian	3700	3.24	641.73	69.05	914.89	1.43	Terrestrial	Meadow	Non-endemic
Orchis wardii	Zhongdian	3750	2.94	641.73	69.05	914.89	1.43	Terrestrial	Meadow	Non-endemic
Orchis wardii	Zhongdian	3800	2.64	641.73	69.05	914.89	1.43	Terrestrial	Meadow	Non-endemic
Oreorchis erythrochrysea	Jingdong	1900	14.18	1128.40	76.80	1142.11	1.01	Terrestrial	Grassy slope	Non-endemic
Oreorchis erythrochrysea	Yangbi	2120	13.27	1044.23	72.19	1161.15	1.11	Terrestrial	Dense forest	Non-endemic
Oreorchis erythrochrysea	Yangbi	2180	12.91	1044.23	72.19	1161.15	1.11	Terrestrial	Dense forest	Non-endemic
Oreorchis erythrochrysea	Jingdong	2200	12.38	1128.40	76.80	1142.11	1.01	Terrestrial	Dense forest	Non-endemic
Oreorchis erythrochrysea	Jingdong	2200	12.38	1128.40	76.80	1142.11	1.01	Terrestrial	Grassy slope	Non-endemic
Oreorchis erythrochrysea	Gongshan	2300	10.28	1738.42	78.47	860.43	0.50	Terrestrial	Dense forest	Non-endemic
Oreorchis erythrochrysea	Heqing	2400	12.39	977.00	65.16	1211.84	1.24	Terrestrial	Forest edge	Non-endemic
Oreorchis erythrochrysea	Lijiang	2400	12.67	982.53	63.24	1077.65	1.10	Terrestrial	Meadow	Non-endemic
Oreorchis erythrochrysea	Lijiang	2440	12.43	982.53	63.24	1077.65	1.10	Terrestrial	Meadow	Non-endemic

Oreorchis erythrochrysea	Lijiang	2600	11.47	982.53	63.24	1077.65	1.10	Terrestrial	Meadow	Non-endemic
Oreorchis erythrochrysea	Zhongdian	3000	7.44	641.73	69.05	914.89	1.43	Terrestrial	Dense forest	Non-endemic
Oreorchis erythrochrysea	Zhongdian	3000	7.44	641.73	69.05	914.89	1.43	Terrestrial	Dense forest	Non-endemic
Oreorchis erythrochrysea	Zhongdian	3000	7.44	641.73	69.05	914.89	1.43	Terrestrial	Dense forest	Non-endemic
Oreorchis erythrochrysea	Weixi	3000	7.43	970.70	69.95	1021.03	1.05	Terrestrial	Shrubland	Non-endemic
Oreorchis erythrochrysea	Zhongdian	3000	7.44	641.73	69.05	914.89	1.43	Terrestrial	Shrubland	Non-endemic
Oreorchis erythrochrysea	Lijiang	3010	9.01	982.53	63.24	1077.65	1.10	Terrestrial	Forest edge	Non-endemic
Oreorchis erythrochrysea	Dali	3100	8.22	1082.70	68.61	1256.27	1.16	Terrestrial	Shrubland	Non-endemic
Oreorchis erythrochrysea	Weixi	3200	6.23	970.70	69.95	1021.03	1.05	Terrestrial	Dense forest	Non-endemic
Oreorchis erythrochrysea	Weixi	3200	6.23	970.70	69.95	1021.03	1.05	Terrestrial	Dense forest	Non-endemic
Oreorchis erythrochrysea	Weixi	3200	6.23	970.70	69.95	1021.03	1.05	Terrestrial	Grassy slope	Non-endemic
Oreorchis erythrochrysea	Lijiang	3200	7.87	982.53	63.24	1077.65	1.10	Terrestrial	Meadow	Non-endemic
Oreorchis erythrochrysea	Lijiang	3200	7.87	982.53	63.24	1077.65	1.10	Terrestrial	Meadow	Non-endemic
Oreorchis erythrochrysea	Lijiang	3300	7.27	982.53	63.24	1077.65	1.10	Terrestrial	Dense forest	Non-endemic
Oreorchis erythrochrysea	Deqin	3350	5.07	639.48	70.85	896.66	1.40	Terrestrial	Shrubland	Non-endemic
Oreorchis erythrochrysea	Zhongdian	3450	4.74	641.73	69.05	914.89	1.43	Terrestrial	Dense forest	Non-endemic
Oreorchis erythrochrysea	Zhongdian	3500	4.44	641.73	69.05	914.89	1.43	Terrestrial	Dense forest	Non-endemic
Oreorchis erythrochrysea	Weixi	3500	4.43	970.70	69.95	1021.03	1.05	Terrestrial	Open site	Non-endemic
Oreorchis erythrochrysea	Deqin	3600	3.57	639.48	70.85	896.66	1.40	Terrestrial	Open site	Non-endemic
Oreorchis erythrochrysea	Zhongdian	3700	3.24	641.73	69.05	914.89	1.43	Terrestrial	Shrubland	Non-endemic
Oreorchis fargesii	Fuyuan	2500	9.87	1084.02	74.80	1096.10	1.01	Terrestrial	Shrubland	Non-endemic
Oreorchis nana	Zhongdian	3500	4.44	641.73	69.05	914.89	1.43	Epiphyte	Open site	Non-endemic
Oreorchis nana	Zhongdian	3800	2.64	641.73	69.05	914.89	1.43	Epiphyte	Open site	Non-endemic
Oreorchis patens	Gongshan	1800	13.28	1738.42	78.47	860.43	0.50	Terrestrial	Shrubland	Non-endemic
Oreorchis patens	Yangbi	2220	12.67	1044.23	72.19	1161.15	1.11	Terrestrial	Dense forest	Non-endemic
Oreorchis patens	Weixi	2300	11.63	970.70	69.95	1021.03	1.05	Terrestrial	Open site	Non-endemic
Oreorchis patens	Weixi	2400	11.03	970.70	69.95	1021.03	1.05	Terrestrial	Forest edge	Non-endemic
Oreorchis patens	Yongde	2420	12.61	1266.25	69.00	1283.63	1.01	Terrestrial	Forest edge	Non-endemic
Oreorchis patens	Dali	2600	11.22	1082.70	68.61	1256.27	1.16	Terrestrial	Dense forest	Non-endemic
Oreorchis patens	Fugong	2750	7.56	1441.43	79.99	906.18	0.63	Terrestrial	Open site	Non-endemic
Oreorchis patens	Lijiang	3050	8.77	982.53	63.24	1077.65	1.10	Terrestrial	Shrubland	Non-endemic
Ornithochilus difformis	Mengla	900	19.80	1514.70	84.25	1146.68	0.76	Epiphyte	Dense forest	Non-endemic
Ornithochilus difformis	Hekou	1050	17.45	1768.58	84.25	1166.26	0.66	Epiphyte	Dense forest	Non-endemic
Ornithochilus difformis	Eshan	1500	16.21	938.39	78.62	1123.48	1.20	Epiphyte	Dense forest	Non-endemic
Ornithochilus difformis	Menghai	1760	15.00	1314.38	80.83	1150.91	0.88	Epiphyte	Dense forest	Non-endemic
Ornithochilus difformis	Menghai	1800	14.76	1314.38	80.83	1150.91	0.88	Epiphyte	Dense forest	Non-endemic

Ornithochilus difformis	Menghai	1800	14.76	1314.38	80.83	1150.91	0.88	Epiphyte	Dense forest	Non-endemic
Ornithochilus difformis	Shuangbai	1920	15.36	942.53	72.21	1300.52	1.38	Epiphyte	Dense forest	Non-endemic
Ornithochilus difformis	Eryuan	2100	13.93	745.16	68.27	1210.94	1.63	Epiphyte	Dense forest	Non-endemic
Ornithochilus yingjiangensis	Yingjiang	1350	16.29	1505.33	79.98	1158.19	0.77	Epiphyte	Dense forest	Endemic
Otochilus alba	Luchun	1700	16.50	2013.54	78.64	1151.56	0.57	Epiphyte	Dense forest	Non-endemic
Otochilus alba	Jingdong	2300	11.78	1128.40	76.80	1142.11	1.01	Epiphyte	Dense forest	Non-endemic
Otochilus fuscus	Zhenkang	1200	17.64	1602.96	81.14	1089.09	0.68	Epiphyte	Dense forest	Non-endemic
Otochilus fuscus	Gongshan	1320	16.16	1738.42	78.47	860.43	0.50	Epiphyte	Dense forest	Non-endemic
Otochilus fuscus	Gongshan	1500	15.08	1738.42	78.47	860.43	0.50	Epiphyte	Dense forest	Non-endemic
Otochilus fuscus	Gongshan	1500	15.08	1738.42	78.47	860.43	0.50	Epiphyte	Dense forest	Non-endemic
Otochilus fuscus	Gengma	1700	15.47	1327.70	77.28	1181.78	0.89	Epiphyte	Dense forest	Non-endemic
Otochilus fuscus	Luxi	1750	14.58	1650.33	79.33	1183.39	0.72	Epiphyte	Dense forest	Non-endemic
Otochilus fuscus	Longling	1800	13.32	2098.66	84.65	973.45	0.46	Epiphyte	Dense forest	Non-endemic
Otochilus fuscus	Longling	1800	13.32	2098.66	84.65	973.45	0.46	Epiphyte	Dense forest	Non-endemic
Otochilus fuscus	Lushui	2000	14.05	1195.57	70.63	911.65	0.76	Epiphyte	Dense forest	Non-endemic
Otochilus fuscus	Jingdong	2100	12.98	1128.40	76.80	1142.11	1.01	Epiphyte	Dense forest	Non-endemic
Otochilus fuscus	Tengchong	2100	12.40	1501.45	78.06	857.93	0.57	Epiphyte	Dense forest	Non-endemic
Otochilus fuscus	Gongshan	2100	11.48	1738.42	78.47	860.43	0.50	Epiphyte	Dense forest	Non-endemic
Otochilus lancilabius	Yongde	2600	11.53	1266.25	69.00	1283.63	1.01	Epiphyte	Dense forest	Non-endemic
Otochilus porrectus	Malipo	1000	18.26	1063.54	85.83	1053.97	0.99	Epiphyte	Dense forest	Non-endemic
Otochilus porrectus	Xichou	1000	18.93	1267.54	82.98	985.97	0.78	Epiphyte	Dense forest	Non-endemic
Otochilus porrectus	Gongshan	1300	16.28	1738.42	78.47	860.43	0.50	Epiphyte	Dense forest	Non-endemic
Otochilus porrectus	Gongshan	1300	16.28	1738.42	78.47	860.43	0.50	Epiphyte	Dense forest	Non-endemic
Otochilus porrectus	Gongshan	1300	16.28	1738.42	78.47	860.43	0.50	Epiphyte	Forest edge	Non-endemic
Otochilus porrectus	Gongshan	1300	16.28	1738.42	78.47	860.43	0.50	Epiphyte	Shrubland	Non-endemic
Otochilus porrectus	Gongshan	1320	16.16	1738.42	78.47	860.43	0.50	Epiphyte	Dense forest	Non-endemic
Otochilus porrectus	Gongshan	1350	15.98	1738.42	78.47	860.43	0.50	Epiphyte	Dense forest	Non-endemic
Otochilus porrectus	Gongshan	1350	15.98	1738.42	78.47	860.43	0.50	Epiphyte	Dense forest	Non-endemic
Otochilus porrectus	Gongshan	1350	15.98	1738.42	78.47	860.43	0.50	Epiphyte	Dense forest	Non-endemic
Otochilus porrectus	Gongshan	1380	15.80	1738.42	78.47	860.43	0.50	Epiphyte	Dense forest	Non-endemic
Otochilus porrectus	Gongshan	1400	15.68	1738.42	78.47	860.43	0.50	Epiphyte	Dense forest	Non-endemic
Otochilus porrectus	Mengla	1400	16.80	1514.70	84.25	1146.68	0.76	Epiphyte	Dense forest	Non-endemic
Otochilus porrectus	Gongshan	1400	15.68	1738.42	78.47	860.43	0.50	Epiphyte	Dense forest	Non-endemic
Otochilus porrectus	Gongshan	1420	15.56	1738.42	78.47	860.43	0.50	Epiphyte	Dense forest	Non-endemic
Otochilus porrectus	Malipo	1450	15.56	1063.54	85.83	1053.97	0.99	Epiphyte	Dense forest	Non-endemic
Otochilus porrectus	Fugong	1500	15.06	1441.43	79.99	906.18	0.63	Epiphyte	Dense forest	Non-endemic

Otochilus porrectus	Gongshan	1500	15.08	1738.42	78.47	860.43	0.50	Epiphyte	Dense forest	Non-endemic
Otochilus porrectus	Gongshan	1500	15.08	1738.42	78.47	860.43	0.50	Epiphyte	Shrubland	Non-endemic
Otochilus porrectus	Gongshan	1500	15.08	1738.42	78.47	860.43	0.50	Epiphyte	Shrubland	Non-endemic
Otochilus porrectus	Longling	1510	15.06	2098.66	84.65	973.45	0.46	Epiphyte	Dense forest	Non-endemic
Otochilus porrectus	Fugong	1600	14.46	1441.43	79.99	906.18	0.63	Epiphyte	Dense forest	Non-endemic
Otochilus porrectus	Gongshan	1600	14.48	1738.42	78.47	860.43	0.50	Epiphyte	Dense forest	Non-endemic
Otochilus porrectus	Tengchong	1600	15.40	1501.45	78.06	857.93	0.57	Epiphyte	Shrubland	Non-endemic
Otochilus porrectus	Tengchong	1700	14.80	1501.45	78.06	857.93	0.57	Epiphyte	Dense forest	Non-endemic
Otochilus porrectus	Jingdong	1800	14.78	1128.40	76.80	1142.11	1.01	Epiphyte	Dense forest	Non-endemic
Otochilus porrectus	Gongshan	1800	13.28	1738.42	78.47	860.43	0.50	Epiphyte	Shrubland	Non-endemic
Otochilus porrectus	Luchun	1900	15.30	2013.54	78.64	1151.56	0.57	Epiphyte	Dense forest	Non-endemic
Otochilus porrectus	Tengchong	1900	13.60	1501.45	78.06	857.93	0.57	Epiphyte	Dense forest	Non-endemic
Otochilus porrectus	Gongshan	1920	12.56	1738.42	78.47	860.43	0.50	Epiphyte	Dense forest	Non-endemic
Otochilus porrectus	Pingbian	1940	13.18	1648.57	86.34	990.16	0.60	Epiphyte	Dense forest	Non-endemic
Otochilus porrectus	Fugong	2000	12.06	1441.43	79.99	906.18	0.63	Epiphyte	Dense forest	Non-endemic
Otochilus porrectus	Gongshan	2000	12.08	1738.42	78.47	860.43	0.50	Epiphyte	Dense forest	Non-endemic
Otochilus porrectus	Malipo	2000	12.26	1063.54	85.83	1053.97	0.99	Epiphyte	Dense forest	Non-endemic
Otochilus porrectus	Lushui	2000	14.05	1195.57	70.63	911.65	0.76	Epiphyte	Dense forest	Non-endemic
Otochilus porrectus	Malipo	2100	11.66	1063.54	85.83	1053.97	0.99	Epiphyte	Shrubland	Non-endemic
Otochilus porrectus	Luchun	2400	12.30	2013.54	78.64	1151.56	0.57	Epiphyte	Dense forest	Non-endemic
Otochilus porrectus	Luchun	2400	12.30	2013.54	78.64	1151.56	0.57	Epiphyte	Dense forest	Non-endemic
Otochilus porrectus	Fengqing	2450	11.50	1352.80	73.24	1172.52	0.87	Epiphyte	Dense forest	Non-endemic
Otochilus porrectus	Yongde	2550	11.83	1266.25	69.00	1283.63	1.01	Epiphyte	Dense forest	Non-endemic
Otochilus porrectus	Fengqing	2600	10.60	1352.80	73.24	1172.52	0.87	Epiphyte	Dense forest	Non-endemic
Otochilus porrectus	Lincang	2800	9.72	1165.84	72.08	1167.73	1.00	Epiphyte	Dense forest	Non-endemic
Otochilus porrectus	Gongshan	3000	6.08	1738.42	78.47	860.43	0.50	Epiphyte	Dense forest	Non-endemic
Pachystoma pubescens	Guangnan	150	23.35	1044.26	78.94	1090.35	1.04	Terrestrial	Grassy slope	Non-endemic
Pachystoma pubescens	Guangnan	800	19.45	1044.26	78.94	1090.35	1.04	Terrestrial	Grassy slope	Non-endemic
Pachystoma pubescens	Wenshan	1020	19.54	988.87	76.70	1272.98	1.29	Terrestrial	Grassy slope	Non-endemic
Panisea cavalerei	Jiangchuan	1780	15.32	872.74	74.72	1181.99	1.35	Epiphyte	Dense forest	Non-endemic
Panisea cavalerei	Songming	1980	13.71	1010.18	74.14	1134.24	1.12	Epiphyte	Dense forest	Non-endemic
Panisea cavalerei	Songming	2020	13.47	1010.18	74.14	1134.24	1.12	Epiphyte	Sparse forest	Non-endemic
Panisea tricallosa	Pingbian	1500	15.82	1648.57	86.34	990.16	0.60	Epiphyte	Dense forest	Non-endemic
Panisea unifolia	Maguan	1300	17.21	1330.52	83.25	1086.43	0.82	Epiphyte	Dense forest	Non-endemic
Panisea unifolia	Malipo	1400	15.86	1063.54	85.83	1053.97	0.99	Epiphyte	Dense forest	Non-endemic
Panisea unifolia	Lincang	2400	12.12	1165.84	72.08	1167.73	1.00	Epiphyte	Dense forest	Non-endemic

Panisea yunnanensis	Xichou	1200	17.73	1267.54	82.98	985.97	0.78	Epiphyte	Dense forest	Endemic
Panisea yunnanensis	Malipo	1500	15.26	1063.54	85.83	1053.97	0.99	Epiphyte	Dense forest	Endemic
Panisea yunnanensis	Malipo	1500	15.26	1063.54	85.83	1053.97	0.99	Epiphyte	Shrubland	Endemic
Panisea yunnanensis	Malipo	1800	13.46	1063.54	85.83	1053.97	0.99	Epiphyte	Dense forest	Endemic
Paphiopedilum armeniacum	Lushui	1400	17.65	1195.57	70.63	911.65	0.76	Epiphyte	Shrubland	Non-endemic
Paphiopedilum armeniacum	Gongshan	1500	15.08	1738.42	78.47	860.43	0.50	Epiphyte	Dense forest	Non-endemic
Paphiopedilum armeniacum	Baoshan	2010	13.70	992.38	73.87	1160.15	1.17	Epiphyte	Grassy slope	Non-endemic
Paphiopedilum armeniacum	Baoshan	2415	11.27	992.38	73.87	1160.15	1.17	Epiphyte	Shrubland	Non-endemic
Paphiopedilum bellatulum	Yongde	880	21.85	1266.25	69.00	1283.63	1.01	Terrestrial	Dense forest	Non-endemic
Paphiopedilum bellatulum	Puer	1100	19.66	1429.78	79.51	1123.79	0.79	Terrestrial	Dense forest	Non-endemic
Paphiopedilum concolor	Jinping	1200	18.29	2305.17	83.81	1030.46	0.45	Epiphyte	Dense forest	Non-endemic
Paphiopedilum concolor	Jianshui	1430	17.97	793.10	71.73	1413.81	1.78	Epiphyte	Forest edge	Non-endemic
Paphiopedilum dianthum	Xichou	1000	18.93	1267.54	82.98	985.97	0.78	Epiphyte	Dense forest	Non-endemic
Paphiopedilum dianthum	Mengzi	1800	15.66	855.35	71.82	1434.56	1.68	epiphyte	Grassy slope	Non-endemic
Paphiopedilum hirsutissimum	Funing	700	19.42	1161.58	79.07	1147.17	0.99	Epiphyte	Sparse forest	Non-endemic
Paphiopedilum hirsutissimum	Xichou	870	19.71	1267.54	82.98	985.97	0.78	Epiphyte	Dense forest	Non-endemic
Paphiopedilum malipoense	Malipo	1500	15.26	1063.54	85.83	1053.97	0.99	Terrestrial	Dense forest	Non-endemic
Paphiopedilum malipoense	Malipo	1600	14.66	1063.54	85.83	1053.97	0.99	Terrestrial	Dense forest	Non-endemic
Paphiopedilum micranthum	Xichou	870	19.71	1267.54	82.98	985.97	0.78	Epiphyte	Dense forest	Non-endemic
Paphiopedilum micranthum	Xichou	1420	16.41	1267.54	82.98	985.97	0.78	Epiphyte	Dense forest	Non-endemic
Paphiopedilum micranthum	Malipo	1470	15.44	1063.54	85.83	1053.97	0.99	Epiphyte	Dense forest	Non-endemic
Paphiopedilum micranthum	Xichou	1700	14.73	1267.54	82.98	985.97	0.78	Epiphyte	Dense forest	Non-endemic
Paphiopedilum parishii	Mengla	1050	18.90	1514.70	84.25	1146.68	0.76	Epiphyte	Dense forest	Non-endemic
Paphiopedilum tigrinum	Lushui	1920	14.53	1195.57	70.63	911.65	0.76	Epiphyte	Shrubland	Non-endemic
Paphiopedilum villosum var.annanpense	Xichou	880	19.65	1267.54	82.98	985.97	0.78	Epiphyte	Sparse forest	Non-endemic
Paphiopedilum villosum var.yunnanensis	Malipo	1200	17.06	1063.54	85.83	1053.97	0.99	Epiphyte	Dense forest	Endemic
Paphiopedilum villosum var.yunnanensis	Malipo	1800	13.46	1063.54	85.83	1053.97	0.99	Epiphyte	Dense forest	Endemic
Papilionanthe biswasiana	Jinghong	1800	14.92	1161.08	80.46	1256.19	1.08	Epiphyte	Dense forest	Non-endemic
Papilionanthe biswasiana	Menghai	1900	14.16	1314.38	80.83	1150.91	0.88	Epiphyte	Dense forest	Non-endemic
Papilionanthe teres	Mengla	500	22.20	1514.70	84.25	1146.68	0.76	Epiphyte	Dense forest	Non-endemic
Papilionanthe teres	Jinghong	540	22.48	1161.08	80.46	1256.19	1.08	Epiphyte	Dense forest	Non-endemic
Papilionanthe teres	Puer	570	22.84	1429.78	79.51	1123.79	0.79	Epiphyte	Dense forest	Non-endemic
Papilionanthe teres	Jinghong	670	21.70	1161.08	80.46	1256.19	1.08	Epiphyte	Dense forest	Non-endemic

Papilionanthe teres	Mengla	760	20.64	1514.70	84.25	1146.68	0.76	Epiphyte	Dense forest	Non-endemic
Papilionanthe teres	Jinghong	900	20.32	1161.08	80.46	1256.19	1.08	Epiphyte	Dense forest	Non-endemic
Parapteroceras elobe	Mengla	1100	18.60	1514.70	84.25	1146.68	0.76	Epiphyte	Forest edge	Non-endemic
Parapteroceras elobe	Mengla	1500	16.20	1514.70	84.25	1146.68	0.76	Epiphyte	Forest edge	Non-endemic
Pecteilis henryi	Menghai	1800	14.76	1314.38	80.83	1150.91	0.88	Terrestrial	Meadow	Non-endemic
Pecteilis susannae	Puer	1000	20.26	1429.78	79.51	1123.79	0.79	Terrestrial	Sparse forest	Non-endemic
Pecteilis susannae	Shuangjiang	1500	16.98	1006.64	75.26	1272.13	1.26	Terrestrial	Sparse forest	Non-endemic
Pecteilis susannae	Chuxiong	1800	15.77	874.28	69.99	1192.36	1.36	Terrestrial	Grassy slope	Non-endemic
Pecteilis susannae	Kunming	1950	14.58	1019.14	72.30	1197.62	1.18	Terrestrial	Grassy slope	Non-endemic
Pecteilis susannae	Yangbi	2000	13.99	1044.23	72.19	1161.15	1.11	Terrestrial	Grassy slope	Non-endemic
Pecteilis susannae	Tengchong	2000	13.00	1501.45	78.06	857.93	0.57	Terrestrial	Open site	Non-endemic
Pecteilis susannae	Eryuan	2020	14.41	745.16	68.27	1210.94	1.63	Terrestrial	Grassy slope	Non-endemic
Pecteilis susannae	Yangbi	2100	13.39	1044.23	72.19	1161.15	1.11	Terrestrial	Open site	Non-endemic
Pecteilis susannae	Zhongdian	2400	11.04	641.73	69.05	914.89	1.43	Terrestrial	Grassy slope	Non-endemic
Pecteilis susannae	Zhongdian	2500	10.44	641.73	69.05	914.89	1.43	Terrestrial	Dense forest	Non-endemic
Pelatanthe riarivesii	Mengla	600	21.60	1514.70	84.25	1146.68	0.76	Epiphyte	Dense forest	Non-endemic
Pelatanthe riarivesii	Mengla	710	20.94	1514.70	84.25	1146.68	0.76	Epiphyte	Dense forest	Non-endemic
Pelatantheria bicuspidata	Mengla	700	21.00	1514.70	84.25	1146.68	0.76	Epiphyte	Dense forest	Non-endemic
Pelatantheria bicuspidata	Mengla	800	20.40	1514.70	84.25	1146.68	0.76	Epiphyte	Dense forest	Non-endemic
Peristylus affinis	Pingbian	1300	17.02	1648.57	86.34	990.16	0.60	Terrestrial	Open site	Non-endemic
Peristylus affinis	Fugong	1500	15.06	1441.43	79.99	906.18	0.63	Terrestrial	Grassy slope	Non-endemic
Peristylus affinis	Fugong	2000	12.06	1441.43	79.99	906.18	0.63	Terrestrial	Dense forest	Non-endemic
Peristylus bulleyi	Dali	2400	12.42	1082.70	68.61	1256.27	1.16	Terrestrial	Grassy slope	Non-endemic
Peristylus bulleyi	Jianchuan	2500	10.55	753.13	70.16	1154.48	1.53	Terrestrial	Dense forest	Non-endemic
Peristylus bulleyi	Heqing	2500	11.79	977.00	65.16	1211.84	1.24	Terrestrial	Grassy slope	Non-endemic
Peristylus bulleyi	Dali	2500	11.82	1082.70	68.61	1256.27	1.16	Terrestrial	Grassy slope	Non-endemic
Peristylus bulleyi	Dali	2600	11.22	1082.70	68.61	1256.27	1.16	Terrestrial	Dense forest	Non-endemic
Peristylus bulleyi	Heqing	2700	10.59	977.00	65.16	1211.84	1.24	Terrestrial	Grassy slope	Non-endemic
Peristylus bulleyi	Dali	2700	10.62	1082.70	68.61	1256.27	1.16	Terrestrial	Grassy slope	Non-endemic
Peristylus bulleyi	Dayao	2700	10.73	810.80	65.01	1427.38	1.76	Terrestrial	Shrubland	Non-endemic
Peristylus bulleyi	Lijiang	2800	10.27	982.53	63.24	1077.65	1.10	Terrestrial	Grassy slope	Non-endemic
Peristylus bulleyi	Dongchuan	3200	6.63	1021.73	71.65	1186.61	1.16	Terrestrial	Meadow	Non-endemic
Peristylus bulleyi	Zhongdian	3300	5.64	641.73	69.05	914.89	1.43	Terrestrial	Grassy slope	Non-endemic
Peristylus bulleyi	Zhongdian	3300	5.64	641.73	69.05	914.89	1.43	Terrestrial	Grassy slope	Non-endemic
Peristylus calcaratus	Gongshan	1200	16.88	1738.42	78.47	860.43	0.50	Terrestrial	Dense forest	Non-endemic
Peristylus calcaratus	Gongshan	3337	4.06	1738.42	78.47	860.43	0.50	Terrestrial	Dense forest	Non-endemic

Peristylus coeloceras	Deqin	2050	12.87	639.48	70.85	896.66	1.40	Terrestrial	Forest edge	Non-endemic
Peristylus coeloceras	Fuyuan	2250	11.37	1084.02	74.80	1096.10	1.01	Terrestrial	Grassy slope	Non-endemic
Peristylus coeloceras	Heqing	2320	12.87	977.00	65.16	1211.84	1.24	Terrestrial	Sparse forest	Non-endemic
Peristylus coeloceras	Huize	2340	11.33	799.98	71.46	1179.17	1.47	Terrestrial	Meadow	Non-endemic
Peristylus coeloceras	Lijiang	2600	11.47	982.53	63.24	1077.65	1.10	Terrestrial	Meadow	Non-endemic
Peristylus coeloceras	Lijiang	2700	10.87	982.53	63.24	1077.65	1.10	Terrestrial	Grassy slope	Non-endemic
Peristylus coeloceras	Lijiang	2750	10.57	982.53	63.24	1077.65	1.10	Terrestrial	Grassy slope	Non-endemic
Peristylus coeloceras	Lijiang	2800	10.27	982.53	63.24	1077.65	1.10	Terrestrial	Grassy slope	Non-endemic
Peristylus coeloceras	Lijiang	2900	9.67	982.53	63.24	1077.65	1.10	Terrestrial	Meadow	Non-endemic
Peristylus coeloceras	Deqin	3000	7.17	639.48	70.85	896.66	1.40	Terrestrial	Grassy slope	Non-endemic
Peristylus coeloceras	Eryuan	3000	8.53	745.16	68.27	1210.94	1.63	Terrestrial	Grassy slope	Non-endemic
Peristylus coeloceras	Dongchuan	3000	7.83	1021.73	71.65	1186.61	1.16	Terrestrial	Grassy slope	Non-endemic
Peristylus coeloceras	Zhongdian	3050	7.14	641.73	69.05	914.89	1.43	Terrestrial	Grassy slope	Non-endemic
Peristylus coeloceras	Zhongdian	3100	6.84	641.73	69.05	914.89	1.43	Terrestrial	Meadow	Non-endemic
Peristylus coeloceras	Zhongdian	3100	6.84	641.73	69.05	914.89	1.43	Terrestrial	Meadow	Non-endemic
Peristylus coeloceras	Luquan	3190	6.58	965.09	73.60	1160.89	1.20	Terrestrial	Grassy slope	Non-endemic
Peristylus coeloceras	Zhongdian	3200	6.24	641.73	69.05	914.89	1.43	Terrestrial	Grassy slope	Non-endemic
Peristylus coeloceras	Lijiang	3200	7.87	982.53	63.24	1077.65	1.10	Terrestrial	Meadow	Non-endemic
Peristylus coeloceras	Dongchuan	3200	6.63	1021.73	71.65	1186.61	1.16	Terrestrial	Meadow	Non-endemic
Peristylus coeloceras	Zhongdian	3250	5.94	641.73	69.05	914.89	1.43	Terrestrial	Grassy slope	Non-endemic
Peristylus coeloceras	Lijiang	3300	7.27	982.53	63.24	1077.65	1.10	Terrestrial	Grassy slope	Non-endemic
Peristylus coeloceras	Zhongdian	3300	5.64	641.73	69.05	914.89	1.43	Terrestrial	Meadow	Non-endemic
Peristylus coeloceras	Lijiang	3300	7.27	982.53	63.24	1077.65	1.10	Terrestrial	Meadow	Non-endemic
Peristylus coeloceras	Zhongdian	3300	5.64	641.73	69.05	914.89	1.43	Terrestrial	Meadow	Non-endemic
Peristylus coeloceras	Zhongdian	3450	4.74	641.73	69.05	914.89	1.43	Terrestrial	Meadow	Non-endemic
Peristylus coeloceras	Zhongdian	3600	3.84	641.73	69.05	914.89	1.43	Terrestrial	Grassy slope	Non-endemic
Peristylus constrictus	Jinghong	700	21.52	1161.08	80.46	1256.19	1.08	Terrestrial	Dense forest	Non-endemic
Peristylus constrictus	Zhenkang	1700	14.64	1602.96	81.14	1089.09	0.68	Terrestrial	Shrubland	Non-endemic
Peristylus constrictus	Binchuan	2100	14.10	573.87	62.93	1418.42	2.47	Terrestrial	Grassy slope	Non-endemic
Peristylus constrictus	Fengqing	2200	13.00	1352.80	73.24	1172.52	0.87	Terrestrial	Grassy slope	Non-endemic
Peristylus constrictus	Eryuan	2200	13.33	745.16	68.27	1210.94	1.63	Terrestrial	Shrubland	Non-endemic
Peristylus densus	Xichou	1500	15.93	1267.54	82.98	985.97	0.78	Terrestrial	Dense forest	Non-endemic
Peristylus densus	Menglian	1650	15.68	1357.68	80.47	1185.94	0.87	Terrestrial	Grassy slope	Non-endemic
Peristylus densus	Jingdong	1950	13.88	1128.40	76.80	1142.11	1.01	Terrestrial	Dense forest	Non-endemic
Peristylus densus	Binchuan	2240	13.26	573.87	62.93	1418.42	2.47	Terrestrial	Dense forest	Non-endemic
Peristylus densus	Heqing	2300	12.99	977.00	65.16	1211.84	1.24	Terrestrial	Dense forest	Non-endemic

Peristylus densus	Dali	3050	8.52	1082.70	68.61	1256.27	1.16	Terrestrial	Dense forest	Non-endemic
Peristylus fallax	Dongchuan	3200	6.63	1021.73	71.65	1186.61	1.16	Terrestrial	Meadow	Non-endemic
Peristylus fallax	Luquan	3250	6.22	965.09	73.60	1160.89	1.20	Terrestrial	Meadow	Non-endemic
Peristylus fallax	Dali	3400	6.42	1082.70	68.61	1256.27	1.16	Terrestrial	Meadow	Non-endemic
Peristylus forceps	Wenshan	1050	19.36	988.87	76.70	1272.98	1.29	Terrestrial	Grassy slope	Non-endemic
Peristylus forceps	Mengzi	1300	18.66	855.35	71.82	1434.56	1.68	Terrestrial	Dense forest	Non-endemic
Peristylus forceps	Anning	1890	14.69	896.67	71.23	1167.91	1.30	Terrestrial	Dense forest	Non-endemic
Peristylus forceps	Eryuan	1900	15.13	745.16	68.27	1210.94	1.63	Terrestrial	Grassy slope	Non-endemic
Peristylus forceps	Jianchuan	2100	12.95	753.13	70.16	1154.48	1.53	Terrestrial	Dense forest	Non-endemic
Peristylus forceps	Anning	2200	12.83	896.67	71.23	1167.91	1.30	Terrestrial	Shrubland	Non-endemic
Peristylus forceps	Heqing	2500	11.79	977.00	65.16	1211.84	1.24	Terrestrial	Grassy slope	Non-endemic
Peristylus forceps	Zhongdian	2900	8.04	641.73	69.05	914.89	1.43	Terrestrial	Grassy slope	Non-endemic
Peristylus forceps	Zhongdian	3300	5.64	641.73	69.05	914.89	1.43	Terrestrial	Grassy slope	Non-endemic
Peristylus forceps	Zhongdian	3300	5.64	641.73	69.05	914.89	1.43	Terrestrial	Grassy slope	Non-endemic
Peristylus forceps	Zhongdian	3300	5.64	641.73	69.05	914.89	1.43	Terrestrial	Meadow	Non-endemic
Peristylus forceps	Zhongdian	3420	4.92	641.73	69.05	914.89	1.43	Terrestrial	Meadow	Non-endemic
Peristylus forceps	Lijiang	4000	3.07	982.53	63.24	1077.65	1.10	Terrestrial	Grassy slope	Non-endemic
Peristylus forrestii	Kunming	1950	14.58	1019.14	72.30	1197.62	1.18	Terrestrial	Grassy slope	Non-endemic
Peristylus forrestii	Lijiang	2450	12.37	982.53	63.24	1077.65	1.10	Terrestrial	Dense forest	Non-endemic
Peristylus goodyeroides	Funing	300	21.82	1161.58	79.07	1147.17	0.99	Terrestrial	Grassy slope	Non-endemic
Peristylus goodyeroides	Malipo	1000	18.26	1063.54	85.83	1053.97	0.99	Terrestrial	Shrubland	Non-endemic
Peristylus goodyeroides	Jingdong	1380	17.30	1128.40	76.80	1142.11	1.01	Terrestrial	Grassy slope	Non-endemic
Peristylus goodyeroides	Jiangcheng	1450	16.43	2264.27	84.63	1061.90	0.47	Terrestrial	Grassy slope	Non-endemic
Peristylus goodyeroides	Yangbi	2100	13.39	1044.23	72.19	1161.15	1.11	Terrestrial	Grassy slope	Non-endemic
Peristylus goodyeroides	Zhongdian	2150	12.54	641.73	69.05	914.89	1.43	Terrestrial	Grassy slope	Non-endemic
Peristylus jinchuanicus	Lijiang	2800	10.27	982.53	63.24	1077.65	1.10	Terrestrial	Meadow	Non-endemic
Peristylus jinchuanicus	Dongchuan	3200	6.63	1021.73	71.65	1186.61	1.16	Terrestrial	Meadow	Non-endemic
Peristylus jinchuanicus	Zhongdian	3300	5.64	641.73	69.05	914.89	1.43	Terrestrial	Open site	Non-endemic
Peristylus lacertiferus	Jinghong	1000	19.72	1161.08	80.46	1256.19	1.08	Terrestrial	Dense forest	Non-endemic
Peristylus lacertiferus	Puer	1000	20.26	1429.78	79.51	1123.79	0.79	Terrestrial	Sparse forest	Non-endemic
Peristylus lacertiferus	Jinghong	1200	18.52	1161.08	80.46	1256.19	1.08	Terrestrial	Forest edge	Non-endemic
Peristylus lacertiferus	Jinghong	1270	18.10	1161.08	80.46	1256.19	1.08	Terrestrial	Shrubland	Non-endemic
Peristylus mannii	Kunming	1840	15.24	1019.14	72.30	1197.62	1.18	Terrestrial	Shrubland	Non-endemic
Peristylus mannii	Lincang	1850	15.42	1165.84	72.08	1167.73	1.00	Terrestrial	Grassy slope	Non-endemic
Peristylus mannii	Eshan	1890	13.87	938.39	78.62	1123.48	1.20	Terrestrial	Grassy slope	Non-endemic
Peristylus mannii	Kunming	1900	14.88	1019.14	72.30	1197.62	1.18	Terrestrial	Sparse forest	Non-endemic

Peristylus mannii	Kunming	1930	14.70	1019.14	72.30	1197.62	1.18	Terrestrial	Dense forest	Non-endemic
Peristylus mannii	Jingdong	1950	13.88	1128.40	76.80	1142.11	1.01	Terrestrial	Dense forest	Non-endemic
Peristylus mannii	Kunming	2010	14.22	1019.14	72.30	1197.62	1.18	Terrestrial	Grassy slope	Non-endemic
Peristylus mannii	Songming	2200	12.39	1010.18	74.14	1134.24	1.12	Terrestrial	Shrubland	Non-endemic
Peristylus mannii	Kunming	2400	11.88	1019.14	72.30	1197.62	1.18	Terrestrial	Grassy slope	Non-endemic
Peristylus mannii	Lijiang	2500	12.07	982.53	63.24	1077.65	1.10	Terrestrial	Dense forest	Non-endemic
Peristylus mannii	Yangbi	2500	10.99	1044.23	72.19	1161.15	1.11	Terrestrial	Grassy slope	Non-endemic
Peristylus mannii	Luquan	2500	10.72	965.09	73.60	1160.89	1.20	Terrestrial	Sparse forest	Non-endemic
Peristylus mannii	Jingdong	2600	9.98	1128.40	76.80	1142.11	1.01	Terrestrial	Grassy slope	Non-endemic
Peristylus mannii	Yangbi	2700	9.79	1044.23	72.19	1161.15	1.11	Terrestrial	Dense forest	Non-endemic
Peristylus neotineoides	Gongshan	1800	13.28	1738.42	78.47	860.43	0.50	Terrestrial	Dense forest	Non-endemic
Peristylus neotineoides	Zhongdian	3300	5.64	641.73	69.05	914.89	1.43	Terrestrial	Grassy slope	Non-endemic
Peristylus parishii	Menghai	1450	16.86	1314.38	80.83	1150.91	0.88	Terrestrial	Dense forest	Non-endemic
Peristylus tentaculatus	Cangyuan	1200	18.02	1733.34	81.63	1060.82	0.61	Terrestrial	Forest edge	Non-endemic
Peristylus tentaculatus	Gongshan	1200	16.88	1738.42	78.47	860.43	0.50	Terrestrial	Grassy slope	Non-endemic
Peristylus tentaculatus	Jinping	1340	17.45	2305.17	83.81	1030.46	0.45	Terrestrial	Grassy slope	Non-endemic
Phaius flavus	Gejiu	700	22.11	1100.28	77.18	1242.17	1.13	Terrestrial	Dense forest	Non-endemic
Phaius flavus	Mengla	820	20.28	1514.70	84.25	1146.68	0.76	Terrestrial	Dense forest	Non-endemic
Phaius flavus	Xichou	920	19.41	1267.54	82.98	985.97	0.78	Terrestrial	Dense forest	Non-endemic
Phaius flavus	Funing	1000	17.62	1161.58	79.07	1147.17	0.99	Terrestrial	Dense forest	Non-endemic
Phaius flavus	Gejiu	1000	20.31	1100.28	77.18	1242.17	1.13	Terrestrial	Dense forest	Non-endemic
Phaius flavus	Guangnan	1330	16.27	1044.26	78.94	1090.35	1.04	Terrestrial	Open site	Non-endemic
Phaius flavus	Xichou	1350	16.83	1267.54	82.98	985.97	0.78	Terrestrial	Dense forest	Non-endemic
Phaius flavus	Jinghong	1400	17.32	1161.08	80.46	1256.19	1.08	Terrestrial	Shrubland	Non-endemic
Phaius flavus	Malipo	1600	14.66	1063.54	85.83	1053.97	0.99	Terrestrial	Dense forest	Non-endemic
Phaius flavus	Fugong	2100	11.46	1441.43	79.99	906.18	0.63	Terrestrial	Dense forest	Non-endemic
Phaius flavus	Weixi	2200	12.23	970.70	69.95	1021.03	1.05	Terrestrial	Dense forest	Non-endemic
Phaius flavus	Gongshan	2500	9.08	1738.42	78.47	860.43	0.50	Terrestrial	Dense forest	Non-endemic
Phaius longicruris	Jinghong	1400	17.32	1161.08	80.46	1256.19	1.08	Terrestrial	Shrubland	Endemic
Phaius mishmensis	Hekou	330	21.77	1768.58	84.25	1166.26	0.66	Terrestrial	Dense forest	Non-endemic
Phaius mishmensis	Xichou	1000	18.93	1267.54	82.98	985.97	0.78	Terrestrial	Dense forest	Non-endemic
Phaius mishmensis	Xichou	1300	17.13	1267.54	82.98	985.97	0.78	Terrestrial	Dense forest	Non-endemic
Phaius mishmensis	Jianshui	1430	17.97	793.10	71.73	1413.81	1.78	Terrestrial	Dense forest	Non-endemic
Phaius tankervilleae	Jinping	500	22.49	2305.17	83.81	1030.46	0.45	Terrestrial	Dense forest	Non-endemic
Phaius tankervilleae	Puer	850	21.16	1429.78	79.51	1123.79	0.79	Terrestrial	Forest edge	Non-endemic
Phaius tankervilleae	Hekou	900	18.35	1768.58	84.25	1166.26	0.66	Terrestrial	Forest edge	Non-endemic

Phaius tankervilleae	Gejiu	1000	20.31	1100.28	77.18	1242.17	1.13	Terrestrial	Open site	Non-endemic
Phaius tankervilleae	Jinghong	1100	19.12	1161.08	80.46	1256.19	1.08	Terrestrial	Dense forest	Non-endemic
Phaius tankervilleae	Lushui	1300	18.25	1195.57	70.63	911.65	0.76	Terrestrial	Grassy slope	Non-endemic
Phaius tankervilleae	Malipo	1400	15.86	1063.54	85.83	1053.97	0.99	Terrestrial	Dense forest	Non-endemic
Phaius tankervilleae	Malipo	1400	15.86	1063.54	85.83	1053.97	0.99	Terrestrial	Dense forest	Non-endemic
Phaius tankervilleae	Shuangjiang	1480	17.10	1006.64	75.26	1272.13	1.26	Terrestrial	Grassy slope	Non-endemic
Phaius tankervilleae	Xichou	1500	15.93	1267.54	82.98	985.97	0.78	Terrestrial	Shrubland	Non-endemic
Phaius tankervilleae	Zhenkang	1600	15.24	1602.96	81.14	1089.09	0.68	Terrestrial	Dense forest	Non-endemic
Phaius tankervilleae	Jingdong	1600	15.98	1128.40	76.80	1142.11	1.01	Terrestrial	Dense forest	Non-endemic
Phaius tankervilleae	Fugong	1900	12.66	1441.43	79.99	906.18	0.63	Terrestrial	Shrubland	Non-endemic
Phalaenopsis hainanensis	Xichou	1200	17.73	1267.54	82.98	985.97	0.78	Epiphyte	Dense forest	Non-endemic
Phalaenopsis mannii	Mengla	850	20.10	1514.70	84.25	1146.68	0.76	Epiphyte	Forest edge	Non-endemic
Phalaenopsis mannii	Mengla	1350	17.10	1514.70	84.25	1146.68	0.76	Epiphyte	Forest edge	Non-endemic
Phalaenopsis stobariana	Yingjiang	1350	16.29	1505.33	79.98	1158.19	0.77	Epiphyte	Dense forest	Endemic
Phalaenopsis wilsonii	Malipo	1500	15.26	1063.54	85.83	1053.97	0.99	Epiphyte	Dense forest	Non-endemic
Phalaenopsis wilsonii	Lijiang	2990	9.13	982.53	63.24	1077.65	1.10	Epiphyte	Dense forest	Non-endemic
Pholidota articulata	Jinghong	900	20.32	1161.08	80.46	1256.19	1.08	Epiphyte	Sparse forest	Non-endemic
Pholidota articulata	Zhenkang	1000	18.84	1602.96	81.14	1089.09	0.68	Epiphyte	Dense forest	Non-endemic
Pholidota articulata	Xichou	1080	18.45	1267.54	82.98	985.97	0.78	Epiphyte	Dense forest	Non-endemic
Pholidota articulata	Pingbian	1300	17.02	1648.57	86.34	990.16	0.60	Epiphyte	Dense forest	Non-endemic
Pholidota articulata	Menghai	1300	17.76	1314.38	80.83	1150.91	0.88	Epiphyte	Forest edge	Non-endemic
Pholidota articulata	Jinghong	1350	17.62	1161.08	80.46	1256.19	1.08	Epiphyte	Dense forest	Non-endemic
Pholidota articulata	Menghai	1500	16.56	1314.38	80.83	1150.91	0.88	Epiphyte	Dense forest	Non-endemic
Pholidota articulata	Menghai	1500	16.56	1314.38	80.83	1150.91	0.88	Epiphyte	Dense forest	Non-endemic
Pholidota articulata	Yongde	1520	18.01	1266.25	69.00	1283.63	1.01	Epiphyte	Forest edge	Non-endemic
Pholidota articulata	Menghai	1540	16.32	1314.38	80.83	1150.91	0.88	Epiphyte	Dense forest	Non-endemic
Pholidota articulata	Yingjiang	1600	14.79	1505.33	79.98	1158.19	0.77	Epiphyte	Dense forest	Non-endemic
Pholidota articulata	Gongshan	1600	14.48	1738.42	78.47	860.43	0.50	Epiphyte	Forest edge	Non-endemic
Pholidota articulata	Gongshan	1600	14.48	1738.42	78.47	860.43	0.50	Epiphyte	Grassy slope	Non-endemic
Pholidota articulata	Menghai	1600	15.96	1314.38	80.83	1150.91	0.88	Epiphyte	Shrubland	Non-endemic
Pholidota articulata	Shuangjiang	1600	16.38	1006.64	75.26	1272.13	1.26	Epiphyte	Sparse forest	Non-endemic
Pholidota articulata	Gongshan	1650	14.18	1738.42	78.47	860.43	0.50	Epiphyte	Dense forest	Non-endemic
Pholidota articulata	Fengqing	1680	16.12	1352.80	73.24	1172.52	0.87	Epiphyte	Dense forest	Non-endemic
Pholidota articulata	Fengqing	1700	16.00	1352.80	73.24	1172.52	0.87	Epiphyte	Dense forest	Non-endemic
Pholidota articulata	Gongshan	1700	13.88	1738.42	78.47	860.43	0.50	Epiphyte	Forest edge	Non-endemic
Pholidota articulata	Gongshan	1730	13.70	1738.42	78.47	860.43	0.50	Epiphyte	Dense forest	Non-endemic

Pholidota articulata	Luxi	1750	14.58	1650.33	79.33	1183.39	0.72	Epiphyte	Dense forest	Non-endemic
Pholidota articulata	Luxi	1750	14.58	1650.33	79.33	1183.39	0.72	Epiphyte	Dense forest	Non-endemic
Pholidota articulata	Longling	1800	13.32	2098.66	84.65	973.45	0.46	Epiphyte	Dense forest	Non-endemic
Pholidota articulata	Menghai	1800	14.76	1314.38	80.83	1150.91	0.88	Epiphyte	Dense forest	Non-endemic
Pholidota articulata	Jingdong	1800	14.78	1128.40	76.80	1142.11	1.01	Epiphyte	Dense forest	Non-endemic
Pholidota articulata	Gongshan	1800	13.28	1738.42	78.47	860.43	0.50	Epiphyte	Forest edge	Non-endemic
Pholidota articulata	Weixi	1900	14.03	970.70	69.95	1021.03	1.05	Epiphyte	Forest edge	Non-endemic
Pholidota articulata	Jingdong	1950	13.88	1128.40	76.80	1142.11	1.01	Epiphyte	Dense forest	Non-endemic
Pholidota articulata	Jingdong	1950	13.88	1128.40	76.80	1142.11	1.01	Epiphyte	Dense forest	Non-endemic
Pholidota articulata	Jingdong	1950	13.88	1128.40	76.80	1142.11	1.01	Epiphyte	Grassy slope	Non-endemic
Pholidota articulata	Yongde	1970	15.31	1266.25	69.00	1283.63	1.01	Epiphyte	Dense forest	Non-endemic
Pholidota articulata	Gongshan	2000	12.08	1738.42	78.47	860.43	0.50	Epiphyte	Dense forest	Non-endemic
Pholidota articulata	Fugong	2000	12.06	1441.43	79.99	906.18	0.63	Epiphyte	Dense forest	Non-endemic
Pholidota articulata	Yingjiang	2030	12.21	1505.33	79.98	1158.19	0.77	Epiphyte	Dense forest	Non-endemic
Pholidota articulata	Jingdong	2050	13.28	1128.40	76.80	1142.11	1.01	Epiphyte	Dense forest	Non-endemic
Pholidota articulata	Yongde	2100	14.53	1266.25	69.00	1283.63	1.01	Epiphyte	Dense forest	Non-endemic
Pholidota articulata	Fengqing	2100	13.60	1352.80	73.24	1172.52	0.87	Epiphyte	Dense forest	Non-endemic
Pholidota articulata	Shuangjiang	2100	13.38	1006.64	75.26	1272.13	1.26	Epiphyte	Forest edge	Non-endemic
Pholidota articulata	Jingdong	2200	12.38	1128.40	76.80	1142.11	1.01	Epiphyte	Dense forest	Non-endemic
Pholidota articulata	Jingdong	2200	12.38	1128.40	76.80	1142.11	1.01	Epiphyte	Dense forest	Non-endemic
Pholidota articulata	Lincang	2300	12.72	1165.84	72.08	1167.73	1.00	Epiphyte	Dense forest	Non-endemic
Pholidota articulata	Yangbi	2350	11.89	1044.23	72.19	1161.15	1.11	Epiphyte	Dense forest	Non-endemic
Pholidota articulata	Jingdong	2400	11.18	1128.40	76.80	1142.11	1.01	Epiphyte	Dense forest	Non-endemic
Pholidota articulata	Longling	2400	9.72	2098.66	84.65	973.45	0.46	Epiphyte	Dense forest	Non-endemic
Pholidota articulata	Gongshan	2400	9.68	1738.42	78.47	860.43	0.50	Epiphyte	Dense forest	Non-endemic
Pholidota articulata	Fengqing	2450	11.50	1352.80	73.24	1172.52	0.87	Epiphyte	Dense forest	Non-endemic
Pholidota articulata	Yongde	2500	12.13	1266.25	69.00	1283.63	1.01	Epiphyte	Dense forest	Non-endemic
Pholidota articulata	Zhenkang	2700	8.64	1602.96	81.14	1089.09	0.68	Epiphyte	Dense forest	Non-endemic
Pholidota bracteata	Gengma	1200	18.04	1327.70	77.28	1181.78	0.89	Epiphyte	Dense forest	Non-endemic
Pholidota bracteata	Jinghong	1300	17.92	1161.08	80.46	1256.19	1.08	Epiphyte	Sparse forest	Non-endemic
Pholidota chinensis	Pingbian	800	20.02	1648.57	86.34	990.16	0.60	Epiphyte	Dense forest	Non-endemic
Pholidota chinensis	Pingbian	900	19.42	1648.57	86.34	990.16	0.60	Epiphyte	Dense forest	Non-endemic
Pholidota chinensis	Malipo	1000	18.26	1063.54	85.83	1053.97	0.99	Epiphyte	Dense forest	Non-endemic
Pholidota chinensis	Malipo	1150	17.36	1063.54	85.83	1053.97	0.99	Epiphyte	Dense forest	Non-endemic
Pholidota chinensis	Pingbian	1240	17.38	1648.57	86.34	990.16	0.60	Epiphyte	Dense forest	Non-endemic
Pholidota chinensis	Pingbian	1300	17.02	1648.57	86.34	990.16	0.60	Epiphyte	Dense forest	Non-endemic

Pholidota chinensis	Xichou	1300	17.13	1267.54	82.98	985.97	0.78	Epiphyte	Dense forest	Non-endemic
Pholidota chinensis	Xichou	1350	16.83	1267.54	82.98	985.97	0.78	Epiphyte	Dense forest	Non-endemic
Pholidota chinensis	Gongshan	1500	15.08	1738.42	78.47	860.43	0.50	Epiphyte	Dense forest	Non-endemic
Pholidota chinensis	Malipo	1500	15.26	1063.54	85.83	1053.97	0.99	Epiphyte	Dense forest	Non-endemic
Pholidota chinensis	Pingbian	1600	15.22	1648.57	86.34	990.16	0.60	Epiphyte	Dense forest	Non-endemic
Pholidota chinensis	Malipo	1600	14.66	1063.54	85.83	1053.97	0.99	Epiphyte	Dense forest	Non-endemic
Pholidota chinensis	Lancang	2100	13.10	1596.50	77.88	1183.80	0.74	Epiphyte	Dense forest	Non-endemic
Pholidota imbricata	Mengla	800	20.40	1514.70	84.25	1146.68	0.76	Epiphyte	Dense forest	Non-endemic
Pholidota imbricata	Jinghong	830	20.74	1161.08	80.46	1256.19	1.08	Epiphyte	Dense forest	Non-endemic
Pholidota imbricata	Jinghong	1000	19.72	1161.08	80.46	1256.19	1.08	Epiphyte	Dense forest	Non-endemic
Pholidota imbricata	Menghai	1000	19.56	1314.38	80.83	1150.91	0.88	Epiphyte	Shrubland	Non-endemic
Pholidota imbricata	Jinghong	1100	19.12	1161.08	80.46	1256.19	1.08	Epiphyte	Dense forest	Non-endemic
Pholidota imbricata	Jinghong	1100	19.12	1161.08	80.46	1256.19	1.08	Epiphyte	Dense forest	Non-endemic
Pholidota imbricata	Menghai	1100	18.96	1314.38	80.83	1150.91	0.88	Epiphyte	Dense forest	Non-endemic
Pholidota imbricata	Menghai	1250	18.06	1314.38	80.83	1150.91	0.88	Epiphyte	Sparse forest	Non-endemic
Pholidota imbricata	Menghai	1300	17.76	1314.38	80.83	1150.91	0.88	Epiphyte	Dense forest	Non-endemic
Pholidota imbricata	Menghai	1300	17.76	1314.38	80.83	1150.91	0.88	Epiphyte	Sparse forest	Non-endemic
Pholidota imbricata	Gongshan	1320	16.16	1738.42	78.47	860.43	0.50	Epiphyte	Dense forest	Non-endemic
Pholidota imbricata	Menghai	1340	17.52	1314.38	80.83	1150.91	0.88	Epiphyte	Dense forest	Non-endemic
Pholidota imbricata	Menghai	1340	17.52	1314.38	80.83	1150.91	0.88	Epiphyte	Dense forest	Non-endemic
Pholidota imbricata	Jinghong	1350	17.62	1161.08	80.46	1256.19	1.08	Epiphyte	Dense forest	Non-endemic
Pholidota imbricata	Gongshan	1400	15.68	1738.42	78.47	860.43	0.50	Epiphyte	Dense forest	Non-endemic
Pholidota imbricata	Menghai	1400	17.16	1314.38	80.83	1150.91	0.88	Epiphyte	Dense forest	Non-endemic
Pholidota imbricata	Gongshan	1450	15.38	1738.42	78.47	860.43	0.50	Epiphyte	Dense forest	Non-endemic
Pholidota imbricata	Menghai	1500	16.56	1314.38	80.83	1150.91	0.88	Epiphyte	Shrubland	Non-endemic
Pholidota imbricata	Jinghong	1500	16.72	1161.08	80.46	1256.19	1.08	Epiphyte	Shrubland	Non-endemic
Pholidota imbricata	Menghai	1540	16.32	1314.38	80.83	1150.91	0.88	Epiphyte	Dense forest	Non-endemic
Pholidota imbricata	Menghai	1550	16.26	1314.38	80.83	1150.91	0.88	Epiphyte	Dense forest	Non-endemic
Pholidota imbricata	Menghai	1550	16.26	1314.38	80.83	1150.91	0.88	Epiphyte	Dense forest	Non-endemic
Pholidota imbricata	Menghai	1600	15.96	1314.38	80.83	1150.91	0.88	Epiphyte	Shrubland	Non-endemic
Pholidota imbricata	Gongshan	1620	14.36	1738.42	78.47	860.43	0.50	Epiphyte	Shrubland	Non-endemic
Pholidota imbricata	Zhenkang	1640	15.00	1602.96	81.14	1089.09	0.68	Epiphyte	Dense forest	Non-endemic
Pholidota imbricata	Menghai	1650	15.66	1314.38	80.83	1150.91	0.88	Epiphyte	Dense forest	Non-endemic
Pholidota imbricata	Yangbi	1680	15.91	1044.23	72.19	1161.15	1.11	Epiphyte	Shrubland	Non-endemic
Pholidota imbricata	Gengma	1700	15.47	1327.70	77.28	1181.78	0.89	Epiphyte	Dense forest	Non-endemic
Pholidota imbricata	Fengqing	1700	16.00	1352.80	73.24	1172.52	0.87	Epiphyte	Dense forest	Non-endemic

Pholidota imbricata	Luxi	1750	14.58	1650.33	79.33	1183.39	0.72	Epiphyte	Dense forest	Non-endemic
Pholidota imbricata	Gongshan	1800	13.28	1738.42	78.47	860.43	0.50	Epiphyte	Shrubland	Non-endemic
Pholidota imbricata	Jinghong	1900	14.32	1161.08	80.46	1256.19	1.08	Epiphyte	Dense forest	Non-endemic
Pholidota imbricata	Menghai	1950	13.86	1314.38	80.83	1150.91	0.88	Epiphyte	Dense forest	Non-endemic
Pholidota imbricata	Eryuan	2150	13.63	745.16	68.27	1210.94	1.63	Epiphyte	Forest edge	Non-endemic
Pholidota imbricata	Jingdong	2200	12.38	1128.40	76.80	1142.11	1.01	Epiphyte	Dense forest	Non-endemic
Pholidota imbricata	Menglian	2200	12.38	1357.68	80.47	1185.94	0.87	Epiphyte	Dense forest	Non-endemic
Pholidota imbricata	Lincang	2300	12.72	1165.84	72.08	1167.73	1.00	Epiphyte	Dense forest	Non-endemic
Pholidota imbricata	Lijiang	2430	12.49	982.53	63.24	1077.65	1.10	Epiphyte	Dense forest	Non-endemic
Pholidota imbricata	Longling	2800	7.32	2098.66	84.65	973.45	0.46	Epiphyte	Dense forest	Non-endemic
Pholidota longipes	Xichou	1000	18.93	1267.54	82.98	985.97	0.78	Epiphyte	Dense forest	Endemic
Pholidota longipes	Xichou	1400	16.53	1267.54	82.98	985.97	0.78	Epiphyte	Dense forest	Endemic
Pholidota longipes	Xichou	1650	15.03	1267.54	82.98	985.97	0.78	Epiphyte	Dense forest	Endemic
Pholidota longipes	Malipo	2000	12.26	1063.54	85.83	1053.97	0.99	Epiphyte	Dense forest	Endemic
Pholidota missionariorum	Yanshan	1100	18.86	1003.57	79.42	1172.21	1.17	Epiphyte	Dense forest	Non-endemic
Pholidota missionariorum	Pingbian	1500	15.82	1648.57	86.34	990.16	0.60	Epiphyte	Dense forest	Non-endemic
Pholidota missionariorum	Malipo	1700	14.06	1063.54	85.83	1053.97	0.99	Epiphyte	Shrubland	Non-endemic
Pholidota missionariorum	Hekou	2000	11.75	1768.58	84.25	1166.26	0.66	Epiphyte	Dense forest	Non-endemic
Pholidota protracta	Gongshan	1800	13.28	1738.42	78.47	860.43	0.50	Epiphyte	Dense forest	Non-endemic
Pholidota protracta	Gongshan	2380	9.80	1738.42	78.47	860.43	0.50	Epiphyte	Dense forest	Non-endemic
Pholidota protracta	Lijiang	2500	12.07	982.53	63.24	1077.65	1.10	Epiphyte	Dense forest	Non-endemic
Pholidota protracta	Gongshan	2700	7.88	1738.42	78.47	860.43	0.50	Epiphyte	Dense forest	Non-endemic
Pholidota rupestris	Gongshan	1700	13.88	1738.42	78.47	860.43	0.50	Epiphyte	Dense forest	Non-endemic
Pholidota rupestris	Gongshan	1860	12.92	1738.42	78.47	860.43	0.50	Epiphyte	Shrubland	Non-endemic
Pholidota rupestris	Gongshan	2000	12.08	1738.42	78.47	860.43	0.50	Epiphyte	Dense forest	Non-endemic
Pholidota rupestris	Gongshan	2000	12.08	1738.42	78.47	860.43	0.50	Epiphyte	Dense forest	Non-endemic
Pholidota rupestris	Lijiang	2000	15.07	982.53	63.24	1077.65	1.10	Epiphyte	Forest edge	Non-endemic
Pholidota rupestris	Deqin	2000	13.17	639.48	70.85	896.66	1.40	Epiphyte	Open site	Non-endemic
Pholidota rupestris	Zhongdian	2000	13.44	641.73	69.05	914.89	1.43	Epiphyte	Shrubland	Non-endemic
Pholidota rupestris	Zhongdian	2200	12.24	641.73	69.05	914.89	1.43	Epiphyte	Dense forest	Non-endemic
Pholidota rupestris	Weixi	2300	11.63	970.70	69.95	1021.03	1.05	Epiphyte	Dense forest	Non-endemic
Pholidota rupestris	Gongshan	2300	10.28	1738.42	78.47	860.43	0.50	Epiphyte	Dense forest	Non-endemic
Pholidota rupestris	Gongshan	2300	10.28	1738.42	78.47	860.43	0.50	Epiphyte	Dense forest	Non-endemic
Pholidota rupestris	Lijiang	2340	13.03	982.53	63.24	1077.65	1.10	Epiphyte	Forest edge	Non-endemic
Pholidota rupestris	Gongshan	2500	9.08	1738.42	78.47	860.43	0.50	Epiphyte	Dense forest	Non-endemic
Pholidota rupestris	Gongshan	2600	8.48	1738.42	78.47	860.43	0.50	Epiphyte	Dense forest	Non-endemic

Pholidota wenshanica	Wenshan	1400	17.26	988.87	76.70	1272.98	1.29	Epiphyte	Sparse forest	Endemic
Pholidota wenshanica	Malipo	1500	15.26	1063.54	85.83	1053.97	0.99	Epiphyte	Forest edge	Endemic
Pholidota yunnanensis	Suijiang	850	15.23	917.85	78.74	923.40	1.01	Epiphyte	Open site	Non-endemic
Pholidota yunnanensis	Yanshan	1200	18.26	1003.57	79.42	1172.21	1.17	Epiphyte	Dense forest	Non-endemic
Pholidota yunnanensis	Yanshan	1200	18.26	1003.57	79.42	1172.21	1.17	Epiphyte	Dense forest	Non-endemic
Pholidota yunnanensis	Yanshan	1300	17.66	1003.57	79.42	1172.21	1.17	Epiphyte	Dense forest	Non-endemic
Pholidota yunnanensis	Funing	1300	15.82	1161.58	79.07	1147.17	0.99	Epiphyte	Sparse forest	Non-endemic
Pholidota yunnanensis	Pingbian	1380	16.54	1648.57	86.34	990.16	0.60	Epiphyte	Sparse forest	Non-endemic
Pholidota yunnanensis	Pingbian	1400	16.42	1648.57	86.34	990.16	0.60	Epiphyte	Dense forest	Non-endemic
Pholidota yunnanensis	Xichou	1400	16.53	1267.54	82.98	985.97	0.78	Epiphyte	Shrubland	Non-endemic
Pholidota yunnanensis	Xichou	1450	16.23	1267.54	82.98	985.97	0.78	Epiphyte	Open site	Non-endemic
Pholidota yunnanensis	Xichou	1500	15.93	1267.54	82.98	985.97	0.78	Epiphyte	Dense forest	Non-endemic
Pholidota yunnanensis	Jiangchuan	1640	16.16	872.74	74.72	1181.99	1.35	Epiphyte	Dense forest	Non-endemic
Pholidota yunnanensis	Xichou	1650	15.03	1267.54	82.98	985.97	0.78	Epiphyte	Dense forest	Non-endemic
Pholidota yunnanensis	Mengzi	1700	16.26	855.35	71.82	1434.56	1.68	Epiphyte	Shrubland	Non-endemic
Pholidota yunnanensis	Luxi	1750	14.58	1650.33	79.33	1183.39	0.72	Epiphyte	Dense forest	Non-endemic
Pholidota yunnanensis	Yongde	2000	15.13	1266.25	69.00	1283.63	1.01	Epiphyte	Dense forest	Non-endemic
Phreatia formosana	Jinghong	1800	14.92	1161.08	80.46	1256.19	1.08	Epiphyte	Dense forest	Non-endemic
Platanthera bakeriana	Fugong	1590	14.52	1441.43	79.99	906.18	0.63	Terrestrial	Forest edge	Non-endemic
Platanthera bakeriana	Baoshan	1780	15.08	992.38	73.87	1160.15	1.17	Terrestrial	Forest edge	Non-endemic
Platanthera bakeriana	Heqing	2350	12.69	977.00	65.16	1211.84	1.24	Terrestrial	Meadow	Non-endemic
Platanthera bakeriana	Lijiang	2670	11.05	982.53	63.24	1077.65	1.10	Terrestrial	Meadow	Non-endemic
Platanthera bakeriana	Gongshan	2700	7.88	1738.42	78.47	860.43	0.50	Terrestrial	Grassy slope	Non-endemic
Platanthera bakeriana	Dongchuan	2900	8.43	1021.73	71.65	1186.61	1.16	Terrestrial	Meadow	Non-endemic
Platanthera bakeriana	Lijiang	3000	9.07	982.53	63.24	1077.65	1.10	Terrestrial	Meadow	Non-endemic
Platanthera bakeriana	Fugong	3400	3.66	1441.43	79.99	906.18	0.63	Terrestrial	Dense forest	Non-endemic
Platanthera bakeriana	Deqin	3600	3.57	639.48	70.85	896.66	1.40	Terrestrial	Dense forest	Non-endemic
Platanthera bakeriana	Dali	3600	5.22	1082.70	68.61	1256.27	1.16	Terrestrial	Meadow	Non-endemic
Platanthera bakeriana	Luquan	3660	3.76	965.09	73.60	1160.89	1.20	Terrestrial	Meadow	Non-endemic
Platanthera bakeriana	Zhongdian	3800	2.64	641.73	69.05	914.89	1.43	Terrestrial	Grassy slope	Non-endemic
Platanthera bakeriana	Heqing	3800	3.99	977.00	65.16	1211.84	1.24	Terrestrial	Grassy slope	Non-endemic
Platanthera bakeriana	Zhongdian	3950	1.74	641.73	69.05	914.89	1.43	Terrestrial	Meadow	Non-endemic
Platanthera chiloglossa	Gongshan	1750	13.58	1738.42	78.47	860.43	0.50	Terrestrial	Forest edge	Non-endemic
Platanthera chiloglossa	Dongchuan	2900	8.43	1021.73	71.65	1186.61	1.16	Terrestrial	Meadow	Non-endemic
Platanthera chiloglossa	Deqin	3000	7.17	639.48	70.85	896.66	1.40	Terrestrial	Dense forest	Non-endemic
Platanthera chiloglossa	Luquan	3190	6.58	965.09	73.60	1160.89	1.20	Terrestrial	Grassy slope	Non-endemic

Platanthera chiloglossa	Fugong	3200	4.86	1441.43	79.99	906.18	0.63	Terrestrial	Grassy slope	Non-endemic
Platanthera chiloglossa	Luquan	3900	2.32	965.09	73.60	1160.89	1.20	Terrestrial	Meadow	Non-endemic
Platanthera chlorantha	Weixi	1820	14.51	970.70	69.95	1021.03	1.05	Terrestrial	Dense forest	Non-endemic
Platanthera chlorantha	Deqin	3100	6.57	639.48	70.85	896.66	1.40	Terrestrial	Grassy slope	Non-endemic
Platanthera chlorantha	Zhongdian	3100	6.84	641.73	69.05	914.89	1.43	Terrestrial	Shrubland	Non-endemic
Platanthera chlorantha	Zhongdian	3100	6.84	641.73	69.05	914.89	1.43	Terrestrial	Shrubland	Non-endemic
Platanthera chlorantha	Zhongdian	3300	5.64	641.73	69.05	914.89	1.43	Terrestrial	Dense forest	Non-endemic
Platanthera chlorantha	Zhongdian	3450	4.74	641.73	69.05	914.89	1.43	Terrestrial	Grassy slope	Non-endemic
Platanthera clavigera	Zhongdian	3100	6.84	641.73	69.05	914.89	1.43	Terrestrial	Meadow	Non-endemic
Platanthera exelliana	Deqin	2900	7.77	639.48	70.85	896.66	1.40	Terrestrial	Grassy slope	Non-endemic
Platanthera exelliana	Deqin	3300	5.37	639.48	70.85	896.66	1.40	Terrestrial	Grassy slope	Non-endemic
Platanthera exelliana	Gongshan	3800	1.28	1738.42	78.47	860.43	0.50	Terrestrial	Meadow	Non-endemic
Platanthera finetiana	Lushui	2200	12.85	1195.57	70.63	911.65	0.76	Terrestrial	Forest edge	Non-endemic
Platanthera handel-mazzettii	Gongshan	3111	5.41	1738.42	78.47	860.43	0.50	Terrestrial	Dense forest	Endemic
Platanthera handel-mazzettii	Gongshan	3354	3.96	1738.42	78.47	860.43	0.50	Terrestrial	Dense forest	Endemic
Platanthera herminioides	Gongshan	3020	5.96	1738.42	78.47	860.43	0.50	Terrestrial	Grassy slope	Endemic
Platanthera herminioides	Gongshan	3800	1.28	1738.42	78.47	860.43	0.50	Terrestrial	Meadow	Endemic
Platanthera japonica	Lushui	1840	15.01	1195.57	70.63	911.65	0.76	Terrestrial	Grassy slope	Non-endemic
Platanthera japonica	Dali	2600	11.22	1082.70	68.61	1256.27	1.16	Terrestrial	Grassy slope	Non-endemic
Platanthera japonica	Yongde	2810	10.27	1266.25	69.00	1283.63	1.01	Terrestrial	Forest edge	Non-endemic
Platanthera japonica	Dali	2900	9.42	1082.70	68.61	1256.27	1.16	Terrestrial	Shrubland	Non-endemic
Platanthera latilabris	Tengchong	1630	15.22	1501.45	78.06	857.93	0.57	Terrestrial	Grassy slope	Non-endemic
Platanthera latilabris	Jingdong	1950	13.88	1128.40	76.80	1142.11	1.01	Terrestrial	Grassy slope	Non-endemic
Platanthera latilabris	Heqing	1980	14.91	977.00	65.16	1211.84	1.24	Terrestrial	Dense forest	Non-endemic
Platanthera latilabris	Kunming	2000	14.28	1019.14	72.30	1197.62	1.18	Terrestrial	Dense forest	Non-endemic
Platanthera latilabris	Kunming	2250	12.78	1019.14	72.30	1197.62	1.18	Terrestrial	Grassy slope	Non-endemic
Platanthera latilabris	Longling	2400	9.72	2098.66	84.65	973.45	0.46	Terrestrial	Forest edge	Non-endemic
Platanthera latilabris	Heqing	2500	11.79	977.00	65.16	1211.84	1.24	Terrestrial	Grassy slope	Non-endemic
Platanthera latilabris	Dongchuan	2850	8.73	1021.73	71.65	1186.61	1.16	Terrestrial	Forest edge	Non-endemic
Platanthera latilabris	Dongchuan	2900	8.43	1021.73	71.65	1186.61	1.16	Terrestrial	Grassy slope	Non-endemic
Platanthera leptocaulon	Gongshan	2900	6.68	1738.42	78.47	860.43	0.50	Terrestrial	Forest edge	Non-endemic
Platanthera leptocaulon	Gongshan	3000	6.08	1738.42	78.47	860.43	0.50	Terrestrial	Grassy slope	Non-endemic
Platanthera leptocaulon	Fugong	4000	0.06	1441.43	79.99	906.18	0.63	Terrestrial	Forest edge	Non-endemic
Platanthera likiangensis	Lijiang	2800	10.27	982.53	63.24	1077.65	1.10	Terrestrial	Dense forest	Endemic
Platanthera likiangensis	Weixi	2900	8.03	970.70	69.95	1021.03	1.05	Terrestrial	Dense forest	Endemic
Platanthera minor	Wuding	2400	10.99	988.68	74.74	1188.19	1.20	Terrestrial	Dense forest	Non-endemic

Platanthera minutiflora	Yongde	2700	10.93	1266.25	69.00	1283.63	1.01	Terrestrial	Grassy slope	Non-endemic
Platanthera minutiflora	Lijiang	3200	7.87	982.53	63.24	1077.65	1.10	Terrestrial	Dense forest	Non-endemic
Platanthera minutiflora	Deqin	3540	3.93	639.48	70.85	896.66	1.40	Terrestrial	Forest edge	Non-endemic
Platanthera minutiflora	Zhongdian	4000	1.44	641.73	69.05	914.89	1.43	Terrestrial	Meadow	Non-endemic
Platanthera oreophila	Zhongdian	2800	8.64	641.73	69.05	914.89	1.43	Terrestrial	Dense forest	Non-endemic
Platanthera oreophila	Zhongdian	3500	4.44	641.73	69.05	914.89	1.43	Terrestrial	Dense forest	Non-endemic
Platanthera oreophila	Zhongdian	3600	3.84	641.73	69.05	914.89	1.43	Terrestrial	Grassy slope	Non-endemic
Platanthera oreophila	Zhongdian	3800	2.64	641.73	69.05	914.89	1.43	Terrestrial	Grassy slope	Non-endemic
Platanthera oreophila	Zhongdian	3800	2.64	641.73	69.05	914.89	1.43	Terrestrial	Shrubland	Non-endemic
Platanthera platantheroides	Menglian	2450	10.88	1357.68	80.47	1185.94	0.87	Terrestrial	Shrubland	Non-endemic
Platanthera roseotincta	Gongshan	3400	3.68	1738.42	78.47	860.43	0.50	Terrestrial	Meadow	Non-endemic
Platanthera roseotincta	Deqin	3400	4.77	639.48	70.85	896.66	1.40	Terrestrial	Shrubland	Non-endemic
Platanthera roseotincta	Fugong	3500	3.06	1441.43	79.99	906.18	0.63	Terrestrial	Meadow	Non-endemic
Platanthera roseotincta	Gongshan	3600	2.48	1738.42	78.47	860.43	0.50	Terrestrial	Grassy slope	Non-endemic
Platanthera roseotincta	Gongshan	3600	2.48	1738.42	78.47	860.43	0.50	Terrestrial	Sparse forest	Non-endemic
Platanthera roseotincta	Gongshan	3880	0.80	1738.42	78.47	860.43	0.50	Terrestrial	Meadow	Non-endemic
Platanthera sikkimensis	Gongshan	2300	10.28	1738.42	78.47	860.43	0.50	Terrestrial	Dense forest	Non-endemic
Platanthera sikkimensis	Gongshan	2662	8.11	1738.42	78.47	860.43	0.50	Terrestrial	Dense forest	Non-endemic
Platanthera sinica	Eryuan	2280	12.85	745.16	68.27	1210.94	1.63	Terrestrial	Grassy slope	Endemic
Platanthera sinica	Lijiang	2800	10.27	982.53	63.24	1077.65	1.10	Terrestrial	Grassy slope	Endemic
Platanthera sinica	Zhongdian	2900	8.04	641.73	69.05	914.89	1.43	Terrestrial	Grassy slope	Endemic
Platanthera sinica	Gongshan	3200	4.88	1738.42	78.47	860.43	0.50	Terrestrial	Dense forest	Endemic
Platanthera stenantha	Qiaojia	2300	9.82	878.15	76.01	1034.97	1.18	Terrestrial	Grassy slope	Non-endemic
Platanthera stenantha	Gongshan	3500	3.08	1738.42	78.47	860.43	0.50	Terrestrial	Dense forest	Non-endemic
Platanthera stenophylla	Gongshan	2500	9.08	1738.42	78.47	860.43	0.50	Terrestrial	Grassy slope	Non-endemic
Pleione alba	Jingdong	3000	7.58	1128.40	76.80	1142.11	1.01	Epiphyte	Open site	Endemic
Pleione bulbocodioides	Menglian	980	19.70	1357.68	80.47	1185.94	0.87	Epiphyte	Shrubland	Non-endemic
Pleione bulbocodioides	Yongshan	1800	10.90	665.40	74.22	968.77	1.46	Epiphyte	Shrubland	Non-endemic
Pleione bulbocodioides	Daguan	1850	10.72	987.00	80.05	896.37	0.91	Epiphyte	Dense forest	Non-endemic
Pleione bulbocodioides	Dali	1910	15.36	1082.70	68.61	1256.27	1.16	Epiphyte	Shrubland	Non-endemic
Pleione bulbocodioides	Dali	1940	15.18	1082.70	68.61	1256.27	1.16	Epiphyte	Shrubland	Non-endemic
Pleione bulbocodioides	Zhenxiong	1950	9.74	899.34	83.70	852.28	0.95	Epiphyte	Shrubland	Non-endemic
Pleione bulbocodioides	Qiaojia	2100	11.02	878.15	76.01	1034.97	1.18	Epiphyte	Open site	Non-endemic
Pleione bulbocodioides	Songming	2150	12.69	1010.18	74.14	1134.24	1.12	Epiphyte	Sparse forest	Non-endemic
Pleione bulbocodioides	Yongde	2200	13.93	1266.25	69.00	1283.63	1.01	Epiphyte	Open site	Non-endemic
Pleione bulbocodioides	Lijiang	2200	13.87	982.53	63.24	1077.65	1.10	Epiphyte	Shrubland	Non-endemic

Pleione bulbocodioides	Fuming	2200	12.77	871.62	71.93	1209.56	1.39	Epiphyte	Shrubland	Non-endemic
Pleione bulbocodioides	Songming	2210	12.33	1010.18	74.14	1134.24	1.12	Epiphyte	Sparse forest	Non-endemic
Pleione bulbocodioides	Jianchuan	2230	12.17	753.13	70.16	1154.48	1.53	Epiphyte	Dense forest	Non-endemic
Pleione bulbocodioides	Luquan	2300	11.92	965.09	73.60	1160.89	1.20	Epiphyte	Sparse forest	Non-endemic
Pleione bulbocodioides	Lijiang	2380	12.79	982.53	63.24	1077.65	1.10	Epiphyte	Open site	Non-endemic
Pleione bulbocodioides	Lijiang	2400	12.67	982.53	63.24	1077.65	1.10	Epiphyte	Dense forest	Non-endemic
Pleione bulbocodioides	Wenshan	2400	11.26	988.87	76.70	1272.98	1.29	Epiphyte	Grassy slope	Non-endemic
Pleione bulbocodioides	Jianchuan	2450	10.85	753.13	70.16	1154.48	1.53	Epiphyte	Shrubland	Non-endemic
Pleione bulbocodioides	Lijiang	2500	12.07	982.53	63.24	1077.65	1.10	Epiphyte	Dense forest	Non-endemic
Pleione bulbocodioides	Zhongdian	2500	10.44	641.73	69.05	914.89	1.43	Epiphyte	Forest edge	Non-endemic
Pleione bulbocodioides	Yongde	2550	11.83	1266.25	69.00	1283.63	1.01	Epiphyte	Dense forest	Non-endemic
Pleione bulbocodioides	Weixi	2560	10.07	970.70	69.95	1021.03	1.05	Epiphyte	Dense forest	Non-endemic
Pleione bulbocodioides	Zhongdian	2600	9.84	641.73	69.05	914.89	1.43	Epiphyte	Dense forest	Non-endemic
Pleione bulbocodioides	Dali	2600	11.22	1082.70	68.61	1256.27	1.16	Epiphyte	Open site	Non-endemic
Pleione bulbocodioides	Jingdong	2600	9.98	1128.40	76.80	1142.11	1.01	Epiphyte	Shrubland	Non-endemic
Pleione bulbocodioides	Qiaojia	2750	7.12	878.15	76.01	1034.97	1.18	Epiphyte	Open site	Non-endemic
Pleione bulbocodioides	Lijiang	2750	10.57	982.53	63.24	1077.65	1.10	Epiphyte	Shrubland	Non-endemic
Pleione bulbocodioides	Lijiang	2760	10.51	982.53	63.24	1077.65	1.10	Epiphyte	Dense forest	Non-endemic
Pleione bulbocodioides	Dayao	2800	10.13	810.80	65.01	1427.38	1.76	Epiphyte	Dense forest	Non-endemic
Pleione bulbocodioides	Ninglang	2800	9.26	928.64	69.17	1136.01	1.22	Epiphyte	Shrubland	Non-endemic
Pleione bulbocodioides	Dali	2800	10.02	1082.70	68.61	1256.27	1.16	Epiphyte	Shrubland	Non-endemic
Pleione bulbocodioides	Dayao	2850	9.83	810.80	65.01	1427.38	1.76	Epiphyte	Dense forest	Non-endemic
Pleione bulbocodioides	Lijiang	2850	9.97	982.53	63.24	1077.65	1.10	Epiphyte	Forest edge	Non-endemic
Pleione bulbocodioides	Deqin	2900	7.77	639.48	70.85	896.66	1.40	Epiphyte	Dense forest	Non-endemic
Pleione bulbocodioides	Lijiang	2910	9.61	982.53	63.24	1077.65	1.10	Epiphyte	Shrubland	Non-endemic
Pleione bulbocodioides	Luquan	2950	8.02	965.09	73.60	1160.89	1.20	Epiphyte	Shrubland	Non-endemic
Pleione bulbocodioides	Lijiang	3100	8.47	982.53	63.24	1077.65	1.10	Epiphyte	Grassy slope	Non-endemic
Pleione bulbocodioides	Yangbi	3100	7.39	1044.23	72.19	1161.15	1.11	Epiphyte	Open site	Non-endemic
Pleione bulbocodioides	Luquan	3150	6.82	965.09	73.60	1160.89	1.20	Epiphyte	Dense forest	Non-endemic
Pleione bulbocodioides	Zhongdian	3300	5.64	641.73	69.05	914.89	1.43	Epiphyte	Meadow	Non-endemic
Pleione bulbocodioides	Lijiang	3300	7.27	982.53	63.24	1077.65	1.10	Epiphyte	Shrubland	Non-endemic
Pleione bulbocodioides	Lijiang	3370	6.85	982.53	63.24	1077.65	1.10	Epiphyte	Meadow	Non-endemic
Pleione bulbocodioides	Weixi	3400	5.03	970.70	69.95	1021.03	1.05	Epiphyte	Dense forest	Non-endemic
Pleione chunii	Yongde	2500	12.13	1266.25	69.00	1283.63	1.01	Epiphyte	Forest edge	Endemic
Pleione chunii	Yongde	2630	11.35	1266.25	69.00	1283.63	1.01	Epiphyte	Dense forest	Endemic
Pleione forrestii	Yangbi	1950	14.29	1044.23	72.19	1161.15	1.11	Epiphyte	Dense forest	Endemic

Pleione forrestii	Yunlong	3000	8.05	1195.57	70.63	911.65	0.76	Epiphyte	Dense forest	Endemic
Pleione forrestii	Yangbi	3100	7.39	1044.23	72.19	1161.15	1.11	Epiphyte	Open site	Endemic
Pleione forrestii	Fugong	3200	4.86	1441.43	79.99	906.18	0.63	Epiphyte	Dense forest	Endemic
Pleione forrestii	Fugong	3200	4.86	1441.43	79.99	906.18	0.63	Epiphyte	Dense forest	Endemic
Pleione grandiflora	Jingdong	2370	11.36	1128.40	76.80	1142.11	1.01	Epiphyte	Dense forest	Endemic
Pleione grandiflora	Lincang	2500	11.52	1165.84	72.08	1167.73	1.00	Epiphyte	Dense forest	Endemic
Pleione grandiflora	Jingdong	2500	10.58	1128.40	76.80	1142.11	1.01	Epiphyte	Shrubland	Endemic
Pleione hookeriana	Yongde	2750	10.63	1266.25	69.00	1283.63	1.01	Epiphyte	Dense forest	Non-endemic
Pleione hookeriana	Jinping	2100	12.89	2305.17	83.81	1030.46	0.45	Epiphyte	Forest edge	Non-endemic
Pleione hookeriana	Yongde	2500	12.13	1266.25	69.00	1283.63	1.01	Epiphyte	Dense forest	Non-endemic
Pleione praecox	Pingbian	1820	13.90	1648.57	86.34	990.16	0.60	Epiphyte	Dense forest	Non-endemic
Pleione praecox	Pingbian	1940	13.18	1648.57	86.34	990.16	0.60	Epiphyte	Dense forest	Non-endemic
Pleione praecox	Gengma	2100	15.47	1327.70	77.28	1181.78	0.89	Epiphyte	Dense forest	Non-endemic
Pleione saxicola	Gongshan	2400	9.68	1738.42	78.47	860.43	0.50	Epiphyte	Dense forest	Endemic
Pleione saxicola	Gongshan	2500	9.08	1738.42	78.47	860.43	0.50	Epiphyte	Open site	Endemic
Pleione scopulorum	Gongshan	2900	6.68	1738.42	78.47	860.43	0.50	Epiphyte	Dense forest	Non-endemic
Pleione scopulorum	Gongshan	2900	6.68	1738.42	78.47	860.43	0.50	Epiphyte	Dense forest	Non-endemic
Pleione scopulorum	Gongshan	3000	6.08	1738.42	78.47	860.43	0.50	Epiphyte	Meadow	Non-endemic
Pleione scopulorum	Fugong	3400	3.66	1441.43	79.99	906.18	0.63	Epiphyte	Dense forest	Non-endemic
Pleione scopulorum	Deqin	3400	4.77	639.48	70.85	896.66	1.40	Epiphyte	Shrubland	Non-endemic
Pleione scopulorum	Deqin	3500	4.17	639.48	70.85	896.66	1.40	Epiphyte	Shrubland	Non-endemic
Pleione scopulorum	Gongshan	3600	2.48	1738.42	78.47	860.43	0.50	Epiphyte	Dense forest	Non-endemic
Pleione scopulorum	Gongshan	3600	2.48	1738.42	78.47	860.43	0.50	Epiphyte	Grassy slope	Non-endemic
Pleione scopulorum	Gongshan	3600	2.48	1738.42	78.47	860.43	0.50	Epiphyte	Grassy slope	Non-endemic
Pleione scopulorum	Gongshan	3700	1.88	1738.42	78.47	860.43	0.50	Epiphyte	Shrubland	Non-endemic
Pleione scopulorum	Gongshan	3900	0.68	1738.42	78.47	860.43	0.50	Epiphyte	Shrubland	Non-endemic
Pleione yunnanensis	Guangnan	1550	14.95	1044.26	78.94	1090.35	1.04	Epiphyte	Grassy slope	Non-endemic
Pleione yunnanensis	Weixi	1750	14.93	970.70	69.95	1021.03	1.05	Epiphyte	Dense forest	Non-endemic
Pleione yunnanensis	Yangbi	1820	15.07	1044.23	72.19	1161.15	1.11	Epiphyte	Sparse forest	Non-endemic
Pleione yunnanensis	Kunming	1860	15.12	1019.14	72.30	1197.62	1.18	Epiphyte	Dense forest	Non-endemic
Pleione yunnanensis	Fuyuan	1900	13.47	1084.02	74.80	1096.10	1.01	Epiphyte	Dense forest	Non-endemic
Pleione yunnanensis	Kunming	1940	14.64	1019.14	72.30	1197.62	1.18	Epiphyte	Grassy slope	Non-endemic
Pleione yunnanensis	Gongshan	2000	12.08	1738.42	78.47	860.43	0.50	Epiphyte	Grassy slope	Non-endemic
Pleione yunnanensis	Dali	2040	14.58	1082.70	68.61	1256.27	1.16	Epiphyte	Grassy slope	Non-endemic
Pleione yunnanensis	Kunming	2210	13.02	1019.14	72.30	1197.62	1.18	Epiphyte	Grassy slope	Non-endemic
Pleione yunnanensis	Shuangbai	2240	13.44	942.53	72.21	1300.52	1.38	Epiphyte	Shrubland	Non-endemic

Pleione yunnanensis	Shuangbai	2250	13.38	942.53	72.21	1300.52	1.38	Epiphyte	Grassy slope	Non-endemic
Pleione yunnanensis	Songming	2270	11.97	1010.18	74.14	1134.24	1.12	Epiphyte	Dense forest	Non-endemic
Pleione yunnanensis	Xinping	2300	12.60	957.31	74.08	1291.86	1.35	Epiphyte	Dense forest	Non-endemic
Pleione yunnanensis	Kunming	2310	12.42	1019.14	72.30	1197.62	1.18	Epiphyte	Shrubland	Non-endemic
Pleione yunnanensis	Kunming	2330	12.30	1019.14	72.30	1197.62	1.18	Epiphyte	Dense forest	Non-endemic
Pleione yunnanensis	Xinping	2380	12.12	957.31	74.08	1291.86	1.35	Epiphyte	Open site	Non-endemic
Pleione yunnanensis	Gongshan	2500	9.08	1738.42	78.47	860.43	0.50	Epiphyte	Grassy slope	Non-endemic
Pleione yunnanensis	Fuyuan	2500	9.87	1084.02	74.80	1096.10	1.01	Epiphyte	Shrubland	Non-endemic
Pleione yunnanensis	Eshan	2500	10.21	938.39	78.62	1123.48	1.20	Epiphyte	Shrubland	Non-endemic
Pleione yunnanensis	Weixi	2500	10.43	970.70	69.95	1021.03	1.05	Epiphyte	Shrubland	Non-endemic
Pleione yunnanensis	Dali	2600	11.22	1082.70	68.61	1256.27	1.16	Epiphyte	Shrubland	Non-endemic
Pleione yunnanensis	Deqin	2650	9.27	639.48	70.85	896.66	1.40	Epiphyte	Dense forest	Non-endemic
Pleione yunnanensis	Deqin	2650	9.27	639.48	70.85	896.66	1.40	Epiphyte	Dense forest	Non-endemic
Pleione yunnanensis	Zhongdian	2650	9.54	641.73	69.05	914.89	1.43	Epiphyte	Grassy slope	Non-endemic
Pleione yunnanensis	Yangbi	2700	9.79	1044.23	72.19	1161.15	1.11	Epiphyte	Dense forest	Non-endemic
Pleione yunnanensis	Wenshan	2800	8.86	988.87	76.70	1272.98	1.29	Epiphyte	Shrubland	Non-endemic
Podochilus khasianus	Menghai	1300	17.76	1314.38	80.83	1150.91	0.88	Epiphyte	Dense forest	Non-endemic
Podochilus khasianus	Menghai	1540	16.32	1314.38	80.83	1150.91	0.88	Epiphyte	Shrubland	Non-endemic
Podochilus khasianus	Menghai	1900	14.16	1314.38	80.83	1150.91	0.88	Epiphyte	Dense forest	Non-endemic
Pogonia japonica	Zhenxiong	1945	9.77	899.34	83.70	852.28	0.95	Terrestrial	Dense forest	Non-endemic
Pogonia yunnanensis	Dali	2210	13.56	1082.70	68.61	1256.27	1.16	Terrestrial	Dense forest	Non-endemic
Pogonia yunnanensis	Gongshan	2300	10.28	1738.42	78.47	860.43	0.50	Terrestrial	Grassy slope	Non-endemic
Pogonia yunnanensis	Gongshan	3000	6.08	1738.42	78.47	860.43	0.50	Terrestrial	Meadow	Non-endemic
Polystachya concreta	Jinghong	590	22.18	1161.08	80.46	1256.19	1.08	Epiphyte	Dense forest	Non-endemic
Polystachya concreta	Jinghong	670	21.70	1161.08	80.46	1256.19	1.08	Epiphyte	Dense forest	Non-endemic
Porpax ustulata	Yingjiang	1300	16.59	1505.33	79.98	1158.19	0.77	Epiphyte	Dense forest	Non-endemic
Porpax ustulata	Menghai	1450	16.86	1314.38	80.83	1150.91	0.88	Epiphyte	Dense forest	Non-endemic
Pteroceras leopardium	Jinghong	700	21.52	1161.08	80.46	1256.19	1.08	Epiphyte	Dense forest	Non-endemic
Pteroceras leopardium	Mengla	950	19.50	1514.70	84.25	1146.68	0.76	Epiphyte	Dense forest	Non-endemic
Renanthera imschootiana	Yuanjiang	500	23.18	805.59	69.08	1652.32	2.05	Epiphyte	Dense forest	Endemic
Rhynchostylis retusa	Pingbian	320	22.90	1648.57	86.34	990.16	0.60	Epiphyte	Dense forest	Non-endemic
Rhynchostylis retusa	Jinping	350	23.39	2305.17	83.81	1030.46	0.45	Epiphyte	Dense forest	Non-endemic
Rhynchostylis retusa	Jinping	800	20.69	2305.17	83.81	1030.46	0.45	Epiphyte	Dense forest	Non-endemic
Rhynchostylis retusa	Shizong	800	20.25	1204.89	79.59	1053.84	0.88	Epiphyte	Forest edge	Non-endemic
Rhynchostylis retusa	Shizong	850	19.95	1204.89	79.59	1053.84	0.88	Epiphyte	Dense forest	Non-endemic
Rhynchostylis retusa	Jinping	910	20.03	2305.17	83.81	1030.46	0.45	Epiphyte	Dense forest	Non-endemic

Rhynchostylis retusa	Lincang	1500	17.52	1165.84	72.08	1167.73	1.00	Epiphyte	Dense forest	Non-endemic
Risleya atropurpurea	Gongshan	3385	3.77	1738.42	78.47	860.43	0.50	Saprophyte	Dense forest	Non-endemic
Risleya atropurpurea	Weixi	3500	4.43	970.70	69.95	1021.03	1.05	Saprophyte	Shrubland	Non-endemic
Robiquetia succisa	Jinghong	800	20.92	1161.08	80.46	1256.19	1.08	Epiphyte	Dense forest	Non-endemic
Robiquetia succisa	Jinghong	1100	19.12	1161.08	80.46	1256.19	1.08	Epiphyte	Dense forest	Non-endemic
Satyrium ciliatum	Yanshan	1200	18.26	1003.57	79.42	1172.21	1.17	Terrestrial	Dense forest	Non-endemic
Satyrium ciliatum	Wenshan	1750	15.16	988.87	76.70	1272.98	1.29	Terrestrial	Grassy slope	Non-endemic
Satyrium ciliatum	Shuangbai	1870	15.66	942.53	72.21	1300.52	1.38	Terrestrial	Dense forest	Non-endemic
Satyrium ciliatum	Kunming	1900	14.88	1019.14	72.30	1197.62	1.18	Terrestrial	Dense forest	Non-endemic
Satyrium ciliatum	Wenshan	2000	13.66	988.87	76.70	1272.98	1.29	Terrestrial	Dense forest	Non-endemic
Satyrium ciliatum	Gongshan	2000	12.08	1738.42	78.47	860.43	0.50	Terrestrial	Grassy slope	Non-endemic
Satyrium ciliatum	Kunming	2000	14.28	1019.14	72.30	1197.62	1.18	Terrestrial	Grassy slope	Non-endemic
Satyrium ciliatum	Wenshan	2000	13.66	988.87	76.70	1272.98	1.29	Terrestrial	Sparse forest	Non-endemic
Satyrium ciliatum	Fuming	2100	13.37	871.62	71.93	1209.56	1.39	Terrestrial	Dense forest	Non-endemic
Satyrium ciliatum	Kunming	2100	13.68	1019.14	72.30	1197.62	1.18	Terrestrial	Grassy slope	Non-endemic
Satyrium ciliatum	Jiangchuan	2100	13.40	872.74	74.72	1181.99	1.35	Terrestrial	Open site	Non-endemic
Satyrium ciliatum	Lijiang	2180	13.99	982.53	63.24	1077.65	1.10	Terrestrial	Meadow	Non-endemic
Satyrium ciliatum	Chuxiong	2200	13.37	874.28	69.99	1192.36	1.36	Terrestrial	Dense forest	Non-endemic
Satyrium ciliatum	Dali	2200	13.62	1082.70	68.61	1256.27	1.16	Terrestrial	Dense forest	Non-endemic
Satyrium ciliatum	Wenshan	2200	12.46	988.87	76.70	1272.98	1.29	Terrestrial	Grassy slope	Non-endemic
Satyrium ciliatum	Kunming	2200	13.08	1019.14	72.30	1197.62	1.18	Terrestrial	Grassy slope	Non-endemic
Satyrium ciliatum	Malipo	2200	11.06	1063.54	85.83	1053.97	0.99	Terrestrial	Open site	Non-endemic
Satyrium ciliatum	Songming	2200	12.39	1010.18	74.14	1134.24	1.12	Terrestrial	Shrubland	Non-endemic
Satyrium ciliatum	Heqing	2230	13.41	977.00	65.16	1211.84	1.24	Terrestrial	Dense forest	Non-endemic
Satyrium ciliatum	Yongde	2250	13.63	1266.25	69.00	1283.63	1.01	Terrestrial	Meadow	Non-endemic
Satyrium ciliatum	Dali	2250	13.32	1082.70	68.61	1256.27	1.16	Terrestrial	Open site	Non-endemic
Satyrium ciliatum	Zhaotong	2300	9.52	708.66	74.85	1046.05	1.48	Terrestrial	Dense forest	Non-endemic
Satyrium ciliatum	Gongshan	2300	10.28	1738.42	78.47	860.43	0.50	Terrestrial	Shrubland	Non-endemic
Satyrium ciliatum	Heqing	2310	12.93	977.00	65.16	1211.84	1.24	Terrestrial	Dense forest	Non-endemic
Satyrium ciliatum	Eshan	2350	11.11	938.39	78.62	1123.48	1.20	Terrestrial	Dense forest	Non-endemic
Satyrium ciliatum	Songming	2370	11.37	1010.18	74.14	1134.24	1.12	Terrestrial	Shrubland	Non-endemic
Satyrium ciliatum	Qiaojia	2400	9.22	878.15	76.01	1034.97	1.18	Terrestrial	Forest edge	Non-endemic
Satyrium ciliatum	Yongde	2400	12.73	1266.25	69.00	1283.63	1.01	Terrestrial	Forest edge	Non-endemic
Satyrium ciliatum	Kunming	2400	11.88	1019.14	72.30	1197.62	1.18	Terrestrial	Grassy slope	Non-endemic
Satyrium ciliatum	Jingdong	2400	11.18	1128.40	76.80	1142.11	1.01	Terrestrial	Grassy slope	Non-endemic
Satyrium ciliatum	Eryuan	2400	12.13	745.16	68.27	1210.94	1.63	Terrestrial	Grassy slope	Non-endemic

Satyrium ciliatum	Jingdong	2400	11.18	1128.40	76.80	1142.11	1.01	Terrestrial	Grassy slope	Non-endemic
Satyrium ciliatum	Kunming	2400	11.88	1019.14	72.30	1197.62	1.18	Terrestrial	Grassy slope	Non-endemic
Satyrium ciliatum	Yangbi	2400	11.59	1044.23	72.19	1161.15	1.11	Terrestrial	Grassy slope	Non-endemic
Satyrium ciliatum	Kunming	2500	11.28	1019.14	72.30	1197.62	1.18	Terrestrial	Dense forest	Non-endemic
Satyrium ciliatum	Songming	2500	10.59	1010.18	74.14	1134.24	1.12	Terrestrial	Dense forest	Non-endemic
Satyrium ciliatum	Luquan	2500	10.72	965.09	73.60	1160.89	1.20	Terrestrial	Grassy slope	Non-endemic
Satyrium ciliatum	Jingdong	2540	10.34	1128.40	76.80	1142.11	1.01	Terrestrial	Dense forest	Non-endemic
Satyrium ciliatum	Weixi	2600	9.83	970.70	69.95	1021.03	1.05	Terrestrial	Dense forest	Non-endemic
Satyrium ciliatum	Weixi	2600	9.83	970.70	69.95	1021.03	1.05	Terrestrial	Dense forest	Non-endemic
Satyrium ciliatum	Yongde	2600	11.53	1266.25	69.00	1283.63	1.01	Terrestrial	Forest edge	Non-endemic
Satyrium ciliatum	Gongshan	2600	8.48	1738.42	78.47	860.43	0.50	Terrestrial	Grassy slope	Non-endemic
Satyrium ciliatum	Lijiang	2600	11.47	982.53	63.24	1077.65	1.10	Terrestrial	Grassy slope	Non-endemic
Satyrium ciliatum	Jingdong	2600	9.98	1128.40	76.80	1142.11	1.01	Terrestrial	Shrubland	Non-endemic
Satyrium ciliatum	Lijiang	2620	11.35	982.53	63.24	1077.65	1.10	Terrestrial	Shrubland	Non-endemic
Satyrium ciliatum	Gongshan	2700	7.88	1738.42	78.47	860.43	0.50	Terrestrial	Dense forest	Non-endemic
Satyrium ciliatum	Lijiang	2700	10.87	982.53	63.24	1077.65	1.10	Terrestrial	Dense forest	Non-endemic
Satyrium ciliatum	Yongde	2700	10.93	1266.25	69.00	1283.63	1.01	Terrestrial	Grassy slope	Non-endemic
Satyrium ciliatum	Lincang	2700	10.32	1165.84	72.08	1167.73	1.00	Terrestrial	Grassy slope	Non-endemic
Satyrium ciliatum	Dayao	2700	10.73	810.80	65.01	1427.38	1.76	Terrestrial	Shrubland	Non-endemic
Satyrium ciliatum	Lijiang	2780	10.39	982.53	63.24	1077.65	1.10	Terrestrial	Dense forest	Non-endemic
Satyrium ciliatum	Weixi	2800	8.63	970.70	69.95	1021.03	1.05	Terrestrial	Dense forest	Non-endemic
Satyrium ciliatum	Lijiang	2800	10.27	982.53	63.24	1077.65	1.10	Terrestrial	Dense forest	Non-endemic
Satyrium ciliatum	Lijiang	2800	10.27	982.53	63.24	1077.65	1.10	Terrestrial	Dense forest	Non-endemic
Satyrium ciliatum	Zhongdian	2800	8.64	641.73	69.05	914.89	1.43	Terrestrial	Grassy slope	Non-endemic
Satyrium ciliatum	Heqing	2800	9.99	977.00	65.16	1211.84	1.24	Terrestrial	Grassy slope	Non-endemic
Satyrium ciliatum	Lijiang	2800	10.27	982.53	63.24	1077.65	1.10	Terrestrial	Grassy slope	Non-endemic
Satyrium ciliatum	Weixi	2800	8.63	970.70	69.95	1021.03	1.05	Terrestrial	Grassy slope	Non-endemic
Satyrium ciliatum	Gongshan	2800	7.28	1738.42	78.47	860.43	0.50	Terrestrial	Grassy slope	Non-endemic
Satyrium ciliatum	Lijiang	2800	10.27	982.53	63.24	1077.65	1.10	Terrestrial	Shrubland	Non-endemic
Satyrium ciliatum	Fengqing	2800	9.40	1352.80	73.24	1172.52	0.87	Terrestrial	Shrubland	Non-endemic
Satyrium ciliatum	Lijiang	2830	10.09	982.53	63.24	1077.65	1.10	Terrestrial	Sparse forest	Non-endemic
Satyrium ciliatum	Dongchuan	2880	8.55	1021.73	71.65	1186.61	1.16	Terrestrial	Dense forest	Non-endemic
Satyrium ciliatum	Weixi	2900	8.03	970.70	69.95	1021.03	1.05	Terrestrial	Dense forest	Non-endemic
Satyrium ciliatum	Binchuan	2900	9.30	573.87	62.93	1418.42	2.47	Terrestrial	Dense forest	Non-endemic
Satyrium ciliatum	Qiaojia	2900	6.22	878.15	76.01	1034.97	1.18	Terrestrial	Dense forest	Non-endemic
Satyrium ciliatum	Lincang	2900	9.12	1165.84	72.08	1167.73	1.00	Terrestrial	Grassy slope	Non-endemic

Satyrium ciliatum	Jingdong	2900	8.18	1128.40	76.80	1142.11	1.01	Terrestrial	Grassy slope	Non-endemic
Satyrium ciliatum	Qiaojia	2900	6.22	878.15	76.01	1034.97	1.18	Terrestrial	Meadow	Non-endemic
Satyrium ciliatum	Lijiang	2900	9.67	982.53	63.24	1077.65	1.10	Terrestrial	Sparse forest	Non-endemic
Satyrium ciliatum	Dongchuan	3000	7.83	1021.73	71.65	1186.61	1.16	Terrestrial	Grassy slope	Non-endemic
Satyrium ciliatum	Zhongdian	3000	7.44	641.73	69.05	914.89	1.43	Terrestrial	Grassy slope	Non-endemic
Satyrium ciliatum	Lijiang	3000	9.07	982.53	63.24	1077.65	1.10	Terrestrial	Meadow	Non-endemic
Satyrium ciliatum	Dali	3029	8.65	1082.70	68.61	1256.27	1.16	Terrestrial	Grassy slope	Non-endemic
Satyrium ciliatum	Lijiang	3050	8.77	982.53	63.24	1077.65	1.10	Terrestrial	Dense forest	Non-endemic
Satyrium ciliatum	Lijiang	3080	8.59	982.53	63.24	1077.65	1.10	Terrestrial	Meadow	Non-endemic
Satyrium ciliatum	Dali	3100	8.22	1082.70	68.61	1256.27	1.16	Terrestrial	Grassy slope	Non-endemic
Satyrium ciliatum	Huize	3150	6.47	799.98	71.46	1179.17	1.47	Terrestrial	Grassy slope	Non-endemic
Satyrium ciliatum	Luquan	3190	6.58	965.09	73.60	1160.89	1.20	Terrestrial	Shrubland	Non-endemic
Satyrium ciliatum	Dali	3200	7.62	1082.70	68.61	1256.27	1.16	Terrestrial	Grassy slope	Non-endemic
Satyrium ciliatum	Luquan	3200	6.52	965.09	73.60	1160.89	1.20	Terrestrial	Grassy slope	Non-endemic
Satyrium ciliatum	Huize	3200	6.17	799.98	71.46	1179.17	1.47	Terrestrial	Meadow	Non-endemic
Satyrium ciliatum	Lanping	3200	6.09	989.98	74.21	939.09	0.95	Terrestrial	Open site	Non-endemic
Satyrium ciliatum	Zhongdian	3300	5.64	641.73	69.05	914.89	1.43	Terrestrial	Meadow	Non-endemic
Satyrium ciliatum	Zhongdian	3350	5.34	641.73	69.05	914.89	1.43	Terrestrial	Grassy slope	Non-endemic
Satyrium ciliatum	Lijiang	3360	6.91	982.53	63.24	1077.65	1.10	Terrestrial	Shrubland	Non-endemic
Satyrium ciliatum	Weixi	3400	5.03	970.70	69.95	1021.03	1.05	Terrestrial	Dense forest	Non-endemic
Satyrium ciliatum	Zhongdian	3500	4.44	641.73	69.05	914.89	1.43	Terrestrial	Dense forest	Non-endemic
Satyrium ciliatum	Lijiang	3500	6.07	982.53	63.24	1077.65	1.10	Terrestrial	Grassy slope	Non-endemic
Satyrium ciliatum	Heqing	3500	5.79	977.00	65.16	1211.84	1.24	Terrestrial	Grassy slope	Non-endemic
Satyrium ciliatum	Zhongdian	3600	3.84	641.73	69.05	914.89	1.43	Terrestrial	Dense forest	Non-endemic
Satyrium ciliatum	Deqin	3630	3.39	639.48	70.85	896.66	1.40	Terrestrial	Dense forest	Non-endemic
Satyrium ciliatum	Weixi	3650	3.53	970.70	69.95	1021.03	1.05	Terrestrial	Shrubland	Non-endemic
Satyrium ciliatum	Weixi	3650	3.53	970.70	69.95	1021.03	1.05	Terrestrial	Shrubland	Non-endemic
Satyrium ciliatum	Zhongdian	3800	2.64	641.73	69.05	914.89	1.43	Terrestrial	Meadow	Non-endemic
Satyrium ciliatum	Fugong	4000	0.06	1441.43	79.99	906.18	0.63	Terrestrial	Dense forest	Non-endemic
Satyrium ciliatum	Fugong	4000	0.06	1441.43	79.99	906.18	0.63	Terrestrial	Dense forest	Non-endemic
Satyrium ciliatum	Luquan	4000	1.72	965.09	73.60	1160.89	1.20	Terrestrial	Grassy slope	Non-endemic
Satyrium nepalense	Yuanjiang	1100	19.58	805.59	69.08	1652.32	2.05	Terrestrial	Grassy slope	Non-endemic
Satyrium nepalense	Lancang	1200	18.50	1596.50	77.88	1183.80	0.74	Terrestrial	Grassy slope	Non-endemic
Satyrium nepalense	Yuliang	1800	14.72	905.45	75.03	1218.48	1.35	Terrestrial	Grassy slope	Non-endemic
Satyrium nepalense	Baoshan	1830	14.78	992.38	73.87	1160.15	1.17	Terrestrial	Dense forest	Non-endemic
Satyrium nepalense	Jingdong	1900	14.18	1128.40	76.80	1142.11	1.01	Terrestrial	Grassy slope	Non-endemic

Satyrium nepalense	Jingdong	2000	13.58	1128.40	76.80	1142.11	1.01	Terrestrial	Grassy slope	Non-endemic
Satyrium nepalense	Gongshan	2200	10.88	1738.42	78.47	860.43	0.50	Terrestrial	Dense forest	Non-endemic
Satyrium nepalensis	Yongde	2700	10.93	1266.25	69.00	1283.63	1.01	Terrestrial	Grassy slope	Non-endemic
Satyrium yunnanense	Lincang	2100	13.92	1165.84	72.08	1167.73	1.00	Terrestrial	Grassy slope	Non-endemic
Satyrium yunnanense	Dali	2320	12.90	1082.70	68.61	1256.27	1.16	Terrestrial	Dense forest	Non-endemic
Satyrium yunnanense	Heqing	2350	12.69	977.00	65.16	1211.84	1.24	Terrestrial	Forest edge	Non-endemic
Satyrium yunnanense	Lijiang	2400	12.67	982.53	63.24	1077.65	1.10	Terrestrial	Dense forest	Non-endemic
Satyrium yunnanense	Kunming	2400	11.88	1019.14	72.30	1197.62	1.18	Terrestrial	Shrubland	Non-endemic
Satyrium yunnanense	Zhongdian	2400	11.04	641.73	69.05	914.89	1.43	Terrestrial	Shrubland	Non-endemic
Satyrium yunnanense	Dali	2450	12.12	1082.70	68.61	1256.27	1.16	Terrestrial	Dense forest	Non-endemic
Satyrium yunnanense	Eryuan	2500	11.53	745.16	68.27	1210.94	1.63	Terrestrial	Grassy slope	Non-endemic
Satyrium yunnanense	Dali	2500	11.82	1082.70	68.61	1256.27	1.16	Terrestrial	Shrubland	Non-endemic
Satyrium yunnanense	Wuding	2530	10.21	988.68	74.74	1188.19	1.20	Terrestrial	Dense forest	Non-endemic
Satyrium yunnanense	Lijiang	2540	11.83	982.53	63.24	1077.65	1.10	Terrestrial	Sparse forest	Non-endemic
Satyrium yunnanense	Wuding	2570	9.97	988.68	74.74	1188.19	1.20	Terrestrial	Dense forest	Non-endemic
Satyrium yunnanense	Lijiang	2610	11.41	982.53	63.24	1077.65	1.10	Terrestrial	Grassy slope	Non-endemic
Satyrium yunnanense	Heqing	2700	10.59	977.00	65.16	1211.84	1.24	Terrestrial	Grassy slope	Non-endemic
Satyrium yunnanense	Lijiang	2780	10.39	982.53	63.24	1077.65	1.10	Terrestrial	Forest edge	Non-endemic
Satyrium yunnanense	Zhongdian	2800	8.64	641.73	69.05	914.89	1.43	Terrestrial	Grassy slope	Non-endemic
Satyrium yunnanense	Lijiang	2800	10.27	982.53	63.24	1077.65	1.10	Terrestrial	Grassy slope	Non-endemic
Satyrium yunnanense	Lijiang	3100	8.47	982.53	63.24	1077.65	1.10	Terrestrial	Grassy slope	Non-endemic
Satyrium yunnanense	Zhongdian	3250	5.94	641.73	69.05	914.89	1.43	Terrestrial	Grassy slope	Non-endemic
Schoenorchis gemmata	Malipo	980	18.38	1063.54	85.83	1053.97	0.99	Epiphyte	Dense forest	Non-endemic
Schoenorchis gemmata	Xichou	1200	17.73	1267.54	82.98	985.97	0.78	Epiphyte	Dense forest	Non-endemic
Schoenorchis gemmata	Pingbian	1300	17.02	1648.57	86.34	990.16	0.60	Epiphyte	Dense forest	Non-endemic
Schoenorchis gemmata	Xichou	1300	17.13	1267.54	82.98	985.97	0.78	Epiphyte	Dense forest	Non-endemic
Schoenorchis gemmata	Gongshan	1350	15.98	1738.42	78.47	860.43	0.50	Epiphyte	Dense forest	Non-endemic
Schoenorchis gemmata	Gongshan	1380	15.80	1738.42	78.47	860.43	0.50	Epiphyte	Dense forest	Non-endemic
Schoenorchis gemmata	Gongshan	1380	15.80	1738.42	78.47	860.43	0.50	Epiphyte	Dense forest	Non-endemic
Schoenorchis gemmata	Gongshan	1400	15.68	1738.42	78.47	860.43	0.50	Epiphyte	Dense forest	Non-endemic
Schoenorchis gemmata	Gongshan	1400	15.68	1738.42	78.47	860.43	0.50	Epiphyte	Dense forest	Non-endemic
Schoenorchis gemmata	Gongshan	1450	15.38	1738.42	78.47	860.43	0.50	Epiphyte	Dense forest	Non-endemic
Schoenorchis gemmata	Gongshan	1500	15.08	1738.42	78.47	860.43	0.50	Epiphyte	Dense forest	Non-endemic
Schoenorchis gemmata	Jinping	1500	16.49	2305.17	83.81	1030.46	0.45	Epiphyte	Dense forest	Non-endemic
Schoenorchis gemmata	Gongshan	1540	14.84	1738.42	78.47	860.43	0.50	Epiphyte	Dense forest	Non-endemic
Schoenorchis gemmata	Gongshan	1550	14.78	1738.42	78.47	860.43	0.50	Epiphyte	Dense forest	Non-endemic

Schoenorchis gemmata	Gongshan	1580	14.60	1738.42	78.47	860.43	0.50	Epiphyte	Dense forest	Non-endemic
Schoenorchis gemmata	Gongshan	1580	14.60	1738.42	78.47	860.43	0.50	Epiphyte	Dense forest	Non-endemic
Schoenorchis gemmata	Gongshan	1620	14.36	1738.42	78.47	860.43	0.50	Epiphyte	Dense forest	Non-endemic
Schoenorchis gemmata	Gongshan	1700	13.88	1738.42	78.47	860.43	0.50	Epiphyte	Dense forest	Non-endemic
Schoenorchis gemmata	Gongshan	1700	13.88	1738.42	78.47	860.43	0.50	Epiphyte	Dense forest	Non-endemic
Schoenorchis gemmata	Jinping	1750	14.99	2305.17	83.81	1030.46	0.45	Epiphyte	Dense forest	Non-endemic
Schoenorchis gemmata	Yongde	2000	15.13	1266.25	69.00	1283.63	1.01	Epiphyte	Dense forest	Non-endemic
Schoenorchis gemmata	Gongshan	2000	12.08	1738.42	78.47	860.43	0.50	Epiphyte	Dense forest	Non-endemic
Schoenorchis gemmata	Gongshan	2000	12.08	1738.42	78.47	860.43	0.50	Epiphyte	Dense forest	Non-endemic
Schoenorchis hainanensis	Jinping	1600	15.89	2305.17	83.81	1030.46	0.45	Epiphyte	Dense forest	Non-endemic
Schoenorchis tixieri	Mengla	1000	19.20	1514.70	84.25	1146.68	0.76	Epiphyte	Dense forest	Endemic
Sedirea japonica	Yingjiang	1350	16.29	1505.33	79.98	1158.19	0.77	Epiphyte	dense forest	Non-endemic
Spathoglottis pubescens	Mengla	1040	18.96	1514.70	84.25	1146.68	0.76	Terrestrial	Dense forest	Non-endemic
Spathoglottis pubescens	Lincang	1200	19.32	1165.84	72.08	1167.73	1.00	Terrestrial	Dense forest	Non-endemic
Spathoglottis pubescens	Pingbian	1200	17.62	1648.57	86.34	990.16	0.60	Terrestrial	Dense forest	Non-endemic
Spathoglottis pubescens	Hekou	1250	16.25	1768.58	84.25	1166.26	0.66	Terrestrial	Grassy slope	Non-endemic
Spathoglottis pubescens	Yanshan	1250	17.96	1003.57	79.42	1172.21	1.17	Terrestrial	Grassy slope	Non-endemic
Spathoglottis pubescens	Tengchong	1320	17.08	1501.45	78.06	857.93	0.57	Terrestrial	Dense forest	Non-endemic
Spathoglottis pubescens	Pingbian	1400	16.42	1648.57	86.34	990.16	0.60	Terrestrial	Dense forest	Non-endemic
Spathoglottis pubescens	Shuangjiang	1500	16.98	1006.64	75.26	1272.13	1.26	Terrestrial	Sparse forest	Non-endemic
Spathoglottis pubescens	Tengchong	1550	15.70	1501.45	78.06	857.93	0.57	Terrestrial	Dense forest	Non-endemic
Spathoglottis pubescens	Menglian	1600	15.98	1357.68	80.47	1185.94	0.87	Terrestrial	Grassy slope	Non-endemic
Spathoglottis pubescens	Yuxi	1650	15.87	928.04	74.41	1159.15	1.25	Terrestrial	Open site	Non-endemic
Spathoglottis pubescens	Dayao	1700	16.73	810.80	65.01	1427.38	1.76	Terrestrial	Grassy slope	Non-endemic
Spathoglottis pubescens	Zhenkang	1700	14.64	1602.96	81.14	1089.09	0.68	Terrestrial	Grassy slope	Non-endemic
Spathoglottis pubescens	Maguan	1750	14.51	1330.52	83.25	1086.43	0.82	Terrestrial	Grassy slope	Non-endemic
Spathoglottis pubescens	Yuxi	1800	14.97	928.04	74.41	1159.15	1.25	Terrestrial	Forest edge	Non-endemic
Spathoglottis pubescens	Jingdong	1900	14.18	1128.40	76.80	1142.11	1.01	Terrestrial	Dense forest	Non-endemic
Spathoglottis pubescens	Kunming	1900	14.88	1019.14	72.30	1197.62	1.18	Terrestrial	Grassy slope	Non-endemic
Spathoglottis pubescens	Dali	1920	15.30	1082.70	68.61	1256.27	1.16	Terrestrial	Dense forest	Non-endemic
Spathoglottis pubescens	Hekou	2000	11.75	1768.58	84.25	1166.26	0.66	Terrestrial	Open site	Non-endemic
Spathoglottis pubescens	Dali	2030	14.64	1082.70	68.61	1256.27	1.16	Terrestrial	Dense forest	Non-endemic
Spathoglottis pubescens	Dali	2040	14.58	1082.70	68.61	1256.27	1.16	Terrestrial	Grassy slope	Non-endemic
Spathoglottis pubescens	Kunming	2070	13.86	1019.14	72.30	1197.62	1.18	Terrestrial	Open site	Non-endemic
Spathoglottis pubescens	Kunming	2150	13.38	1019.14	72.30	1197.62	1.18	Terrestrial	Dense forest	Non-endemic
Spathoglottis pubescens	Heqing	2200	13.59	977.00	65.16	1211.84	1.24	Terrestrial	Grassy slope	Non-endemic

Spathoglottis pubescens	Dali	2280	13.14	1082.70	68.61	1256.27	1.16	Terrestrial	Dense forest	Non-endemic
Spathoglottis pubescens	Yuxi	2350	11.67	928.04	74.41	1159.15	1.25	Terrestrial	Grassy slope	Non-endemic
Spiranthes sinensis	Xichou	1250	17.43	1267.54	82.98	985.97	0.78	Terrestrial	Grassy slope	Non-endemic
Spiranthes sinensis	Weixin	1450	11.74	1041.32	84.86	804.04	0.77	Terrestrial	Grassy slope	Non-endemic
Spiranthes sinensis	Xichou	1460	16.17	1267.54	82.98	985.97	0.78	Terrestrial	Grassy slope	Non-endemic
Spiranthes sinensis	Gongshan	1500	15.08	1738.42	78.47	860.43	0.50	Terrestrial	Dense forest	Non-endemic
Spiranthes sinensis	Dali	1500	17.82	1082.70	68.61	1256.27	1.16	Terrestrial	Grassy slope	Non-endemic
Spiranthes sinensis	Heqing	1500	17.79	977.00	65.16	1211.84	1.24	Terrestrial	Open site	Non-endemic
Spiranthes sinensis	Gongshan	1550	14.78	1738.42	78.47	860.43	0.50	Terrestrial	Open site	Non-endemic
Spiranthes sinensis	Luchun	1600	17.10	2013.54	78.64	1151.56	0.57	Terrestrial	Open site	Non-endemic
Spiranthes sinensis	Jingdong	1600	15.98	1128.40	76.80	1142.11	1.01	Terrestrial	Shrubland	Non-endemic
Spiranthes sinensis	Luchun	1620	16.98	2013.54	78.64	1151.56	0.57	Terrestrial	Open site	Non-endemic
Spiranthes sinensis	Eshan	1700	15.01	938.39	78.62	1123.48	1.20	Terrestrial	Forest edge	Non-endemic
Spiranthes sinensis	Eshan	1750	14.71	938.39	78.62	1123.48	1.20	Terrestrial	Dense forest	Non-endemic
Spiranthes sinensis	Jiangchuan	1780	15.32	872.74	74.72	1181.99	1.35	Terrestrial	Grassy slope	Non-endemic
Spiranthes sinensis	Jingdong	1800	14.78	1128.40	76.80	1142.11	1.01	Terrestrial	Grassy slope	Non-endemic
Spiranthes sinensis	Gongshan	1800	13.28	1738.42	78.47	860.43	0.50	Terrestrial	Open site	Non-endemic
Spiranthes sinensis	Anning	1820	15.11	896.67	71.23	1167.91	1.30	Terrestrial	Meadow	Non-endemic
Spiranthes sinensis	Kunming	1900	14.88	1019.14	72.30	1197.62	1.18	Terrestrial	Shrubland	Non-endemic
Spiranthes sinensis	Jinping	1900	14.09	2305.17	83.81	1030.46	0.45	Terrestrial	Sparse forest	Non-endemic
Spiranthes sinensis	Daguan	1950	10.12	987.00	80.05	896.37	0.91	Terrestrial	Dense forest	Non-endemic
Spiranthes sinensis	Eryuan	1950	14.83	745.16	68.27	1210.94	1.63	Terrestrial	Grassy slope	Non-endemic
Spiranthes sinensis	Yongde	2000	15.13	1266.25	69.00	1283.63	1.01	Terrestrial	Dense forest	Non-endemic
Spiranthes sinensis	Kunming	2050	13.98	1019.14	72.30	1197.62	1.18	Terrestrial	Grassy slope	Non-endemic
Spiranthes sinensis	Dali	2080	14.34	1082.70	68.61	1256.27	1.16	Terrestrial	Dense forest	Non-endemic
Spiranthes sinensis	Jingdong	2100	12.98	1128.40	76.80	1142.11	1.01	Terrestrial	Dense forest	Non-endemic
Spiranthes sinensis	Gongshan	2100	11.48	1738.42	78.47	860.43	0.50	Terrestrial	Grassy slope	Non-endemic
Spiranthes sinensis	Tengchong	2100	12.40	1501.45	78.06	857.93	0.57	Terrestrial	Grassy slope	Non-endemic
Spiranthes sinensis	Anning	2150	13.13	896.67	71.23	1167.91	1.30	Terrestrial	Dense forest	Non-endemic
Spiranthes sinensis	Heqing	2160	13.83	977.00	65.16	1211.84	1.24	Terrestrial	Open site	Non-endemic
Spiranthes sinensis	Heqing	2200	13.59	977.00	65.16	1211.84	1.24	Terrestrial	Grassy slope	Non-endemic
Spiranthes sinensis	Jianchuan	2200	12.35	753.13	70.16	1154.48	1.53	Terrestrial	Open site	Non-endemic
Spiranthes sinensis	Heqing	2260	13.23	977.00	65.16	1211.84	1.24	Terrestrial	Forest edge	Non-endemic
Spiranthes sinensis	Weixi	2300	11.63	970.70	69.95	1021.03	1.05	Terrestrial	Meadow	Non-endemic
Spiranthes sinensis	Deqin	2300	11.37	639.48	70.85	896.66	1.40	Terrestrial	Shrubland	Non-endemic
Spiranthes sinensis	Lijiang	2320	13.15	982.53	63.24	1077.65	1.10	Terrestrial	Open site	Non-endemic

Spiranthes sinensis	Kunming	2350	12.18	1019.14	72.30	1197.62	1.18	Terrestrial	Grassy slope	Non-endemic
Spiranthes sinensis	Songming	2380	11.31	1010.18	74.14	1134.24	1.12	Terrestrial	Grassy slope	Non-endemic
Spiranthes sinensis	Kunming	2400	11.88	1019.14	72.30	1197.62	1.18	Terrestrial	Dense forest	Non-endemic
Spiranthes sinensis	Lijiang	2400	12.67	982.53	63.24	1077.65	1.10	Terrestrial	Grassy slope	Non-endemic
Spiranthes sinensis	Yongde	2400	12.73	1266.25	69.00	1283.63	1.01	Terrestrial	Grassy slope	Non-endemic
Spiranthes sinensis	Jingdong	2400	11.18	1128.40	76.80	1142.11	1.01	Terrestrial	Shrubland	Non-endemic
Spiranthes sinensis	Lijiang	2410	12.61	982.53	63.24	1077.65	1.10	Terrestrial	Meadow	Non-endemic
Spiranthes sinensis	Yangbi	2500	10.99	1044.23	72.19	1161.15	1.11	Terrestrial	Dense forest	Non-endemic
Spiranthes sinensis	Yangbi	2500	10.99	1044.23	72.19	1161.15	1.11	Terrestrial	Dense forest	Non-endemic
Spiranthes sinensis	Lijiang	2500	12.07	982.53	63.24	1077.65	1.10	Terrestrial	Open site	Non-endemic
Spiranthes sinensis	Yongde	2500	12.13	1266.25	69.00	1283.63	1.01	Terrestrial	Open site	Non-endemic
Spiranthes sinensis	Wuding	2550	10.09	988.68	74.74	1188.19	1.20	Terrestrial	Grassy slope	Non-endemic
Spiranthes sinensis	Wuding	2550	10.09	988.68	74.74	1188.19	1.20	Terrestrial	Shrubland	Non-endemic
Spiranthes sinensis	Wuding	2560	10.03	988.68	74.74	1188.19	1.20	Terrestrial	Open site	Non-endemic
Spiranthes sinensis	Kunming	2600	10.68	1019.14	72.30	1197.62	1.18	Terrestrial	Grassy slope	Non-endemic
Spiranthes sinensis	Heqing	2600	11.19	977.00	65.16	1211.84	1.24	Terrestrial	Grassy slope	Non-endemic
Spiranthes sinensis	Lijiang	2700	10.87	982.53	63.24	1077.65	1.10	Terrestrial	Grassy slope	Non-endemic
Spiranthes sinensis	Lijiang	2700	10.87	982.53	63.24	1077.65	1.10	Terrestrial	Grassy slope	Non-endemic
Spiranthes sinensis	Lijiang	2720	10.75	982.53	63.24	1077.65	1.10	Terrestrial	Shrubland	Non-endemic
Spiranthes sinensis	Zhongdian	2800	8.64	641.73	69.05	914.89	1.43	Terrestrial	Grassy slope	Non-endemic
Spiranthes sinensis	Lijiang	2850	9.97	982.53	63.24	1077.65	1.10	Terrestrial	Dense forest	Non-endemic
Spiranthes sinensis	Dongchuan	2890	8.49	1021.73	71.65	1186.61	1.16	Terrestrial	Meadow	Non-endemic
Spiranthes sinensis	Dongchuan	2900	8.43	1021.73	71.65	1186.61	1.16	Terrestrial	Grassy slope	Non-endemic
Spiranthes sinensis	Qiaojia	2900	6.22	878.15	76.01	1034.97	1.18	Terrestrial	Meadow	Non-endemic
Spiranthes sinensis	Lijiang	3000	9.07	982.53	63.24	1077.65	1.10	Terrestrial	Forest edge	Non-endemic
Spiranthes sinensis	Dongchuan	3000	7.83	1021.73	71.65	1186.61	1.16	Terrestrial	Grassy slope	Non-endemic
Spiranthes sinensis	Gongshan	3000	6.08	1738.42	78.47	860.43	0.50	Terrestrial	Meadow	Non-endemic
Spiranthes sinensis	Dali	3100	8.22	1082.70	68.61	1256.27	1.16	Terrestrial	Grassy slope	Non-endemic
Spiranthes sinensis	Dali	3100	8.22	1082.70	68.61	1256.27	1.16	Terrestrial	Grassy slope	Non-endemic
Spiranthes sinensis	Lijiang	3100	8.47	982.53	63.24	1077.65	1.10	Terrestrial	Meadow	Non-endemic
Spiranthes sinensis	Lijiang	3145	8.20	982.53	63.24	1077.65	1.10	Terrestrial	Forest edge	Non-endemic
Spiranthes sinensis	Zhongdian	3200	6.24	641.73	69.05	914.89	1.43	Terrestrial	Grassy slope	Non-endemic
Spiranthes sinensis	Zhongdian	3200	6.24	641.73	69.05	914.89	1.43	Terrestrial	Meadow	Non-endemic
Spiranthes sinensis	Zhongdian	3200	6.24	641.73	69.05	914.89	1.43	Terrestrial	Meadow	Non-endemic
Spiranthes sinensis	Zhongdian	3300	5.64	641.73	69.05	914.89	1.43	Terrestrial	Grassy slope	Non-endemic
Spiranthes sinensis	Huize	3400	4.97	799.98	71.46	1179.17	1.47	Terrestrial	Meadow	Non-endemic

Spiranthes sinensis	Zhongdian	3600	3.84	641.73	69.05	914.89	1.43	Terrestrial	Shrubland	Non-endemic
Spiranthes sinensis var.amoena	Dali	1800	16.02	1082.70	68.61	1256.27	1.16	Terrestrial	Grassy slope	Non-endemic
Spiranthes sinensis var.amoena	Dali	2100	14.22	1082.70	68.61	1256.27	1.16	Terrestrial	Grassy slope	Non-endemic
Spiranthes sinensis var.amoena	Dali	2100	14.22	1082.70	68.61	1256.27	1.16	Terrestrial	Grassy slope	Non-endemic
Staurochilus dawsoniana	Mengla	600	21.60	1514.70	84.25	1146.68	0.76	Epiphyte	Forest edge	Non-endemic
Staurochilus dawsoniana	Mengla	700	21.00	1514.70	84.25	1146.68	0.76	Epiphyte	Dense forest	Non-endemic
Staurochilus loratus	Mengla	700	21.00	1514.70	84.25	1146.68	0.76	Epiphyte	Forest edge	Non-endemic
Staurochilus loratus	Mengla	1000	19.20	1514.70	84.25	1146.68	0.76	Epiphyte	Forest edge	Non-endemic
Staurochilus loratus	Menghai	1420	17.04	1314.38	80.83	1150.91	0.88	Epiphyte	Dense forest	Non-endemic
Stereosandra javanica	Menghai	1200	18.36	1314.38	80.83	1150.91	0.88	Saprophyte	Dense forest	Non-endemic
Sunipia andersonii	Mengla	1200	18.00	1514.70	84.25	1146.68	0.76	Epiphyte	Dense forest	Non-endemic
Sunipia andersonii	Menghai	1500	16.56	1314.38	80.83	1150.91	0.88	Epiphyte	Dense forest	Non-endemic
Sunipia andersonii	Fugong	1600	14.46	1441.43	79.99	906.18	0.63	Epiphyte	Dense forest	Non-endemic
Sunipia bicolor	Gongshan	1800	13.28	1738.42	78.47	860.43	0.50	Epiphyte	Dense forest	Non-endemic
Sunipia bicolor	Gongshan	1900	12.68	1738.42	78.47	860.43	0.50	Epiphyte	Dense forest	Non-endemic
Sunipia bicolor	Pingbian	1900	13.42	1648.57	86.34	990.16	0.60	Epiphyte	Dense forest	Non-endemic
Sunipia bicolor	Gongshan	2080	11.60	1738.42	78.47	860.43	0.50	Epiphyte	Dense forest	Non-endemic
Sunipia bicolor	Gongshan	2100	11.48	1738.42	78.47	860.43	0.50	Epiphyte	Dense forest	Non-endemic
Sunipia bicolor	Luchun	2100	14.10	2013.54	78.64	1151.56	0.57	Epiphyte	Forest edge	Non-endemic
Sunipia bicolor	Jingdong	2200	12.38	1128.40	76.80	1142.11	1.01	Epiphyte	Dense forest	Non-endemic
Sunipia bicolor	Zhenkang	2300	11.04	1602.96	81.14	1089.09	0.68	Epiphyte	Dense forest	Non-endemic
Sunipia bicolor	Jingdong	2400	11.18	1128.40	76.80	1142.11	1.01	Epiphyte	Dense forest	Non-endemic
Sunipia bicolor	Jingdong	2400	11.18	1128.40	76.80	1142.11	1.01	Epiphyte	Dense forest	Non-endemic
Sunipia bicolor	Yongde	2400	12.73	1266.25	69.00	1283.63	1.01	Epiphyte	Dense forest	Non-endemic
Sunipia bicolor	Jingdong	2400	11.18	1128.40	76.80	1142.11	1.01	Epiphyte	Shrubland	Non-endemic
Sunipia bicolor	Jingdong	2400	11.18	1128.40	76.80	1142.11	1.01	Epiphyte	Shrubland	Non-endemic
Sunipia bicolor	Jingdong	2500	10.58	1128.40	76.80	1142.11	1.01	Epiphyte	Dense forest	Non-endemic
Sunipia bicolor	Jingdong	2800	8.78	1128.40	76.80	1142.11	1.01	Epiphyte	Grassy slope	Non-endemic
Sunipia candida	Luchun	1700	16.50	2013.54	78.64	1151.56	0.57	Epiphyte	Dense forest	Non-endemic
Sunipia candida	Gongshan	1900	12.68	1738.42	78.47	860.43	0.50	Epiphyte	Dense forest	Non-endemic
Sunipia candida	Yongde	2000	15.13	1266.25	69.00	1283.63	1.01	Epiphyte	Dense forest	Non-endemic
Sunipia candida	Gongshan	2000	12.08	1738.42	78.47	860.43	0.50	Epiphyte	Dense forest	Non-endemic
Sunipia candida	Jingdong	2000	13.58	1128.40	76.80	1142.11	1.01	Epiphyte	Dense forest	Non-endemic
Sunipia candida	Gongshan	2030	11.90	1738.42	78.47	860.43	0.50	Epiphyte	Dense forest	Non-endemic
Sunipia candida	Gongshan	2100	11.48	1738.42	78.47	860.43	0.50	Epiphyte	Dense forest	Non-endemic
Sunipia candida	Gongshan	2100	11.48	1738.42	78.47	860.43	0.50	Epiphyte	Dense forest	Non-endemic

Sunipia candida	Zhongdian	2100	12.84	641.73	69.05	914.89	1.43	Epiphyte	Dense forest	Non-endemic
Sunipia candida	Longling	2400	9.72	2098.66	84.65	973.45	0.46	Epiphyte	Dense forest	Non-endemic
Sunipia candida	Gengma	2500	13.07	1327.70	77.28	1181.78	0.89	Epiphyte	Dense forest	Non-endemic
Sunipia candida	Gongshan	2900	6.68	1738.42	78.47	860.43	0.50	Epiphyte	Dense forest	Non-endemic
Sunipia candida	Jingdong	2900	8.18	1128.40	76.80	1142.11	1.01	Epiphyte	Dense forest	Non-endemic
Sunipia intermedia	Jingdong	2103	12.97	1128.40	76.80	1142.11	1.01	Epiphyte	Dense forest	Non-endemic
Sunipia rimannii	Hekou	1600	14.15	1768.58	84.25	1166.26	0.66	Epiphyte	Forest edge	Non-endemic
Sunipia scariosa	Mengla	620	21.48	1514.70	84.25	1146.68	0.76	Epiphyte	Dense forest	Non-endemic
Sunipia scariosa	Mengla	1050	18.90	1514.70	84.25	1146.68	0.76	Epiphyte	Dense forest	Non-endemic
Sunipia scariosa	Menghai	1600	15.96	1314.38	80.83	1150.91	0.88	Epiphyte	Dense forest	Non-endemic
Sunipia soidaoensis	Jingdong	1950	13.88	1128.40	76.80	1142.11	1.01	Epiphyte	Dense forest	Non-endemic
Sunipia thailandica	Menghai	1400	17.16	1314.38	80.83	1150.91	0.88	Epiphyte	Dense forest	Non-endemic
Taeniophyllum glandulosum	Jinghong	800	20.92	1161.08	80.46	1256.19	1.08	Epiphyte	Forest edge	Non-endemic
Taeniophyllum obtusum	Gongshan	1770	13.46	1738.42	78.47	860.43	0.50	Epiphyte	Dense forest	Non-endemic
Tainia angustifolia	Jinghong	1050	19.42	1161.08	80.46	1256.19	1.08	Terrestrial	Dense forest	Non-endemic
Tainia dunnii	Mengla	700	21.00	1514.70	84.25	1146.68	0.76	Epiphyte	Dense forest	Non-endemic
Tainia dunnii	Menghai	1150	18.66	1314.38	80.83	1150.91	0.88	Epiphyte	Dense forest	Non-endemic
Tainia latifolia	Mengla	820	20.28	1514.70	84.25	1146.68	0.76	Terrestrial	Dense forest	Non-endemic
Tainia latifolia	Jinghong	1050	19.42	1161.08	80.46	1256.19	1.08	Terrestrial	Dense forest	Non-endemic
Tainia latifolia	Menghai	1300	17.76	1314.38	80.83	1150.91	0.88	Terrestrial	Sparse forest	Non-endemic
Tainia latifolia	Lushui	1400	17.65	1195.57	70.63	911.65	0.76	Terrestrial	Dense forest	Non-endemic
Tainia latifolia	Yongde	1700	16.93	1266.25	69.00	1283.63	1.01	Terrestrial	Dense forest	Non-endemic
Tainia latifolia	Yongde	2900	9.73	1266.25	69.00	1283.63	1.01	Terrestrial	Grassy slope	Non-endemic
Tainia latifolia	Yongde	2950	9.43	1266.25	69.00	1283.63	1.01	Terrestrial	Dense forest	Non-endemic
Tainia minor	Mengla	1100	18.60	1514.70	84.25	1146.68	0.76	Terrestrial	Dense forest	Non-endemic
Tainia minor	Gongshan	1480	15.20	1738.42	78.47	860.43	0.50	Terrestrial	Dense forest	Non-endemic
Tainia minor	Gongshan	1700	13.88	1738.42	78.47	860.43	0.50	Terrestrial	Dense forest	Non-endemic
Tainia minor	Wenshan	1900	14.26	988.87	76.70	1272.98	1.29	Terrestrial	Dense forest	Non-endemic
Tainia minor	Tengchong	1900	13.60	1501.45	78.06	857.93	0.57	Terrestrial	Dense forest	Non-endemic
Tainia minor	Jinping	1920	13.97	2305.17	83.81	1030.46	0.45	Terrestrial	Forest edge	Non-endemic
Tainia minor	Fugong	2400	9.66	1441.43	79.99	906.18	0.63	Terrestrial	Dense forest	Non-endemic
Tainia ovifolia	Mengla	1150	18.30	1514.70	84.25	1146.68	0.76	Terrestrial	Dense forest	Endemic
Tainia viridifusca	Yingjiang	1350	16.29	1505.33	79.98	1158.19	0.77	Terrestrial	Dense forest	Non-endemic
Thelasis pygmaea	Mengla	720	20.88	1514.70	84.25	1146.68	0.76	Epiphyte	Dense forest	Non-endemic
Thelasis pygmaea	Mengla	850	20.10	1514.70	84.25	1146.68	0.76	Epiphyte	Dense forest	Non-endemic
Thelasis pygmaea	Menglian	1000	19.58	1357.68	80.47	1185.94	0.87	Epiphyte	Dense forest	Non-endemic

Thelasis pygmaea	Jinghong	1100	19.12	1161.08	80.46	1256.19	1.08	Epiphyte	Shrubland	Non-endemic
Thelasis pygmaea	Menghai	2000	13.56	1314.38	80.83	1150.91	0.88	Epiphyte	Sparse forest	Non-endemic
Thrixspermum centipeda	Hekou	106	23.09	1768.58	84.25	1166.26	0.66	Epiphyte	Sparse forest	Non-endemic
Thrixspermum centipeda	Hekou	260	22.19	1768.58	84.25	1166.26	0.66	Epiphyte	Dense forest	Non-endemic
Thrixspermum centipeda	Hekou	330	21.77	1768.58	84.25	1166.26	0.66	Epiphyte	Dense forest	Non-endemic
Thrixspermum centipeda	Hekou	410	21.29	1768.58	84.25	1166.26	0.66	Epiphyte	Dense forest	Non-endemic
Thrixspermum centipeda	Mengla	500	22.20	1514.70	84.25	1146.68	0.76	Epiphyte	Dense forest	Non-endemic
Thrixspermum centipeda	Mengla	560	21.84	1514.70	84.25	1146.68	0.76	Epiphyte	Dense forest	Non-endemic
Thrixspermum centipeda	Mengla	600	21.60	1514.70	84.25	1146.68	0.76	Epiphyte	Sparse forest	Non-endemic
Thrixspermum centipeda	Jinghong	620	22.00	1161.08	80.46	1256.19	1.08	Epiphyte	Dense forest	Non-endemic
Thrixspermum centipeda	Mengla	700	21.00	1514.70	84.25	1146.68	0.76	Epiphyte	Sparse forest	Non-endemic
Thrixspermum centipeda	Funing	750	19.12	1161.58	79.07	1147.17	0.99	Epiphyte	Sparse forest	Non-endemic
Thrixspermum centipeda	Funing	750	19.12	1161.58	79.07	1147.17	0.99	Epiphyte	Sparse forest	Non-endemic
Thrixspermum centipeda	Jinghong	950	20.02	1161.08	80.46	1256.19	1.08	Epiphyte	Dense forest	Non-endemic
Thrixspermum centipeda	Mengla	1000	19.20	1514.70	84.25	1146.68	0.76	Epiphyte	Dense forest	Non-endemic
Thrixspermum centipeda	Menghai	1100	18.96	1314.38	80.83	1150.91	0.88	Epiphyte	Dense forest	Non-endemic
Thrixspermum centipeda	Menghai	1200	18.36	1314.38	80.83	1150.91	0.88	Epiphyte	Dense forest	Non-endemic
Thrixspermum centipeda	Jinghong	1300	17.92	1161.08	80.46	1256.19	1.08	Epiphyte	Dense forest	Non-endemic
Thrixspermum centipeda	Xichou	1300	17.13	1267.54	82.98	985.97	0.78	Epiphyte	Dense forest	Non-endemic
Thrixspermum japonicum	Mengla	900	19.80	1514.70	84.25	1146.68	0.76	Epiphyte	Dense forest	Non-endemic
Thrixspermum saruwatarii	Gongshan	1700	13.88	1738.42	78.47	860.43	0.50	Epiphyte	Dense forest	Non-endemic
Thrixspermum trichoglottis	Mengla	700	21.00	1514.70	84.25	1146.68	0.76	Epiphyte	Dense forest	Non-endemic
Thrixspermum trichoglottis	Mengla	800	20.40	1514.70	84.25	1146.68	0.76	Epiphyte	Dense forest	Non-endemic
Thrixspermum tsii	Xichou	1300	17.13	1267.54	82.98	985.97	0.78	Epiphyte	Dense forest	Endemic
Thunia alba	Xichou	1280	17.25	1267.54	82.98	985.97	0.78	Epiphyte	Shrubland	Non-endemic
Thunia alba	Xinping	1500	17.40	957.31	74.08	1291.86	1.35	Epiphyte	Dense forest	Non-endemic
Thunia alba	Jingdong	1700	15.38	1128.40	76.80	1142.11	1.01	Epiphyte	Shrubland	Non-endemic
Thunia alba	Jingdong	1900	14.18	1128.40	76.80	1142.11	1.01	Epiphyte	Dense forest	Non-endemic
Thunia alba	Jingdong	2000	13.58	1128.40	76.80	1142.11	1.01	Epiphyte	Dense forest	Non-endemic
Thunia alba	Fengqing	2280	12.52	1352.80	73.24	1172.52	0.87	Epiphyte	Shrubland	Non-endemic
Tipularia szechuanica	Gongshan	2534	8.88	1738.42	78.47	860.43	0.50	Terrestrial	Dense forest	Non-endemic
Tipularia szechuanica	Zhongdian	3850	2.34	641.73	69.05	914.89	1.43	Terrestrial	Dense forest	Non-endemic
Tipularia szechuanica	Zhongdian	3900	2.04	641.73	69.05	914.89	1.43	Terrestrial	Dense forest	Non-endemic
Tipularia szechuanica	Zhongdian	4000	1.44	641.73	69.05	914.89	1.43	Terrestrial	Dense forest	Non-endemic
Trichoglottis triflora	Menghai	1180	18.48	1314.38	80.83	1150.91	0.88	Epiphyte	Dense forest	Non-endemic
Tropidia angulosa	Luquan	800	20.92	965.09	73.60	1160.89	1.20	Terrestrial	Dense forest	Non-endemic

Tropidia angulosa	Mengla	850	20.10	1514.70	84.25	1146.68	0.76	Terrestrial	Dense forest	Non-endemic
Tropidia angulosa	Jinghong	1200	18.52	1161.08	80.46	1256.19	1.08	Terrestrial	Dense forest	Non-endemic
Tropidia angulosa	Pingbian	1900	13.42	1648.57	86.34	990.16	0.60	Terrestrial	Dense forest	Non-endemic
Tropidia angulosa	Yingjiang	2100	11.79	1505.33	79.98	1158.19	0.77	Terrestrial	Dense forest	Non-endemic
Tropidia curculigoides	Gengma	510	10.67	1327.70	77.28	1181.78	0.89	Terrestrial	Dense forest	Non-endemic
Tropidia curculigoides	Mengla	600	21.60	1514.70	84.25	1146.68	0.76	Terrestrial	Dense forest	Non-endemic
Tropidia curculigoides	Jinghong	800	20.92	1161.08	80.46	1256.19	1.08	Terrestrial	Dense forest	Non-endemic
Tropidia curculigoides	Jinghong	800	20.92	1161.08	80.46	1256.19	1.08	Terrestrial	Shrubland	Non-endemic
Tropidia curculigoides	Cangyuan	820	20.30	1733.34	81.63	1060.82	0.61	Terrestrial	Dense forest	Non-endemic
Tropidia curculigoides	Jinghong	840	20.68	1161.08	80.46	1256.19	1.08	Terrestrial	Dense forest	Non-endemic
Tropidia nipponica	Pingbian	1900	13.42	1648.57	86.34	990.16	0.60	Terrestrial	Dense forest	Non-endemic
Tulotis fuscescens	Pingbian	1700	14.62	1648.57	86.34	990.16	0.60	Terrestrial	Dense forest	Non-endemic
Uncifera acuminata	Xichou	1300	17.13	1267.54	82.98	985.97	0.78	Epiphyte	Dense forest	Non-endemic
Uncifera acuminata	Xichou	1300	17.13	1267.54	82.98	985.97	0.78	Epiphyte	Dense forest	Non-endemic
Uncifera acuminata	Pingbian	1200	17.62	1648.57	86.34	990.16	0.60	Epiphyte	Sparse forest	Non-endemic
Uncifera acuminata	Pingbian	1800	14.02	1648.57	86.34	990.16	0.60	Epiphyte	Dense forest	Non-endemic
Uncifera acuminata	Wenshan	2250	12.16	988.87	76.70	1272.98	1.29	Epiphyte	Dense forest	Non-endemic
Vanda brunnea	Mengla	800	20.40	1514.70	84.25	1146.68	0.76	Epiphyte	Forest edge	Non-endemic
Vanda brunnea	Menghai	1350	17.46	1314.38	80.83	1150.91	0.88	Epiphyte	Dense forest	Non-endemic
Vanda brunnea	Luxi	1600	15.48	1650.33	79.33	1183.39	0.72	Epiphyte	Dense forest	Non-endemic
Vanda brunnea	Zhenkang	1640	15.00	1602.96	81.14	1089.09	0.68	Epiphyte	Dense forest	Non-endemic
Vanda brunnea	Luxi	1650	15.18	1650.33	79.33	1183.39	0.72	Epiphyte	Dense forest	Non-endemic
Vanda brunnea	Xinping	1950	14.70	957.31	74.08	1291.86	1.35	Epiphyte	Dense forest	Non-endemic
Vanda coerulea	Mengla	900	19.80	1514.70	84.25	1146.68	0.76	Epiphyte	Dense forest	Non-endemic
Vanda coerulea	Menghai	1150	18.66	1314.38	80.83	1150.91	0.88	Epiphyte	Forest edge	Non-endemic
Vanda coerulea	Jinghong	1600	16.12	1161.08	80.46	1256.19	1.08	Epiphyte	Dense forest	Non-endemic
Vanda coerulescens	Yuanyang	300	24.51	1100.28	77.18	1242.17	1.13	Epiphyte	Dense forest	Non-endemic
Vanda coerulescens	Jinghong	530	22.54	1161.08	80.46	1256.19	1.08	Epiphyte	Dense forest	Non-endemic
Vanda coerulescens	Jinghong	850	20.62	1161.08	80.46	1256.19	1.08	Epiphyte	Dense forest	Non-endemic
Vanda coerulescens	Jinghong	890	20.38	1161.08	80.46	1256.19	1.08	Epiphyte	Dense forest	Non-endemic
Vanda coerulescens	Yongde	1000	21.13	1266.25	69.00	1283.63	1.01	Epiphyte	Dense forest	Non-endemic
Vanda coerulescens	Yongde	1000	21.13	1266.25	69.00	1283.63	1.01	Epiphyte	Dense forest	Non-endemic
Vanda coerulescens	Lancang	1100	19.10	1596.50	77.88	1183.80	0.74	Epiphyte	Dense forest	Non-endemic
Vanda coerulescens	Zhenkang	1100	18.24	1602.96	81.14	1089.09	0.68	Epiphyte	Dense forest	Non-endemic
Vanda coerulescens	Jinghong	1100	19.12	1161.08	80.46	1256.19	1.08	Epiphyte	Sparse forest	Non-endemic
Vanda coerulescens	Yongde	1200	19.93	1266.25	69.00	1283.63	1.01	Epiphyte	Dense forest	Non-endemic

Vanda coerulescens	Yongde	1300	19.33	1266.25	69.00	1283.63	1.01	Epiphyte	Dense forest	Non-endemic
Vanda coerulescens	Lancang	1400	17.30	1596.50	77.88	1183.80	0.74	Epiphyte	Dense forest	Non-endemic
Vanda concolor	Funing	900	18.22	1161.58	79.07	1147.17	0.99	Epiphyte	Dense forest	Non-endemic
Vanda concolor	Xichou	1000	18.93	1267.54	82.98	985.97	0.78	Epiphyte	Dense forest	Non-endemic
Vanda concolor	Malipo	1100	17.66	1063.54	85.83	1053.97	0.99	Epiphyte	Dense forest	Non-endemic
Vanda concolor	Jianshui	1430	17.97	793.10	71.73	1413.81	1.78	Epiphyte	Dense forest	Non-endemic
Vanda concolor	Wenshan	1500	16.66	988.87	76.70	1272.98	1.29	Epiphyte	Dense forest	Non-endemic
Vanda concolor	Lancang	1500	16.70	1596.50	77.88	1183.80	0.74	Epiphyte	Shrubland	Non-endemic
Vanda concolor	Menghai	1550	16.26	1314.38	80.83	1150.91	0.88	Epiphyte	Dense forest	Non-endemic
Vanda cristata	Zhenkang	1465	16.05	1602.96	81.14	1089.09	0.68	Epiphyte	Shrubland	Non-endemic
Vanda denisoniana	Jinghong	890	20.38	1161.08	80.46	1256.19	1.08	Epiphyte	Dense forest	Non-endemic
Vanda pumila	Jinping	530	22.31	2305.17	83.81	1030.46	0.45	Epiphyte	Dense forest	Non-endemic
Vanda pumila	Jinghong	1000	19.72	1161.08	80.46	1256.19	1.08	Epiphyte	Dense forest	Non-endemic
Vanda pumila	Jinghong	1100	19.12	1161.08	80.46	1256.19	1.08	Epiphyte	Dense forest	Non-endemic
Vanda pumila	Menghai	1180	18.48	1314.38	80.83	1150.91	0.88	Epiphyte	Dense forest	Non-endemic
Vanda pumila	Menghai	1400	17.16	1314.38	80.83	1150.91	0.88	Epiphyte	Sparse forest	Non-endemic
Vanda pumila	Menghai	1530	16.38	1314.38	80.83	1150.91	0.88	Epiphyte	Dense forest	Non-endemic
Vanda pumila	Jinping	1700	15.29	2305.17	83.81	1030.46	0.45	Epiphyte	Dense forest	Non-endemic
Vanda pumila	Menghai	1800	14.76	1314.38	80.83	1150.91	0.88	Epiphyte	Dense forest	Non-endemic
Vanda teres	Mengla	570	21.78	1514.70	84.25	1146.68	0.76	Epiphyte	Dense forest	Non-endemic
Vandopsis gigantea	Jinghong	650	21.82	1161.08	80.46	1256.19	1.08	Epiphyte	Dense forest	Non-endemic
Vandopsis gigantea	Jinghong	1100	19.12	1161.08	80.46	1256.19	1.08	Epiphyte	Dense forest	Non-endemic
Vandopsis gigantea	Menghai	1500	16.56	1314.38	80.83	1150.91	0.88	Epiphyte	Dense forest	Non-endemic
Vandopsis undulata	Jingdong	1500	16.58	1128.40	76.80	1142.11	1.01	Epiphyte	Shrubland	Non-endemic
Vandopsis undulata	Jingdong	1600	15.98	1128.40	76.80	1142.11	1.01	Epiphyte	Dense forest	Non-endemic
Vandopsis undulata	Tengchong	1850	13.90	1501.45	78.06	857.93	0.57	Epiphyte	Dense forest	Non-endemic
Vandopsis undulata	Jingdong	1900	14.18	1128.40	76.80	1142.11	1.01	Epiphyte	Dense forest	Non-endemic
Vandopsis undulata	Gongshan	2000	12.08	1738.42	78.47	860.43	0.50	Epiphyte	Dense forest	Non-endemic
Vandopsis undulata	Jingdong	2200	12.38	1128.40	76.80	1142.11	1.01	Epiphyte	Dense forest	Non-endemic
Vandopsis undulata	Gongshan	2300	10.28	1738.42	78.47	860.43	0.50	Epiphyte	Dense forest	Non-endemic
Vandopsis undulata	Yongde	2300	13.33	1266.25	69.00	1283.63	1.01	Epiphyte	Dense forest	Non-endemic
Vandopsis undulata	Fugong	2420	9.54	1441.43	79.99	906.18	0.63	Epiphyte	Dense forest	Non-endemic
Vanilla siamensis	Jinghong	660	21.76	1161.08	80.46	1256.19	1.08	Epiphyte	Dense forest	Non-endemic
Vanilla siamensis	Menghai	850	20.46	1314.38	80.83	1150.91	0.88	Epiphyte	Sparse forest	Non-endemic
Vanilla siamensis	Jinghong	1100	19.12	1161.08	80.46	1256.19	1.08	Epiphyte	Dense forest	Non-endemic
Zeuxine affinis	Jinghong	560	22.36	1161.08	80.46	1256.19	1.08	Terrestrial	Dense forest	Non-endemic

Zeuxine affinis	Jinghong	580	22.24	1161.08	80.46	1256.19	1.08	Terrestrial	Dense forest	Non-endemic
Zeuxine affinis	Mengla	700	21.00	1514.70	84.25	1146.68	0.76	Terrestrial	Dense forest	Non-endemic
Zeuxine affinis	Jinghong	800	20.92	1161.08	80.46	1256.19	1.08	Terrestrial	Shrubland	Non-endemic
Zeuxine affinis	Mengla	1150	18.30	1514.70	84.25	1146.68	0.76	Terrestrial	Forest edge	Non-endemic
Zeuxine affinis	Jinghong	1180	18.64	1161.08	80.46	1256.19	1.08	Terrestrial	Shrubland	Non-endemic
Zeuxine affinis	Zhenkang	1640	15.00	1602.96	81.14	1089.09	0.68	Terrestrial	Dense forest	Non-endemic
Zeuxine affinis	Yongde	2230	13.75	1266.25	69.00	1283.63	1.01	Terrestrial	Forest edge	Non-endemic
Zeuxine Goodyeroides	Xichou	1000	18.93	1267.54	82.98	985.97	0.78	Terrestrial	Dense forest	Non-endemic
Zeuxine Goodyeroides	Wenshan	1200	18.46	988.87	76.70	1272.98	1.29	Terrestrial	Dense forest	Non-endemic
Zeuxine Goodyeroides	Funing	1270	16.00	1161.58	79.07	1147.17	0.99	Terrestrial	Dense forest	Non-endemic
Zeuxine Goodyeroides	Pingbian	1400	16.42	1648.57	86.34	990.16	0.60	Terrestrial	Dense forest	Non-endemic
Zeuxine Goodyeroides	Pingbian	1400	16.42	1648.57	86.34	990.16	0.60	Terrestrial	Grassy slope	Non-endemic
Zeuxine Goodyeroides	Luchun	1600	17.10	2013.54	78.64	1151.56	0.57	Terrestrial	Dense forest	Non-endemic
Zeuxine Goodyeroides	Pingbian	1820	13.90	1648.57	86.34	990.16	0.60	Terrestrial	Dense forest	Non-endemic
Zeuxine Goodyeroides	Luchun	2500	11.70	2013.54	78.64	1151.56	0.57	Terrestrial	Dense forest	Non-endemic
Zeuxine nervosa	Tengchong	1200	17.80	1501.45	78.06	857.93	0.57	Terrestrial	Dense forest	Non-endemic
